# Cu-Catalyzed Chemoselective Borylcupration of Borylated (*Z*)‑Skipped Dienoates: A Case Study for the Synthesis of *gem*-diborylcyclobutanes

**DOI:** 10.1021/acscatal.5c02260

**Published:** 2025-07-01

**Authors:** Mireia Pujol, Gerard Bru, Taras Mazuryk, Anika Tarasewicz, Jorge J. Carbó, María Méndez, Elena Fernández

**Affiliations:** † Faculty of Chemistry, 16777 University Rovira i Virgili, 43007 Tarragona, Spain; ‡ Integrated Drug Discovery, Industriepark Höchst, Sanofi R&D, Bldg. G838, 65926 Frankfurt am Main, Germany

**Keywords:** Cu-catalysis, borylated (*Z*)-skipped dienoate, alkylidene *gem*-diborylcyclobutane, DFT studies, ring closing

## Abstract

Chemoselective borylcupration of borylated (*Z*)-skipped dienoates is controlled by the ester group to access 3,3-di­(pinacol)­borylalkenoates. Electrophilic trapping with H^+^, D^+^, alkyl-, benzyl-, or allyl halides, as well as isocyanates has proved to be efficient for α-functionalized products. The Cu-catalyzed borylcupration of skipped dienoates containing C–Br bonds resulted in concomitant ring closing sequences toward alkylidene *gem*-diborylcyclobutane scaffolds. We performed DFT calculations to characterize the reaction mechanism of the formation of *gem*-diborylcyclobutanes. The key steps of the proposal comprise a selective borylcupration directed by alkene substituents, followed by an intramolecular C–C coupling toward strained four-membered rings assisted by the potassium cation. We also analyzed the effect of the nature of the halogen leaving group on the selectivity. The versatility of alkylidene cyclobutanes has been demonstrated through postfunctionalization reactions.

## Graphical abstract

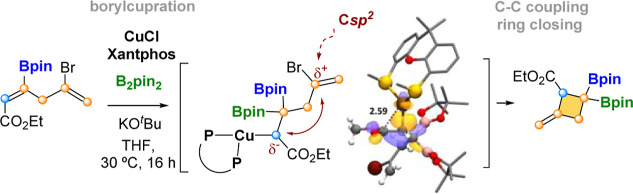

## Introduction

The shape and size of molecular rings are intimately linked to their physical and chemical properties. Three- and four-membered rings are considered small rings that attract significant attention in medicinal chemistry for their beneficial physicochemical properties, which can lead to improved ADME (absorption, distribution, metabolism, and excretion) profiles.^1^ The planar conformation of these small cyclic scaffolds correlates with the **angle strain** concept, in which the four bonds around the sp^3^-hybridized carbons are forced out of their preferred tetrahedral angles. The efforts to construct functionalized aliphatic cyclopropane and cyclobutane rings are justified since they enable better assessment of their value to drug discovery programs.^2^

The installation of boryl moieties on the periphery of the cyclopropane^3^ and cyclobutane^4^ rings has enriched the functional properties of these small rings, due to their unique reactivity and divergent synthetic capability along the defined vectors. In that context, copper catalysis has become one of the most powerful approaches to synthesize borylcyclobutanes by installing boron using borylcupration methods on π-systems, with concomitant intramolecular cyclization.^5^ We recently described that the catalytic system based on Cu­(I)-Xantphos conducts regioselective borylcupration of borylated skipped (*Z*)-dienes, in the presence of bis­(pinacolato)­diboron (B_2_pin_2_), generating the alkylcopper species that suffers stereospecific B/Cu 1,3-rearrangement by remote B shift from C­(sp^2^) to C­(sp^3^) (Scheme 1a).^6^ The boryl migration occurs via a four-membered boracycle intermediate, followed by in situ electrophilic trapping with I_2_. Subsequent palladium-catalyzed regioselective intramolecular cross-coupling generates alkylidene 3-(pinacolboryl)­cyclobutane **A** (Scheme 1a), considered highly strained yet stable molecules found in biologically active natural products.^7^ This approach involved two steps and two catalysts: CuCl/Xantphos for borylcupration/1,3-migration (followed by electrophilic trapping with I_2_) and Pd­(OAc)_2_/RuPhos for regioselective cyclization (Scheme 1a).

**Scheme 1:**
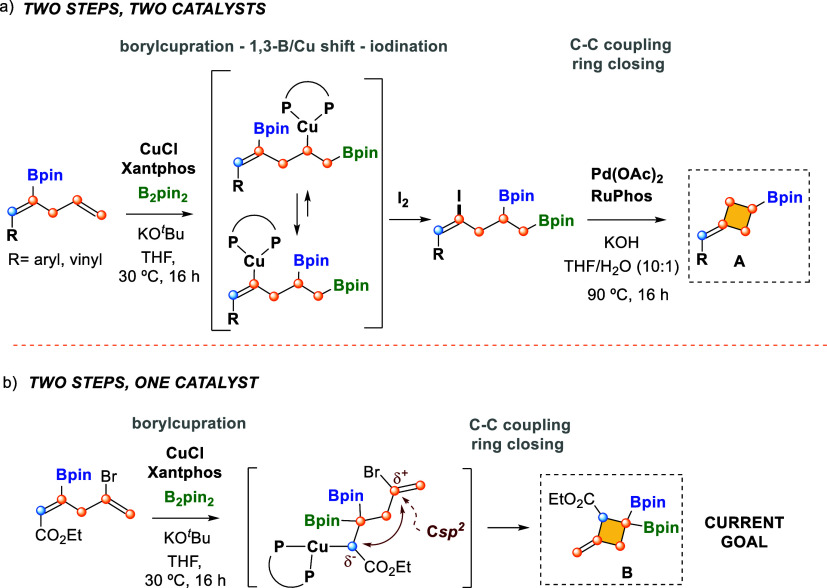
Synthesis of Functionalized Mono- and Diborylated Alkylidene Cyclobutanes

Interested in the benefits of copper catalyzed borylation/ring closing reactions,^8^ we envisioned the development of a catalytic method that would enable the synthesis of alkylidene 3,3′-bis­(pinacolboryl)­cyclobutane **B** (Scheme 1b), where the chemoselective borylcupration of borylated skipped (*Z*)-dienes might be controlled by a terminal ester group, followed by concomitant intramolecular Cu-assisted C–C coupling with the terminal vinyl halide moiety.

Our aim is to develop a new catalytic methodology to construct strained alkylidene *gem*-diborylcyclobutane scaffolds, which have been unreported except for the uncatalyzed reaction between bis­(catecholato)­diboron (B_2_cat_2_) and propellane (Scheme 2a).^9a^ The synthesis of cyclobutanes containing geminal diboryl moieties has been an elusive goal until Aggarwal and co-workers demonstrated the isolation of *gem*-diborylcyclobutane **I** through iridium-catalyzed allylation-induced 1,2-metalate rearrangement of bicyclo[1.1.0]­butyl (BCB) boronate complexes (Scheme 2b).^9b^ More recently, an energy-transfer strategy for photosensitized [2 + 2]-cycloadditions of 1,1-diborylalkenes or dienes, with olefins, enabled the regioselective synthesis of polyfunctionalized *gem*-diborylcyclobutanes **II** and **III**, as described by Masarwa^9c^ as well as Funes-Ardoiz and Molloy^9d^ (Scheme 2c,d). The lack of examples to synthesize *gem*-diborylcyclobutane synthons contrasts with the well stablished protocols for the preparation of *gem*-diborylcyclopropanes.^10^

**Scheme 2:**
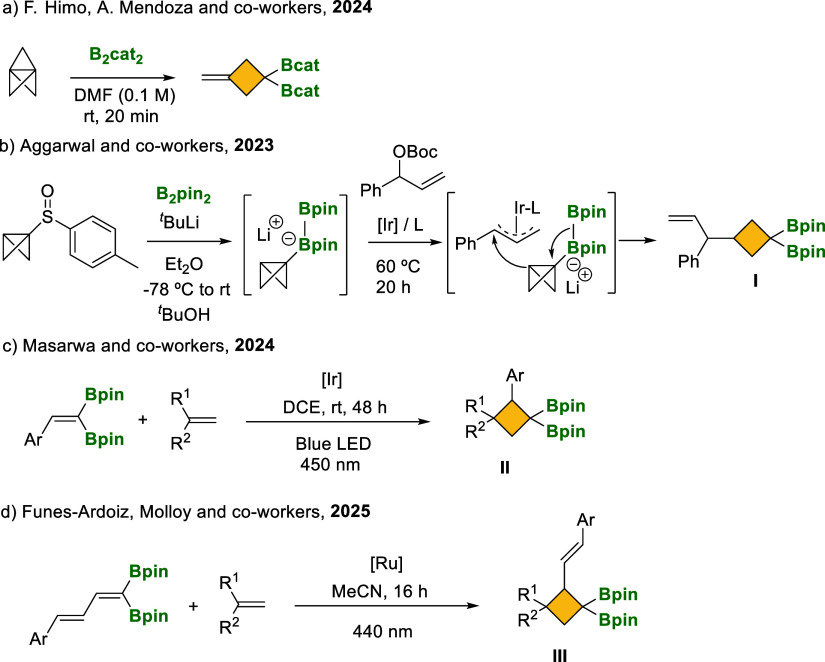
Synthesis of *gem*-Diborylated Cyclobutanes

## Results and Discussion

### Reaction Development

Focused on the synthesis of alkylidene *gem*-diborylcyclobutane scaffolds, we selected the borylated (*Z*)-skipped dienoate model substrate, ethyl (*Z*)-3-pinacolboryl-2,5-hexadienoate (**2**), to conduct a preliminary systematic study of selective borylcupration.^11^ Substrate **2** was prepared from copper-catalyzed stereoselective C–B activation of β,β-diboryl acrylate **1,** followed by allylic alkylation with 3-bromoprop-1-ene, in the presence of K_3_PO_4_ at 60 °C (Scheme 3a).^12^ Next, we conducted the borylcupration of **2** with 10 mol % of CuCl/Xantphos, in the presence of 1.2 equiv of B_2_pin_2_ and KO^*t*^Bu as a base, at 30 °C, in THF (Scheme 3b). Although borylcupration could have reacted with the less sterically hindered π-system of the skipped substrate,^6,13^ we proved that the electronic deficiency of the internal alkene controlled the borylcupration in a complete chemoselective way, leading to interesting product **3** containing a tetrasubstituted carbon. Formally, ethyl 3,3-di­(pinacol)­borylhex-5-enoate (**3**) was synthesized by Cu-catalyzed β-boration of **2** and subsequent electrophilic trapping with H^+^, in a high isolated yield (83%, Scheme 3b). We also explored the *one-pot* two-step protocol, and diborated product **3** could be isolated from β,β-diboryl acrylate **1** in moderate yield (68%, Scheme 3c), avoiding isolation of **2**. It is worthy to mention that whereas the synthesis of *gem*-diboryl alkanes is well established,^14^ the preparation of 3,3-diboryl carboxyesters is understudied.^15^ The use of bis­(neopenthyl glycolato)­diboron (B_2_neo_2_) and (4S,4′S,5S,5′S)-4,4′,5,5′-tetraphenyl-2,2′-bi­(1,3,2-dioxaborolane) (B_2_pai_2_) proved the formation of mixed diboron products **3-Bneo** and **3-Bpai** in a diastereoselective manner (Scheme 3c).

**Scheme 3:**
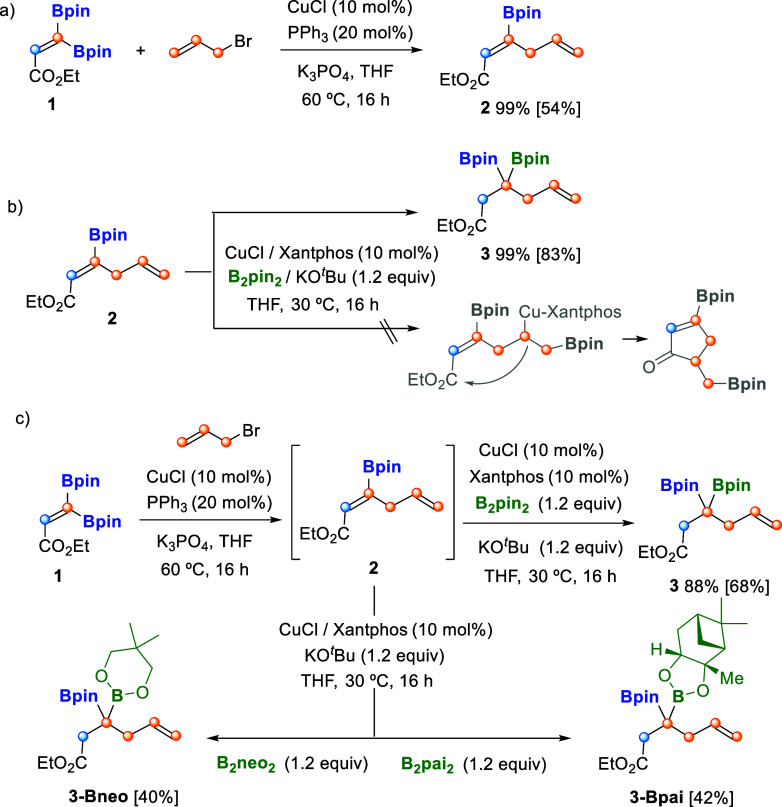
Cu-Catalyzed Synthesis of Borylated (*Z*)-Skipped Dienoate **2** and Ehyl 3,3-Diborylhex-5-Enoate **3** ^a^

a General conditions. Synthesis of **2**: **1** (0.2 mmol), allyl halide (1.5 equiv), CuCl (10 mol %), PPh_3_ (20 mol %), K_3_PO_4_ (2 equiv), THF (4 mL), 60 °C, 16 h. Synthesis of **3**: **2** (0.2 mmol), diboron (1.2 equiv), CuCl (10 mol %), Xantphos (10 mol %), KO^*t*^Bu (1.2 equiv), THF (4 mL), 30 °C, 16 h. Yields determined by NMR with naphthalene as internal standard and isolated yields in brackets.

Next, we explored the compatibility of the copper-catalyzed β-boration of a series of borylated (*Z*)-skipped dienoate substrates, modifying the substituents along the diene system (Scheme 4). Product **4** was isolated in 70% yield proving the chemoselective Cu-catalyzed borylcupration on the α,β-unsaturated ester, despite the fact that difluoro substituents enhanced the electron deficiency of the terminal alkene (Scheme 4). Complementarily, 3,3-di­(pinacol)­borylalkenoates **5–7** were synthesized in moderate to high isolated yields, demonstrating compatibility with sterically hindered borylated (*Z*)-skipped dienoates (Scheme 4). Alternative electrophilic trapping was next studied, involving MeOD for the synthesis of deuterated product **8** in a 76% isolated yield (Scheme 4). When MeI or BnI were used to trap the intermediate after the borylcuprartion of **2**, products **9** and **10** were easily prepared and isolated, demonstrating the compatibility of C–C bond formation with alkyl groups at the α position (Scheme 4). Eventually, the electrophilic trapping with allyl bromides resulted in the formation of functionalized 1,7-diene systems **11–13** (Scheme 4). In particular, the reaction of **2** with 3-bromo-3,3-difluoroprop-1-ene allowed for the formation of the difluorinated 1,7-diene product **12,** suggesting that the C–C coupling might proceed through a S_N_2′ mechanism (Scheme 4). However, the coupling between **2** and (*E*)-1,4-dibromobut-2-ene generated exclusively the α-selective product **13**, versus the γ-selective isomer, suggesting the S_N_2 mechanistic pathway renders the less sterically hindered α-substituted 3,3-di­(pinacol)­borylalkenoate.

**Scheme 4:**
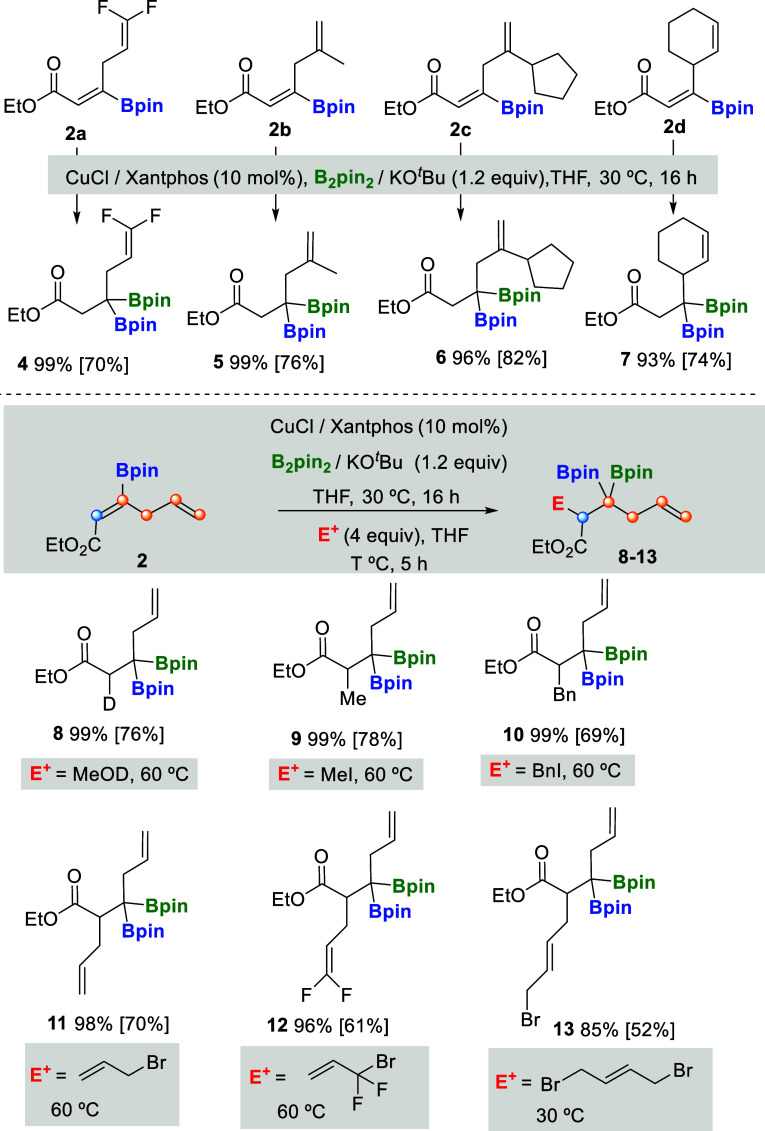
Cu-Catalyzed Borylcupration/Electrophilic Trapping of Borylated (*Z*)-Skipped Dienoates^a^

a General conditions. Borylcupration: (*Z*)-skipped diene (0.2 mmol), B_2_pin_2_ (1.2 equiv), CuCl (10 mol %), Xantphos (10 mol %), KO^*t*^Bu (1.2 equiv), THF (4 mL), 30 °C, 16 h. Electrophilic trapping: E^+^ (4 equiv), THF (2 mL), T = 30 °C–60 °C, 5 h. Yields determined by NMR with naphthalene as internal standard and isolated yields in brackets.

When we used isocyanates as electrophilic reagents to be trapped after the borylcupration of **2**, we observed the formation of the corresponding amide group, independently of the isocyanate used, generating the arylamides **14–17**, alkylamide **18,** or benzyl amide **19** in moderate isolated yields (Scheme 5). Despite the usefulness of isocyanates for the generation of a wide range of amides, their role as electrophiles in borylcupration of alkenes has only been reported to transform vinyl arenes into boryl alkyl amides.^16^ We found that our methodology allows for a straightforward access to *N*H-*tert*-butyl malonamide and *N*H-*aryl* malonamides as an alternative platform for β-lactam synthesis.^17^ The overall process from β,β-diborylacrylate **1** formally results in the regioselective functionalization of the internal double bond, generating two vicinal tetrasubstituted and trisubstituted carbons.

**Scheme 5:**
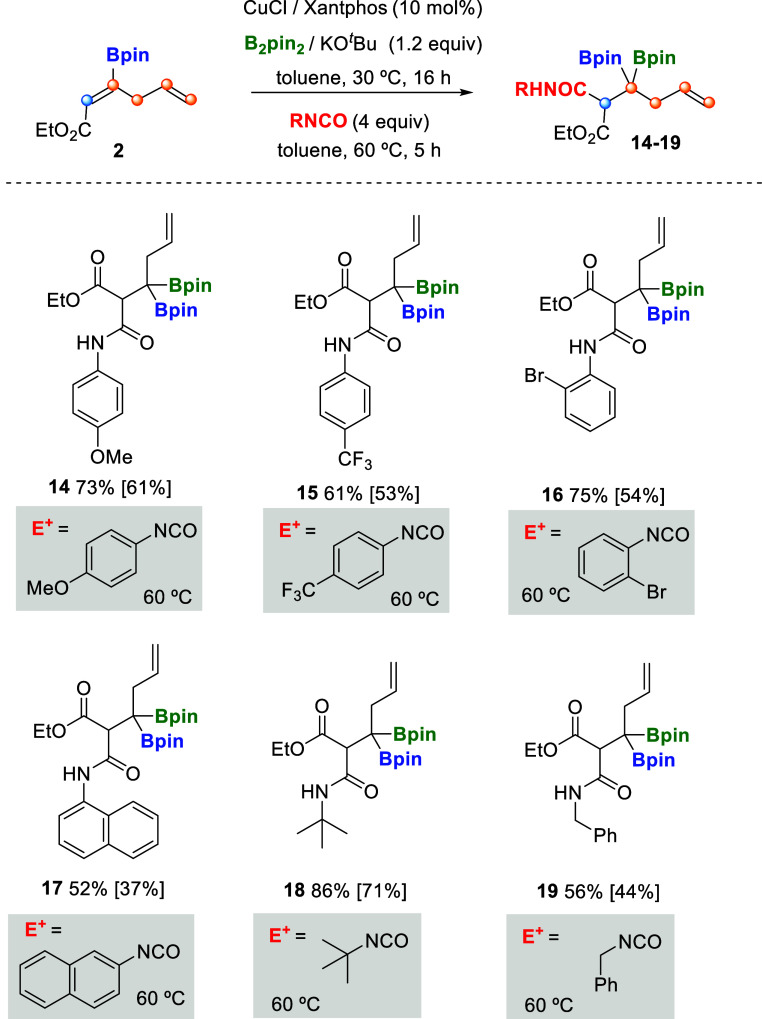
Cu-Catalyzed Borylcupration/Electrophilic Trapping with Isocyanates^a^

a General conditions. Borylcupration: (*Z*)-skipped diene (0.2 mmol), B_2_pin_2_ (1.2 equiv), CuCl (10 mol %), Xantphos (10 mol %), KO^*t*^Bu (1.2 equiv), toluene (4 mL), 30 °C, 16 h. Electrophilic trapping: RNCO (4 equiv), toluene (2 mL), T = 60 °C, 5 h. Yields determined by NMR with naphthalene as internal standard and isolated yields in brackets.

By applying the Cu-catalyzed synthesis of borylated (*Z*)-skipped dienoates, but using (*E*)-1,4-dibromobut-2-ene, we could obtain diene **20** (Scheme 6a),^12^ which led to new reactivity. Under the optimized reaction conditions (10 mol % of CuCl/Xantphos, 1.2 equiv of B_2_pin_2_ and KO^*t*^Bu as a base, at 30 °C in THF or toluene), we observed the formation of compound **21** containing a three-membered ring with an exclusive *cis* stereoselectivity of the substituents (Scheme 6b). This highly functionalized cyclopropane has not been reported before, and only related compounds have been described in recent synthesis of boronate ester bullvalenes.^18^

**Scheme 6:**
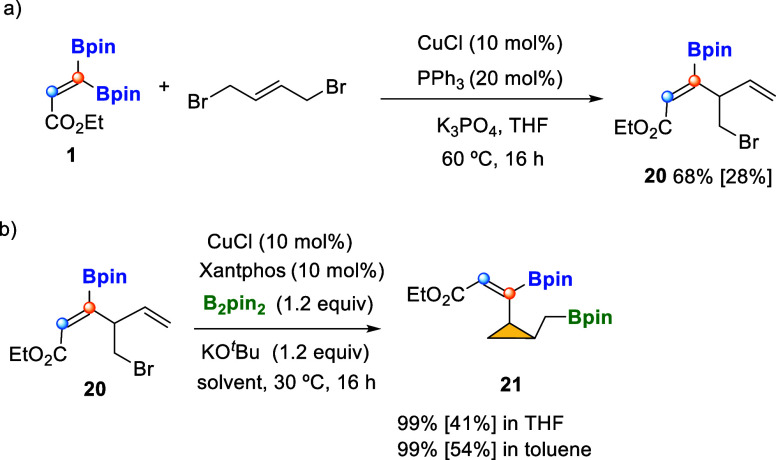
Cu-Catalyzed Synthesis of Ethyl (*Z*)-4-(bromomethyl)-3-(pinacolboryl)­hexa-2,5-dienoate **20** and Substituted Cyclopropane **21** ^a^

a General conditions. Synthesis of **20**: 1,1-diborylalkene **1** (0.2 mmol), (*E*)-1,4-dibromobut-2-ene (1.5 equiv), CuCl (10 mol %), PPh_3_ (20 mol %), K_3_PO_4_ (2 equiv), THF (4 mL), 60 °C, 16 h. Synthesis of **21**: (*Z*)-skipped diene **20** (0.2 mmol), B_2_pin_2_ (1.2 equiv), CuCl (10 mol %), Xantphos (10 mol %), KO^*t*^Bu (1.2 equiv), THF or toluene (4 mL), 30 °C, 16 h. Yields determined by NMR with naphthalene as internal standard and isolated yields in brackets.

Similarly, the skipped diene **22** was prepared from **1** and 2,3-dibromoprop-1-ene, throughout Cu-catalyzed site-selective C-Bpin activation (Scheme 7a). When we conducted the Cu/Xantphos-catalyzed borylcupration of substrate **22**, and KO^*t*^Bu as a base, at 30 °C in THF, we proved that electronic deficiency of the internal alkene controlled the borylcupration in a complete chemoselective way, generating the expected product **23**, although the formation of the alkylidene 3,3′-bis­(pinacolboryl)­cyclobutane **24** was also observed in 23% yield (Scheme 7b). The replacement of KO^*t*^Bu, as a base, by NaO^*t*^Bu or LiO^*t*^Bu did not improve the ratio on product **24**. However, when the reaction mixture was heated to 60 °C, we noted a preferred formation of the cyclic product, with a ratio **23**/**24** = 37/63 (Scheme 7b). The use of toluene, instead of THF, did not favor the formation of the cyclic product, and neither did the use of PPh_3_ and PCy_3_ as ligands (Scheme 7b). Interestingly, the use of PBu_3_ led to the formation of **24** with the highest ratio of **23**/**24** = 30/70 and 42% isolated yield (Scheme 7b).

**Scheme 7:**
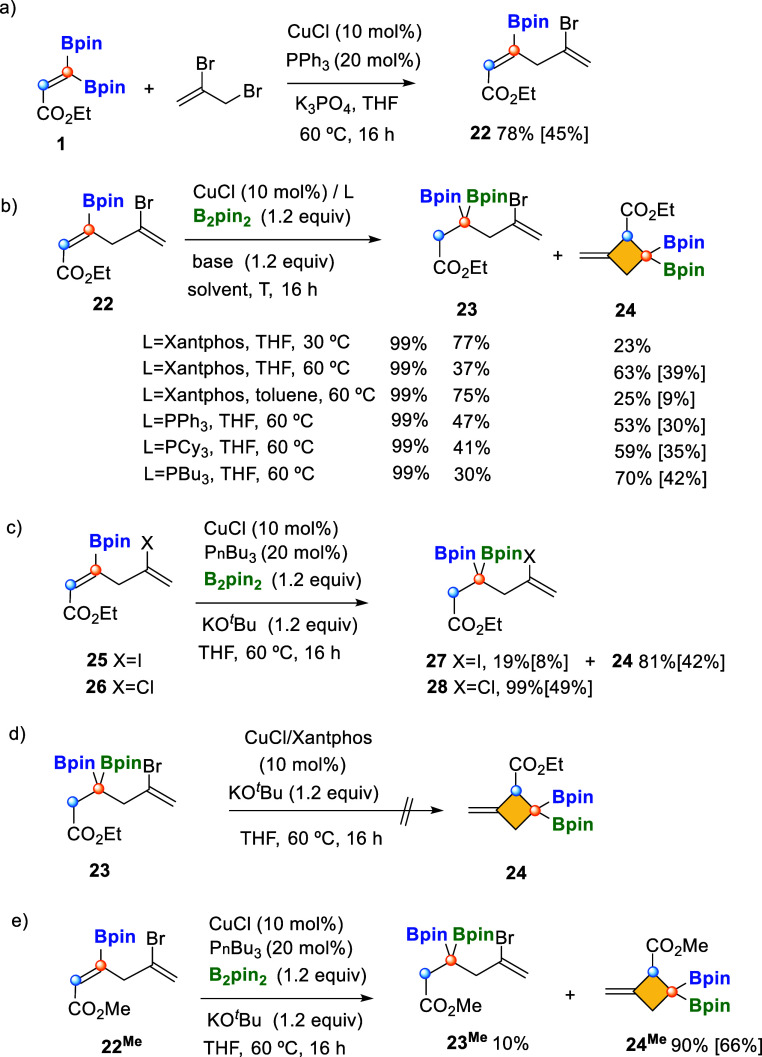
Synthesis of Skipped Dienoate **22** and Cu-Catalyzed Borylcupration with Concomitant Ring Closing Sequence^a^

a General conditions. Synthesis of **22**: 1,1-diborylalkene **1** (0.2 mmol), 2,3-dibromoprop-1-ene (1.5 equiv), CuCl (10 mol %), PPh_3_ (20 mol %), K_3_PO_4_ (2 equiv), THF (4 mL), 60 °C, 16 h. Borylcupration of **22**: (*Z*)-skipped diene **22** (0.2 mmol), B_2_pin_2_ (1.2 equiv), CuCl (10 mol %), Xantphos (10 mol %), KO^*t*^Bu (1.2 equiv), THF or toluene (4 mL), 30 or 60 °C, 16 h. Yields determined by NMR with naphthalene as internal standard and isolated yields in brackets.

We studied the influence of the halide X = Br versus X = I, Cl toward the cyclization pathway. Substrates **25** and **26** were prepared and submitted to the borylcupration under optimized reaction conditions. Whereas **25** (X = I) evolved toward the cyclic product **24** similarly to substrate **22** (Scheme 7c), the use of **26** (X = Cl) inhibited the formation of the cyclic product (Scheme 7c). The alkylidenecyclobutane **24** shows a relative disposition of the substituents that results complementary to all other reported methods.^9^ To understand the formation of the intramolecular cyclic product **24**, we explored the plausible transformation of compound **23** into **24** under the same Cu-catalyzed reaction conditions (10 mol % of CuCl/Xantphos, 1.2 equiv of B_2_pin_2_ and KO^*t*^Bu as a base, at 60 °C in THF); however, we did not observe any transformation from **23** to the cyclic product **24** (Scheme 7d). The formation of four-membered-ring compound **24** ^**Me**^ from skipped diene **22** ^**Me**^ has also been demonstrated, extending the generality of this method (Scheme 7e).

### Mechanistic Studies

To understand the copper-catalyzed borylation/ring closing reaction mechanism, we performed DFT calculations^19^ on the formation of four-membered ring compound **24** ^**Me**^ from skipped diene **22** ^**Me**^. Figure 1 shows the free-energy profile for the proposed mechanism, which shares some features with our previous computational studies on copper-catalyzed borylative ring closing reactions.^8a,8c^ The mechanism starts with the reaction between the CuCl/Xantphos precatalyst with KO^*t*^Bu to form the active catalytic species **I**_**1**_ leaving KCl as a side product.^6,8a,8c,20^ In the next step, diboron B_2_pin_2_ reacts with **I**_**1**_ to produce the Cu-boryl species **I**_**2**_. This intermediate can coordinate the skipped diene **22** ^**Me**^, forming the stable η^2^-alkene-Cu bond complex **I**_**3**_. Then, 1,2-insertion of the alkene moiety into the Cu–B bond proceeds with the observed regioselectivity through transition state **TS1** with a low free-energy barrier (+7.4 kcal·mol^–1^). The regioselectivity can be rationalized by analyzing the electronic structure of reactants and intermediates. The copper-boryl species behaves as the nucleophile^21^ and attacks an electrophilic moiety of the substrate. In the skipped diene **22** ^**Me**^, the lowest unoccupied molecular orbital (LUMO) corresponds to the π-antibonding orbital of the internal alkene that is stabilized via interaction with the p-type orbitals of the Bpin and the ester substituents (see Figure S4). This internal double bond is polarized toward the boryl-substituted sp^2^ carbon (C1) as reflected by the contribution of the atomic orbitals to the C1=C2 π* NBO orbital (52% for C1 vs 48% for C2). Moreover, the computed free-energy barrier for the borylcupration of the terminal alkene in substrate **22** ^**Me**^ through transition state **TS1t** is significantly higher, the **TS1t** structure laying 11.7 kcal·mol^–1^ higher in free-energy than **TS1** structure (Figure S5). We also noted that this regioselectivity is also observed for substrate **20** (see Figure S5), whose reactivity cannot proceed through four-membered ring closing, as observed for **22** ^**Me**^, because it lacks a bromide leaving group. For **20**, a different mechanism should operate after borylcupration, yielding the unexpected product **21**, but the characterization of this mechanism is out of the scope of this work.

**Figure 1:**
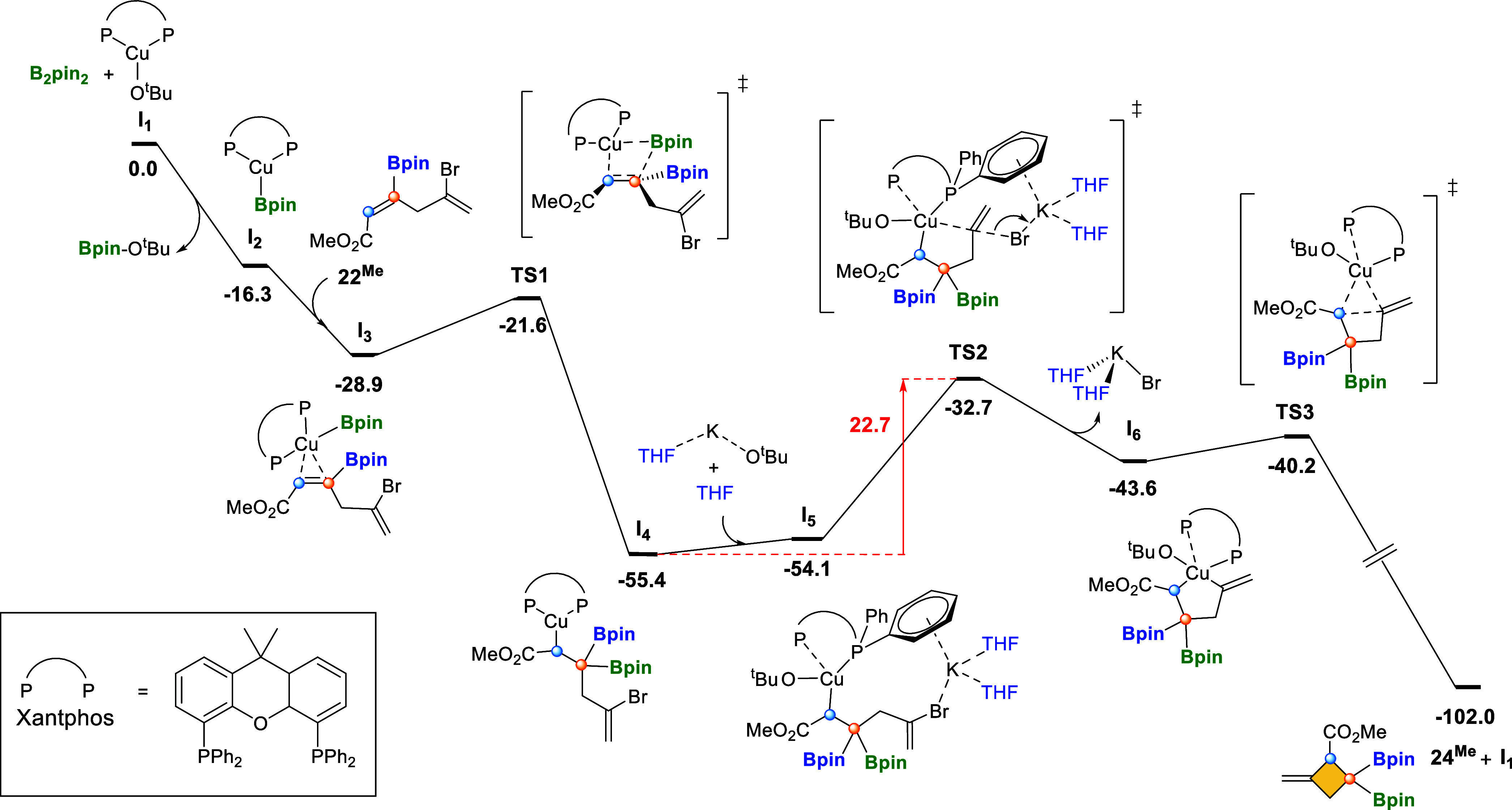
Free-energy profile (kcal·mol^–1^) for the Cu-catalyzed borylcupration with concomitant ring closing.

The intermediate resulting from borylation, **I**_**4**_, contains a *gem*-diboryl fragment and its formation is energetically favorable, −26.5 kcal·mol^–1^ with respect to intermediate **I**_**3**_ (Figure 1). The alkyl ligand in **I**_**4**_ shows some delocalization of the negative charge over the carbonyl group of the ester. On going from the reactant **22** ^**Me**^ to the intermediate **I**_**4**_, the distance of the C=O bond increases from 1.21 to 1.24 Å, while the C–C bond distance with the ester substituent decreases from 1.48 to 1.45 Å. Accordantly, its highest occupied molecular orbital (HOMO) shows the interaction between the Cu d orbitals and the alkyl carbon, with some contribution of p-type orbitals in the ester moiety (Figure S6). This type of interaction is similar to that previously characterized for α-boryl alkyl copper complexes by means of DFT calculations and crystallographic database search.^22^ From intermediate **I**_**4**_, the reaction can evolve to the formation of **23** ^**Me**^ via protonation of the Cu-alkyl bond. Alternatively, as observed experimentally, the reaction can proceed with halogen abstraction and ring closing, assisted by the ^*t*^BuOK base. In this stage, and in line with our previous computational studies,^8a,8c^ the *tert*-butoxide coordinates to Cu (Cu–O distance 2.01 Å), forming an anionic complex with the potassium acting as a countercation, intermediate **I**_**5**_, which is almost isoenergetic to **I**_**4**_. Here, the potassium cation interacts simultaneously with the bromide and the aromatic ring of the phosphine phenyl group, in the presence of two interacting THF molecules to model the explicit solvent.^8a,8b^ From **I**_**5**_, the potassium abstracts the bromide from the C­(sp^2^), assisted by the Cu center, overcoming a free-energy barrier of 22.7 kcal·mol^–1^ (**I**_**4**_ → **TS2**). Remarkably, we could characterize computationally a Cu­(III) intermediate with a Cu-alkenyl and a Cu-alkyl bonds (**I**_**6**_), which is the result of bromide abstraction along with the formation of (THF)_2_·KBr salt. Nevertheless, the Cu­(III) complex is kinetically unstable with a free-energy barrier associated with the reductive elimination and ring closing steps of 3.4 kcal·mol^–1^ (**TS3**). Thus, the bromide abstraction and the ring closing could be viewed as a mostly concerted, irreversible step. Alternatively, we evaluated the oxidative addition of the Csp^2^–Br bond to the Cu­(I) center without the assistance of a potassium cation. Nevertheless, the corresponding free-energy barrier (32.0 kcal·mol^–1^) is significantly higher (Figure S7).

The highest free-energy barrier of the catalytic process corresponds to the energy difference between **TS2** and **I**_**4**_, with a computed value of 22.7 kcal·mol^–1^, which is consistent with a reaction occurring at moderate temperatures. The change of selectivity toward cyclic product **24** ^**Me**^ upon increasing the temperature can be explained as follows: Higher temperatures accelerate the rate of the ring closing pathway to yield **24** ^**Me**^ by increasing the apparent rate constant, while the rate of the protonation pathway to give **23** ^**Me**^ in aprotic solvents is controlled by the concentration of the proton source (water) and is less sensitive to the temperature. Moreover, the rate-determining process involves halide abstraction in key transition state **TS2**, which is also in line with experimental observations. Thus, replacing the bromide with chloride (less effective leaving group) inhibits the formation of the cyclic product **24** with the exclusive observation of the product **28**. For X = Cl, the Δ*G* ^‡^(**I**_**4**_ → **TS2**) free energy barrier (24.8 kcal·mol^–1^) is larger than that for X = Br. On the other hand, when replacing the bromide with iodide (better leaving group), the selectivity improves, and the cyclic product is obtained in a ratio **24**/**27** = 19/81 (Scheme 7b). For X = I, the Δ*G* ^‡^(**I**_**4**_ → **TS2**) free energy barrier (18.6 kcal·mol^–1^) is lower than for X = Br. Overall, this reaction represents a singular intramolecular copper catalyzed cross-coupling of secondary alkylcopper with haloalkenyl moieties, with concomitant ring closing and generation of exocyclic double bond.^23^

### Synthetic Applications

Although in general alkylidene cyclobutanes are known for their enhanced reactivity,^24^ compound **24** has proven to be strikingly stable under several reaction conditions. However, it offers a valuable synthetic tridimensional platform featuring several functional groups in a congested space that can be orthogonally functionalized, as exemplified in Scheme 8. We explored the synthetic application of the alkylidene *gem*-diborylcyclobutane scaffold **24** to prepare spiro compounds conducting the Simmons–Smith cyclopropanation of the exocyclic alkene, under Furukawa conditions.^25^ The *gem*-diborylated spiro compound **29** was afforded in moderate yield, as a 55:45 mixture of both stereoisomers (Scheme 8a), indicating that the ester group had no direct influence on the stereoselectivity. Alternatively, the protodeborylation of **24** took place with NaO^*t*^Bu as the base, with concomitant isomerization of the exocyclic double bond, toward the conjugated product ethyl 2-methyl-4-(pinacolboryl)­cyclobut-1-ene-1-carboxylate (**30**) (Scheme 8b). The site-selective activation of one of the Bpin moieties in alkylidene *gem*-diborylcyclobutane compound **24** was next explored through an homologation pathway, and interestingly, we obtained a single diastereoisomer **31**, where the Bpin *trans* to the ester group was exclusively transformed into primary C-Bpin bond (Scheme 8d). The ester group in **24** seems to protect the vicinal *cis*-Bpin moiety in the homologation sequence. In fact, calculations have shown that coordination of the ^–^O^*t*^Bu base to the Bpin moiety *cis* to the ester is disfavored by 1–2 kcal·mol^–1^ due to the steric repulsion between the ^*t*^Bu and the ester groups (Figure S8). Subsequent treatment of **31** with NaO^*t*^Bu, as a base, proved to be efficient for isomerization of the exocyclic double bond, but the primary boronic ester remained unaltered, isolating product **32** in moderate yield (Scheme 8c).

**Scheme 8:**
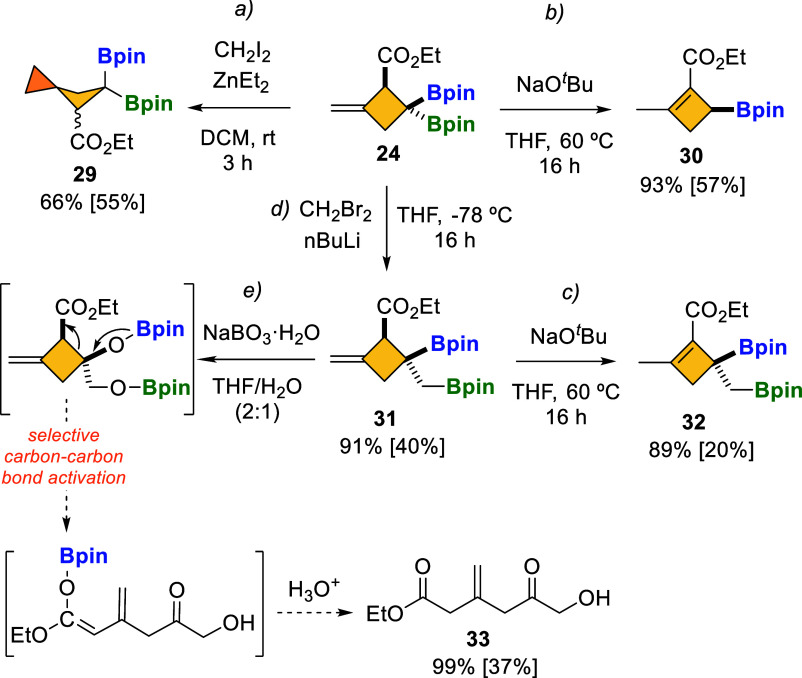
Transformation of Alkylidene *gem*-Diborylcyclobutane **24** ^a^

a General conditions for 0.2 mmol of **24**: a) ZnEt_2_ (0.4 mmol), CH_2_I_2_ (0.6 mmol), DCM (2 mL); b,c) NaO^*t*^Bu (0.3 mmol), THF (0.5 mL); d) nBuLi (0.5 mmol), DCM (0.6 mL); e) NaBO_3_·H_2_O (0.3 mmol), THF (2 mL).

Finally, we conducted the oxidation of the homologated compound **31**, and whereas the primary boronic ester was oxidized toward the corresponding alcohol, the tertiary boronic ester underwent a regioselective ring–opening reaction to provide the versatile boryl homoenolate that was eventually stabilized as the polyfunctionalized compound **33** (Scheme 8e). The oxidation of strained substrates with concomitant carbon–carbon bond cleavage may be difficult to control,^26,27^ but we hypothesized that the ester group could facilitate this regioselective ring opening.

## Conclusions

In summary, we have developed a chemoselective borylcupration of borylated (*Z*)-skipped dienoates, acceding to 3,3-di­(pinacol)­borylalkenoates and opening an electrophilic trapping platform with H^+^, D^+^, alkyl-, benzyl- or allyl halides, and isocyanates for α-functionalized products. Interestingly, for skipped dienoates containing C–Br bonds, the Cu-catalyzed borylcupration conducts a concomitant ring closing sequence toward alkylidene *gem*-diborylcyclobutane scaffolds. The reaction mechanism for the formation of *gem*-diborylcyclobutanes derived from DFT calculations indicates that the selective borylcupration is governed by the electronic features of the alkene substituents. Then, the copper catalyzes the intramolecular cross-coupling of secondary alkylcopper with haloalkenyl moieties, with concomitant ring closing and generation of exocyclic double bonds. The ring closing is assisted by a potassium cation and depends on the nature of the halogen as a leaving group. The postfunctionalizaion of *gem*-diborylcyclobutane scaffolds demonstrates the influence of the ester to protect the Bpin moiety in *cis* configuration, allowing the *trans* Bpin moiety to be homologated or deborylated.

## Methods

### General Procedure for Cu-Catalyzed Borylcupration–Protonation

In a flamed Schlenk-tube equipped with a magnetic stir bar, CuCl (1.98 mg, 10 mol %, 0.02 mmol), diboron reagent (60.9 mg, 1.2 equiv, 0.24 mmol), and Xantphos (277,6 mg, 10 mol %, 0.02 mmol) were placed. The vial was evacuated and backfilled with nitrogen, and THF (1 mL) was added. Next, KO^*t*^Bu (26.9 mg, 1.2 equiv, 0.24 mmol) in THF (1 mL) was poured in the vial through the rubber septum. Then, the borylated (*Z*)-skipped dienoate **2** (1 equiv, 0.2 mmol) in THF (1 mL) was added dropwise at 30 °C. After the reaction was completed, the reaction mixture was filtered over Celite. The solvents were evaporated at the rotatory evaporator and the crude was purified by silica gel chromatography to obtain the desired product **3** in 83% isolated yield (46 mg).

### General Procedure for Cu-Catalyzed Borylcupration–Protonation–Electrophilic Trapping with Alkyl or Allyl Halides

In a flamed Schlenk-tube equipped with a magnetic stir bar, CuCl (1.98 mg, 10 mol %, 0.02 mmol), diboron reagent (60.9 mg, 1.2 equiv, 0.24 mmol), and Xantphos (277,6 mg, 10 mol %, 0.02 mmol) were placed. The vial was evacuated and backfilled with nitrogen, and THF (1 mL) was added. Next, KO^*t*^Bu (26.9 mg, 1.2 equiv, 0.24 mmol) in THF (1 mL) was poured in the vial through the rubber septum. Then, borylated (*Z*)-skipped dienoate **2** (1 equiv, 0.2 mmol) in THF (1 mL) was added dropwise. After being stirred at 30 °C for 16 h, the corresponding alkyl or allyl halide (4 equiv) was added into the reaction at 60 °C for 5 h. After the reaction was completed, the reaction mixture was filtered over Celite. The solvents were evaporated at the rotatory evaporator, and the crude was purified by silica gel chromatography to obtain the desired product.

### Synthesis of Compound **24**

In a flamed Schlenk-tube equipped with a magnetic stir bar, CuCl (1.98 mg, 10 mol %, 0.02 mmol), diboron reagent (60.9 mg, 1.2 equiv, 0.24 mmol), and PnBu_3_ (10 mL, 20 mol %, 0.04 mmol) were placed. The vial was evacuated and backfilled with nitrogen, and THF (1 mL) was added. Next, KO^*t*^Bu (26.9 mg, 1.2 equiv, 0.24 mmol) in THF (1 mL) was poured in the vial through the rubber septum. Then, the borylated (*Z*) skipped dienoate (1 equiv, 0.2 mmol) in THF (1 mL) was added dropwise at 60 °C. After the reaction was completed, the reaction mixture was filtered over Celite. The solvents were evaporated at the rotatory evaporator, and the crude product was purified by silica gel chromatography to obtain the desired product **24** in 42% isolated yield (55 mg).

### Synthesis of Compound **29**

We followed the general procedure for Simmons–Smith cyclopropanation reactions. To a solution of product **24** (78 mg, 0.2 mmol, 1.0 equiv) in anhydrous DCM (2.0 mL, 0.1 M) was added ZnEt_2_ (1.0 M in hexane, 0.4 mL, 0.4 mmol, 2.0 equiv) at 0 °C. After stirring for 10 min, CH_2_I_2_ (161 mg, 0.6 mmol, 3.0 equiv) was added. The mixture was stirred at room temperature, and a white precipitate was gradually generated. After 3 h, the mixture was quenched with saturated aqueous NH_4_Cl (8 mL) and extracted with EtOAc (3 × 10 mL). The combined organic layers were dried with Na_2_SO_4_, filtered through Celite, and concentrated in vacuo. The residue was purified by flash column chromatography on silica gel to give the corresponding product **29** in a 55% isolated yield (39 mg).

### Synthesis of Compound **30**

A Schlenk-tube equipped with a magnetic stir bar was charged with NaO^*t*^Bu (28.83 mg, 3 equiv, 0.3 mmol), product **24** (39.2 mg, 1 equiv, 0.1 mmol), and THF (0.5 mL). The Schlenk-tube was closed with a Teflon cap, and the reaction was stirred for 16 h at 60 °C (oil bath). After the reaction was complete, the reaction mixture was filtered over Celite. The solvents were evaporated at the rotatory evaporator, and the crude product was purified by silica gel chromatography to obtain the desired product **30** in 57% isolated yield (12 mg).

### Synthesis of Compound **31**

A Schlenk-tube equipped with a magnetic stir bar was evacuated and backfilled with N_2_ three times. Then, it was charged with product **24** (78.42 mg, 0.2 mmol, 1.00 equiv) in THF (2.00 mL), dibromomethane (50 μL, 0.64 mmol, 3.2 equiv) was added sequentially via a syringe, and the mixture was cooled to −78 °C in a dry ice/acetone bath. *n*-butyllithium (0.2 mL, 2.5 M in hexanes, 0.5 mmol, 2.5 equiv) was added dropwise via a syringe over 2 min. The reaction was stirred at 78 °C for 1 h and then placed in the freezer at −20 °C for 24 h without stirring. The reaction mixture was warmed to room temperature and quenched with H_2_O (10 mL). The layers were separated, and the aqueous layer was extracted with ethyl acetate (3 × 10 mL); the combined organic layers were dried over MgSO_4_, filtered over Celite. The solvents were evaporated at the rotatory evaporator, and the crude product was purified by silica gel chromatography to obtain the desired product **31** in 40% isolated yield (32 mg).

### Synthesis of Compound **33**

In an opened-air flask, charged with a magnetic stir bar, the alkylidene cyclobutane **31** (40.61 mg, 0.1 mmol, 1 equiv), NaBO_3_·H_2_O (0.3 mmol, 3 equiv), THF (2 mL), and distilled water (1 mL) were added. The reaction was closed with a septum with a needle to avoid overpressure and was stirred for 16 h at room temperature. After this period of time, the mixture was extracted with Et_2_O (3 × 15 mL), the organic layer was dried with anhydrous magnesium sulfate and filtered, and the solvents were evaporated. The crude residue was purified by silica gel chromatography to obtain product **33** in a 37% isolated yield (3 mg).

## Supplementary Material


