# Supplementary material for: Cu-Catalyzed Chemoselective Borylcupration of Borylated (Z)‑Skipped Dienoates: A Case Study for the Synthesis of gem-diborylcyclobutanes
Source: ACS Catal. 2025 Jul 1;15(14):12063–74. doi: 10.1021/acscatal.5c02260 (PMC12309546; doi:10.1021/acscatal.5c02260)
Supplement: Supplementary file 1 [file cs5c02260_si_001.pdf]

Cu catalyzed chemoselective borylcupration of borylated (*Z*)-skipped dienoates: a case study for the synthesis of *gem*-diborylcyclobutanes

Mireia Pujol,[a] Gerard Bru,[a] Taras Mazuryk,[a] Anika Tarasewicz,[b] Jorge J. Carbó,[a]* María Méndez,[b]* Elena Fernández[a]*

[a]*Faculty of Chemistry, University Rovira i Virgili, 43007 Tarragona, Spain.*
[b]*Sanofi R&D, Integrated Drug Discovery, Industriepark Höchst, Bldg. G838, 65926 Frankfurt am Main, Germany.*
mariaelena.fernandez@urv.cat, Maria.MendezPerez@sanofi.com, j.carbo@urv.cat

Contents

-General information 2
-General procedure for the synthesis of β,β-diborylacrylates 3
-General procedure for site-selective Cu activation of β,β-diborylacrylates and nucleophilic allylic coupling: *representative example for synthesis of substrate* ***2*** 3
-General procedure for Cu-catalyzed borylcupration – protonation 4
-General procedure for Cu-catalyzed borylcupration – electrophilic trapping with alkyl or allyl halides 4
-General procedure for Cu-catalyzed borylcupration – electrophilic trapping with isocyanates 5
-Synthesis of compound **21** 5
-Synthesis of compound **24** 6
-Synthesis of compound **29** 6
-Synthesis of compound **30** 7
-General procedure for homologation reaction 7
-Oxidation of alkylidenecyclobutane **31** 8
-Characterization data for borylated (*Z*)-skipped dienoates 8
-Characterization data for products of Cu-catalyzed borylcupration and electrophilic trapping 11
-Characterization data for cyclic and oxidized products 22
-^{1}H, ^{13}C, ^{11}B, ^{19}F NMR spectra 26
-Complementary Reactivity 83
-Computational Studies 87
-References 128

General information

Solvents and reagents: Solvents and reagents were obtained from commercial suppliers and dried and/or purified (if needed) by standard procedures. Diboron reagents were purchased from Ally Chem and used without further purification. All reactions were conducted in oven and flame-dried glassware under an inert atmosphere of argon, using Schlenk-type techniques. *Flash chromatography* was performed on standard silica gel (Merck Kieselgel 60 F254 400-630 mesh). *Thin layer chromatography* was performed on Merck Kieselgel 60 F254 which was developed using standard visualizing agents: UV fluorescence (254 and 366 nm) or potassium permanganate/Δ. *NMR spectra* were recorded at a Varian Goku 400 or a Varian Mercury 400 spectrometer. ^{1}H NMR and ^{13}C{^{1}H} NMR chemical shifts (δ) are reported in ppm with the solvent resonance as the internal standard ($CDCl_3$: 7.26 ppm (^{1}H) and $CDCl_3$: 77.16 ppm (^{13}C)). ^{11}B{^{1}H} NMR chemical shifts (δ) are reported in ppm relative to $(CH_3)_2O\cdots BF_3$. Data are reported as follows: chemical shift, multiplicity (s = singlet, d = doublet, t = triplet, q = quartet, hept = heptuplet, br = broad, m = multiplet), coupling constants (Hz) and integration. *High resolution mass spectra (HRMS)* were recorded using a 6210 Time of Flight (TOF) mass spectrometer from Agilent Technologies (Waldbronn, Germany) with an ESI interface and it was performed at the Servei de Recursos Científics i Tècnics (Universitat Rovira i Virgili, Tarragona) or using a BIOTOF II Time of Flight (TOF) mass spectrometer from Bruker with an APCI interface or EI interface and it was performed at the Unidade de Espectrometría de Masas e Proteómica (Universidade de Santiago de Compostela, Santiago de Compostela). GC-MS analyses were performed on a HP6890 gas chromatograph and an Agilent Technologies 5973 Mass selective detector (Waldbronn, Germany) equipped with an achiral capillary column HP-5 (30m, 0.25mm i. d., 0.25μm thickness) using He as the carrier gas. Full sphere single crystal data collection for product **21** where performed at 100 K on a Bruker Kappa Apex II DUO diffractometer equiped with a Cryostream 700 plus low temperature device, a microsource anode with Mo *Kα* (λ= 0.71073 Å).

General procedure for the synthesis of β,β-diborylacrylates[1]

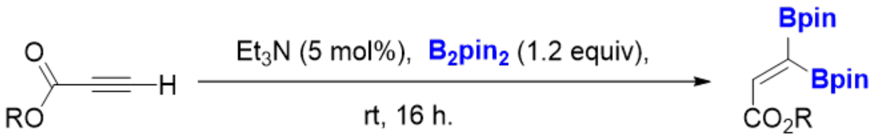

Bis(pinacolato)diboron (B_2pin_2, 3 mmol, 1 equiv) was placed in a vial containing a magnetic stirring bar. The vial was sealed with a Teflon®-coated silicon rubber septum and the vial was evacuated and filled with argon. The alkyne (3 mmol, 1 equiv) and Et_3N (0.15 mmol, 5 mol%) were sequentially added to the vial. After 16 h stirring at rt, the mixture was filtered through a short plug of silica gel, which was then washed with ethyl acetate. The solvent was removed under reduced pressure to afford the desired product in quantitative yield.

General procedure for site-selective Cu activation of β,β -diborylacrylates and nucleophilic allylic coupling: *representative example for synthesis of substrate 2*[2]

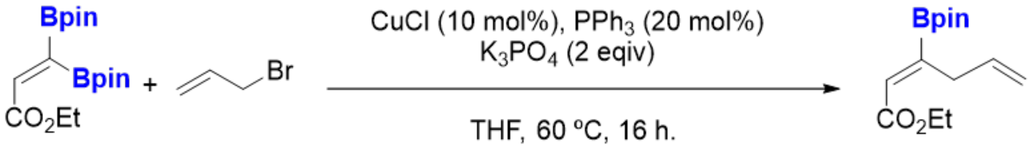

2

In a flamed Schlenk-tube equipped with a magnetic stir bar, β,β-diborylacrylate (1 mmol, 1 equiv, 352.04 mg), CuCl (0.1 mmol, 10 mmol %, 9.90 mg), PPh_3 (0.2 mmol, 20 mol%, 52.46 mg) and K_3PO_4 (2 mmol, 2 equiv, 424.53 mg) were added in THF (20 mL) under argon atmosphere. Next, allylbromide (1.5 mmol, 1.5 equiv, 0.130 mL) was introduced into the reaction mixture. After being stirred at 60 °C in an oil bath for 16 h, the reaction was concentrated under vacuum and the NMR yield was calculated by comparison to an internal standard (naphthalene). The crude residue was purified by silica gel flash chromatography to afford the desired product **2** (44% isolated yield, 118 mg).

Substrates **2a**, **2b**, **2c** and **2d** were also synthesized using the same methodology.[2]

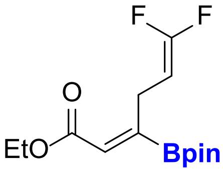

2a

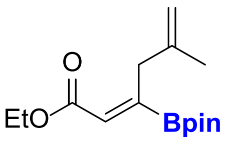

2b

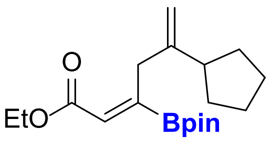

2c

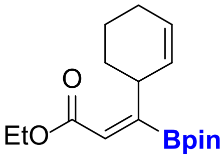

2d

General procedure for Cu-catalyzed borylcupration – protonation

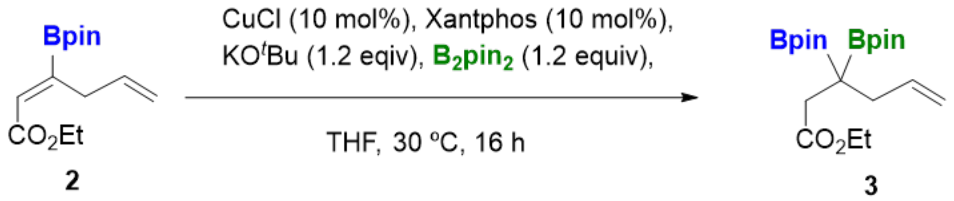

In a flamed Schlenk-tube equipped with a magnetic stir bar CuCl (1.98 mg, 10 mol%, 0.02 mmol), diboron reagent (60.9 mg, 1.2 equiv, 0.24 mmol) and Xantphos (277,6 mg, 10 mol%, 0.02 mmol) were placed. The vial was evacuated and backfilled with nitrogen and THF (1 mL) was added. Next, KO^{t}Bu (26.9 mg, 1.2 equiv, 0.24 mmol) in THF (1 mL) was poured in the vial through the rubber septum. Then, the borylated (*Z*)-skipped dienoate **2** (1 equiv, 0.2 mmol) in THF (1 mL) was added dropwise at 30 °C. After the reaction was completed, the reaction mixture was filtered over Celite. The solvents were evaporated at the rotatory evaporator and the crude was purified by silica gel chromatography to obtain the desired product **3** in 83% isolated yield (46 mg).

General procedure for Cu-catalyzed borylcupration – electrophilic trapping with alkyl or allyl halides

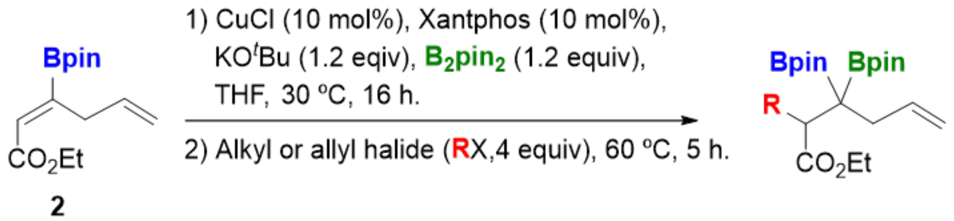

In a flamed Schlenk-tube equipped with a magnetic stir bar CuCl (1.98 mg, 10 mol%, 0.02 mmol), diboron reagent (60.9 mg, 1.2 equiv, 0.24 mmol) and Xantphos (277,6 mg, 10 mol%, 0.02 mmol) were placed. The vial was evacuated and backfilled with nitrogen and THF (1 mL) was added. Next, KO^{t}Bu (26.9 mg, 1.2 equiv, 0.24 mmol) in THF (1 mL) was poured in the vial through the rubber septum. Then, the borylated (*Z*)-skipped dienoate **2** (1 equiv, 0.2 mmol) in THF (1 mL) was added dropwise. After being stirred at 30 °C for 16 h, the corresponding alkyl or allyl halide (4 equiv) was added into the reaction at 60 ºC for 5 h. After the reaction was completed, the reaction mixture was filtered over Celite. The solvents were evaporated at the rotatory evaporator and the crude was purified by silica gel chromatography to obtain the desired product.

General procedure for Cu-catalyzed borylcupration – electrophilic trapping with isocyanates

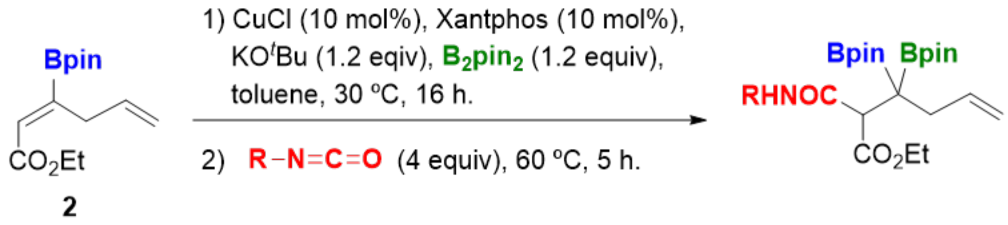

In a flamed Schlenk-tube equipped with a magnetic stir bar CuCl (1.98 mg, 10 mol%, 0.02 mmol), diboron reagent (60.9 mg, 1.2 equiv, 0.24 mmol) and Xantphos (277,6 mg, 10 mol%, 0.02 mmol) were placed. The vial was evacuated and backfilled with nitrogen and toluene (1 mL) was added. Next, KO^{t}Bu (26.9 mg, 1.2 equiv, 0.24 mmol) in toluene (1 mL) was poured in the vial through the rubber septum. Then, the borylated (Z) skipped dienoate (1 equiv, 0.2 mmol) in toluene (1 mL) was added dropwise. After being stirred at 30 °C for 16 h, the corresponding isocyanate (RNCO,4 equiv) was added into the reaction at 60 ºC for 5 h. After the reaction was completed, the reaction mixture was filtered over Celite. The solvents were evaporated at the rotatory evaporator and the crude was purified by silica gel chromatography to obtain the desired product.

Synthesis of compound 21

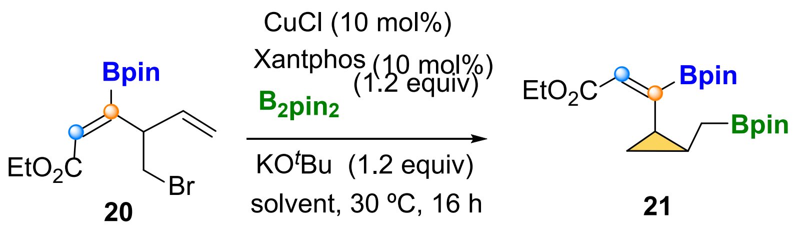

In a flamed Schlenk-tube equipped with a magnetic stir bar CuCl (1.98 mg, 10 mol%, 0.02 mmol), diboron reagent (60.9 mg, 1.2 equiv, 0.24 mmol) and Xantphos (277,6 mg, 10 mol%, 0.02 mmol) were placed. The vial was evacuated and backfilled with nitrogen and THF (1 mL) was added. Next, KO^{t}Bu (26.9 mg, 1.2 equiv, 0.24 mmol) in THF (1 mL) was poured in the vial through the rubber septum. Then, the borylated (*Z*) skipped dienoate (1 equiv, 0.2 mmol) in THF (1 mL) was added dropwise at 60 °C. After the reaction was completed, the reaction mixture was filtered over Celite. The solvents were evaporated at the rotatory evaporator and the crude was purified by silica gel chromatography to obtain the desired product **21** in 41% isolated yield (40 mg).

Synthesis of compound 24

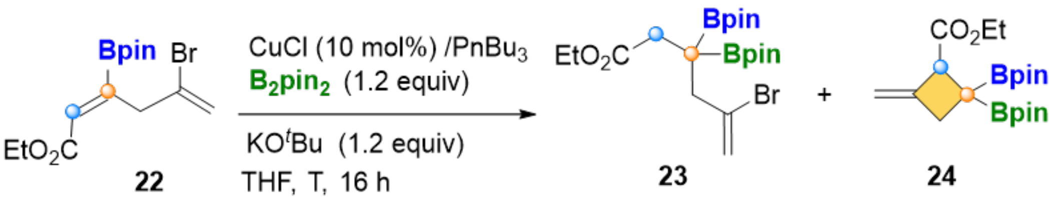

In a flamed Schlenk-tube equipped with a magnetic stir bar CuCl (1.98 mg, 10 mol%, 0.02 mmol), diboron reagent (60.9 mg, 1.2 equiv, 0.24 mmol) and $PnBu_3$ (10 μL, 20 mol%, 0.04 mmol) were placed. The vial was evacuated and backfilled with nitrogen and THF (1 mL) was added. Next, KO^{t}Bu (26.9 mg, 1.2 equiv, 0.24 mmol) in THF (1 mL) was poured in the vial through the rubber septum. Then, the borylated (*Z*) skipped dienoate (1 equiv, 0.2 mmol) in THF (1 mL) was added dropwise at 60 °C. After the reaction was completed, the reaction mixture was filtered over Celite. The solvents were evaporated at the rotatory evaporator and the crude was purified by silica gel chromatography to obtain the desired product **24** in 42% isolated yield (55 mg).

Synthesis of compound 29

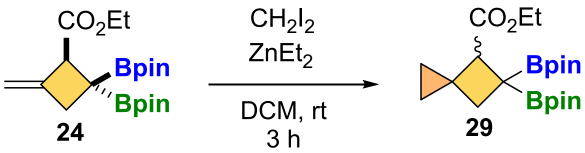

We followed the general procedure for Simmons–Smith cyclopropanation reactions.[3] To a solution of product **24** (78 mg, 0.2 mmol, 1.0 equiv) in anhydrous DCM (2.0 mL, 0.1 M) was added $ZnEt_2$ (1.0 M in hexane, 0.4 mL, 0.4 mmol, 2.0 equiv) at 0 °C. After stirring for 10 min, CH_2I_2 (161 mg, 0.6 mmol, 3.0 equiv) was added. The mixture was stirred at room temperature and a white precipitate was gradually generated. After 3 hours, the mixture was quenched with saturated aqueous NH_4Cl (8 mL) and extracted with EtOAc (3 x 10 mL). The combined organic layers were dried with Na_2SO_4, filtered through Celite, and concentrated in vacuo. The residue was purified by flash column chromatography on silica gel to give the corresponding product **29** in 55% isolated yield (39 mg).

Synthesis of compound 30

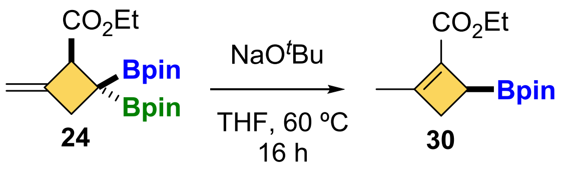

A Schlenk-tube equipped with a magnetic stir bar was charged with NaO^{t}Bu (28.83 mg, 3 equiv, 0.3 mmol), product **24** (39.2 mg, 1 equiv, 0.1 mmol,) and THF (0.5 mL). The Schlenk-tube was closed with a Teflon cap and the reaction was stirred for 16 h at 60 °C (oil bath). After the reaction was complete, the reaction mixture was filtered over Celite. The solvents were evaporated at the rotatory evaporator and the crude was purified by silica gel chromatography to obtain the desired product **30** in 57% isolated yield (12 mg).

General procedure for homologation reaction[4]

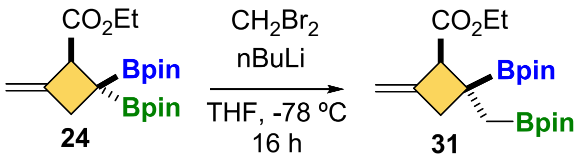

A Schlenk-tube equipped with a magnetic stir bar was evacuated and backfilled with N_2 three times. Then, was charged with product **24** (78.42 mg, 0.2 mmol, 1.00 equiv.) in THF (2.00 mL) and dibromomethane (50 μL, 0.64 mmol, 3.2 equiv.) were added sequentially via syringe and the mixture was cooled to -78 ºC in a dry ice/acetone bath. *n*-butyllithium (0.2 mL, 2.5 M in hexanes, 0.5 mmol, 2.5 equiv.) was added dropwise via syringe over 2 minutes. The reaction was stirred at 78 °C for 1 h and then placed in the freezer at -20 ºC for 24 h without stirring. The reaction was warmed to room temperature and quenched with H_2O (10 mL). The layers were separated, and the aqueous layer was extracted with ethyl acetate (3x10 mL), the combined organic layers were dried over $MgSO_4$, filtered over Celite. The solvents were evaporated at the rotatory evaporator and the crude was purified by silica gel chromatography to obtain the desired product **31** in 40% isolated yield (32 mg).

Oxidation of alkylidenecyclobutane 31

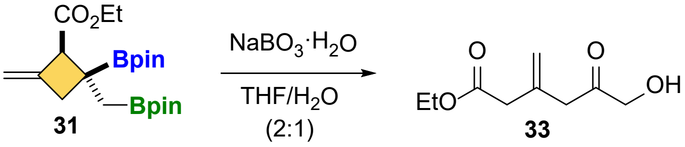

In an opened-air flask, charged with a magnetic stir bar, was added the alkylidenecyclobutane **31** (40.61 mg, 0.1 mmol, 1 equiv), $NaBO_3·H_2O$ (0.3 mmol, 3 equiv), THF (2 mL) and distilled water (1 mL). The reaction was closed with a septum with a needle to avoid over pressures and was stirred for 16 h at room temperature. After this period of time, the mixture was extracted with Et_2O (3 x 15 mL), the organic layer was dried with anhydrous magnesium sulphate, filtered and the solvents were evaporated. The crude residue was purified by silica gel chromatography to obtain product **33** in 37% isolated yield (3 mg).

Characterization data for borylated (*Z*)-skipped dienoates

Ethyl(*Z*)-3-(4,4,5,5-tetramethyl-1,3,2-dioxaborolan-2-yl)hexa-2,5-dienoate (2)

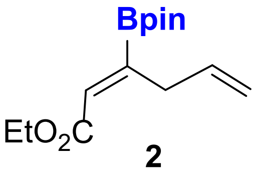

The product was purified by flash chromatography using as eluent a mixture of petroleum ether/ethyl acetate (100:1). The product was isolated as a pail yellowish oil (47 mg, 0.177 mmol, 52%).

^{1}H NMR (400 MHz, $CDCl_3$) δ= 6.44 (s, 1H), 5.88 (ddt, *J* = 16.7, 10.1, 6.5 Hz, 1H), 5.05 (dd, *J* = 17.2, 1.8 Hz, 1H), 4.96 (dd, *J* = 10.1, 1.5 Hz, 1H), 4.17 (q, *J* = 7.1 Hz, 2H), 3.44 (dd, *J* = 6.5, 1.4 Hz, 2H), 1.27 (t, *J* = 7.2, 3H), 1.25 (s, 12H).

^{13}C NMR (100 MHz, $CDCl_3$) δ= 166.0, 135.9, 130.4, 115.6, 84.2, 60.0, 34.1, 24.8, 14.3.

^{11}B NMR (129 MHz, $CDCl_3$) δ= 30.33.

HRMS-(ESI+) for $C_{14}H_{24}BO_4$ [M+H]$^+$: calculated 267.1765; found: 267.1755.

Ethyl (*Z*)-4-(bromomethyl)-3-(4,4,5,5-tetramethyl-1,3,2-dioxaborolan-2-yl)hexa-2,5-dienoate (20)

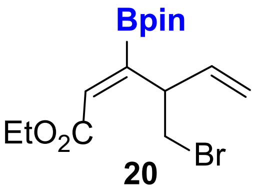

20

The product was purified by flash chromatography using as eluent a mixture of petroleum ether/ethyl acetate (100:1). The product was isolated as a pail yellowish oil (31 mg, 0.086 mmol, 28%).

^{1}H NMR (400 MHz, CDCl$_3$) δ= 6.53 (s, 1H), 5.92 (ddd, *J* = 17.2, 10.2, 7.9 Hz, 1H), 5.19 (dd, *J* = 17.2, 1.4 Hz, 1H), 5.10 (dd, *J* = 10.2, 1.2 Hz, 1H), 4.70 (bs, 1H), 4.19 (q, *J* = 7.1 Hz, 2H), 3.72 (t, *J* = 9.3 Hz, 1H), 3.52 (dd, *J* = 9.5, 7.2 Hz, 1H), 1.30 (t, *J* = 7.1 Hz, 3H), 1.27 (s, 12H).

^{13}C NMR (100 MHz, CDCl$_3$) δ= 165.7, 137.6, 132.3, 117.6, 84.3, 60.3, 46.5, 35.3, 24.8, 14.3.

^{11}B NMR (129 MHz, CDCl$_3$) δ= 29.86.

HRMS-(ESI+) for $C_{15}H_{25}BBrO_4$ [M+H]$^+$: calculated 359.1024; found: 359.1040.

Ethyl (*Z*)-5-bromo-3-(4,4,5,5-tetramethyl-1,3,2-dioxaborolan-2-yl)hexa-2,5-dienoate (22)

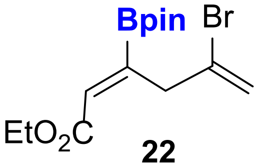

22

The product was purified by flash chromatography using as eluent a mixture of petroleum ether/ethyl acetate (100:1). The product was isolated as a pail yellowish oil (20 mg, 0.108 mmol, 36%).

^{1}H NMR (400 MHz, CDCl$_3$) δ= 6.57 (s, 1H), 5.56 (d, *J* = 1.5 Hz, 1H), 5.40 (d, *J* = 1.8Hz, 1H), 4.18 (q, *J* = 7.1 Hz, 2H), 3.89 (bs, 2H), 1.29 (t, *J* = 7.1 Hz, 3H), 1.26 (s, 12H).

^{13}C NMR (100 MHz, CDCl$_3$) δ= 165.6, 132.6, 131.2, 117.3, 84.5, 60.3, 40.5, 24.8, 14.3.

^{11}B NMR (129 MHz, CDCl$_3$) δ= 30.33.

HRMS-(ESI+) for $C_{14}H_{23}BBrO_4$ [M+H]$^+$: calculated 345.0867; found: 345.0867.

Ethyl (*Z*)-5-iodo-3-(4,4,5,5-tetramethyl-1,3,2-dioxaborolan-2-yl)hexa-2,5-dienoate (26)

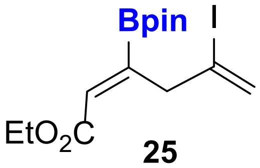

25

The product was purified by flash chromatography using as eluent a mixture of petroleum ether/ethyl acetate (100:1). The product was isolated as a pail yellowish oil (92 mg, 0.235 mmol, 18%).

^{1}H NMR (400 MHz, CDCl$_3$) δ= 6.60 (t, *J* = 1.2 Hz, 1H), 6.03 (q, *J* = 1.6 Hz, 1H), 5.73 (q, *J* = 1.2 Hz, 1H), 4.18 (q, *J* = 7.1 Hz, 2H), 3.93 (q, *J* = 1.3 Hz, 2H), 1.29 (t, *J* = 7.1 Hz, 3H), 1.27 (s, 12H).

^{13}C NMR (100 MHz, CDCl$_3$) δ= 165.7, 132.5, 126.3, 107.3, 84.5, 60.4, 44.5, 24.8, 14.4.

^{11}B NMR (129 MHz, CDCl$_3$) δ= 29.30.

HRMS-(ESI+) for $C_{14}H_{23}BIO_4$ [M+H]$^+$: calculated 393.0731; found: 393.0735.

Ethyl (*Z*)-5-chloro-3-(4,4,5,5-tetramethyl-1,3,2-dioxaborolan-2-yl)hexa-2,5-dienoate (26)

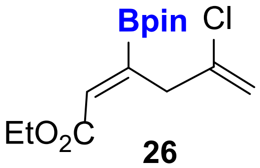

26

The product was purified by flash chromatography using as eluent a mixture of petroleum ether/ethyl acetate (100:1). The product was isolated as a pail yellowish oil (42 mg, 0.140 mmol, 5%).

^{1}H NMR (400 MHz, CDCl$_3$) δ= 6.56 (d, *J* = 1.1 Hz, 1H), 5.15 (d, *J* = 1.0 Hz, 1H), 5.12 (d, *J* = 1.3 Hz, 1H), 4.18 (q, *J* = 7.1 Hz, 2H), 3.78 (d, *J* = 1.2 Hz, 2H), 1.29 (t, *J* = 7.2 Hz, 3H), 1.26 (s, 12H).

^{13}C NMR (100 MHz, CDCl$_3$) δ= 165.5, 140.4, 132.6, 112.6, 84.4, 60.2, 38.3, 24.7, 14.2.

^{11}B NMR (129 MHz, CDCl$_3$) δ= 30.27.

HRMS-(ESI+) for $C_{14}H_{23}BClO_4$ [M+H]$^+$: calculated 301.1390; found: 301.1394.

Characterization data for products of Cu-catalyzed borylcupration and electrophilic trapping

Ethyl 3,3-bis(4,4,5,5-tetramethyl-1,3,2-dioxaborolan-2-yl)hex-5-enoate (3)

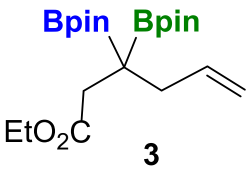

3

The product was purified by flash chromatography using as eluent a mixture of petroleum ether/ethyl acetate (100:1.5). The product was isolated as a yellowish oil (46 mg, 0.117 mmol, 83%).

^{1}H NMR (400 MHz, $CDCl_3$) δ= 5.71 (ddt, *J* = 17.5, 10.2, 7.4 Hz, 1H), 4.99 – 4.90 (m, 2H), 4.08 (q, *J* = 7.1 Hz, 2H), 2.61 (s, 2H), 2.41 (dt, *J* = 7.5, 1.2 Hz, 2H), 1.23 (s, 12H), 1.21 (s, 12H), 1.20 (t, *J* = 7.2 Hz, 3H).

^{13}C NMR (100 MHz, $CDCl_3$) δ= 171.6, 134.8, 113.7, 80.7, 57.5, 32.2, 32.2, 22.1, 22.1, 11.8.

^{11}B NMR (129 MHz, $CDCl_3$) δ= 33.40.

HRMS-(ESI+) for $C_{20}H_{37}B_2O_6$ [M+H]$^+$: calculated 395.2771; found: 395.2776

Ethyl 3-(5,5-dimethyl-1,3,2-dioxaborinan-2-yl)-3-(4,4,5,5-tetramethyl-1,3,2-dioxaborolan-2-yl)hex-5-enoate (3-Bneo)

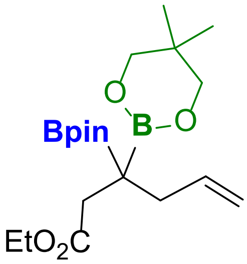

3-Bneo

The product was purified by flash chromatography using as eluent a mixture of petroleum ether/ethyl acetate (100:1.5). The product was isolated as a yellowish oil (28 mg, 0.074 mmol, 40%).

^{1}H NMR (400 MHz, $CDCl_3$) δ= 5.72 (ddt, *J* = 17.5, 10.1, 7.4 Hz, 1H), 4.99 – 4.89 (m, 2H), 4.09 (dq, *J* = 6.9, 3.7 Hz, 2H), 3.58 (s, 4H), 2.67 (d, *J* = 14.2, 1H), 2.51 (d, *J* = 14.2, 1H), 2.41 (dd, *J* = 14.1, 6.9 Hz, 1H), 2.28 (dd, *J* = 14.1, 7.3 Hz, 1H), 1.24 – 1.19 (m, 15H), 0.95 (s, 6H).

^{13}C NMR (100 MHz, $CDCl_3$) δ= 175.1, 138.1, 116.1, 83.3, 72.2, 60.1, 35.3, 35.1, 31.8, 24.9, 24.7, 22.1, 14.5.

^{11}B NMR (129 MHz, $CDCl_3$) δ= 34.49, 30.54.

HRMS-(ESI+) for $C_{19}H_{35}B_2O_6$ [M+H]$^+$: calculated 381.2614; found: 381.2618.

Ethyl 3-(4,4,5,5-tetramethyl-1,3,2-dioxaborolan-2-yl)-3-((3aS,7aR)-3a,5,5 trimethyl-hexahydro-4,6-methanobenzo[d][1,3,2]dioxaborol-2-yl)hex-5-enoate (3-Bpai)

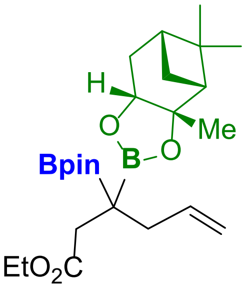

3-Bpai

The product was purified by flash chromatography using as eluent a mixture of petroleum ether/ethyl acetate (100:1.5). The product was isolated as a yellowish oil (50 mg, 0.112 mmol, 42%).

^{1}H NMR (400 MHz, CDCl$_3$) δ= 5.72 (ddt, J = 17.5, 10.2, 7.4 Hz, 1H), 4.99 – 4.90 (m, 2H), 4.28 (dd, J = 8.9, 2.0 Hz, 1H), 4.10 (dq, J = 9.2, 7.1 Hz, 2H), 2.62 (d, J = 2.5 Hz, 2H), 2.50 – 2.36 (m, 2H), 2.30 (tdd, J = 11.0, 5.3, 3.0 Hz, 1H), 2.14 (dtd, J = 12.7, 6.1, 2.2 Hz, 1H), 2.05 – 2.00 (m, 1H), 1.90 – 1.78 (m, 2H), 1.32 (d, J = 6.9 Hz, 3H), 1.27 – 1.17 (m, 19H), 0.82 (s, 3H).

^{13}C NMR (100 MHz, CDCl$_3$) δ= 174.3, 137.5, 116.4, 85.8, 83.5, 78.1, 60.2, 51.4, 39.6, 38.3, 35.7, 35.2, 35.1, 28.7, 27.2, 26.5, 24.9, 24.8, 24.2, 14.5.

^{11}B NMR (129 MHz, CDCl$_3$) δ= 32.49.

HRMS-(ESI+) for $C_{24}H_{41}B_2O_6$ [M+H]$^+$: calculated 447.3084; found: 447.3083.

Ethyl 6,6-difluoro-3,3-bis(4,4,5,5-tetramethyl-1,3,2-dioxaborolan-2-yl)hex-5-enoate (4)

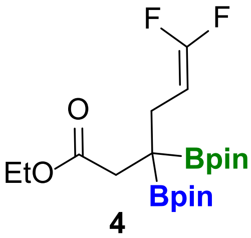

4

The product was purified by flash chromatography using as eluent a mixture of petroleum ether/ethyl acetate (100:1.5). The product was isolated as a yellowish oil (14 mg, 0.033 mmol, 70%).

^{1}H NMR (400 MHz, CDCl$_3$) δ= 4.15 (dd, J = 8.3, 3.0 Hz, 1H), 4.09 (q, J = 7.1 Hz, 2H), 2.60 (s, 2H), 2.33 (dd, J = 8.3, 1.8 Hz, 2H), 1.23 (s, 12H), 1.22 (s, 12H), 1.21 (t, J = 7.1 Hz, 3H).

^{13}C NMR (100 MHz, CDCl$_3$) δ= 174.0, 159.7 (dd, $^{1}J_{C-F}$ = 287.4, 284.6 Hz), 83.6, 77.1 (dd, $^{2}J_{C-F}$ = 30.4, 41.2 Hz), 60.4, 35.2, 31.1, 24.9, 24.8, 23.4 (d, $^{3}J_{C-F}$ = 4.3 Hz), 14.4.

^{11}B NMR (129 MHz, CDCl$_3$) δ= 33.07.

^{19}F NMR (377 MHz, CDCl$_3$) δ= -88.45 (d, $^{1}J_{F-F}$ = 47.2,1F), -91.32 (dd, $^{1}J_{F-F}$ = 47.3, $^{2}J_{F-H}$ 25.1 Hz, 1F).

HRMS-(ESI+) for $C_{20}H_{35}B_2F_2O_6$ [M+H]$^+$: calculated 431.2582; found: 431.2596.

Ethyl 5-methyl-3,3-bis(4,4,5,5-tetramethyl-1,3,2-dioxaborolan-2-yl)hex-5-enoate (5)

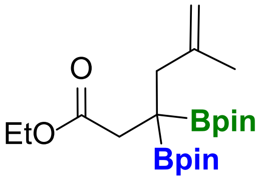

5

The product was purified by flash chromatography using as eluent a mixture of petroleum ether/ethyl acetate (100:1.5). The product was isolated as a yellowish oil (86 mg, 0.211 mmol, 76%).

^{1}H NMR (400 MHz, CDCl$_3$) δ= 4.67 (d, J = 2.9 Hz, 1H), 4.58 (d, J = 2.5 Hz, 1H), 4.06 (q, J = 7.1 Hz, 2H), 2.61 (d, J = 1.6 Hz, 2H), 2.49 (d, J = 1.6 Hz, 2H), 1.63 (s, 3H), 1.26 – 1.19 (m, 27H).

^{13}C NMR (100 MHz, CDCl$_3$) δ= 171.7, 142.1, 109.5, 80.8, 57.4, 35.1, 31.9, 22.2, 22.2, 20.5, 11.7.

^{11}B NMR (129 MHz, CDCl$_3$) δ= 33.85.

HRMS-(ESI+) for $C_{21}H_{39}B_2O_6$ [M+H]$^+$: calculated 409.2927; found: 409.2933.

Ethyl 5-cyclopentyl-3,3-bis(4,4,5,5-tetramethyl-1,3,2-dioxaborolan-2-yl)hex-5-enoate (6)

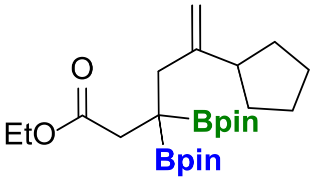

6

The product was purified by flash chromatography using as eluent a mixture of petroleum ether/ethyl acetate (100:1.5). The product was isolated as a yellowish oil (51 mg, 0.135 mmol, 82%).

^{1}H NMR (400 MHz, CDCl$_3$) δ= 4.75 (s, 1H), 4.58 (s, 1H), 4.06 (q, *J* = 7.1 Hz, 2H), 2.66 (s, 2H), 2.54 (s, 2H), 2.25 – 2.13 (m, 1H), 1.86 – 1.75 (m, 2H), 1.71 – 1.61 (m, 2H), 1.58 – 1.41 (m, 2H), 1.40 – 1.27 (m, 2H), 1.24 (s, 12H), 1.22 (s, 12H), 1.19 (t, *J* = 7.1 Hz, 3H).

^{13}C NMR (100 MHz, CDCl$_3$) δ= 174.6, 152.2, 107.7, 83.4, 60.1, 45.6, 36.1, 34.9, 31.9, 25.0, 24.9, 14.5.

^{11}B NMR (129 MHz, CDCl$_3$) δ= 34.19.

HRMS-(ESI+) for $C_{25}H_{45}B_2O_6$ [M+H]$^+$: calculated 463.3397; found: 463.3398.

Ethyl 3-(cyclohex-2-en-1-yl)-3,3-bis(4,4,5,5-tetramethyl-1,3,2-dioxaborolan-2-yl)propanate (7)

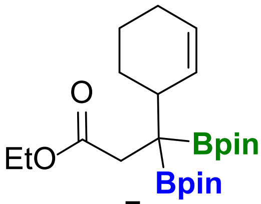

7

The product was purified by flash chromatography using as eluent a mixture of petroleum ether/ethyl acetate (100:1.5). The product was isolated as a yellowish oil (104 mg, 0.240 mmol, 74%).

^{1}H NMR (400 MHz, CDCl$_3$) δ= 5.71 – 5.57 (m, 2H), 4.05 (q, *J* = 7.1 Hz, 2H), 2.68 (dt, *J* = 10.8, 5.4, 1H), 2.59 (d, *J* = 1.5 Hz, 2H), 1.88 (dd, *J* = 8.8, 5.1, 2H), 1.82 – 1.67 (m, 2H), 1.64 (bs, 1H), 1.50 (bs, 1H), 1.24 (s, 12H), 1.23 (s, 12H), 1.20 (t, *J* = 7.1 Hz, 3H).

^{13}C NMR (100 MHz, CDCl$_3$) δ= 175.3, 132.3, 127.9, 83.3, 83.3, 60.1, 37.8, 33.8, 27.2, 25.2, 25.0, 24.9, 24.9, 23.2, 14.4.

^{11}B NMR (129 MHz, CDCl$_3$) δ= 34.67.

HRMS-(ESI+) for $C_{23}H_{41}B_2O_6$ [M+H]$^+$: calculated 435.3084; found: 435.3092.

Ethyl 3,3-bis(4,4,5,5-tetramethyl-1,3,2-dioxaborolan-2-yl)hex-5-enoate-2-*d* (8)

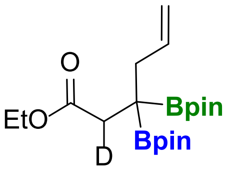

8

The product was purified by flash chromatography using as eluent a mixture of petroleum ether/ethyl acetate (100:1.5). The product was isolated as a yellowish oil (65 mg, 0.165 mmol, 76%).

^{1}H NMR (400 MHz, CDCl$_3$) δ= 5.78 – 5.64 (m, 1H), 5.00 – 4.90 (m, 2H), 4.09 (q, *J* = 7.1 Hz, 2H), 2.61 (d, *J* = 6.6 Hz, 1H), 2.41 (d, *J* = 7.4 Hz, 2H), 1.23 (s, 12H), 1.22 (s, 12H), 1.21 (t, *J* = 7.2 Hz, 3H).

^{13}C NMR (100 MHz, CDCl$_3$) δ= 174.3, 137.6, 116.4, 83.5, 60.2, 34.9, 34.9, 24.9, 24.9, 14.5.

^{11}B NMR (129 MHz, CDCl$_3$) δ= 34.40.

^{2}H NMR (61.28 MHz,CHCl$_3$) δ= 2.23.

Ethyl 2-methyl-3,3-bis(4,4,5,5-tetramethyl-1,3,2-dioxaborolan-2-yl)hex-5-enoate (9)

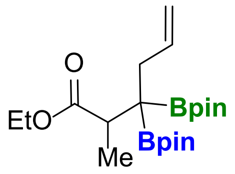

9

The product was purified by flash chromatography using as eluent a mixture of petroleum ether/ethyl acetate (100:1.5). The product was isolated as a yellowish oil (112 mg, 0.274 mmol, 78%).

^{1}H NMR (400 MHz, CDCl$_3$) δ= 5.88 (m, 1H), 4.95 (dd, *J* = 17.0, 2.8, 1H), 4.88 (dd, *J* = 10.0, 2.4, 1H), 4.16 – 4.02 (m, 2H), 2.85 (q, *J* = 7.3 Hz, 1H), 2.46 (dd, *J* = 14.6, 6.7, 1H), 2.26 (dd, *J* = 14.6, 8.1, Hz, 1H), 1.32 (d, *J* = 7.2 Hz, 3H), 1.25 – 1.19 (m, 27H).

^{13}C NMR (100 MHz, CDCl$_3$) δ= 177.5, 138.6, 115.5, 83.1, 83.1, 60.1, 42.0, 35.5, 25.0, 24.9, 24.9, 24.8, 16.0, 14.5.

^{11}B NMR (129 MHz, CDCl$_3$) δ= 34.26.

HRMS-(ESI+) for $C_{21}H_{39}B_2O_6$ [M+H]$^+$: calculated 409.2927; found: 409.2937.

Ethyl 2-benzyl-3,3-bis(4,4,5,5-tetramethyl-1,3,2-dioxaborolan-2-yl)hex-5-enoate (10)

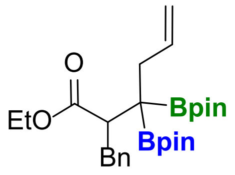

10

The product was purified by flash chromatography using as eluent a mixture of petroleum ether/ethyl acetate (100:1.5). The product was isolated as a yellowish oil (63 mg, 0.130 mmol, 69%).

^{1}H NMR (400 MHz, CDCl$_3$) δ= 7.25 – 7.19 (m, 4H), 7.18 – 7.11 (m, 1H), 5.89 (m, 1H), 5.01 (dd, *J* = 16.9, 2.6, 1H), 4.93 (dd, *J* = 10.1, 2.3, 1H), 3.91 (qd, *J* = 7.1, 3.1 Hz, 2H), 3.18 (dd, *J* = 13.4, 3.5 Hz, 1H), 3.11 (dd, *J* = 13.3, 11.2 Hz, 1H), 2.96 (dd, *J* = 11.2, 3.5 Hz, 1H), 2.53 (dd, *J* = 14.6, 7.1, 1H), 2.21 (dd, *J* = 14.6, 7.6, 1H), 1.31 (s, 6H), 1.29 (s, 6H), 1.26 (s, 6H), 1.25 (s, 6H), 0.98 (t, *J* = 7.1 Hz, 3H).

^{13}C NMR (100 MHz, CDCl$_3$) δ= 176.6, 141.8, 137.7, 129.2, 128.1, 125.8, 116.2, 83.4, 83.2, 59.7, 51.1, 38.2, 36.2, 25.1, 25.0, 25.0, 24.8, 14.2.

^{11}B NMR (129 MHz, CDCl$_3$) δ= 34.62.

HRMS-(ESI+) for $C_{27}H_{43}B_2O_6$ [M+H]$^+$: calculated 485.3240; found: 485.3243.

Ethyl 2-allyl-3,3-bis(4,4,5,5-tetramethyl-1,3,2-dioxaborolan-2-yl) hex-5-enoate (11)

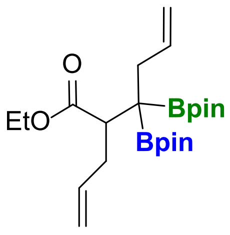

11

The product was purified by flash chromatography using as eluent a mixture of petroleum ether/ethyl acetate (100:1.5). The product was isolated as a yellowish oil (54 mg, 0.124 mmol, 70%).

^{1}H NMR (400 MHz, CDCl$_3$) δ= 5.84 (m, 2H), 4.99 (dd, *J* = 17.1, 2.2 Hz, 2H), 4.92 (dd, *J* = 10.3, 3.2 Hz, 2H), 4.09 (dq, *J* = 6.9, 3.6 Hz, 2H), 2.76 (dd, *J* = 10.0, 4.6 Hz, 1H), 2.64 – 2.54 (m, 2H), 2.48 (dd, *J* = 14.6, 6.8 Hz, 1H), 2.17 (dd, *J* = 14.5, 7.9 Hz, 1H), 1.25 (d, *J* = 3.0 Hz, 12H), 1.22 – 1.18 (m, 15H).

^{13}C NMR (100 MHz, CDCl$_3$) δ= 176.5, 137.9, 137.8, 116.1, 115.4, 83.3, 83.2, 59.9, 48.3, 36.3, 36.1, 25.0, 25.0, 24.8, 14.6.

^{11}B NMR (129 MHz, CDCl$_3$) δ= 34.01.

HRMS-(ESI+) for $C_{23}H_{41}B_2O_6$ [M+H]$^+$: calculated 435.3090; found: 435.3084.

Ethyl 2-(3,3-difluoroallyl)-3,3-bis(4,4,5,5-tetramethyl-1,3,2-dioxaborolan-2-yl)hex-5-enoate (12)

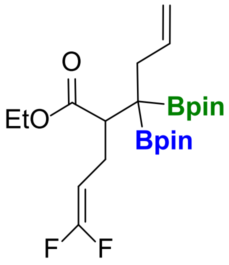

12

The product was purified by flash chromatography using as eluent a mixture of petroleum ether/ethyl acetate (100:1.5). The product was isolated as a yellowish oil (57 mg, 0.121 mmol, 61%).

^{1}H NMR (400 MHz, CDCl$_3$) δ= 5.91 – 5.76 (m, 1H), 5.02 – 4.88 (m, 2H), 4.27 – 4.15 (m, 1H), 4.11 (q, *J* = 7.2 Hz, 2H), 2.70 (dd, *J* = 10.9, 3.6 Hz, 1H), 2.62 – 2.53 (m, 1H), 2.52 – 2.42 (m, 2H), 2.18 (dd, *J* = 14.6, 7.9 Hz, 1H), 1.27 – 1.19 (m, 27H).

^{13}C NMR (100 MHz, CDCl$_3$) δ= 176.1, 156.3 (dd,$^{1}J_{C\text{-}F}$ = 287.8, 284.3 Hz), 137.7, 116.2, 83.4, 83.3, 77.5 (d, $^{2}J_{C\text{-}F}$ = 42.9 Hz), 60.2, 48.4, 35.9, 25.0, 24.9, 24.9, 24.8, 24.5 (d, $^{3}J_{C\text{-}F}$ = 4.5 Hz), 14.5.

^{11}B NMR (129 MHz, CDCl$_3$) δ= 34.79.

^{19}F NMR (377 MHz, CDCl$_3$) δ= -89.31 (d, $^{1}J_{F\text{-}F}$ = 46.4 Hz), -91.32 (dd, $^{1}J_{F\text{-}F}$ = 47.3, $^{2}J_{F\text{-}H}$ =25.1 Hz).

HRMS-(ESI+) for $C_{23}H_{39}B_2F_2O_6$ [M+H]$^+$: calculated 471.2895; found: 471.2900.

Ethyl (*E*)-2-(1,1-bis(4,4,5,5-tetramethyl-1,3,2-dioxaborolan-2-yl)but-3-en-1-yl)-6-bromohex-4-enoate (13)

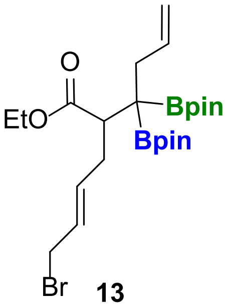

13

The product was purified by flash chromatography using as eluent a mixture of petroleum ether/ethyl acetate (100:1.5). The product was isolated as a yellowish oil (111 mg, 0.211 mmol, 52%).

^{1}H NMR (400 MHz, CDCl$_3$) δ= 5.91 – 5.74 (m, 2H), 5.73 – 5.62 (m, 1H), 5.05 – 4.89 (m, 2H), 4.09 (dq, *J* = 7.2, 3.6 Hz, 2H), 3.95 – 3.88 (m, 2H), 2.74 (dd, *J* = 9.9, 4.6 Hz, 1H), 2.68 – 2.57 (m, 2H), 2.48 (dd, *J* = 14.6, 6.9, 1H), 2.16 (dd, *J* = 14.6, 7.9, 1H), 1.25 (s, 6H), 1.24 (s, 6H), 1.23 – 1.20 (m, 15H).

^{13}C NMR (100 MHz, CDCl$_3$) δ= 176.3, 137.7, 135.6, 127.2, 116.3, 83.4, 83.2, 60.1, 48.1, 36.1, 34.5, 33.7, 25.0, 25.0, 24.8, 24.8, 14.6.

^{11}B NMR (129 MHz, CDCl$_3$) δ= 34.05.

HRMS-(ESI+) for $C_{24}H_{42}B_2BrO_6$ [M+H]$^+$: calculated 527.2345; found: 527.2354.

Ethyl 2-((4-methoxyphenyl)carbamoyl)-3,3-bis(4,4,5,5-tetramethyl-1,3,2-dioxaborolan-2-yl)hex-5-enoate (14)

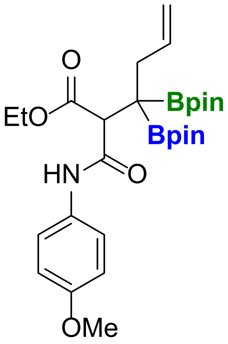

14

The product was purified by flash chromatography using as eluent a mixture of petroleum ether/ethyl acetate (100:3). The product was isolated as a colorless oil (67 mg, 0.123 mmol, 61%).

^{1}H NMR (400 MHz, CDCl$_3$) δ= 9.63 (s, 1H), 7.48 – 7.38 (m, 2H), 6.88 – 6.80 (m, 2H), 5.86 (m, 1H), 4.96 (dd, *J* = 17.0, 2.6, 1H), 4.89 (dd, *J* = 9.9, 2.2, 1H), 4.27 – 4.10 (m, 2H), 3.78 (s, 3H), 3.78 (s, 1H), 2.56 (dd, *J* = 14.4, 7.2, 1H), 2.43 (dd, *J* = 14.3, 7.7, 1H), 1.31 (s, 12H), 1.26 (t, *J* = 7.1 Hz, 3H), 1.17 (s, 6H), 1.15 (s, 6H).

^{13}C NMR (100 MHz, CDCl$_3$) δ= 172.2, 167.8, 156.2, 137.6, 132.0, 121.2, 116.7, 114.2, 83.8, 83.6, 61.7, 57.3, 55.6, 36.2, 25.1, 25.1, 25.0, 24.8, 14.3.

^{11}B NMR (129 MHz, CDCl$_3$) δ= 33.13, 22.50.

HRMS-(ESI+) for $C_{28}H_{44}B_2NO_8$ [M+H]$^+$: calculated 544.3248; found: 544.3255.

Ethyl 3,3-bis(4,4,5,5-tetramethyl-1,3,2-dioxaborolan-2-yl)-2-((4 -(trifluoromethyl) phenyl)carbamoyl)hex-5-enoate (15)

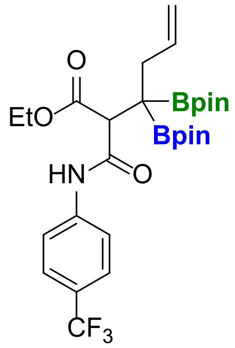

15

The product was purified by flash chromatography using as eluent a mixture of petroleum ether/ethyl acetate (100:3). The product was isolated as a colorless oil (59 mg, 0.101 mmol, 53%).

^{1}H NMR (400 MHz, CDCl$_3$) δ= 9.93 (s, 1H), 7.64 (d, *J* = 8.5 Hz, 2H), 7.55 (d, *J* = 8.6 Hz, 2H), 5.80 (m, 1H), 5.04 – 4.88 (m, 2H), 4.28 – 4.09 (m, 2H), 3.74 (s, 1H), 2.58 (dd, *J* = 14.5, 6.9, 1H), 2.40 (dd, *J* = 14.5, 7.9 Hz, 1H), 1.34 (s, 12H), 1.25 (t, *J* = 7.1 Hz, 3H), 1.14 (s, 6H), 1.10 (s, 6H).

^{13}C NMR (100 MHz, CDCl$_3$) δ= 171.7, 167.7, 142.1, 136.9, 126.2 (q, $^3J_{C\text{-}F}$ = 3.7 Hz), 125.5 (d, $^2J_{C\text{-}F}$ = 32.6 Hz), 123.0, 119.1, 117.3, 84.4, 83.9, 61.9, 58.2, 36.5, 25.0, 25.0, 25.0, 24.7, 14.3.

^{11}B NMR (129 MHz, CDCl$_3$) δ= 32.87, 22.31

^{19}F NMR (377 MHz, CDCl$_3$) δ= -62.02.

HRMS-(ESI+) for $C_{28}H_{41}B_2F_3NO_7$ [M+H]$^+$: calculated 582.3016; found: 582.3043

Ethyl 2-((2-bromophenyl)carbamoyl)-3,3-bis(4,4,5,5-tetramethyl-1,3,2-dioxaboro lan-2-yl)hex-5-enoate (16)

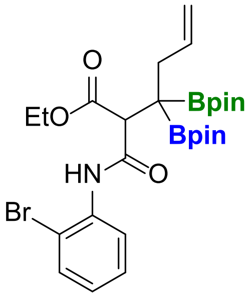

16

The product was purified by flash chromatography using as eluent a mixture of petroleum ether/ethyl acetate (100:3). The product was isolated as a colorless oil (66 mg, 0.111 mmol, 54%).

^{1}H NMR (400 MHz, CDCl$_3$) δ= 9.31 (s, 1H), 8.15 – 8.04 (m, 1H), 7.59 – 7.50 (m, 1H), 7.33 – 7.28 (m, 1H), 7.04 – 6.93 (m, 1H), 5.91 (m, 1H), 5.08 – 4.82 (m, 2H), 4.23 (q, *J* = 7.1, 2H), 3.95 (s, 1H), 2.67 – 2.51 (m, 2H), 1.30 (t, *J* = 7.1 Hz, 3H), 1.27 (s, 12H), 1.23 (d, *J* = 4.2 Hz, 12H).

^{13}C NMR (100 MHz, CDCl$_3$) δ= 171.5, 168.5, 138.0, 136.1, 132.5, 128.1, 125.6, 123.9, 116.5, 114.8, 83.7, 83.6, 61.8, 56.1, 35.3, 25.1, 25.0, 24.9, 24.8, 14.4.

^{11}B NMR (129 MHz, CDCl$_3$) δ= 33.02, 22.48

HRMS-(ESI+) for $C_{27}H_{41}B_2BrNO_7$ [M+H]$^+$: calculated 592.2247; found: 592.2271

Ethyl 2-(naphthalen-1-ylcarbamoyl)-3,3-bis(4,4,5,5-tetramethyl-1,3,2-dioxaborolan-2-yl)hex-5-enoate (17)

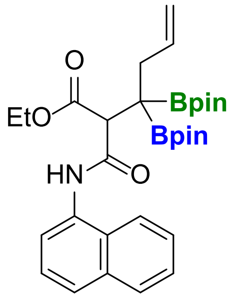

17

The product was purified by flash chromatography using as eluent a mixture of petroleum ether/ethyl acetate (100:3). The product was isolated as a colorless oil (41 mg, 0.072 mmol, 37%).

^{1}H NMR (400 MHz, CDCl$_3$) δ= 9.99 (s, 1H), 8.09 – 8.01 (m, 1H), 7.97 (dd, *J* = 7.5, 1.1 Hz, 1H), 7.89 – 7.81 (m, 1H), 7.67 (d, *J* = 8.2 Hz, 1H), 7.55 – 7.43 (m, 3H), 5.94 (m, 1H), 5.04 – 4.89 (m, 2H), 4.26 (q, *J* = 7.1 Hz, 2H), 3.99 (s, 1H), 2.64 (dd, *J* = 14.3, 7.2 Hz, 1H), 2.53 (dd, *J* = 14.3, 7.7 Hz, 1H), 1.34 – 1.25 (m, 15H), 1.20 (s, 12H).

^{13}C NMR (100 MHz, CDCl$_3$) δ= 172.5, 169.1, 137.8, 134.2, 133.0, 128.6, 127.38, 126.0, 125.9, 125.9, 125.3, 121.9, 120.6, 116.8, 83.9, 83.7, 61.8, 56.9, 36.3, 25.1, 25.0, 24.9, 24.8, 14.4.

^{11}B NMR (129 MHz, CDCl$_3$) δ= 33.26, 22.54

HRMS-(ESI+) for $C_{31}H_{44}B_2NO_7$ [M+H]$^+$: calculated 564.3298; found: 564.3297

Ethyl 2-(*tert*-butylcarbamoyl)-3,3-bis(4,4,5,5-tetramethyl-1,3,2-dioxaborolan-2-yl)hex-5-enoate (18)

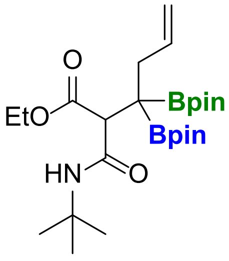

18

The product was purified by flash chromatography using as eluent a mixture of petroleum ether/ethyl acetate (100:3). The product was isolated as a colorless oil (59 mg, 0.109 mmol, 71%).

^{1}H NMR (400 MHz, CDCl$_3$) δ= 8.36 (s, 1H), 6.08 – 5.93 (m, 1H), 4.82 – 4.73 (m, 2H), 4.26 – 4.17 (m, 1H), 4.14 – 4.04 (m, 1H), 3.79 (s, 1H), 2.43 (dd, *J* = 7.6, 1.4 Hz, 2H), 1.36 (s, 9H), 1.30 (t, *J* = 7.2 Hz, 3H), 1.22 (s, 6H), 1.21 (s, 6H), 1.18 (s, 12H).

^{13}C NMR (100 MHz, CDCl$_3$) δ= 174.1, 173.1, 140.0, 115.3, 82.1, 81.5, 61.4, 53.6, 53.2, 35.3, 28.5, 25.6, 25.4, 25.0, 24.9, 14.2.

^{11}B NMR (129 MHz, CDCl$_3$) δ= 28.69, 23.48

HRMS-(ESI+) for $C_{25}H_{46}B_2NO_7$ [M+H]$^+$: calculated 494.3455; found: 494.3463.

Ethyl 2-(benzylcarbamoyl)-3,3-bis(4,4,5,5-tetramethyl-1,3,2-dioxaborolan-2-yl) hex-5-enoate (19)

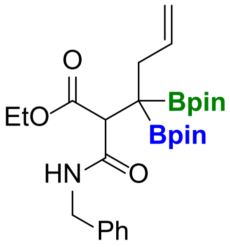

19

The product was purified by flash chromatography using as eluent a mixture of petroleum ether/ethyl acetate (100:3). The product was isolated as a colorless oil (37 mg, 0.070 mmol, 44%).

^{1}H NMR (400 MHz, CDCl$_3$) δ= 8.19 (s, 1H), 7.36 – 7.22 (m, 5H), 5.95 (m, 1H), 4.94 – 4.82 (m, 2H), 4.46 (q, *J* = 14.7, 5.4 Hz, 2H), 4.25 – 4.08 (m, 2H), 3.79 (s, 1H), 2.57 – 2.39 (m, 2H), 1.27 (t, *J* = 7.1 Hz, 3H), 1.21 (s, 6H), 1.19 (s, 6H), 1.18 (s, 6H), 1.16 (s, 6H).

^{13}C NMR (100 MHz, CDCl$_3$) δ= 172.4, 171.7, 138.7, 137.5, 128.8, 128.3, 127.7, 115.6, 83.0, 82.9, 61.5, 54.8, 45.0, 35.7, 25.2, 25.1, 24.9, 24.8, 14.3.

^{11}B NMR (129 MHz, CDCl$_3$) δ= 31.08, 22.39.

HRMS-(ESI+) for $C_{28}H_{44}B_2NO_7$ [M+H]$^+$: calculated 528.3298; found: 528.3301.

Ethyl 5-bromo-3,3-bis(4,4,5,5-tetramethyl-1,3,2-dioxaborolan-2-yl)hex-5-enoate (23)

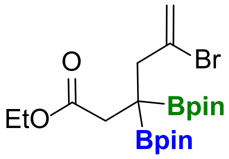

23

The product was purified by flash chromatography using as eluent a mixture of petroleum ether/ethyl acetate (100:1.5). The product was isolated as a white solid (59 mg, 0.125 mmol, 45%).

^{1}H NMR (400 MHz, CDCl$_3$) δ= 5.48 (s, 1H), 5.35 (s, 1H), 4.07 (q, J = 7.2 Hz, 2H), 2.98 (s, 2H), 2.73 (s, 2H), 1.25 (s, 12H), 1.23 (s, 12H), 1.21 (t, J = 7.2 Hz, 3H).

^{13}C NMR (100 MHz, CDCl$_3$) δ= 174.3, 134.5, 118.6, 83.7, 60.3, 41.7, 34.2, 25.0, 24.9, 24.9, 14.5

^{11}B NMR (129 MHz, CDCl$_3$) δ= 33.02.

HRMS-(ESI+) for $C_{20}H_{36}B_2BrO_6$ [M+H]$^+$: calculated 473.1876; found: 473.1888.

Ethyl 5-iodo-3,3-bis(4,4,5,5-tetramethyl-1,3,2-dioxaborolan-2-yl)hex-5-enoate (27)

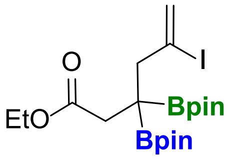

27

The product was purified by flash chromatography using as eluent a mixture of petroleum ether/ethyl acetate (100:1.5). The product was isolated as a yellowish oil (10 mg, 0.019 mmol, 8%).

^{1}H NMR (400 MHz, CDCl$_3$) δ= 5.97 (d, *J* = 1.3 Hz, 1H), 5.69 (d, *J* = 1.4 Hz, 1H), 4.07 (q, *J* = 7.1 Hz, 2H), 3.02 (d, *J* = 1.3 Hz, 2H), 2.76 (s, 2H), 1.26 (s, 12H), 1.24 (s, 12H), 1.21 (t, *J* = 7.1 Hz, 3H).

^{13}C NMR (100 MHz, CDCl$_3$) δ= 174.3, 127.3, 111.4, 83.8, 60.3, 45.3, 34.2, 25.1, 25.0, 14.5

^{11}B NMR (129 MHz, CDCl$_3$) δ= 33.01.

HRMS-(ESI+) for $C_{20}H_{36}B_2IO_6$ [M+H]$^+$: calculated 521.1737; found 521.1743.

Ethyl 5-chloro-3,3-bis(4,4,5,5-tetramethyl-1,3,2-dioxaborolan-2-yl)hex-5-enoate (28)

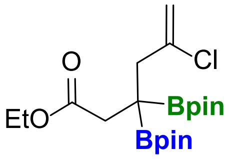

28

The product was purified by flash chromatography using as eluent a mixture of petroleum ether/ethyl acetate (100:1.5). The product was isolated as a yellowish oil (25 mg, 0.058 mmol, 49%).

^{1}H NMR (400 MHz, CDCl$_3$) δ= 5.09 (d, *J* = 0.9 Hz, 1H), 5.04 (d, *J* = 1.0 Hz, 1H), 4.07 (q, *J* = 7.1 Hz, 2H), 2.86 (d, *J* = 0.9 Hz, 2H), 2.70 (s, 2H), 1.24 (s, 12H), 1.22 (s, 12H), 1.20 (t, *J* = 7.1 Hz, 3H).

^{13}C NMR (100 MHz, CDCl$_3$) δ= 174.3, 142.7, 114.1, 83.7, 60.2, 39.7, 34.2, 25.0, 24.9, 14.5

^{11}B NMR (129 MHz, CDCl$_3$) δ= 34.56.

HRMS-(ESI+) for $C_{20}H_{36}B_2ClO_6$ [M+H]$^+$: calculated 429.2379; found 429.2381.

Characterization data for cyclic and oxidized products

Ethyl (*Z*)-3-(4,4,5,5-tetramethyl-1,3,2-dioxaborolan-2-yl)-3-(2-((4,4,5,5-tetramethyl-1,3,2-dioxaborolan-2-yl)methyl)cyclopropyl)acrylate (21)

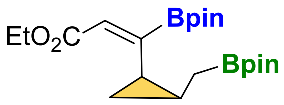

21

The product was purified by flash chromatography using as eluent a mixture of petroleum ether/ethyl acetate (100:1.5). The product was isolated as a yellowish oil (53 mg, 0.131 mmol, 54%).

^{1}H NMR (400 MHz, CDCl$_3$) δ= 6.19 (s, 1H), 4.09 (q, *J* = 7.1 Hz, 2H), 2.68 (m, 1H), 1.33 – 1.25 (m, 1H), 1.20 (t, *J* = 7.1 Hz, 3H), 1.16 (s, 24H), 1.11 (ddd, *J* = 8.8, 5.1, 3.9 Hz, 1H, H), 0.93 (dd, *J* = 15.6, 6.2 Hz, 1H), 0.74 (dd, *J* = 15.6, 7.3 Hz, 1H), 0.65 (ddd, *J* = 8.5, 5.7, 4.0 Hz, 1H).

^{13}C NMR (100 MHz, CDCl$_3$) δ= 166.8, 127.1, 83.9, 83.1, 59.7, 24.9, 24.9, 24.8, 24.7, 23.1, 18.3, 17.5, 14.5.

^{11}B NMR (129 MHz, CDCl$_3$) δ= 33.88, 29.98.

HRMS-(ESI+) for $C_{21}H_{37}B_2O_6$ [M+H]$^+$: calculated 407.2771; found: 407.2781.

Ethyl 4-methylene-2,2-bis(4,4,5,5-tetramethyl-1,3,2-dioxaborolan-2-yl)cyclobutane -1-carboxylate (24)

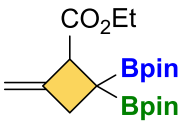

24

The product was purified by flash chromatography using as eluent a mixture of petroleum ether /ethyl acetate (100:3). The product was isolated as a colorless oil (44 mg, 0.112 mmol, 42%).

^{1}H NMR (400 MHz, CDCl$_3$) δ= 4.87 (d, J = 2.6 Hz, 1H), 4.76 (d, J = 2.4 Hz, 1H), 4.12 (q, J = 7.1 Hz, 2H), 3.96 (bs, 1H), 3.23 (dd, J = 15.3, 2.9 Hz, 1H), 2.74 (dd, J = 15.3, 2.3 Hz, 1H), 1.28 – 1.20 (m, 27H).

^{13}C NMR (100 MHz, CDCl$_3$) δ= 173.6, 146.2, 107.5, 83.7, 83.6, 60.9, 53.1, 34.9, 25.0, 24.9, 24.9, 24.7, 14.4.

^{11}B NMR (129 MHz, CDCl$_3$) δ= 34.20.

HRMS-(ESI+) for $C_{20}H_{35}B_2O_6$ [M+H]$^+$: calculated 393.2614; found: 393.2616

Methyl 4-methylene-2,2-bis(4,4,5,5-tetramethyl-1,3,2-dioxaborolan-2-yl)cyclobutane-1-carboxylate (24^{Me})

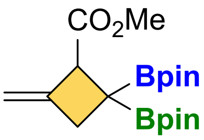

24^{Me}

The product was purified by flash chromatography using as eluent a mixture of petroleum ether /ethyl acetate (100:3). The product was isolated as a colorless oil (50 mg, 0.132 mmol, 66%).

^{1}H NMR (400 MHz, $CDCl_3$) δ= 4.86 (d, *J* = 2.6 Hz, 1H), 4.77 (d, *J* = 2.4 Hz, 1H), 3.97 (s, 1H), 3.67 (s, 3H), 3.23 (dd, *J* = 15.4, 2.9 Hz, 1H), 2.75 (dd, *J* = 15.4, 2.3 Hz, 1H), 1.26 (s, 6H), 1.24 (s, 6H), 1.23 (s, 6H), 1.22 (s, 6H).

^{13}C NMR (100 MHz, $CDCl_3$) δ= 174.1, 145.8, 107.7, 83.8, 83.6, 52.7, 52.1, 35.0, 25.0, 24.9, 24.8, 24.7, 24.7.

^{11}B NMR (129 MHz, $CDCl_3$) δ= 32.61.

Ethyl 5,5-bis(4,4,5,5-tetramethyl-1,3,2-dioxaborolan-2-yl)spiro[2.3]hexane-4-carboxylate (29)

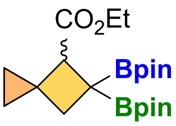

29

The product was purified by flash chromatography using as eluent a mixture of petroleum ether/ethyl acetate (100:3). The product was isolated as a colorless oil (39 mg, 0.096 mmol, 55%) as a mixture of two isomers (55:45).

^{1}H NMR (400 MHz, $CDCl_3$) δ= **29**: 4.17 – 4.02 (m, 2H), 3.12 (s, 1H), 2.16 (dd, *J* = 10.5, 1.3 Hz, 1H), 2.07 (dd, *J* = 10.5, 1.0 Hz, 1H), 1.30 – 1.17 (m, 27H), 0.72 (ddd, *J* = 9.6, 6.2, 3.8 Hz, 1H), 0.50 – 0.38 (m, 3H). **29'**: 4.17 – 4.02 (m, 2H), 3.16 (s, 1H), 2.88 (d, *J* = 10.1 Hz, 1H), 2.00 (dd, *J* = 10.3, 1.6 Hz, 1H), 1.30 – 1.17 (m, 27H), 0.64 (ddd, *J* = 10.4, 6.5, 4.4 Hz, 1H), 0.54 (ddd, *J* = 10.3, 6.2, 4.4 Hz, 1H), 0.50 – 0.38 (m, 1H), 0.29 (ddd, *J* = 9.7, 6.5, 5.2 Hz, 1H).

^{13}C NMR (100 MHz, $CDCl_3$) δ= 175.8, 173.8, 83.6, 83.4, 83.0, 60.4, 59.6, 51.7, 51.5, 39.8, 34.3, 25.1, 25.0, 24.9, 24.9, 24.9, 24.8, 24.7, 24.5, 23.3, 19.1, 14.7, 14.6, 14.5, 12.6, 10.0, 8.8.

^{11}B NMR (129 MHz, $CDCl_3$) δ= 35.18.

HRMS-(ESI+) for $C_{21}H_{37}B_2O_6$ $[M+H]^+$: calculated 407.2779; found: 407.2771

Ethyl 2-methyl-4-(4,4,5,5-tetramethyl-1,3,2-dioxaborolan-2-yl)cyclobut-1-ene-1-carboxylate (30)

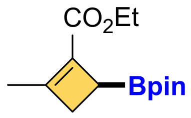

30

The product was purified by flash chromatography using as eluent a mixture of petroleum ether/ethyl acetate (100:3). The product was isolated as a colorless oil (12 mg, 0.145 mmol, 57%).

^{1}H NMR (400 MHz, CDCl$_3$) δ= 4.24 – 4.16 (m, 1H), 4.16 – 4.07 (m, 1H), 2.49 – 2.42 (m, 1H), 2.36 – 2.29 (m, 1H), 2.04 (s, 3H), 1.27 (t, *J* = 7.1 Hz, 3H), 1.24 (s, 6H), 1.23 (s, 6H).

^{13}C NMR (100 MHz, CDCl$_3$) δ= 163.1, 158.7, 131.8, 83.3, 59.6, 32.2, 24.8, 24.8, 16.8, 14.5.

^{11}B NMR (129 MHz, CDCl$_3$) δ= 33.59.

HRMS-(ESI+) for $C_{14}H_{24}BO_4$ [M+H]$^+$: calculated 267.1762; found: 267.1765

Ethyl 4-methylene-2-(4,4,5,5-tetramethyl-1,3,2-dioxaborolan-2-yl)-2-((4,4,5,5-tetramethyl-1,3,2-dioxaborolan-2-yl)methyl)cyclobutane-1-carboxylate (31)

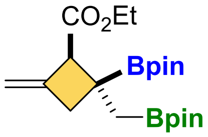

31

The product was purified by flash chromatography using as eluent a mixture of petroleum ether/ethyl acetate (100:1.5). The product was isolated as a yellowish oil (32,4 mg, 0.080 mmol, 40%).

^{1}H NMR (400 MHz, CDCl$_3$) δ= 4.99 (d, *J* = 2.6 Hz, 1H), 4.84 (d, *J* = 2.5 Hz, 1H), 4.22 – 4.03 (m, 2H), 3.47 (bs, 1H), 3.00 (dd, *J* = 15.3, 2.6 Hz, 1H), 2.27 (dd, *J* = 15.4, 2.6 Hz, 1H), 1.32 (d, *J* = 15.4 Hz, 1H), 1.26 – 1.16 (m, 27H), 1.07 (d, *J* = 15.4 Hz, 1H).

^{13}C NMR (100 MHz, CDCl$_3$) δ= 172.5, 144.3, 107.6, 83.5, 83.0, 60.4, 58.8, 40.0, 25.2, 25.2, 25.1, 24.7, 14.5.

^{11}B NMR (129 MHz, CDCl$_3$) δ= 34.21.

HRMS-(ESI+) for $C_{21}H_{37}B_2O_6$ [M+H]$^+$: calculated 407.2771; found 407.2788.

Ethyl 2-methyl-4-(4,4,5,5-tetramethyl-1,3,2-dioxaborolan-2-yl)-4-((4,4,5,5-tetramethyl-1,3,2-dioxaborolan-2-yl)methyl)cyclobut-1-ene-1-carboxylate (32)

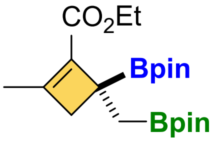

32

The product was purified by flash chromatography using as eluent a mixture of petroleum ether/ethyl acetate (100:1.5). The product was isolated as a yellowish oil (10 mg, 0.025 mmol, 20%).

^{1}H NMR (400 MHz, $CDCl_3$) δ= 4.13 (q, J = 7.1 Hz, 2H), 2.63 (d, J = 15.2 Hz, 1H), 2.03 (s, 3H), 1.98 (d, J = 15.2 Hz, 1H), 1.52 (d, J = 15.8 Hz, 1H), 1.25 (t, J = 7.1 Hz, 3H), 1.22 – 1.19 (m, 24H), 0.79 (d, J = 15.8 Hz, 1H).

^{13}C NMR (100 MHz, $CDCl_3$) δ= 163.1, 158.0, 138.0, 83.3, 82.8, 59.3, 41.3, 29.9, 25.2, 24.9, 24.8, 24.5, 16.7, 14.6.

^{11}B NMR (129 MHz, $CDCl_3$) δ= 34.10.

HRMS-(ESI+) for $C_{21}H_{37}B_2O_6$ $[M+H]^+$: calculated 407.2771; found: 407.2782.

Ethyl 6-hydroxy-3-methylene-5-oxohexanoate (33)

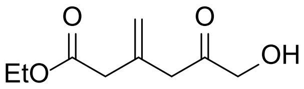

33

The product was purified by flash chromatography using as eluent a mixture of petroleum ether/ethyl acetate (100:1.5). The product was isolated as a yellowish oil (3mg, 0.013 mmol, 37%).

^{1}H NMR (400 MHz, $CDCl_3$) δ= 5.15 (d, J = 1.1 Hz, 1H), 5.08 (d, J = 1.2 Hz, 1H), 4.31 (s, 2H), 4.13 (q, J = 7.2 Hz, 2H), 3.31 (d, J = 1.0 Hz, 2H), 3.15 (d, J = 1.2 Hz, 2H), 1.26 (t, J = 7.1 Hz, 3H).

^{13}C NMR (100 MHz, $CDCl_3$) δ= 207.3, 171.1, 134.8, 119.6, 68.0, 61.1, 45.4, 41.7, 14.3.

HRMS-(ESI+) for $C_9H_{15}O_4$ $[M+H]^+$: calculated 187.0965; found 187.0966.

^{1}H, ^{13}C, ^{11}B, NMR spectra

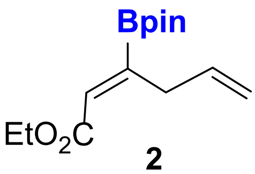

2

^{1}H NMR (400 MHz, $CDCl_3$)

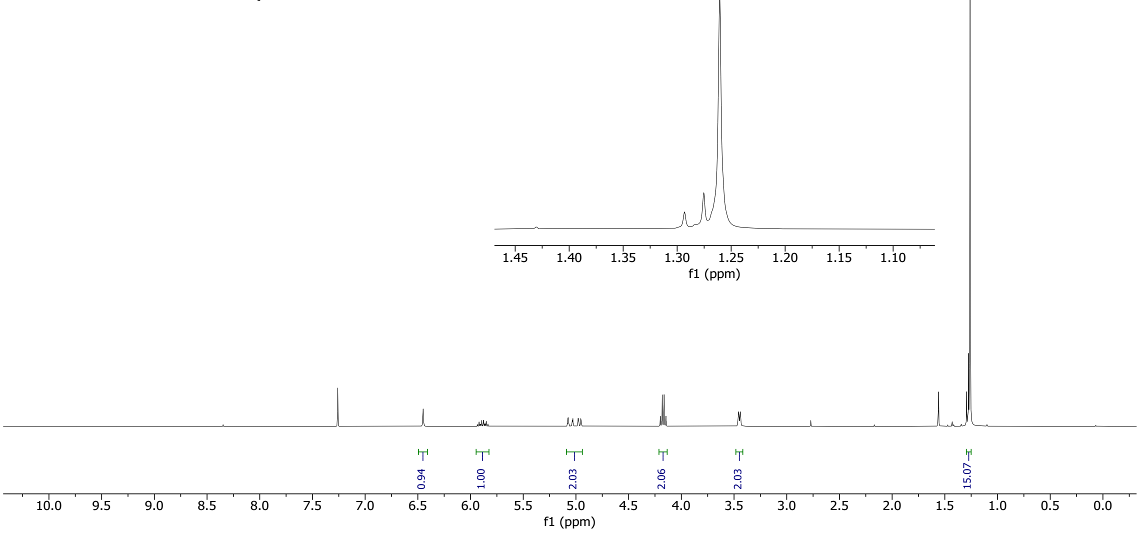

1-D NMR NOE EXPERIMENT

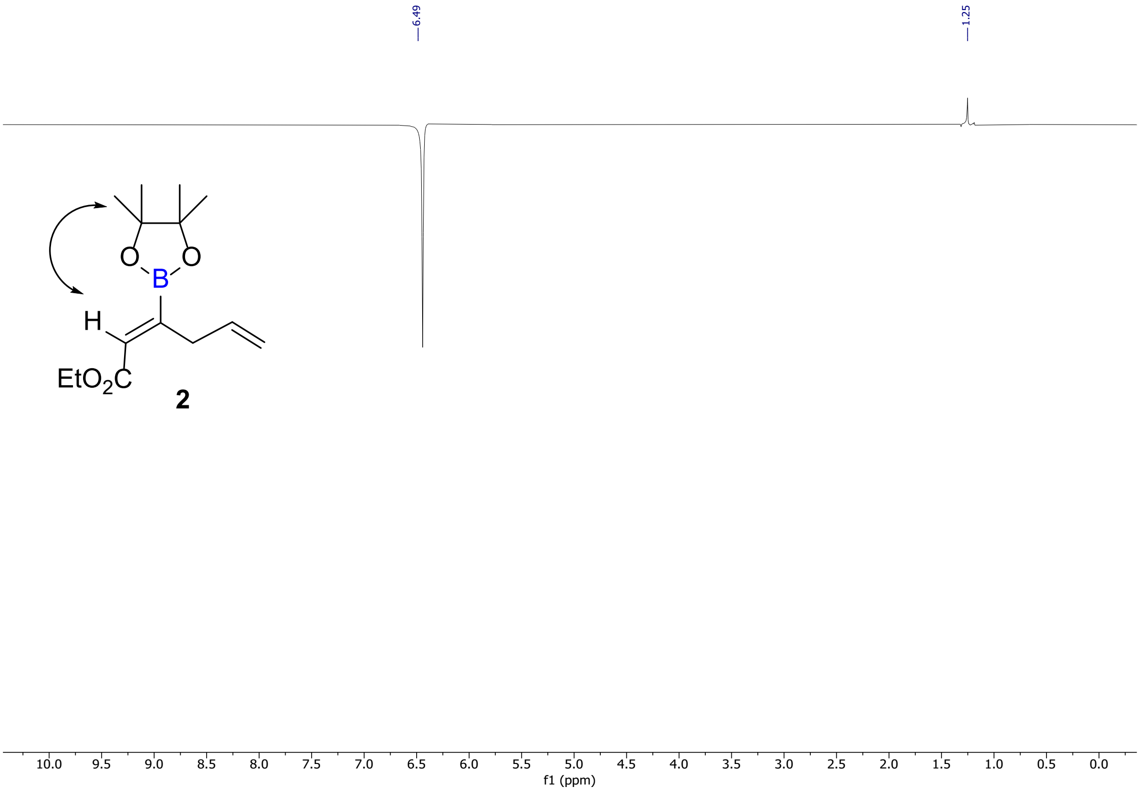

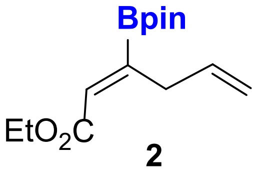

2

^{13}C NMR (100 MHz, CDCl$_3$)

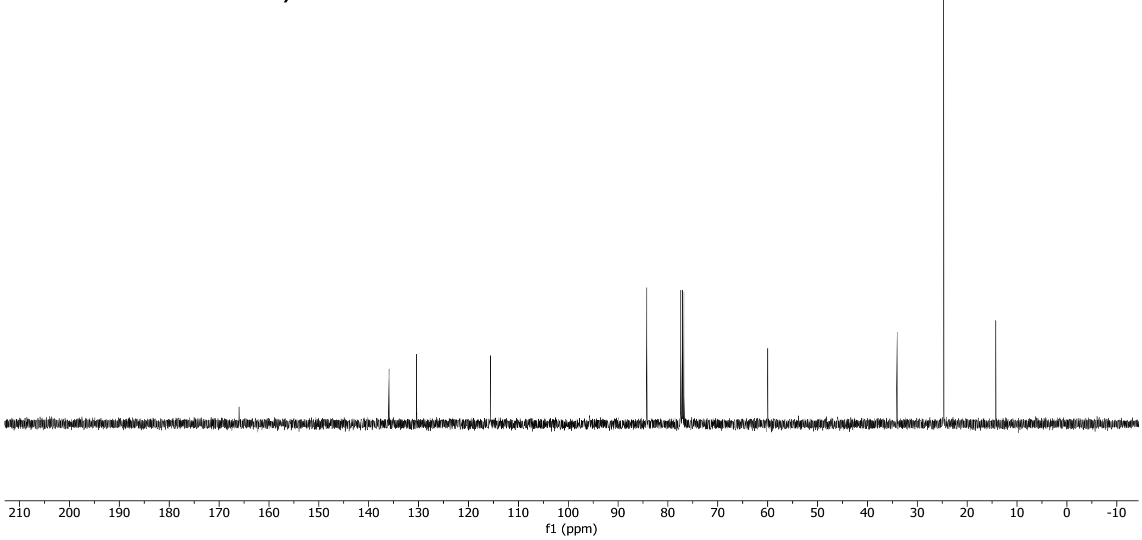

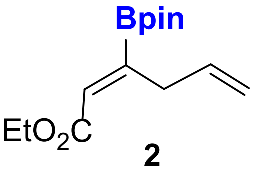

2

^{11}B NMR (129 MHz, CDCl$_3$)

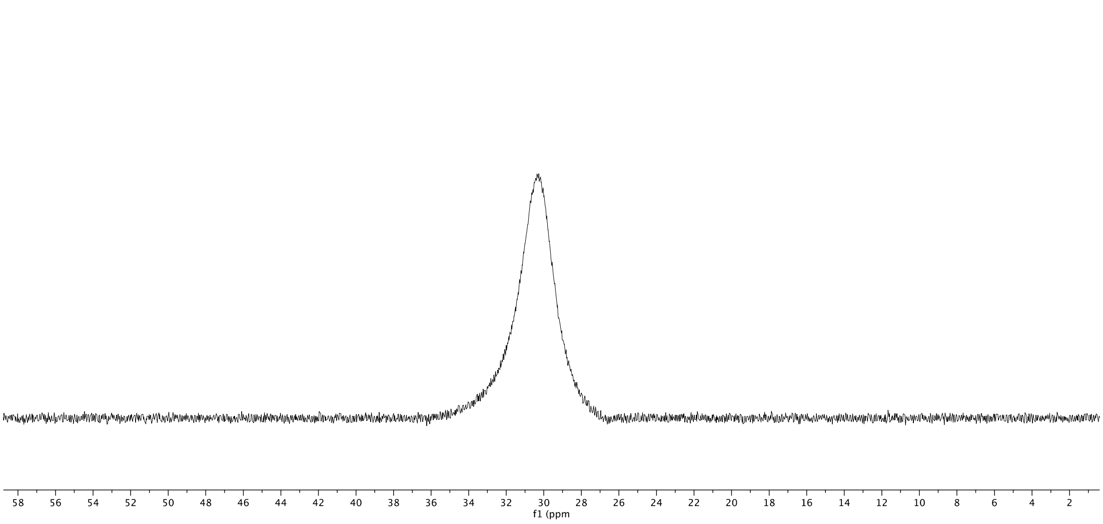

20

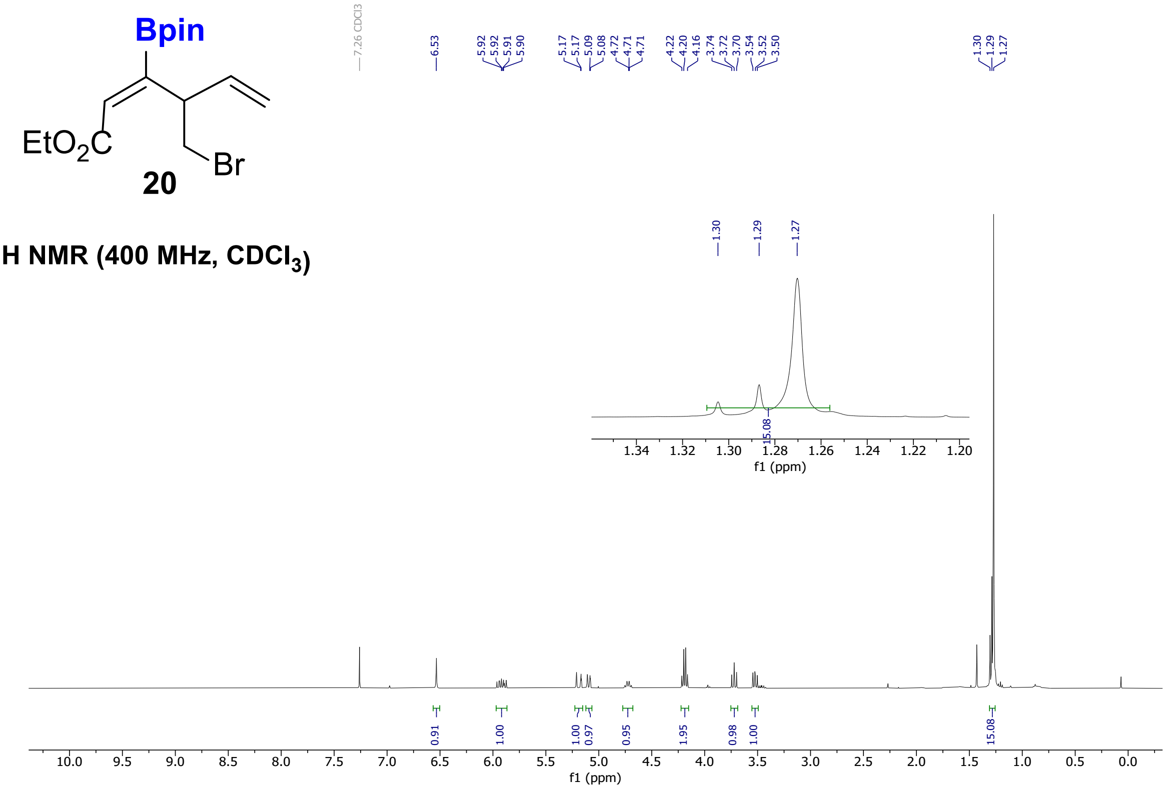

^{1}H NMR (400 MHz, CDCl$_3$)

20

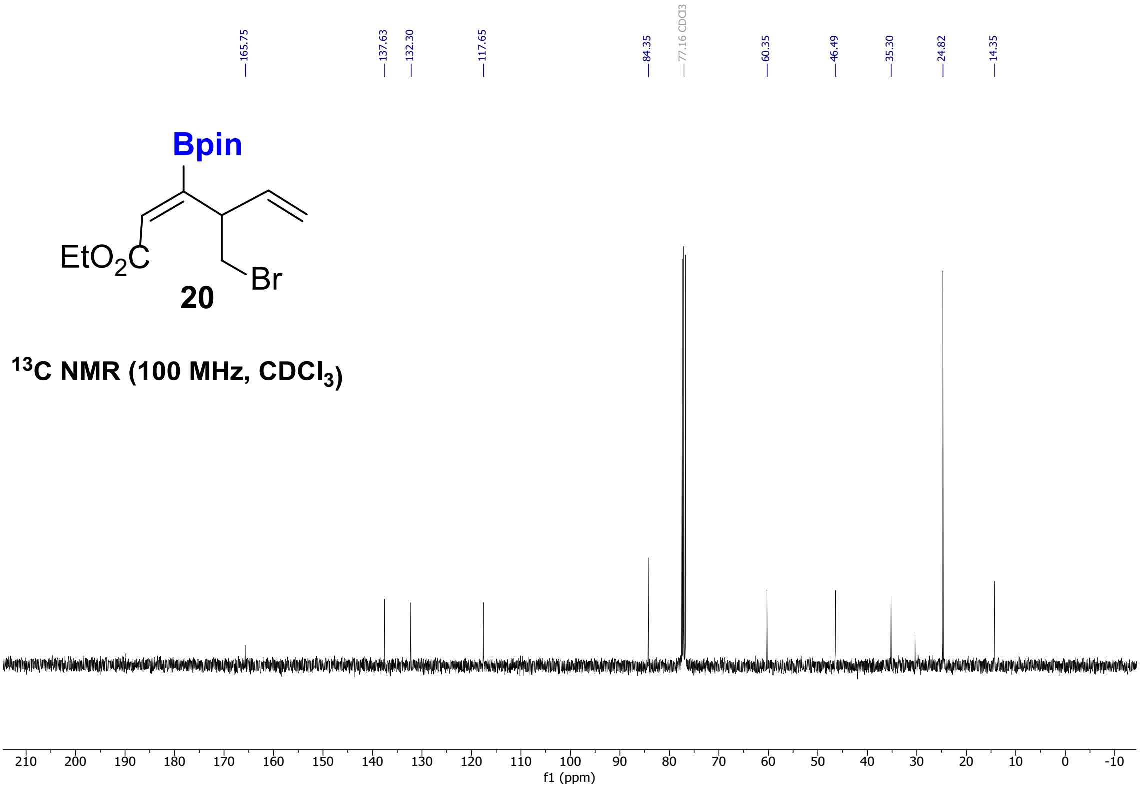

^{13}C NMR (100 MHz, CDCl$_3$)

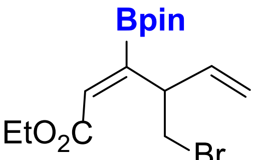

20

^{11}B NMR (129 MHz, CDCl$_3$)

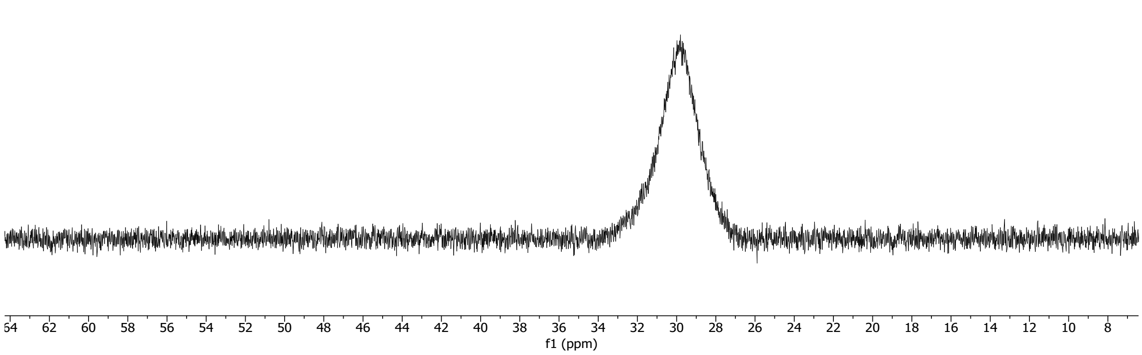

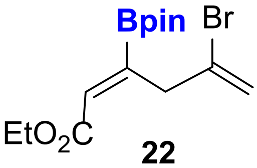

22

^{1}H NMR (400 MHz, CDCl$_3$)

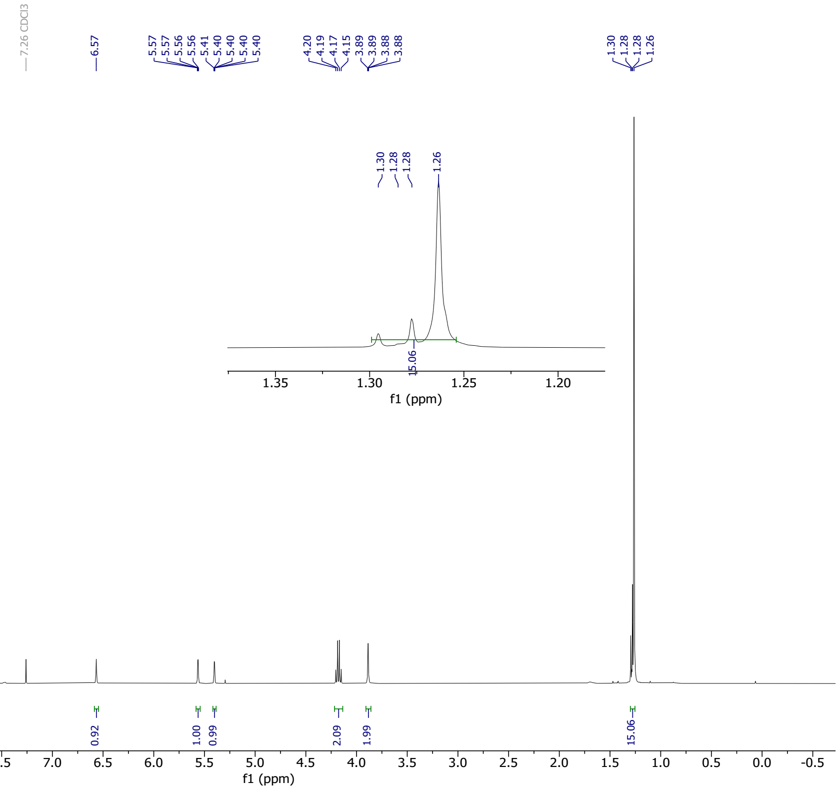

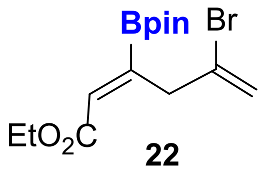

22

^{13}C NMR (100 MHz, $CDCl_3$)

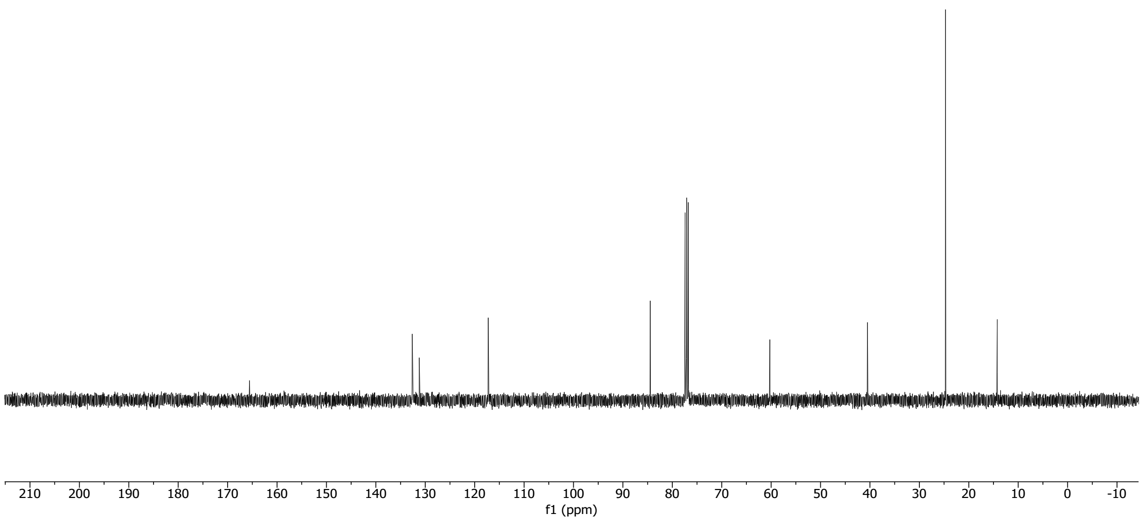

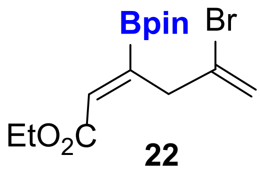

22

^{11}B NMR (129 MHz, $CDCl_3$)

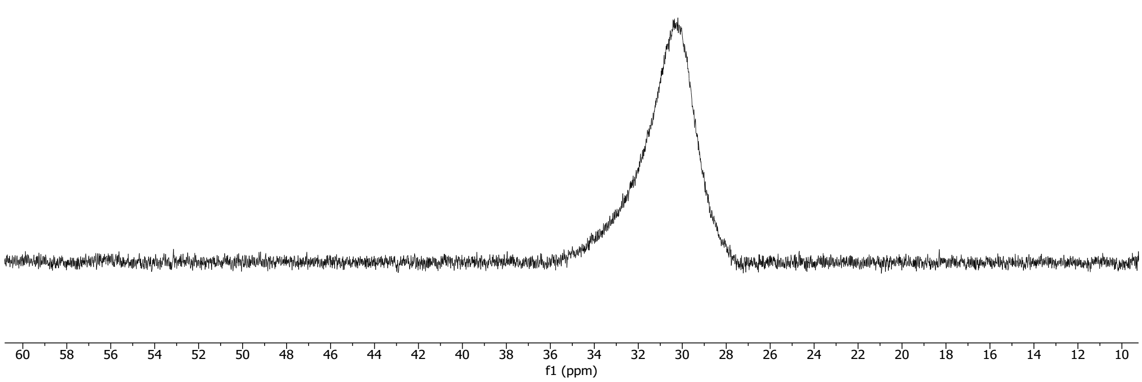

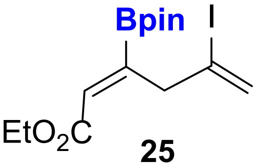

25

^{1}H NMR (400 MHz, CDCl$_3$)

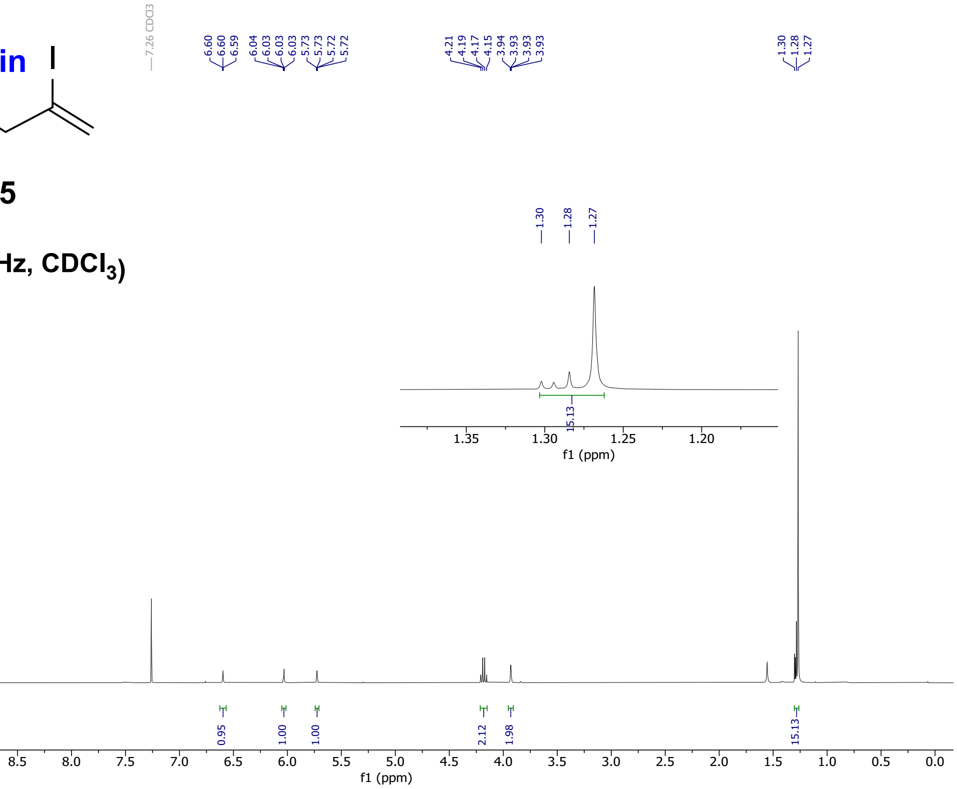

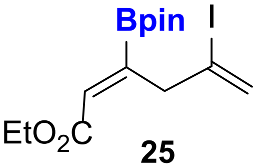

25

^{13}C NMR (100 MHz, CDCl$_3$)

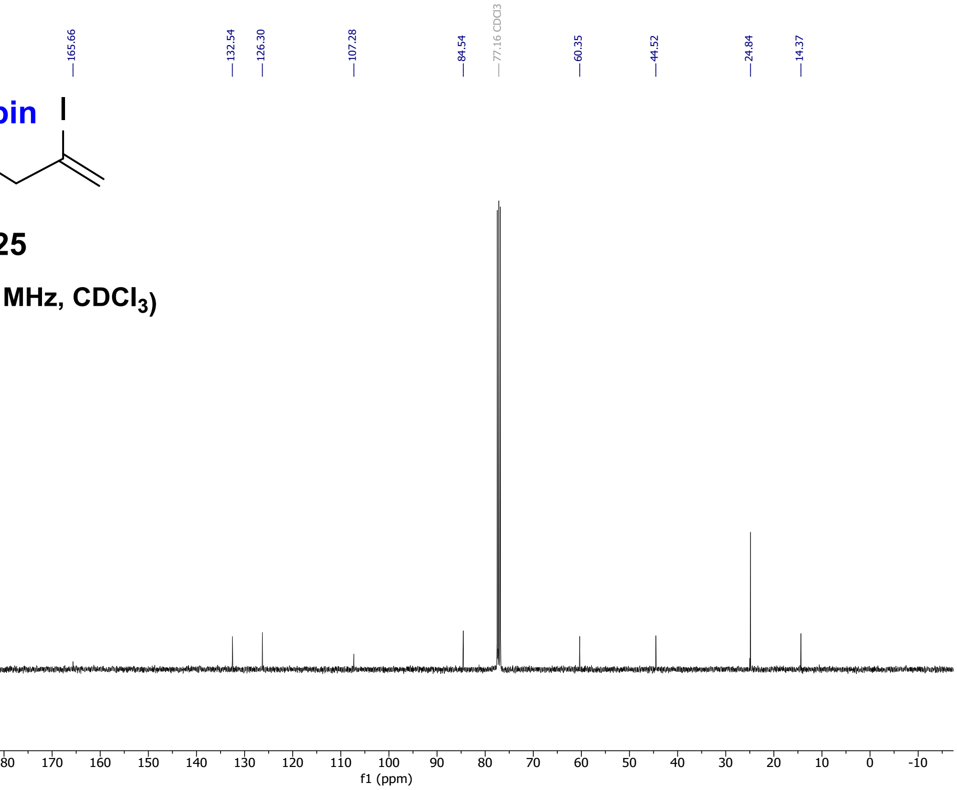

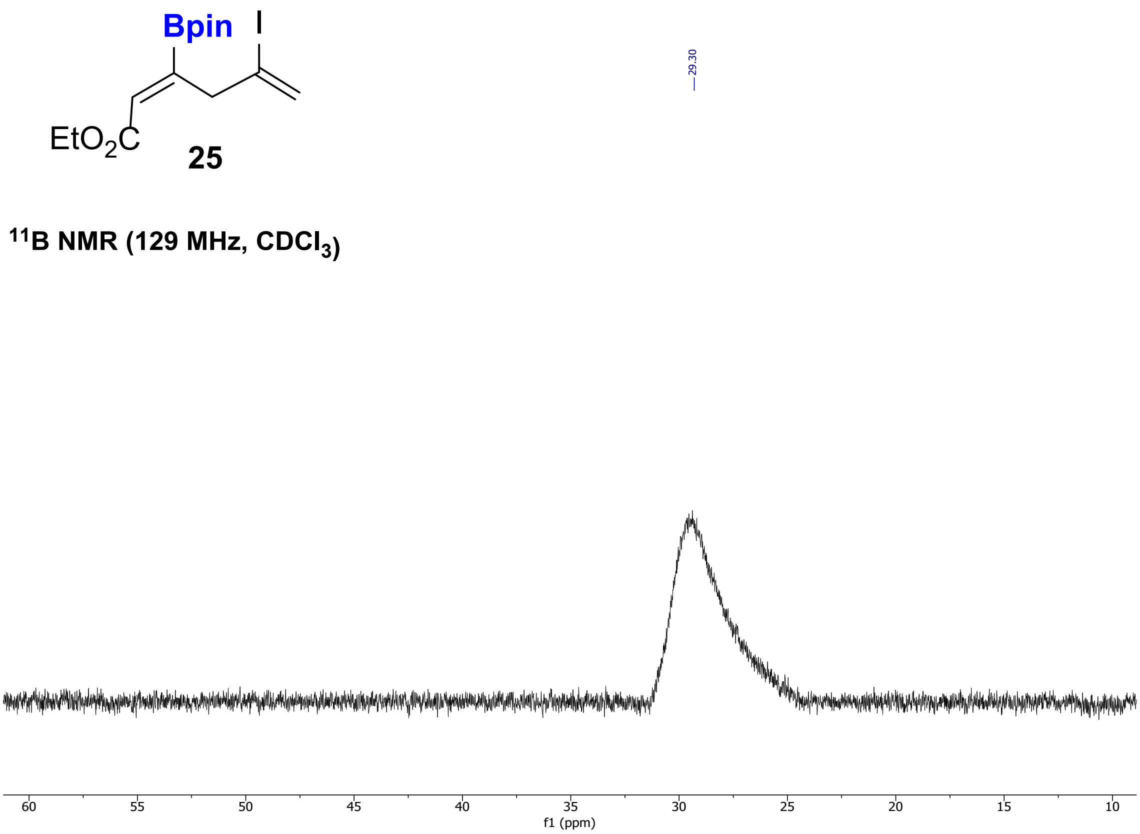

25

^{11}B NMR (129 MHz, CDCl$_3$)

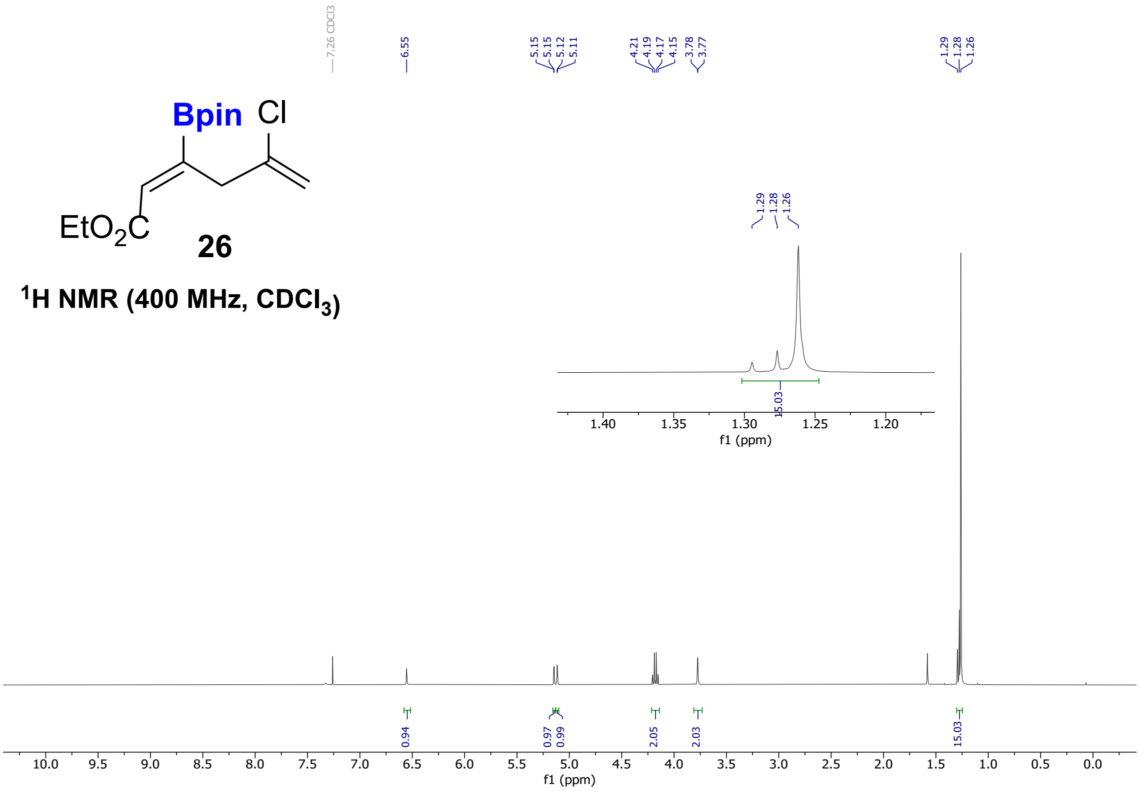

26

^{1}H NMR (400 MHz, CDCl$_3$)

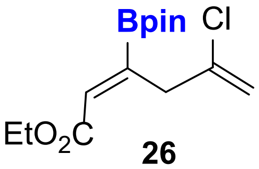

26

^{13}C NMR (100 MHz, $CDCl_3$)

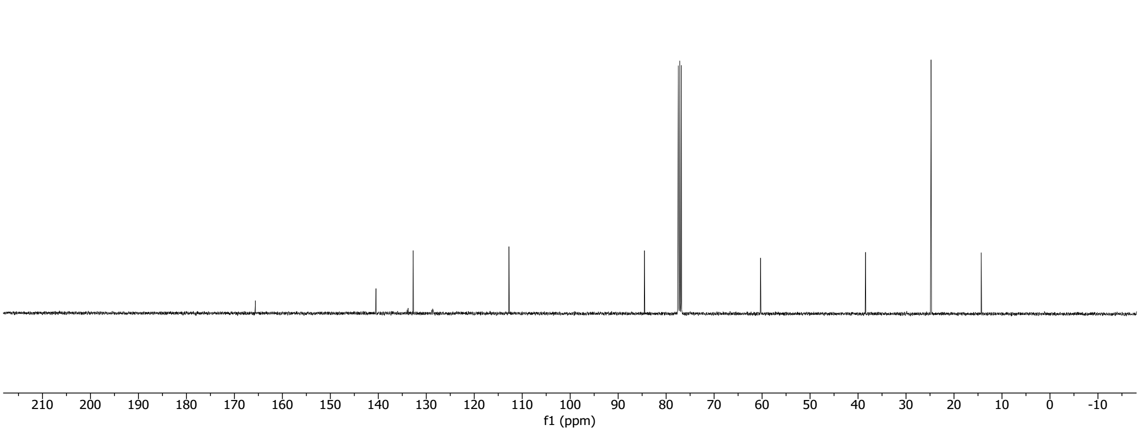

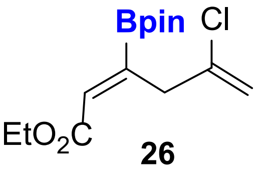

26

^{11}B NMR (129 MHz, $CDCl_3$)

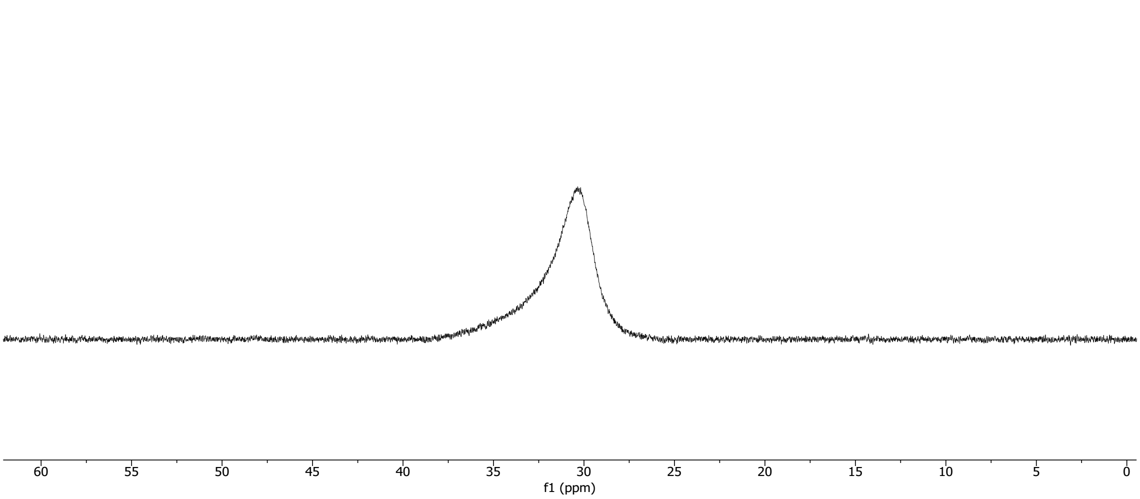

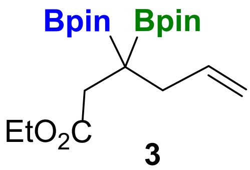

3

^{1}H NMR (400 MHz, $CDCl_3$)

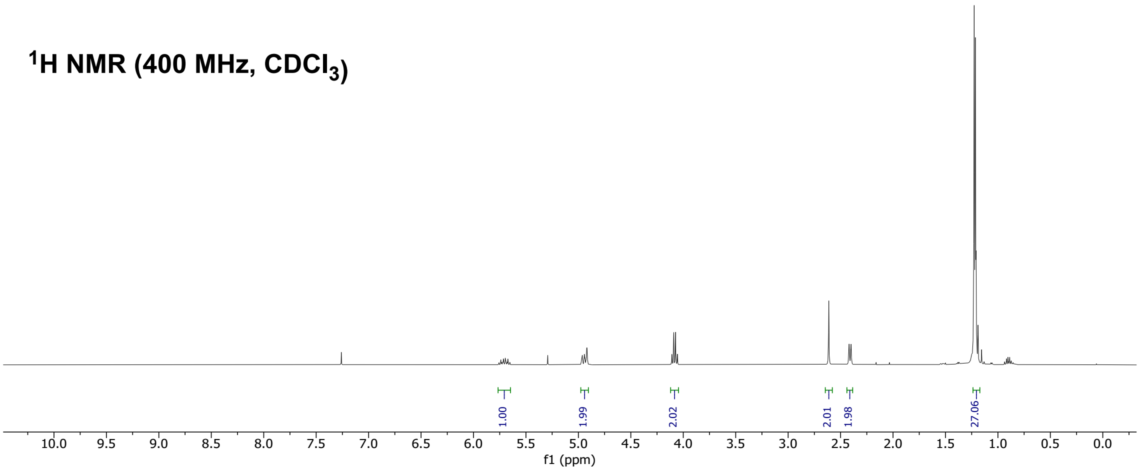

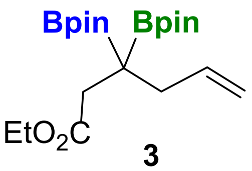

3

^{13}C NMR (100 MHz, $CDCl_3$)

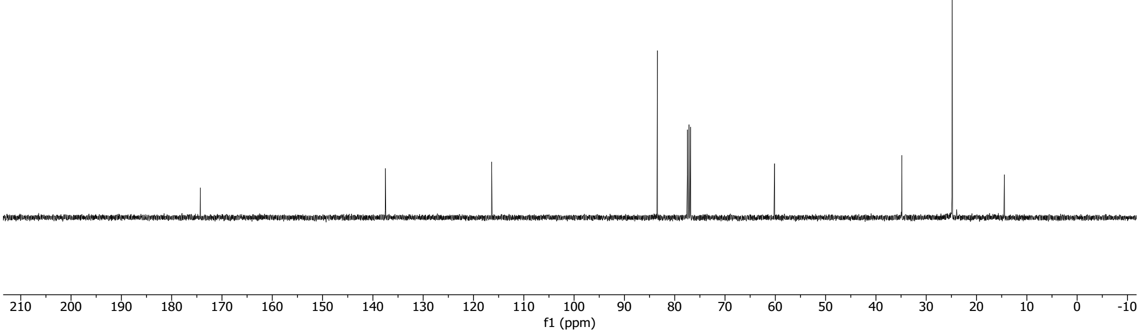

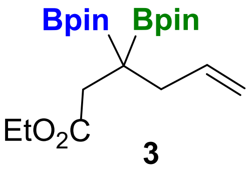

3

^{11}B NMR (129 MHz, $CDCl_3$)

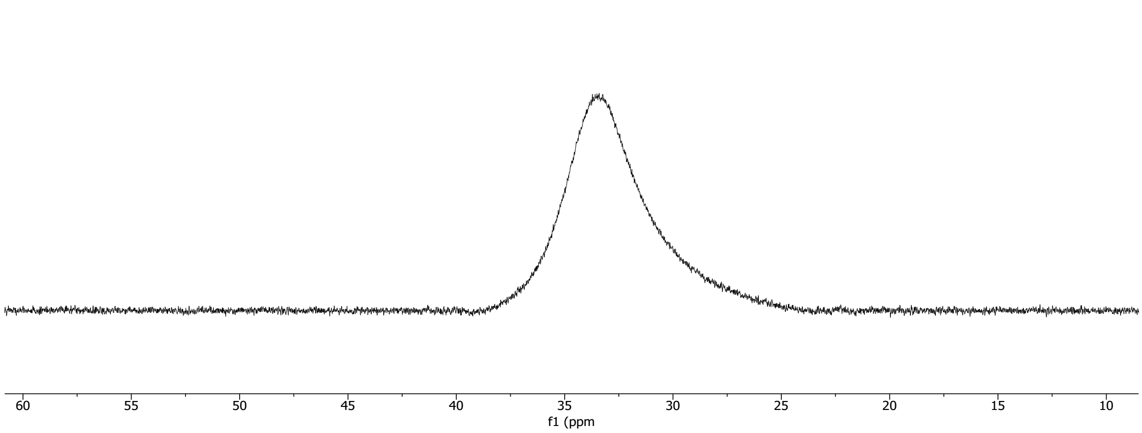

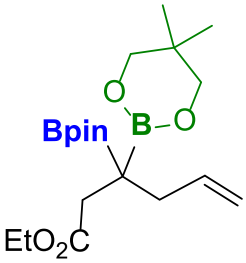

3-Bneo

^{1}H NMR (400 MHz, $CDCl_3$)

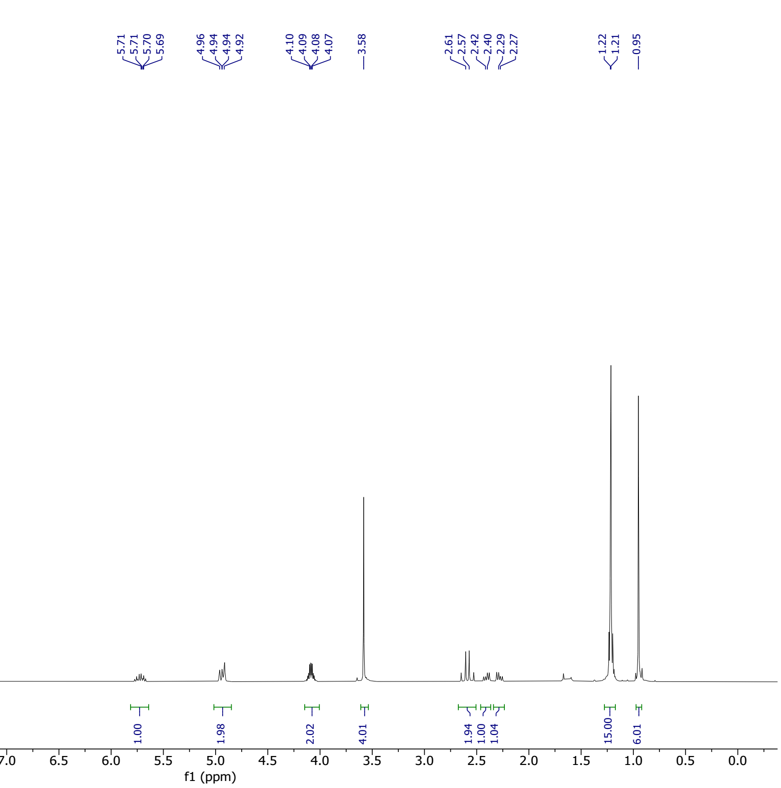

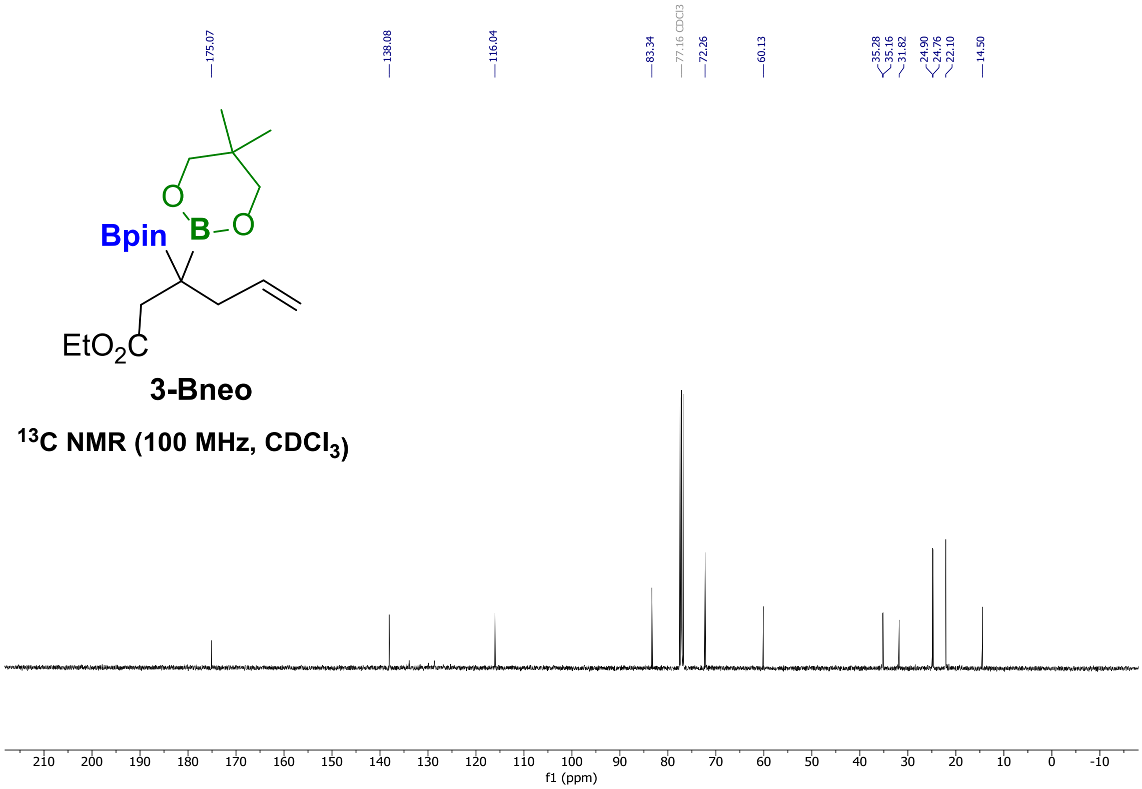
175.07
138.08
116.04
83.34
77.16 CDCl3
72.26
60.13
35.28
35.16
31.82
24.90
24.76
22.10
14.50
Bpin
B
O
O
EtO_2C
3-Bneo
^{13}C NMR (100 MHz, $CDCl_3$)
210 200 190 180 170 160 150 140 130 120 110 100 90 80 70 60 50 40 30 20 10 0 -10
f1 (ppm)

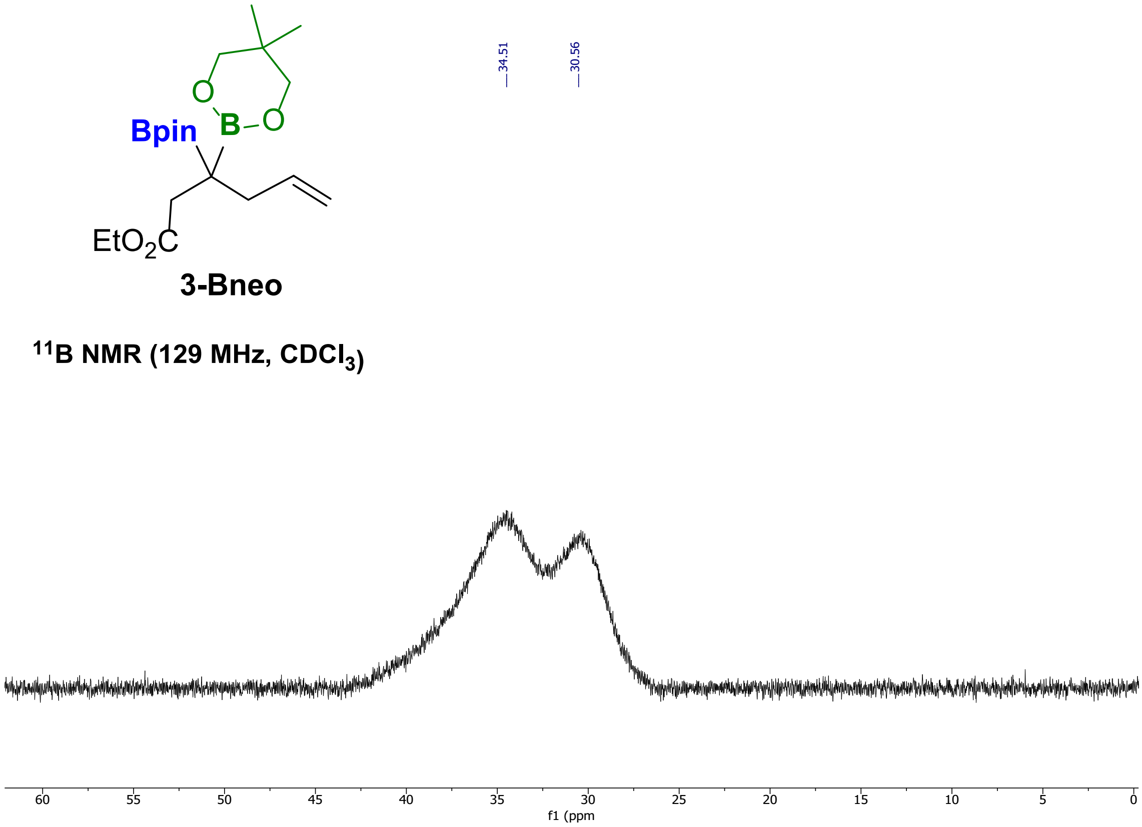
34.51
30.56
Bpin
B
O
O
EtO_2C
3-Bneo
^{11}B NMR (129 MHz, $CDCl_3$)
60 55 50 45 40 35 30 25 20 15 10 5 0
f1 (ppm

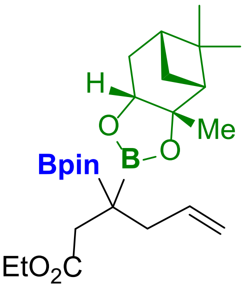

3-Bpai

^{1}H NMR (400 MHz, CDCl$_3$)

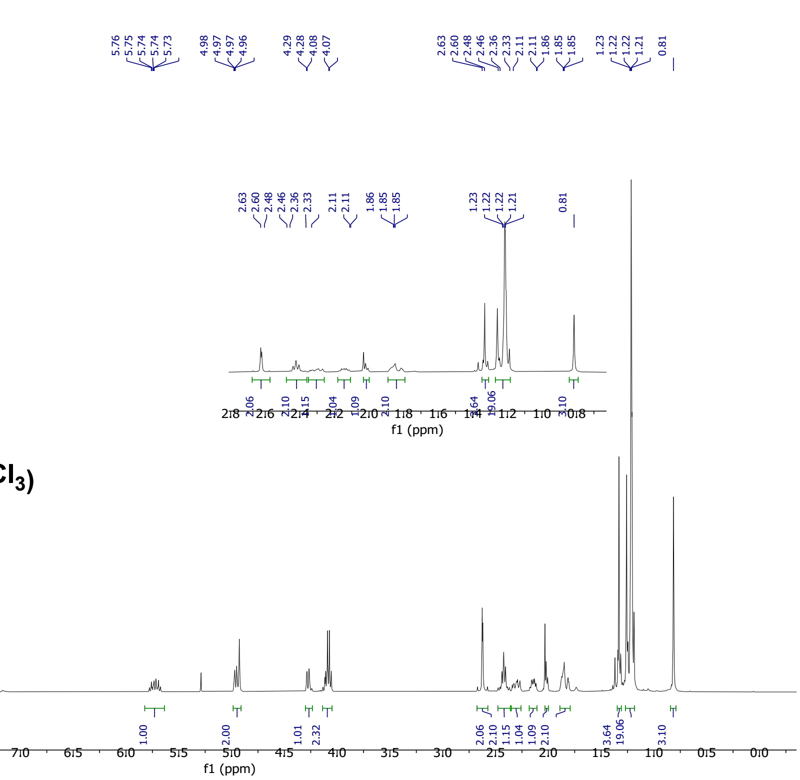

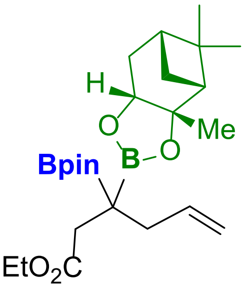

3-Bpai

^{13}C NMR (100 MHz, CDCl$_3$)

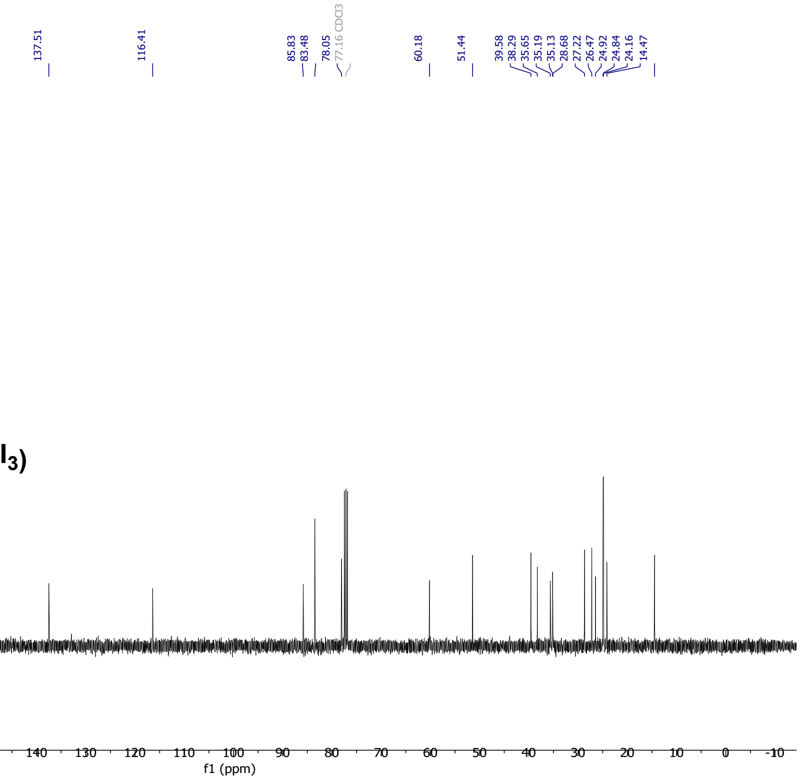

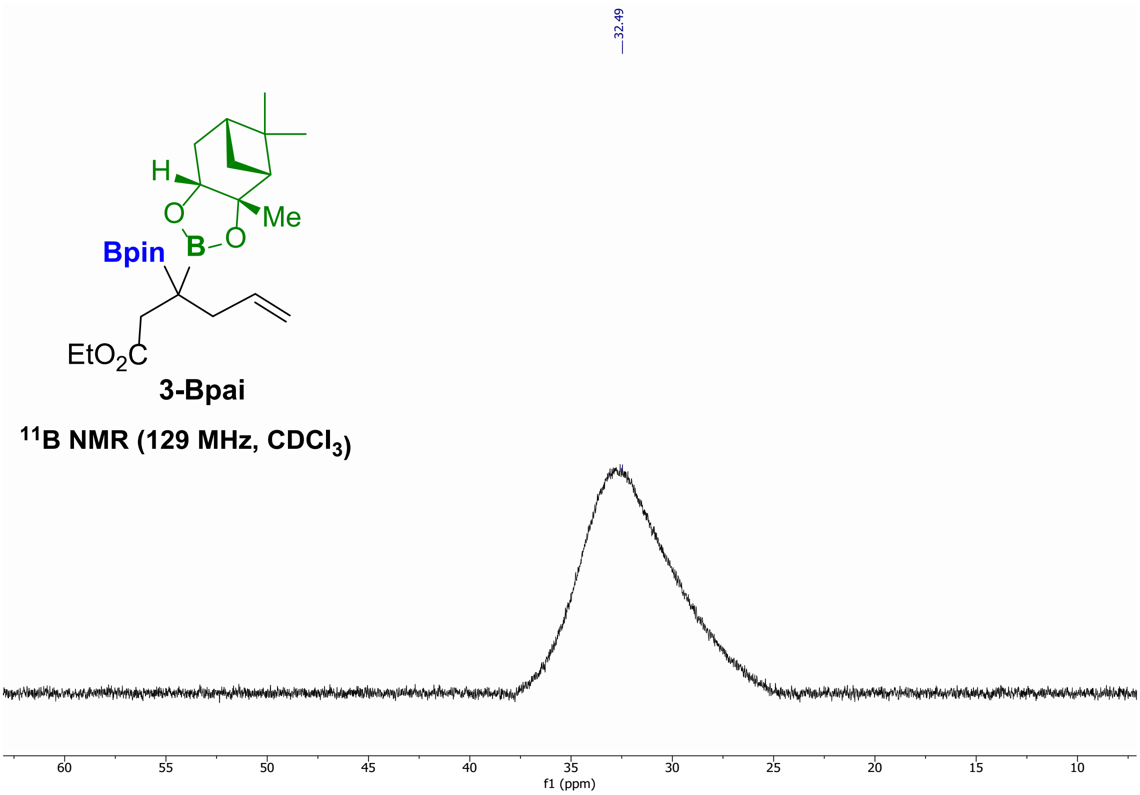
32.49
H
Me
O
B
O
Bpin
EtO2C
3-Bpai
11B NMR (129 MHz, CDCl3)
f1 (ppm)

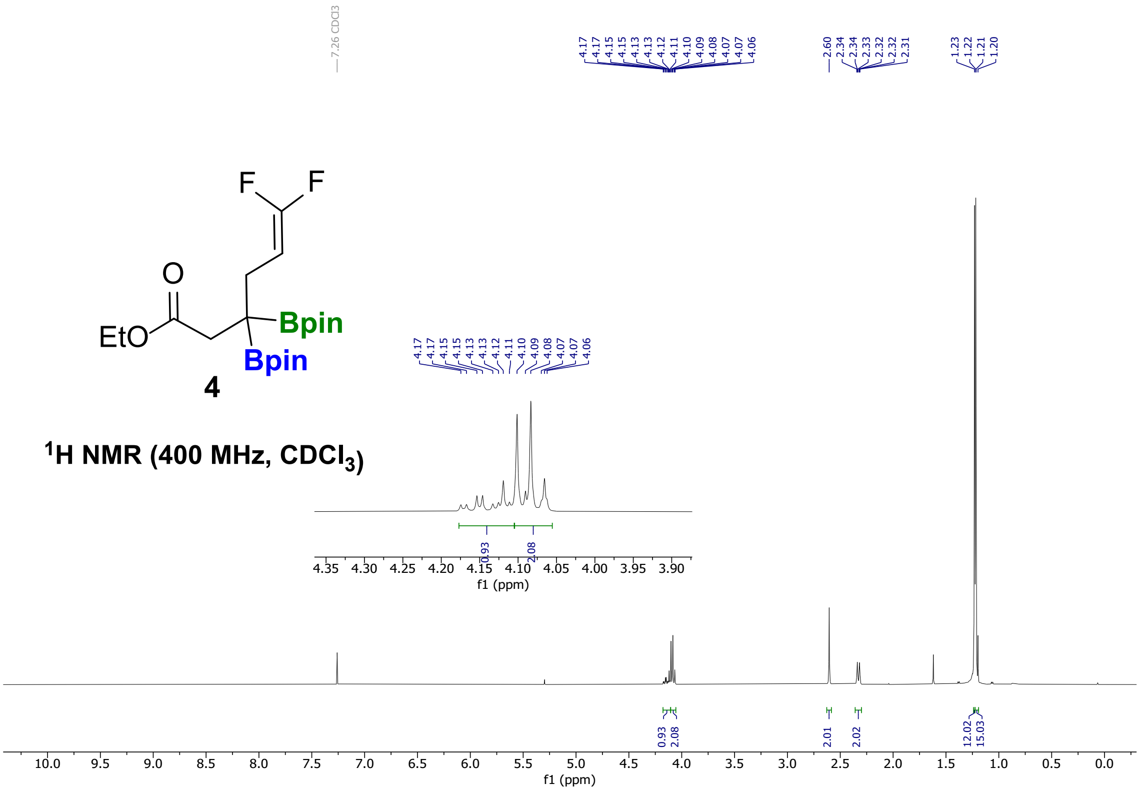
7.26 CDCl3
F
F
O
EtO
Bpin
Bpin
4
1H NMR (400 MHz, CDCl3)
0.93
2.08
2.01
2.02
12.02
15.03
f1 (ppm)

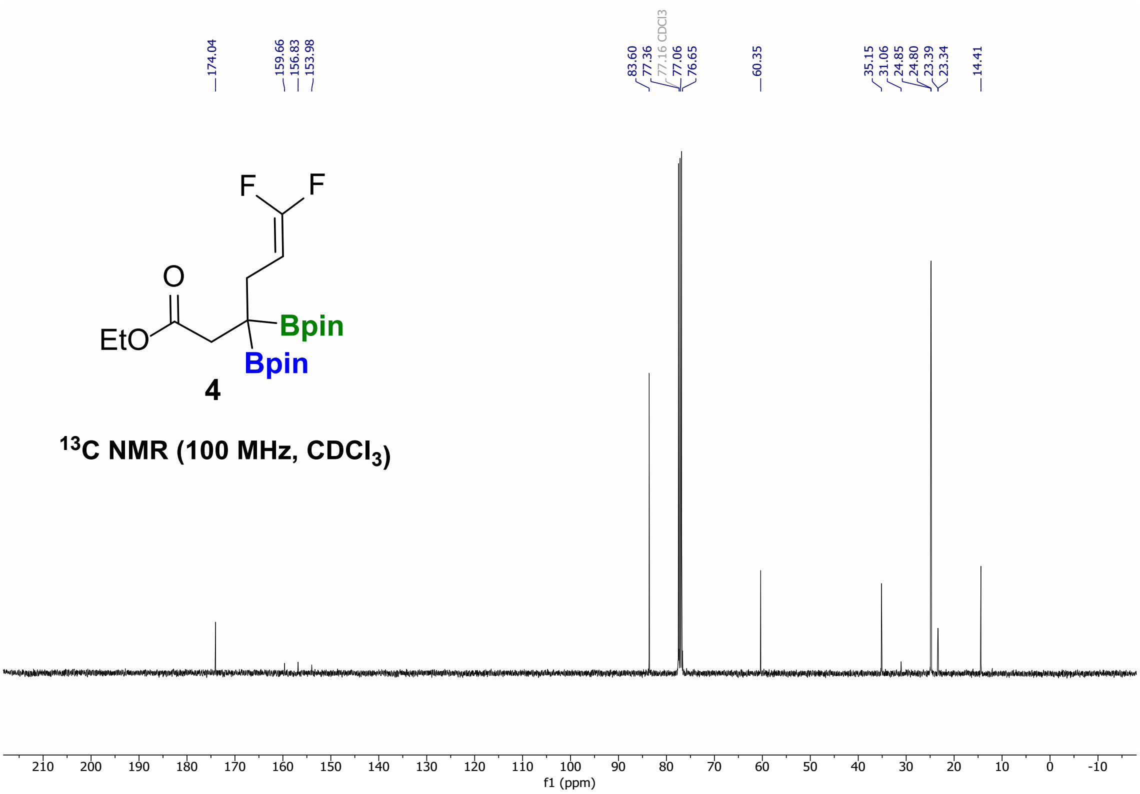
174.04
159.66
156.83
153.98
83.60
77.36
77.16 CDCl3
77.06
76.65
60.35
35.15
31.06
24.85
24.80
23.39
23.34
14.41
F
F
O
EtO
Bpin
Bpin
4
13C NMR (100 MHz, CDCl3)
f1 (ppm)

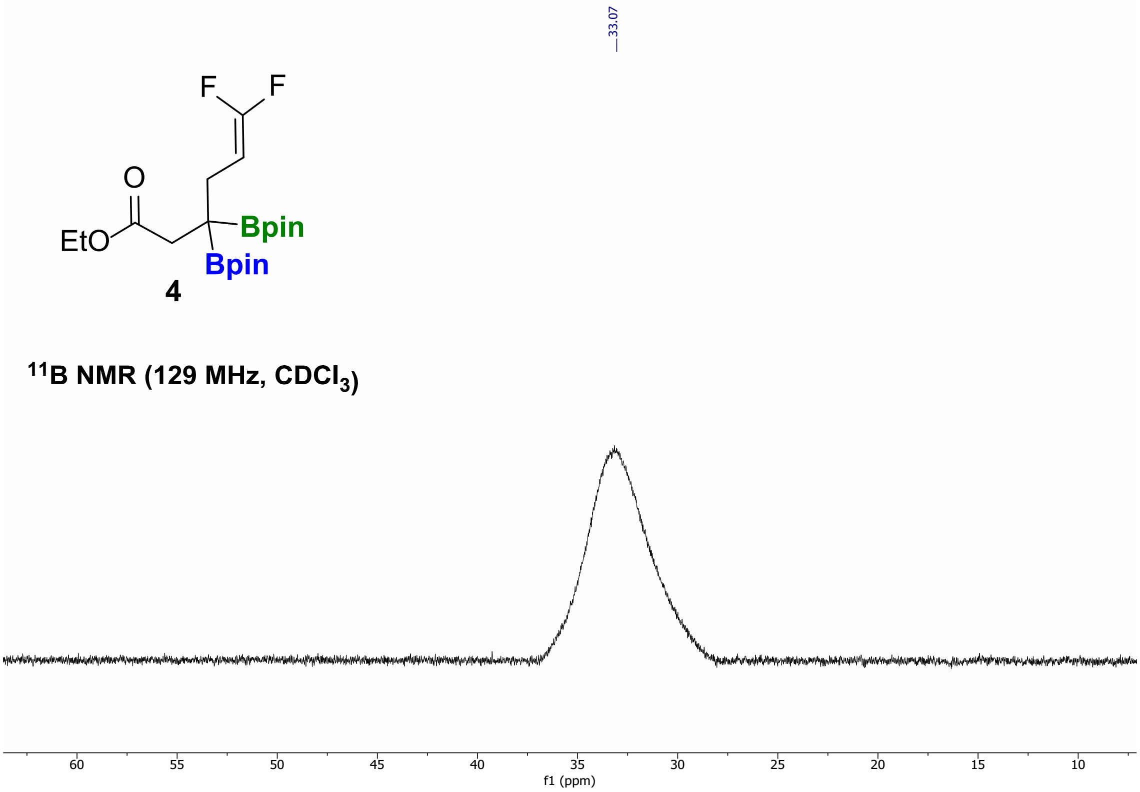
33.07
F
F
O
EtO
Bpin
Bpin
4
11B NMR (129 MHz, CDCl3)
f1 (ppm)

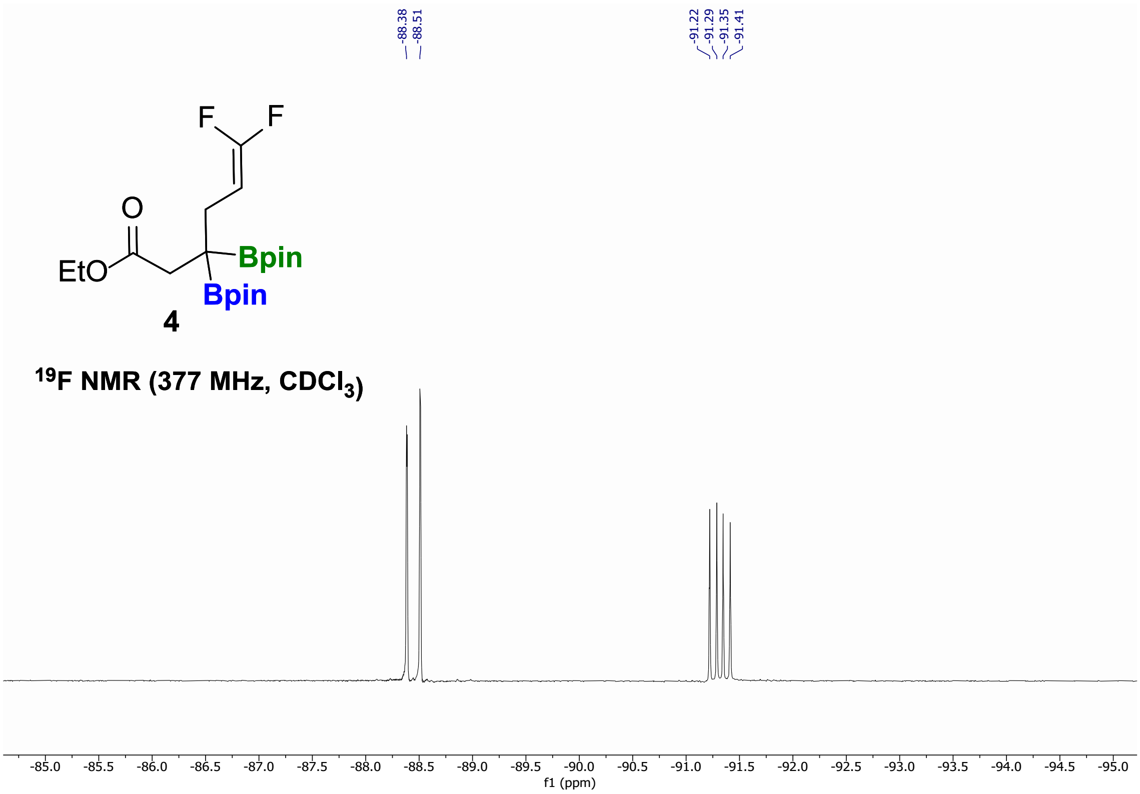
-88.38
-88.51
-91.22
-91.29
-91.35
-91.41
F
F
O
EtO
Bpin
Bpin
4
19F NMR (377 MHz, CDCl3)
f1 (ppm)

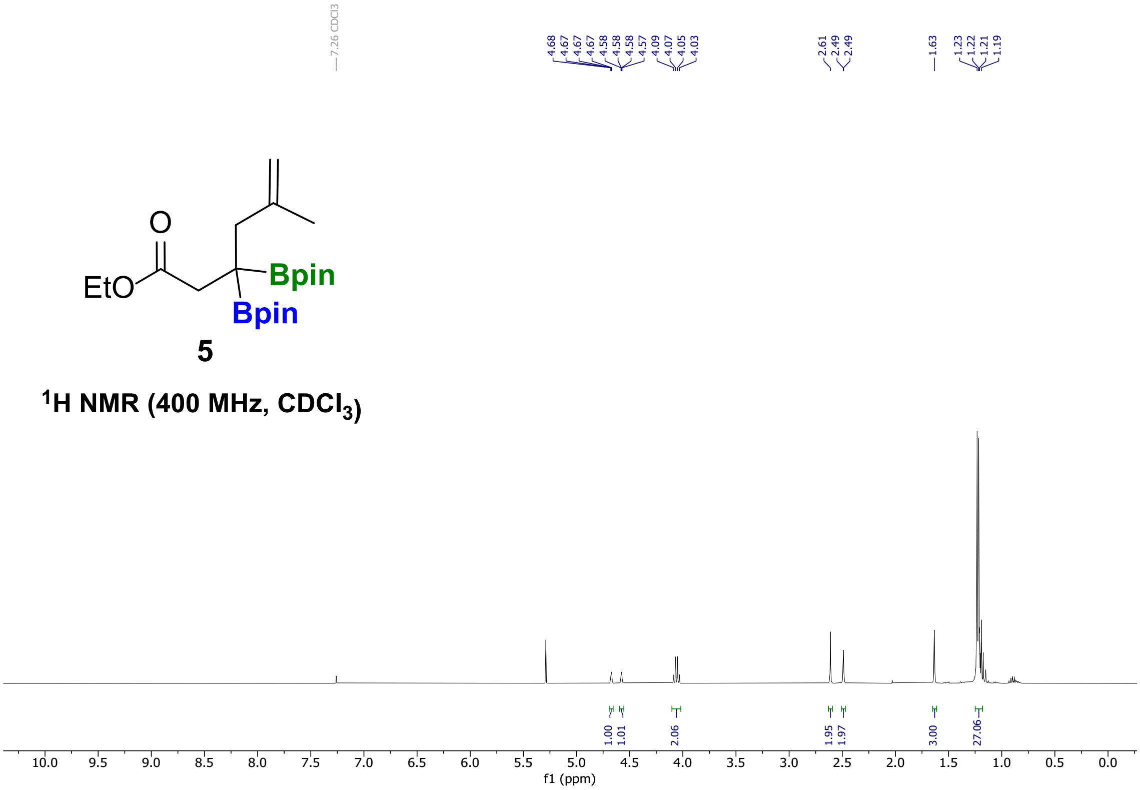
7.26 CDCl3
4.68
4.67
4.67
4.67
4.58
4.58
4.58
4.57
4.09
4.07
4.05
4.03
2.61
2.49
2.49
1.63
1.23
1.22
1.21
1.19
O
EtO
Bpin
Bpin
5
1H NMR (400 MHz, CDCl3)
1.00
1.01
2.06
1.95
1.97
3.00
27.06
f1 (ppm)

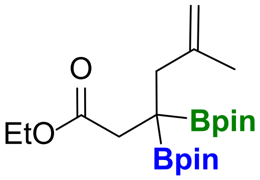

5

^{13}C NMR (100 MHz, $CDCl_3$)

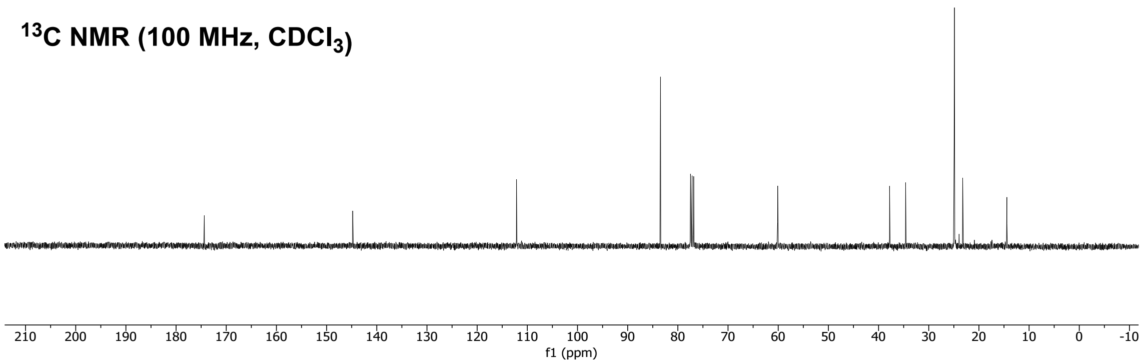

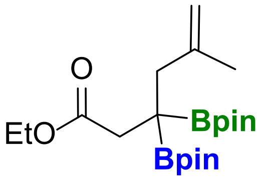

5

^{11}B NMR (129 MHz, $CDCl_3$)

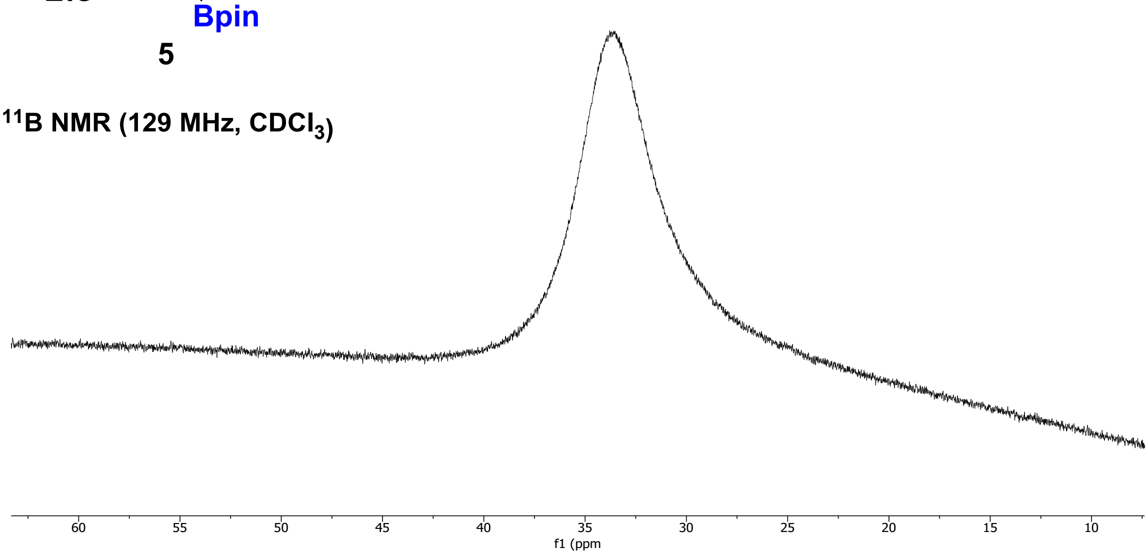

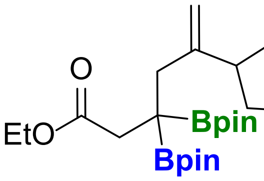

6

^{1}H NMR (400 MHz, $CDCl_3$)

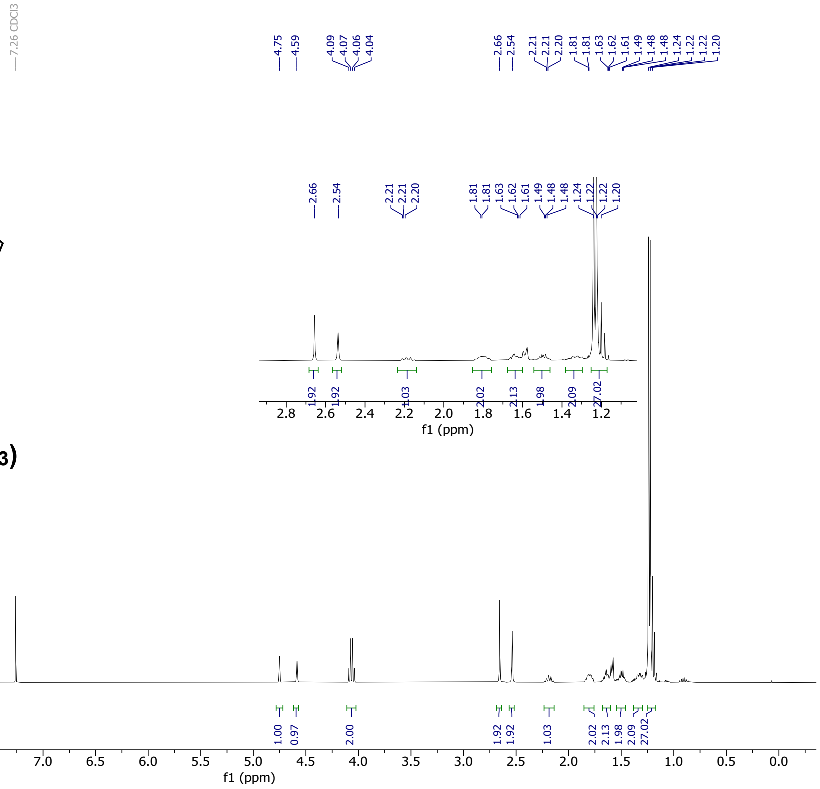

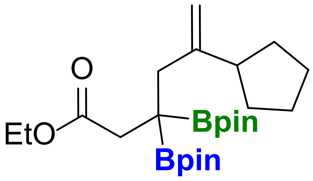

6

^{13}C NMR (100 MHz, $CDCl_3$)

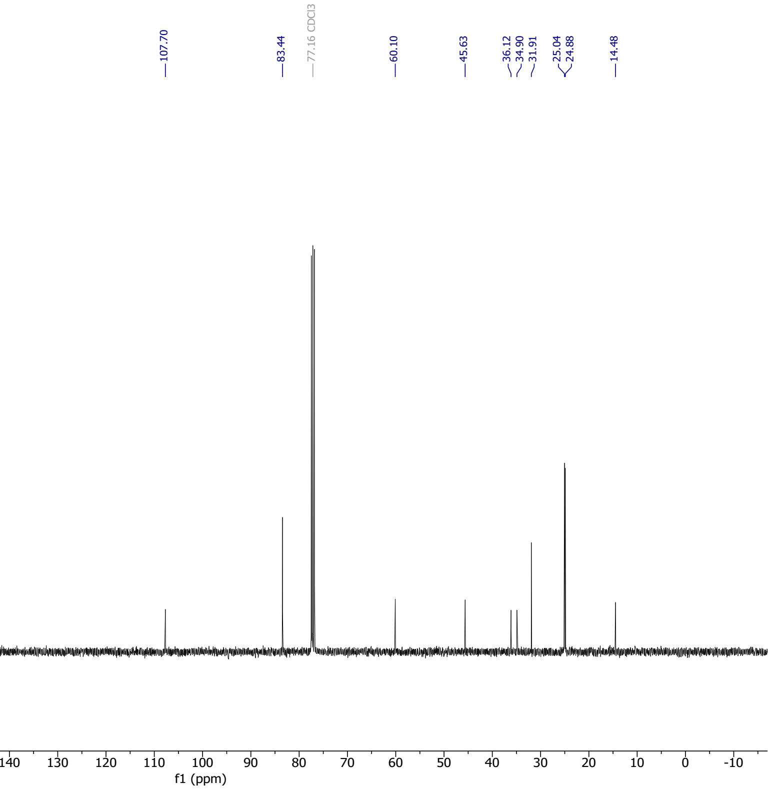

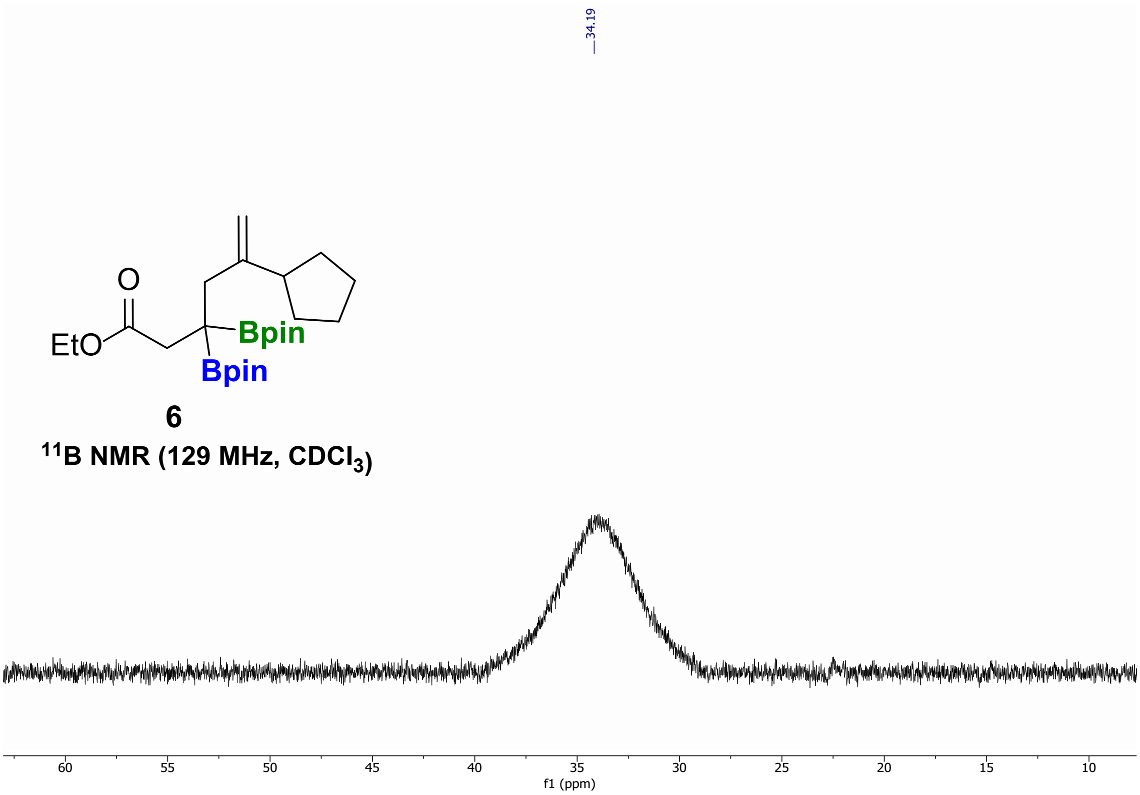
34.19
EtO
O
Bpin
Bpin
6
^{11}B NMR (129 MHz, $CDCl_3$)
f1 (ppm)

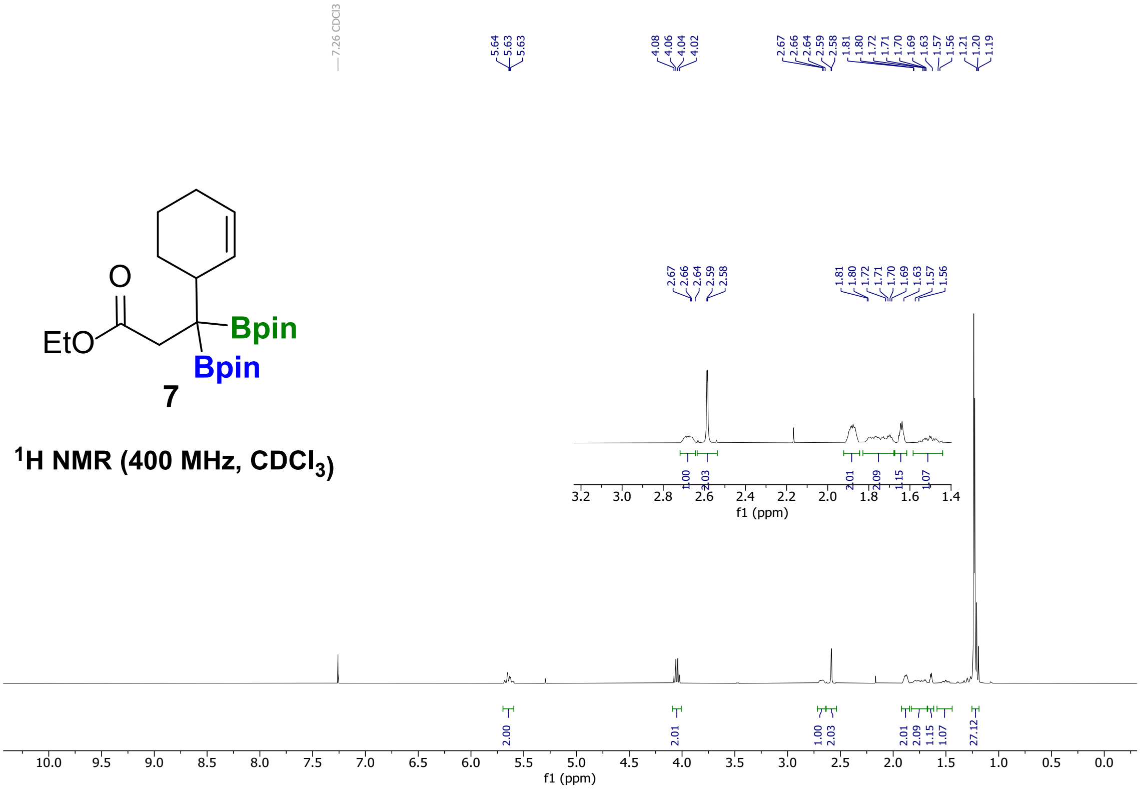
7.26 CDCl3
5.64
5.63
5.63
4.08
4.06
4.04
4.02
2.67
2.66
2.64
2.59
2.58
1.81
1.80
1.72
1.71
1.70
1.69
1.63
1.57
1.56
1.21
1.20
1.19
EtO
O
Bpin
Bpin
7
^{1}H NMR (400 MHz, $CDCl_3$)
1.00
2.03
2.01
2.09
1.15
1.07
2.00
2.01
27.12
f1 (ppm)

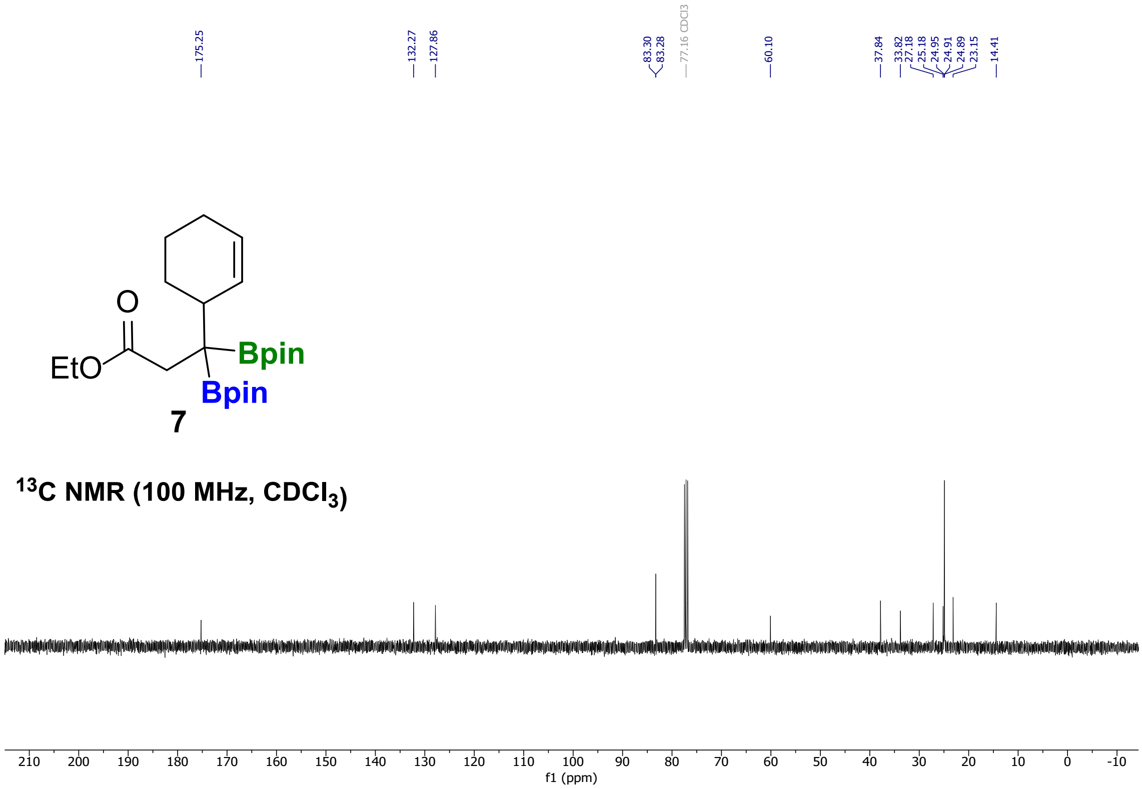
175.25
132.27
127.86
83.30
83.28
77.16 CDCl3
60.10
37.84
33.82
27.18
25.18
24.95
24.91
24.89
23.15
14.41
O
EtO
Bpin
Bpin
7
13C NMR (100 MHz, CDCl3)
f1 (ppm)

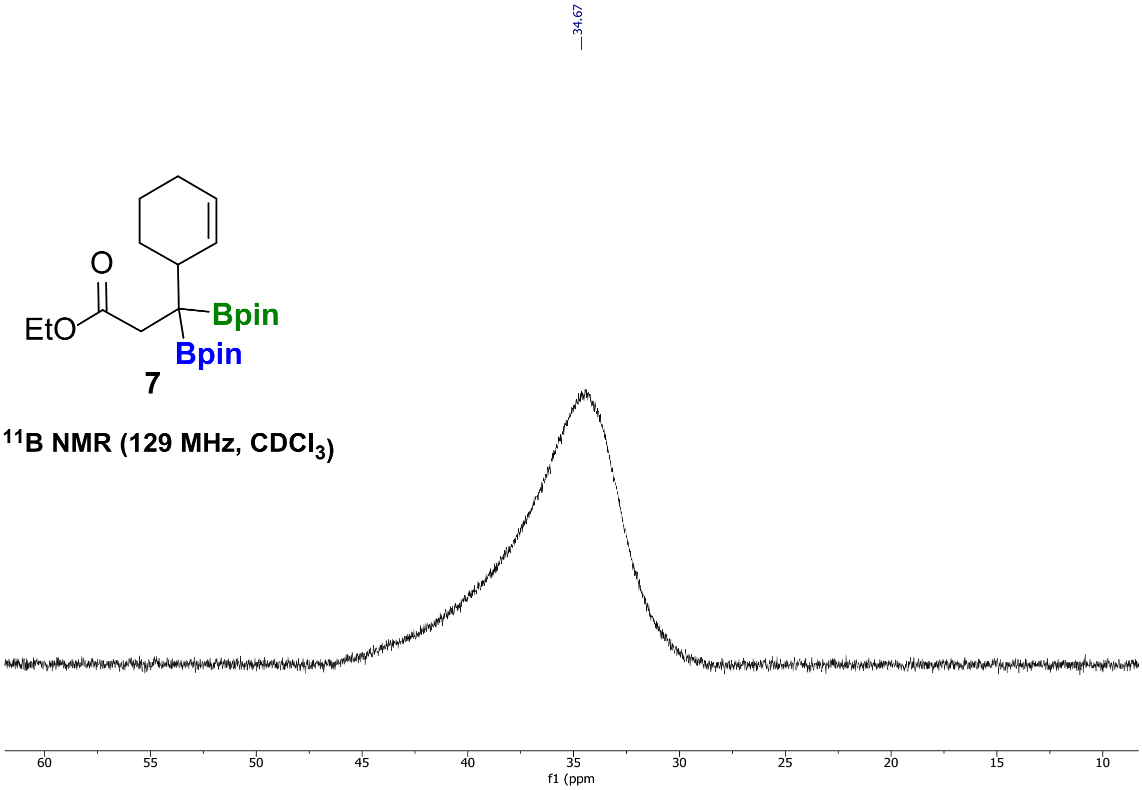
34.67
O
EtO
Bpin
Bpin
7
11B NMR (129 MHz, CDCl3)
f1 (ppm

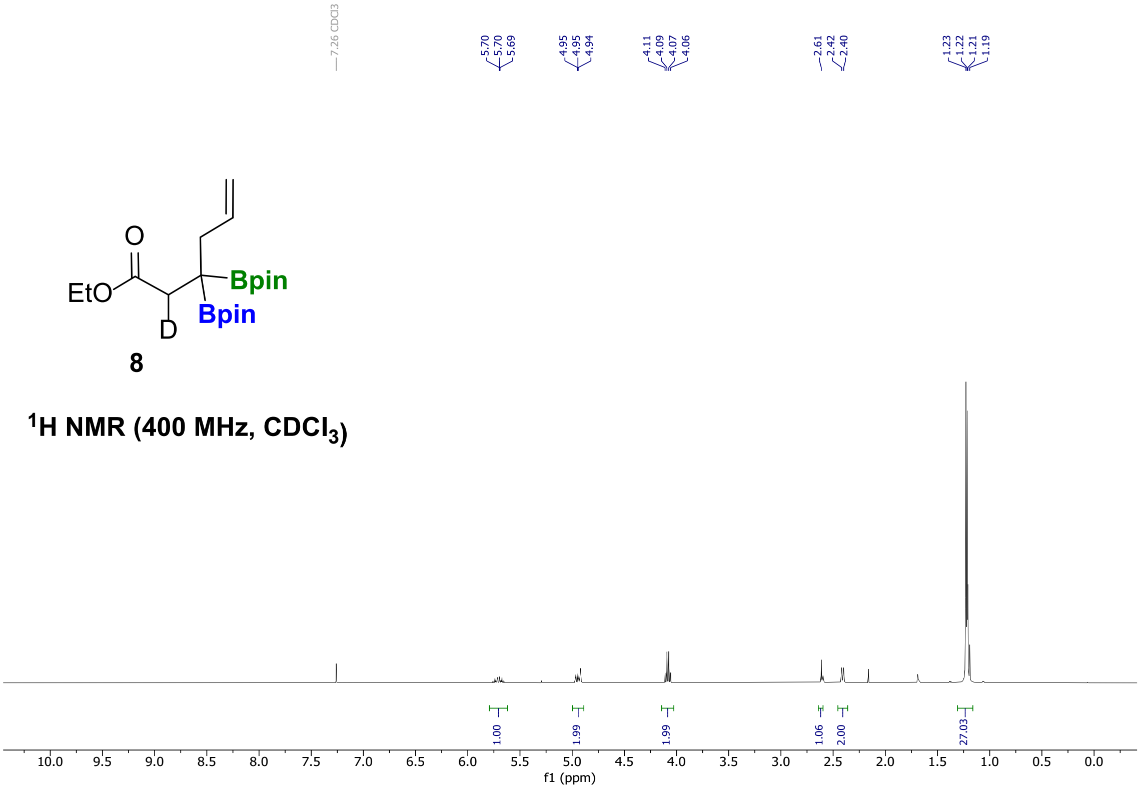

8

¹H NMR (400 MHz, $CDCl_3$)

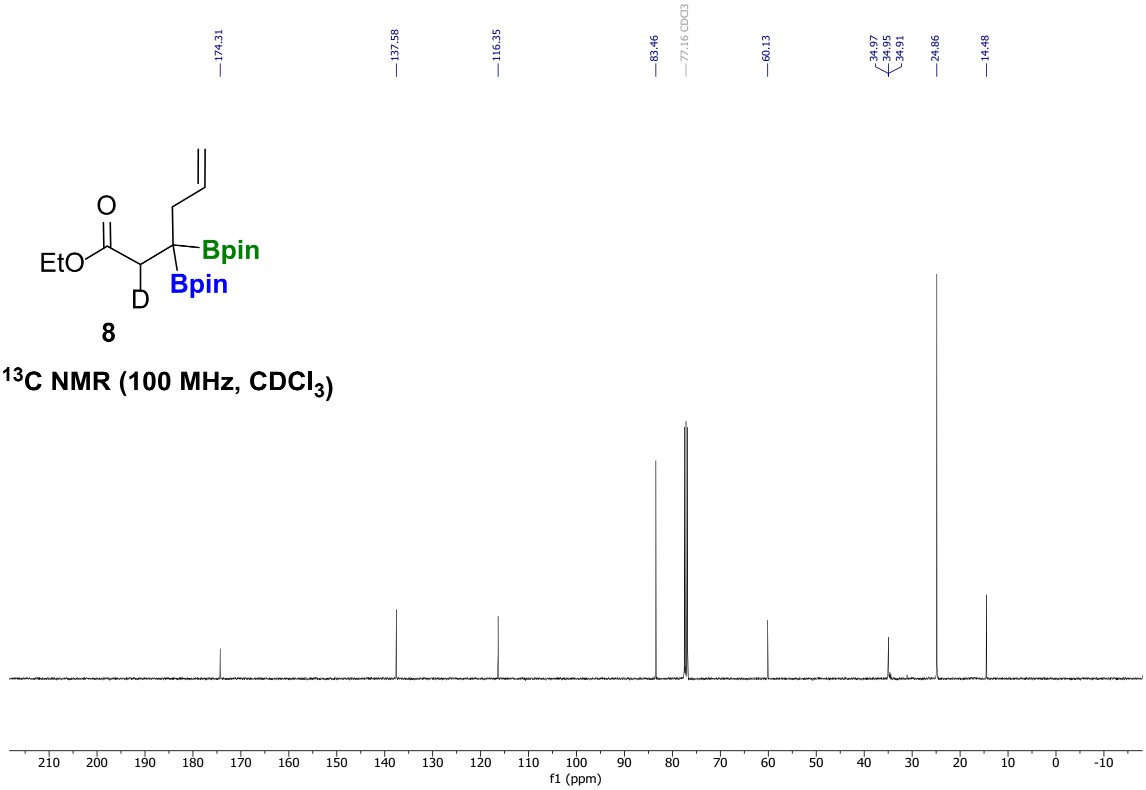

8

¹³C NMR (100 MHz, $CDCl_3$)

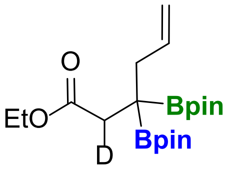

8

^{11}B NMR (129 MHz, $CDCl_3$)

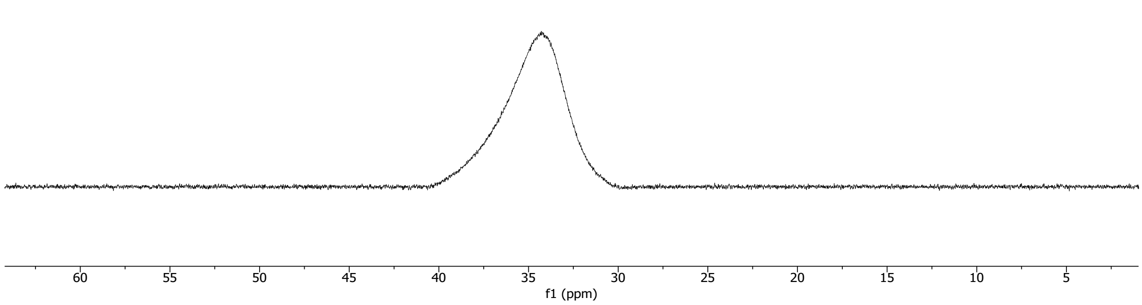

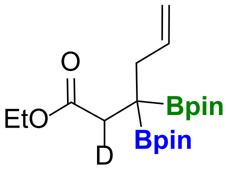

8

^{2}H NMR (61.28 MHz,$CHCl_3$)

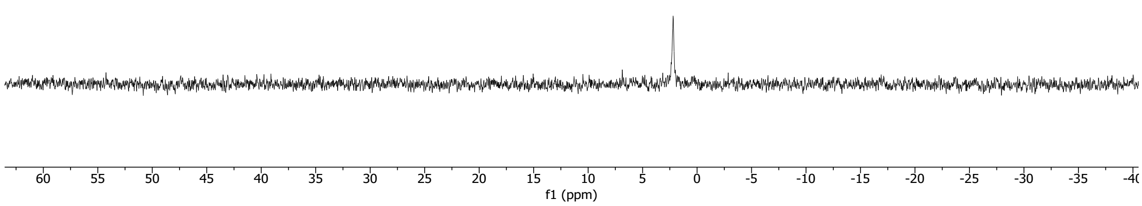

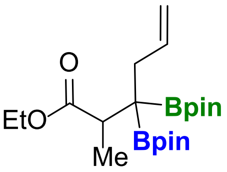

9

^{1}H NMR (400 MHz, CDCl$_3$)

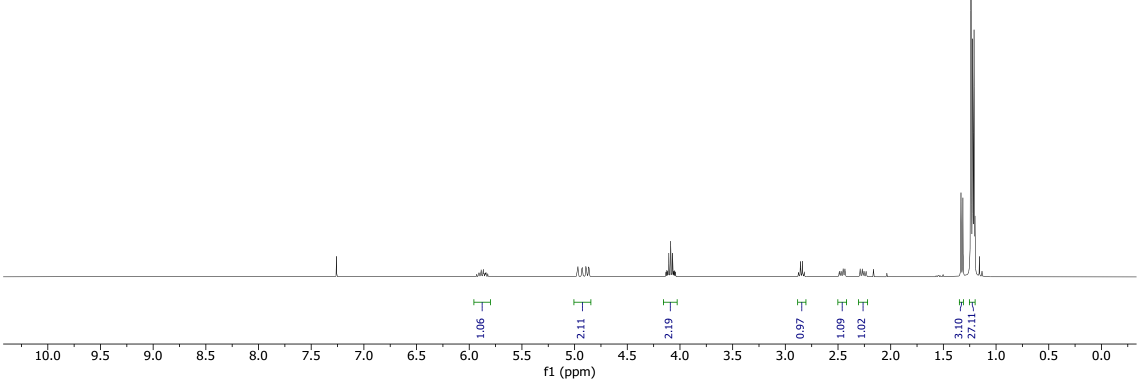

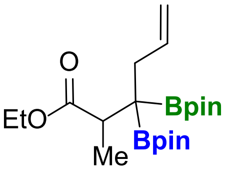

9

^{13}C NMR (100 MHz, CDCl$_3$)

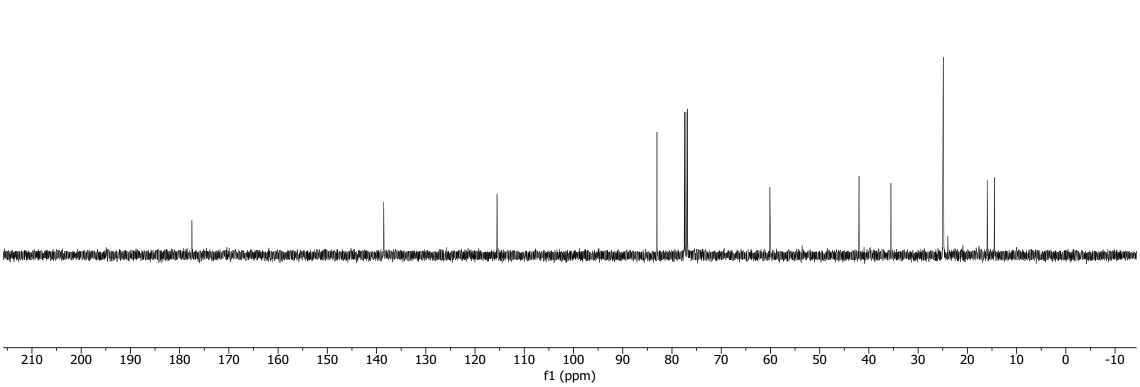

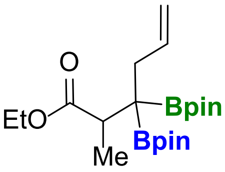

9

^{11}B NMR (129 MHz, CDCl$_3$)

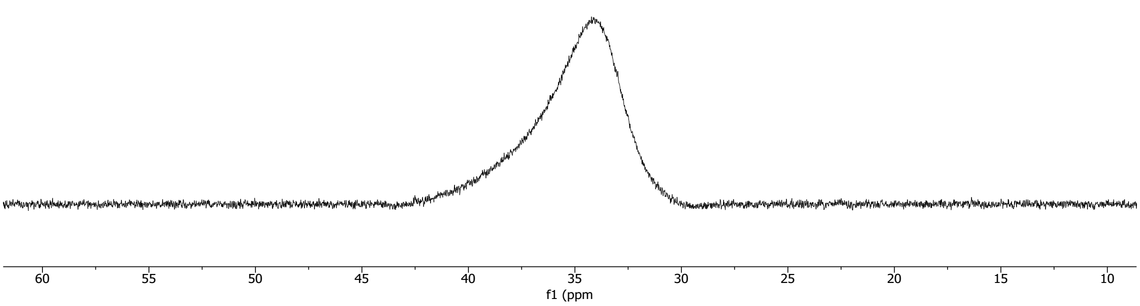

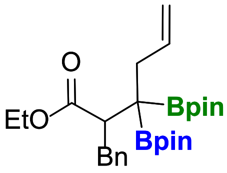

10

^{1}H NMR (400 MHz, CDCl$_3$)

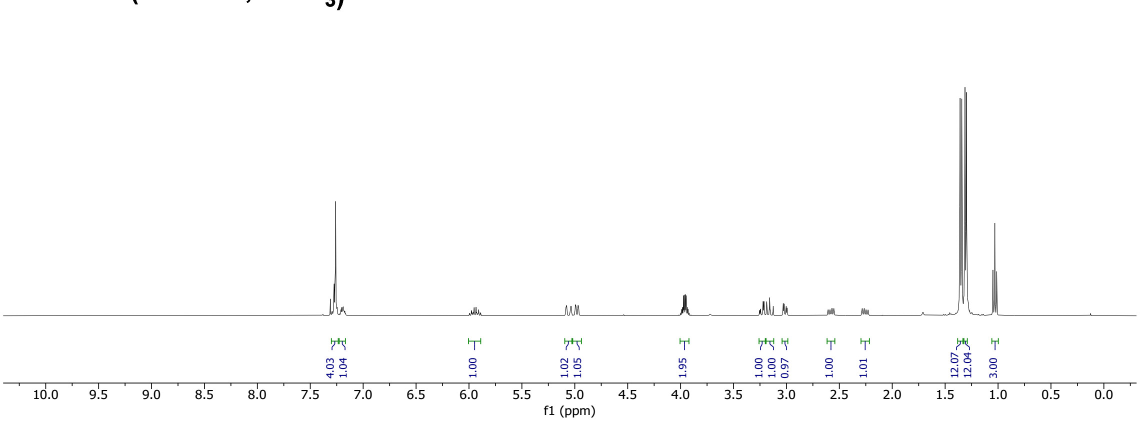

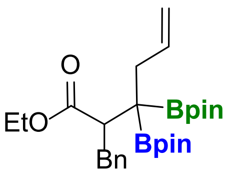

10

^{13}C NMR (100 MHz, $CDCl_3$)

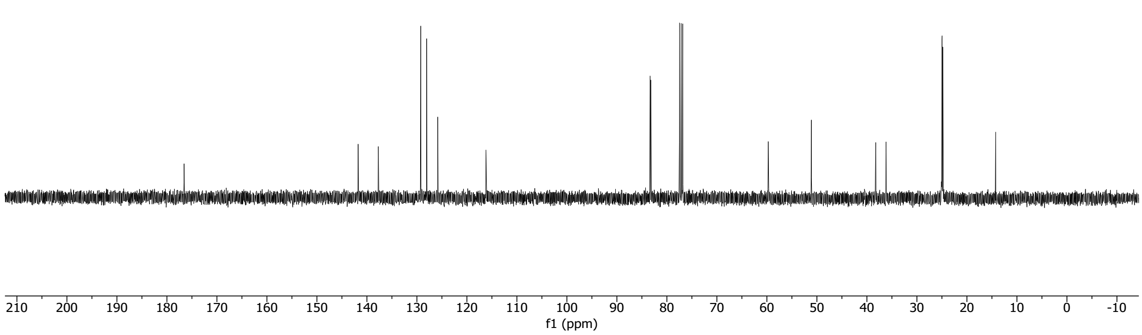

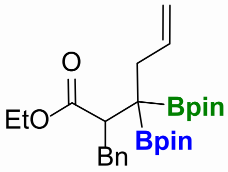

10

^{11}B NMR (129 MHz, $CDCl_3$)

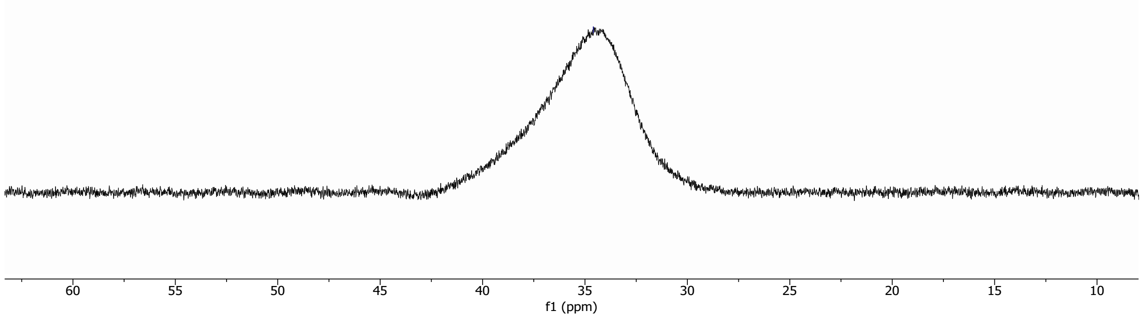

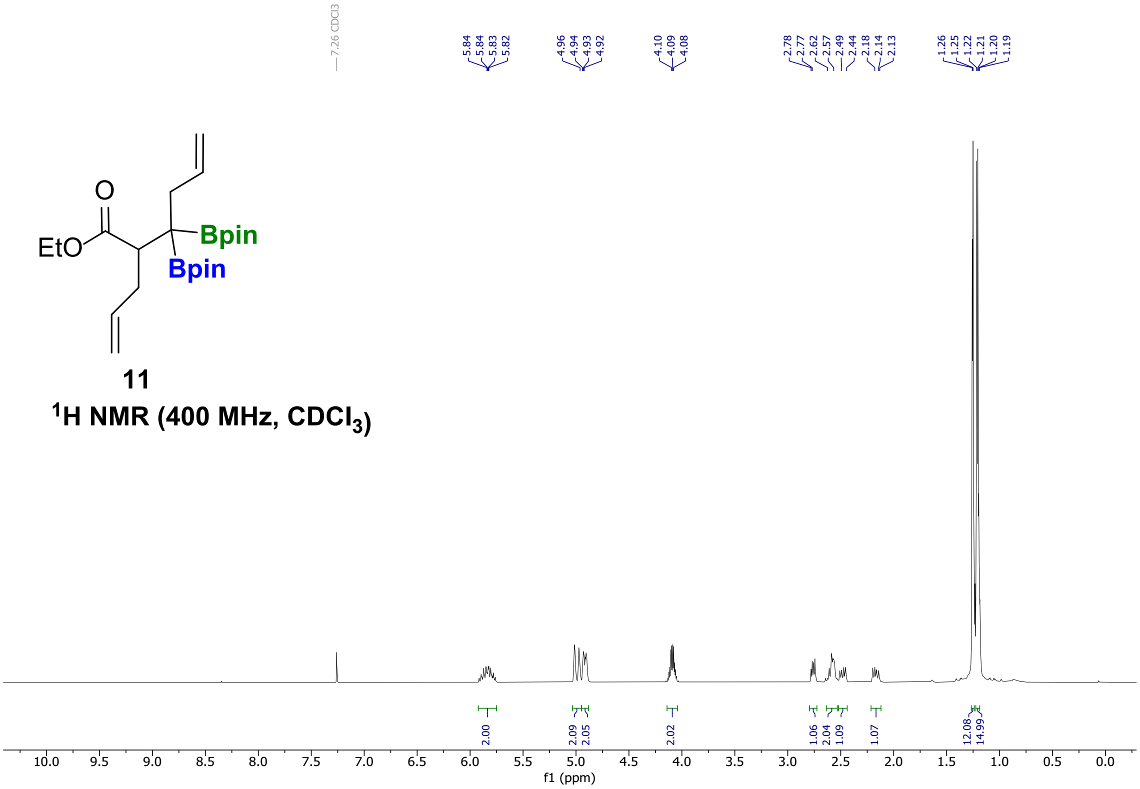
EtO
O
Bpin
Bpin
11
1H NMR (400 MHz, CDCl3)
7.26 CDCl3
f1 (ppm)

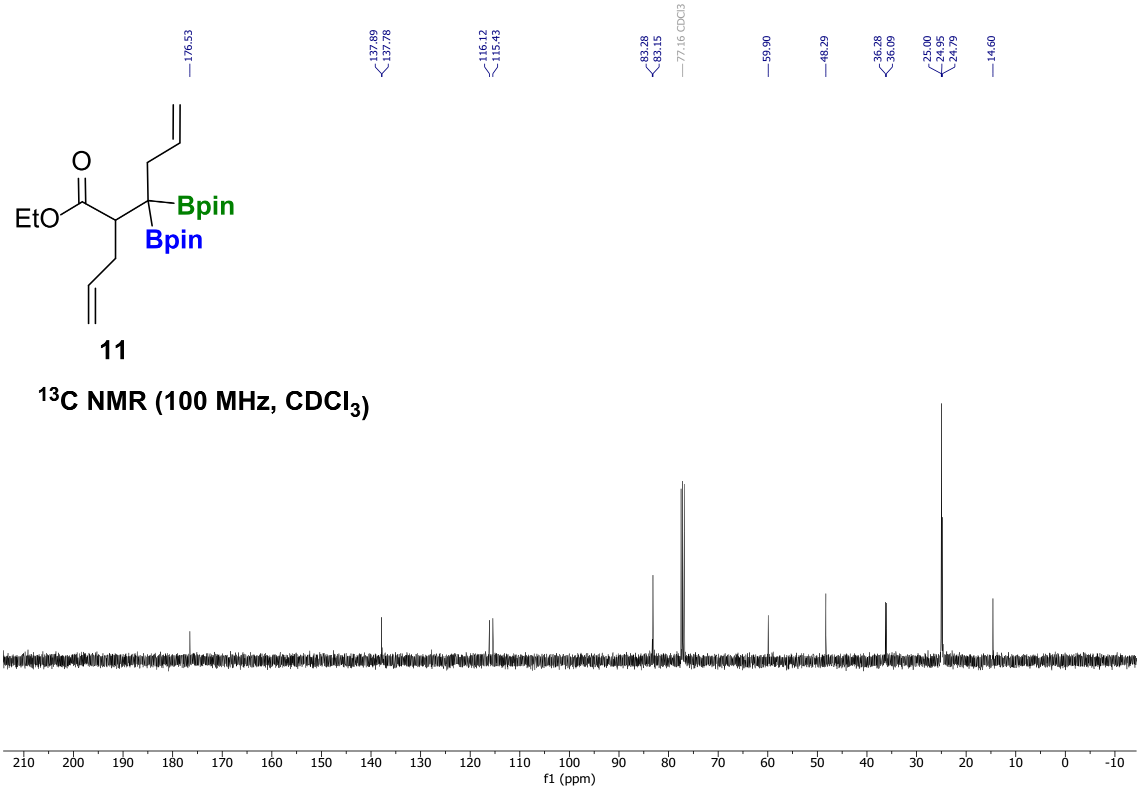
EtO
O
Bpin
Bpin
11
13C NMR (100 MHz, CDCl3)
77.16 CDCl3
f1 (ppm)

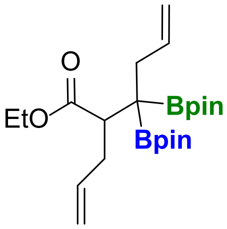

11

^{11}B NMR (129 MHz, CDCl$_3$)

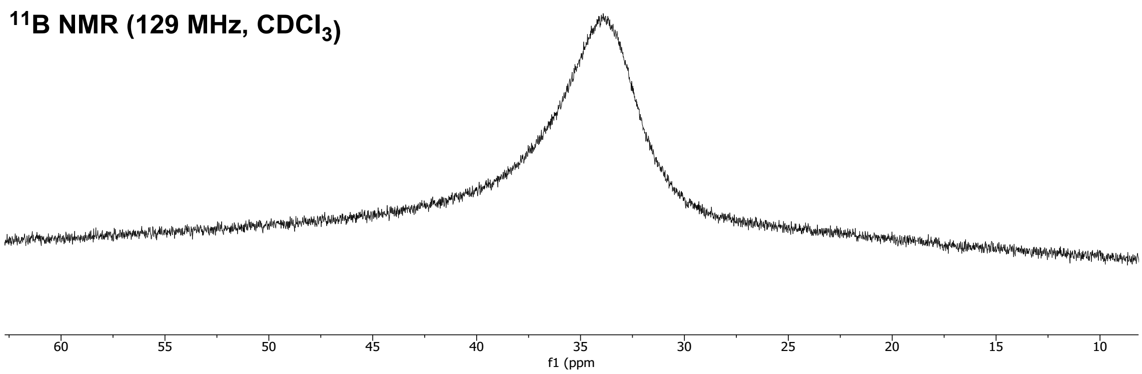

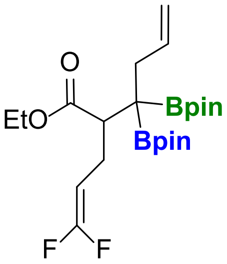

12

^{1}H NMR (400 MHz, CDCl$_3$)

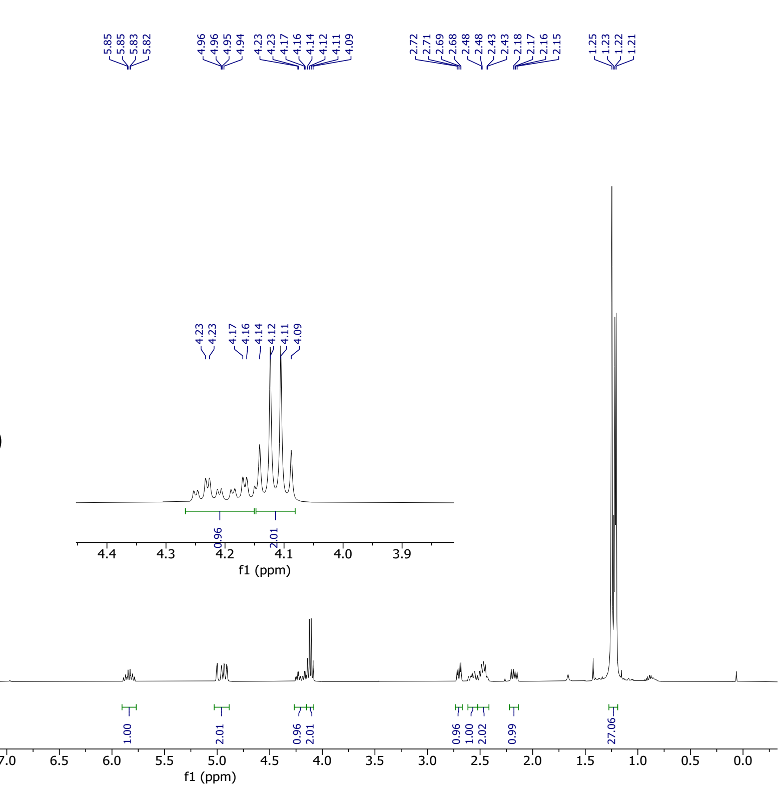

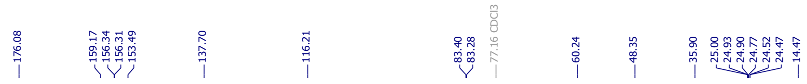

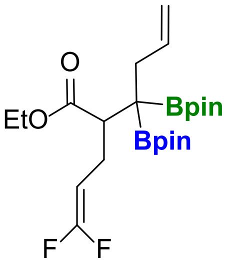

12

^{13}C NMR (100 MHz, $CDCl_3$)

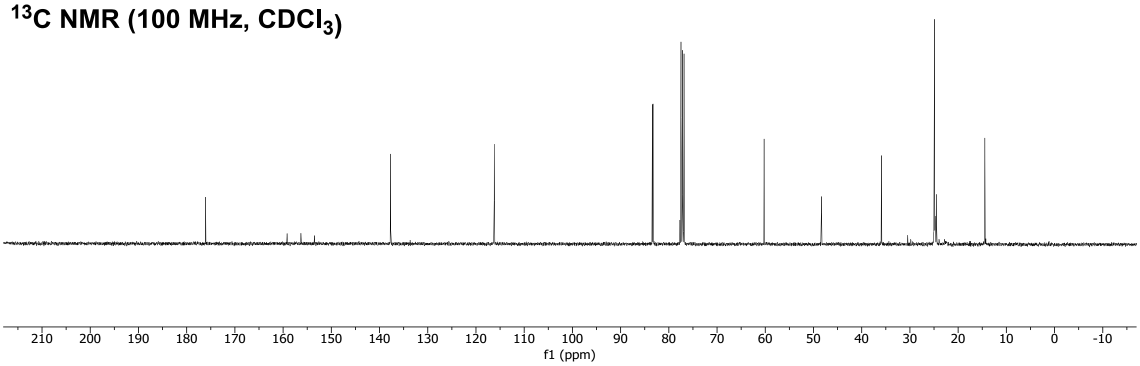

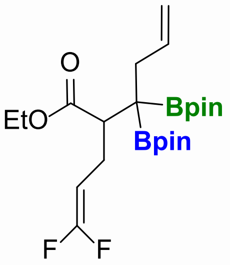

12

^{11}B NMR (129 MHz, $CDCl_3$)

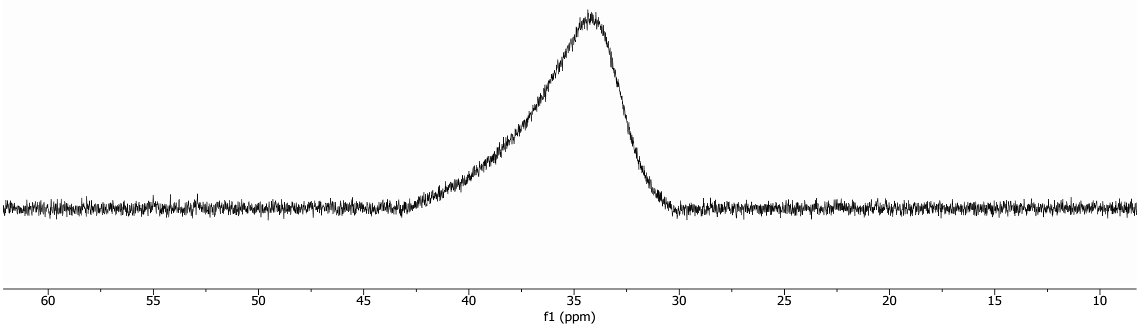

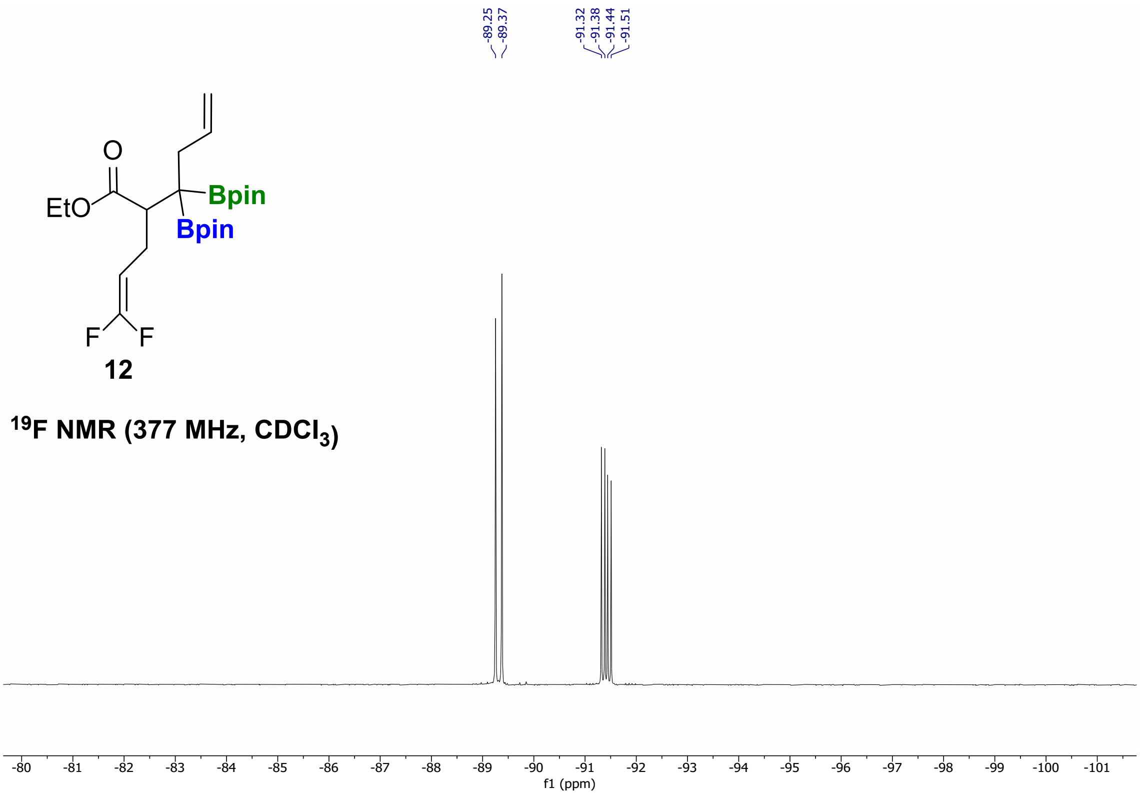
-89.25
-89.37
-91.32
-91.38
-91.44
-91.51
O
EtO
Bpin
Bpin
F
F
12
19F NMR (377 MHz, CDCl3)
-80 -81 -82 -83 -84 -85 -86 -87 -88 -89 -90 -91 -92 -93 -94 -95 -96 -97 -98 -99 -100 -101
f1 (ppm)

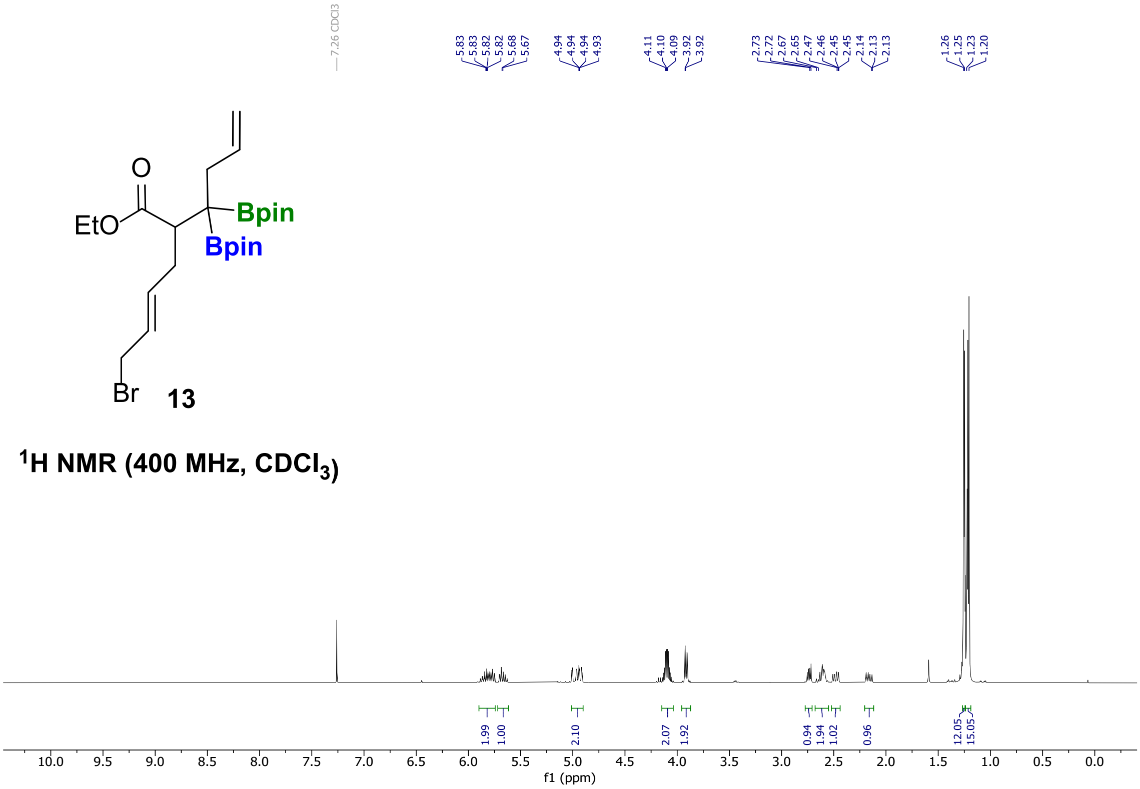
7.26 CDCl3
5.83
5.83
5.82
5.82
5.68
5.67
4.94
4.94
4.94
4.93
4.11
4.10
4.09
3.92
3.92
2.73
2.72
2.67
2.65
2.47
2.46
2.45
2.45
2.14
2.13
2.13
1.26
1.25
1.23
1.20
O
EtO
Bpin
Bpin
Br
13
1H NMR (400 MHz, CDCl3)
1.99
1.00
2.10
2.07
1.92
0.94
1.94
1.02
0.96
12.05
15.05
10.0 9.5 9.0 8.5 8.0 7.5 7.0 6.5 6.0 5.5 5.0 4.5 4.0 3.5 3.0 2.5 2.0 1.5 1.0 0.5 0.0
f1 (ppm)

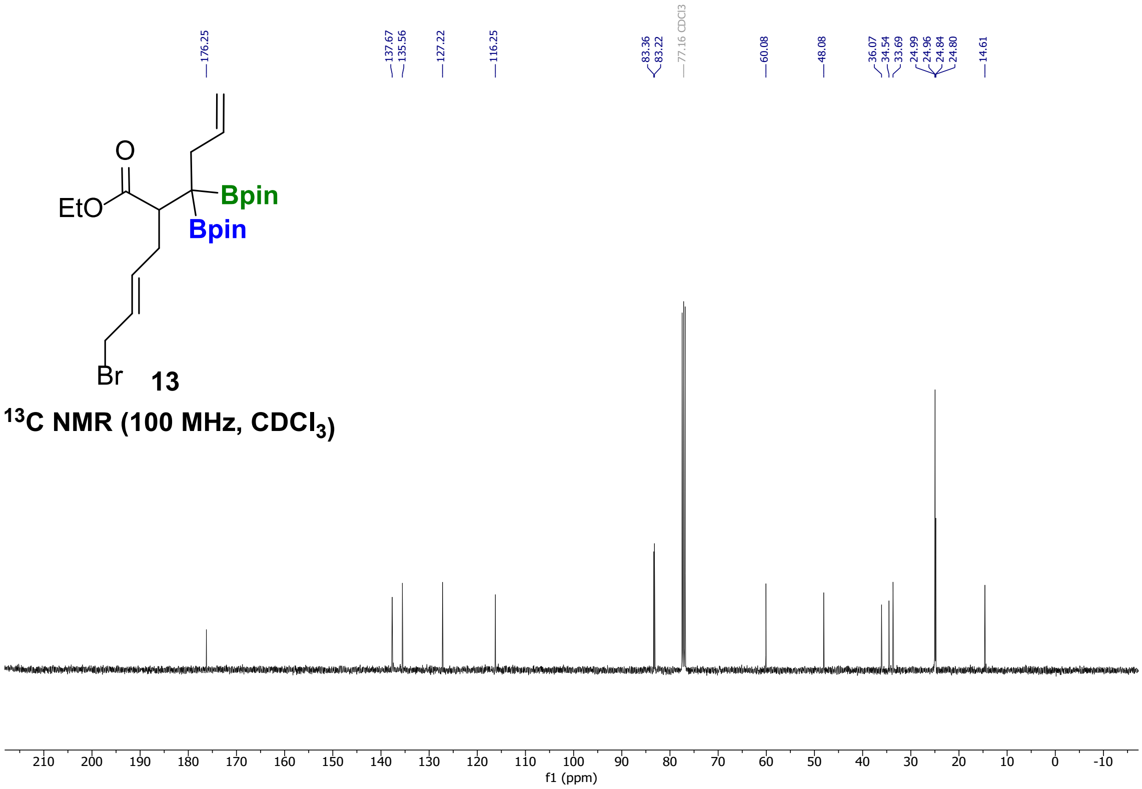
176.25
137.67
135.56
127.22
116.25
83.36
83.22
77.16 CDCl3
60.08
48.08
36.07
34.54
33.69
24.99
24.96
24.84
24.80
14.61
O
EtO
Bpin
Bpin
Br
13
^{13}C NMR (100 MHz, CDCl$_3$)
210 200 190 180 170 160 150 140 130 120 110 100 90 80 70 60 50 40 30 20 10 0 -10
f1 (ppm)

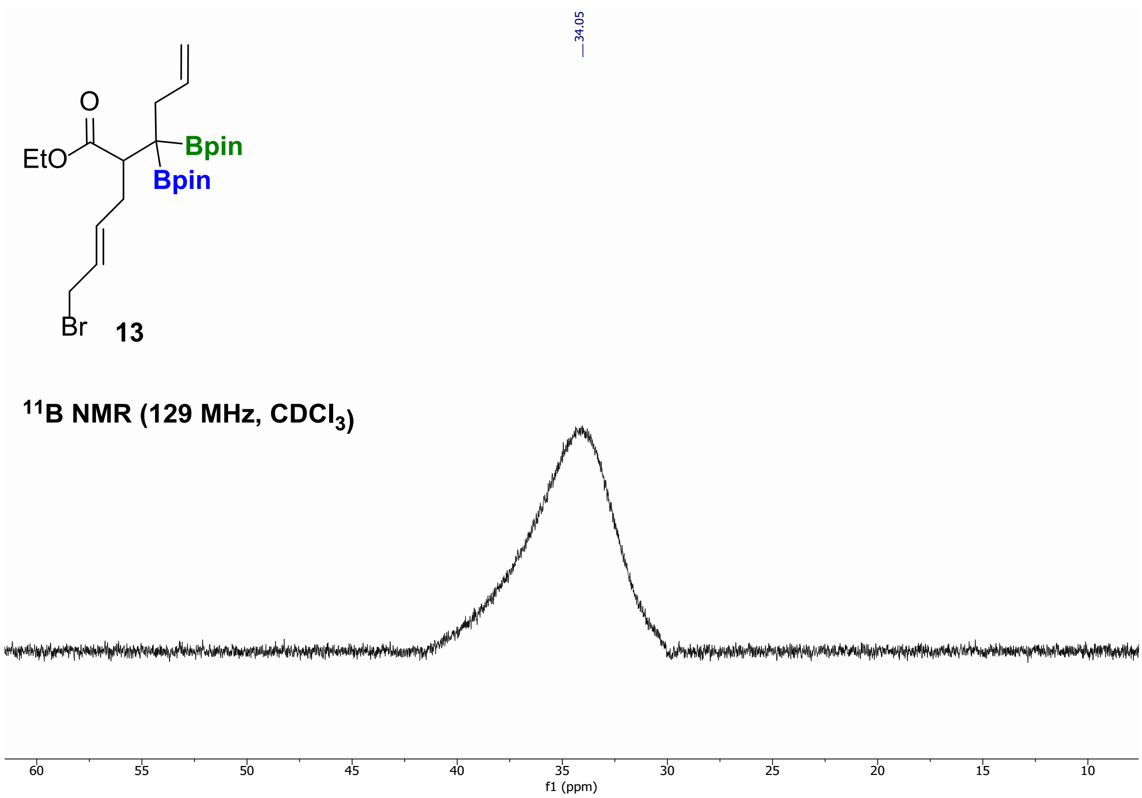
34.05
O
EtO
Bpin
Bpin
Br
13
^{11}B NMR (129 MHz, CDCl$_3$)
60 55 50 45 40 35 30 25 20 15 10
f1 (ppm)

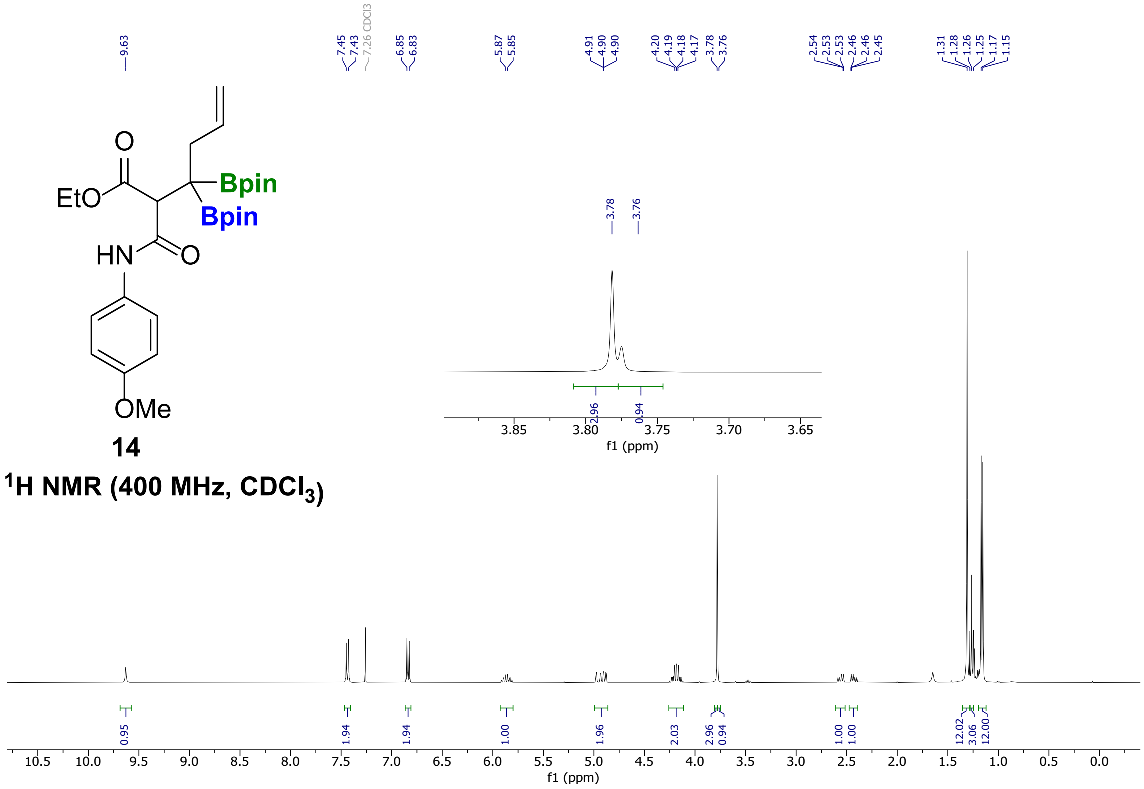

14

^{1}H NMR (400 MHz, $CDCl_3$)

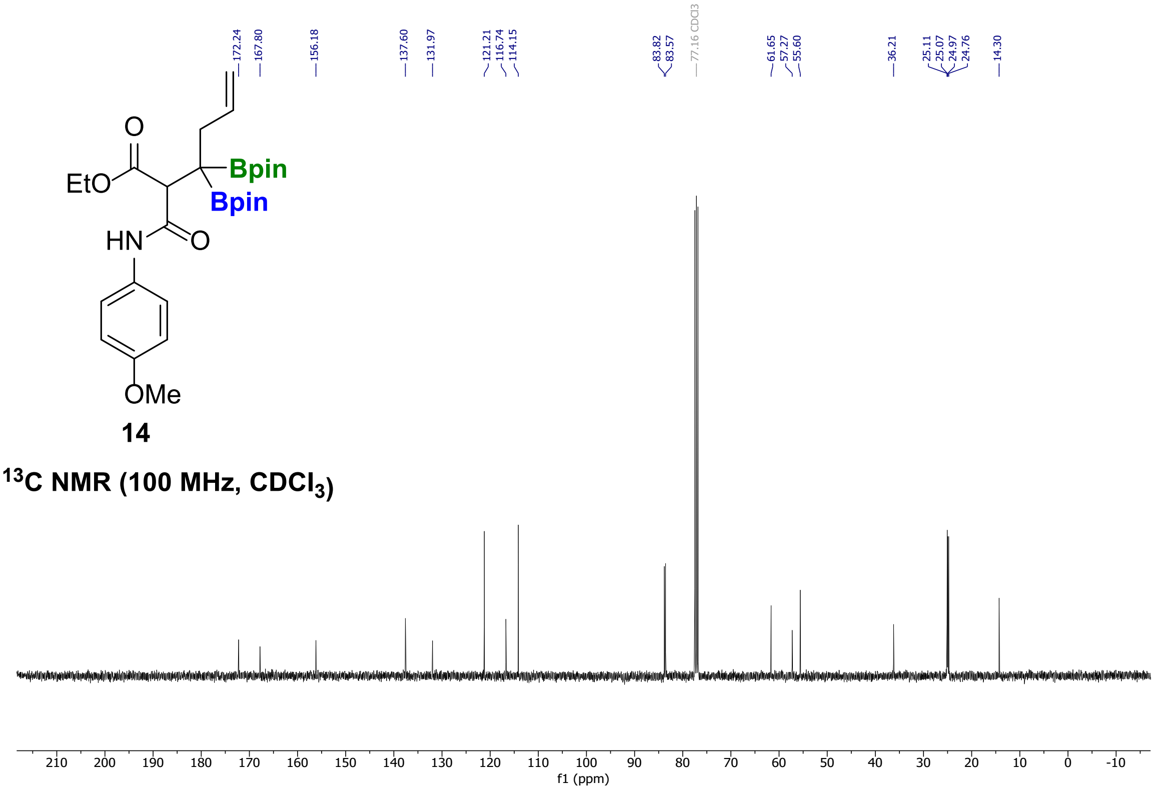

14

^{13}C NMR (100 MHz, $CDCl_3$)

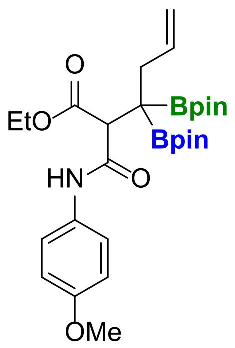

14

^{11}B NMR (129 MHz, $CDCl_3$)

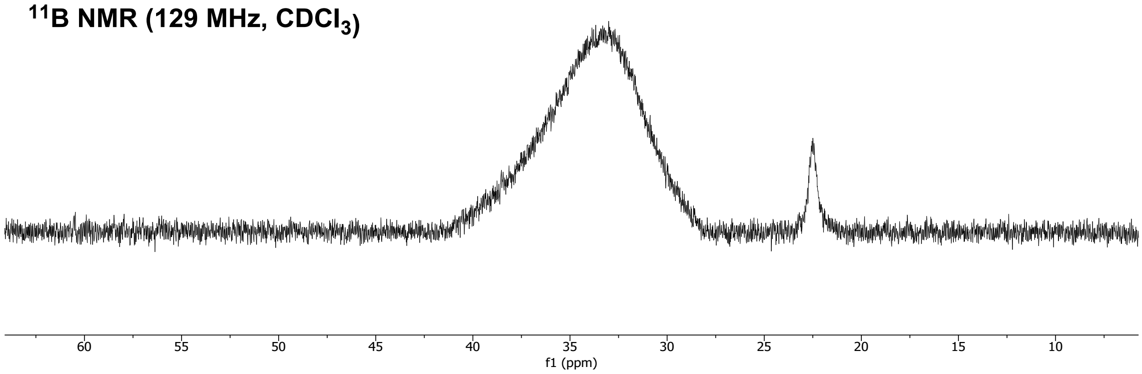

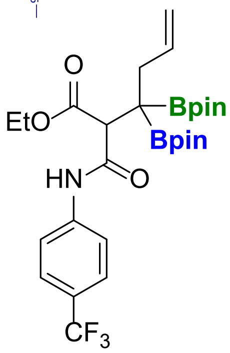

15

^{1}H NMR (400 MHz, $CDCl_3$)

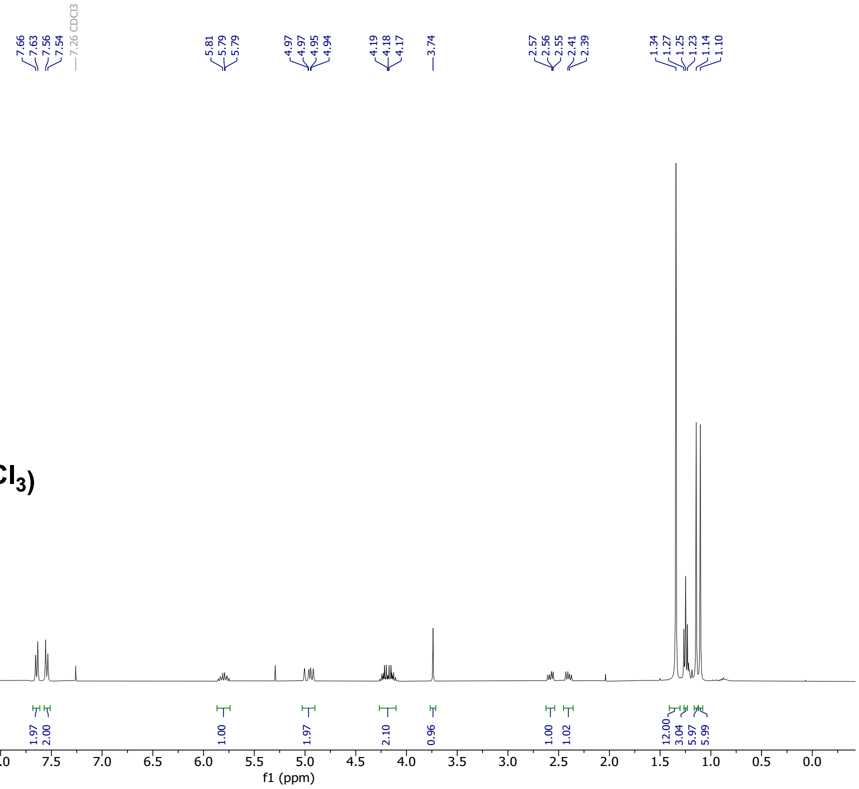

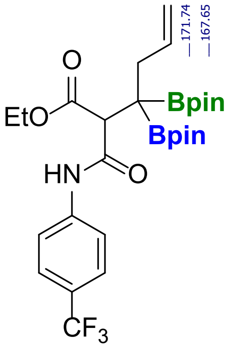

15

^{13}C NMR (100 MHz, $CDCl_3$)

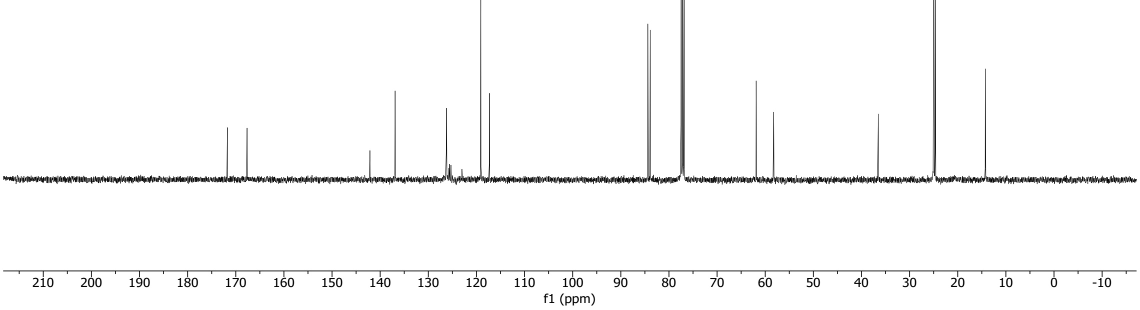

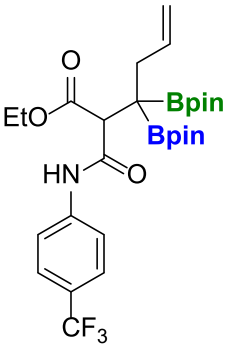

15

^{11}B NMR (129 MHz, $CDCl_3$)

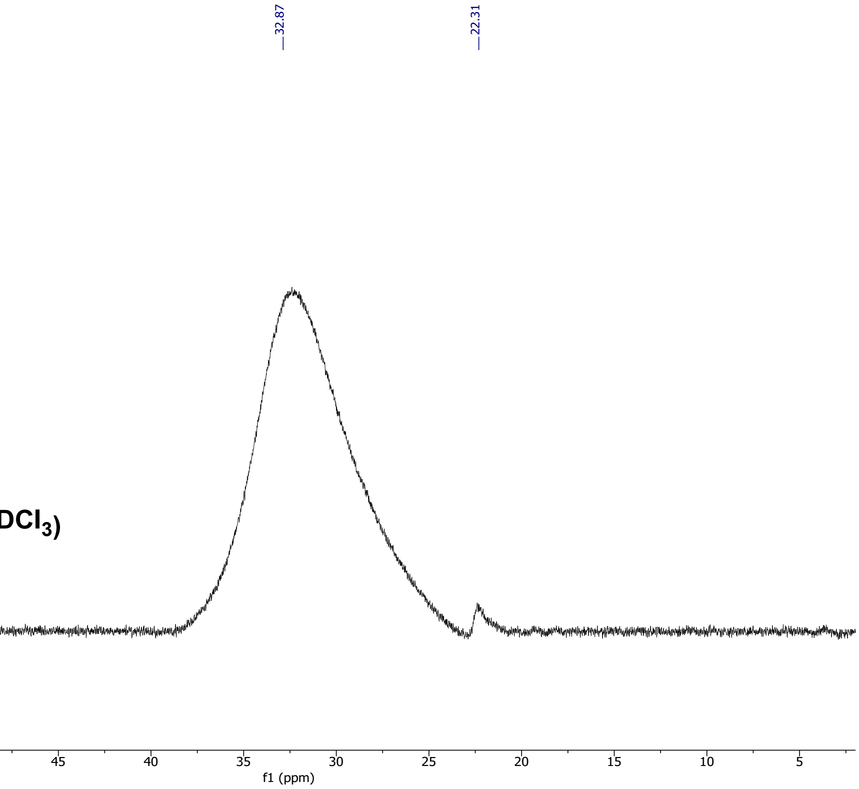

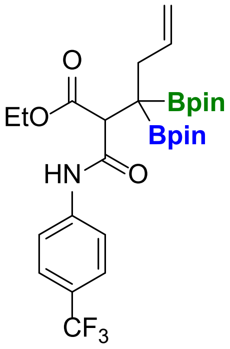

15

^{19}F NMR (377 MHz, CDCl$_3$)

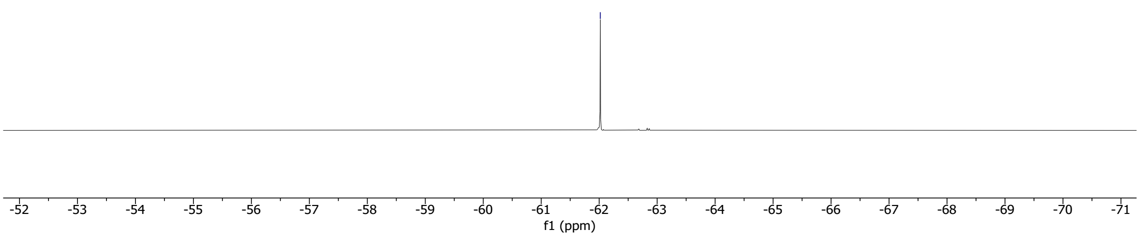

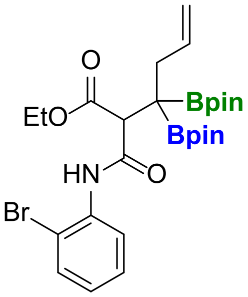

16

^{1}H NMR (400 MHz, CDCl$_3$)

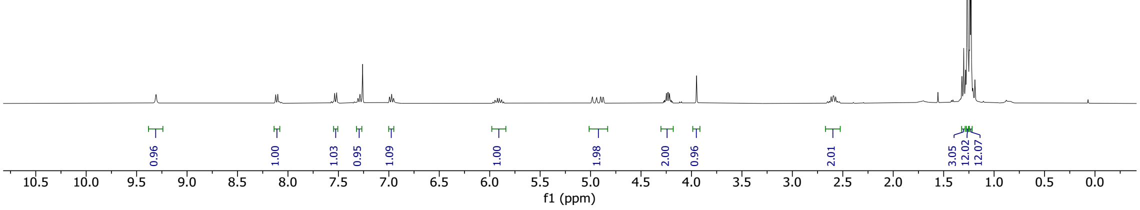

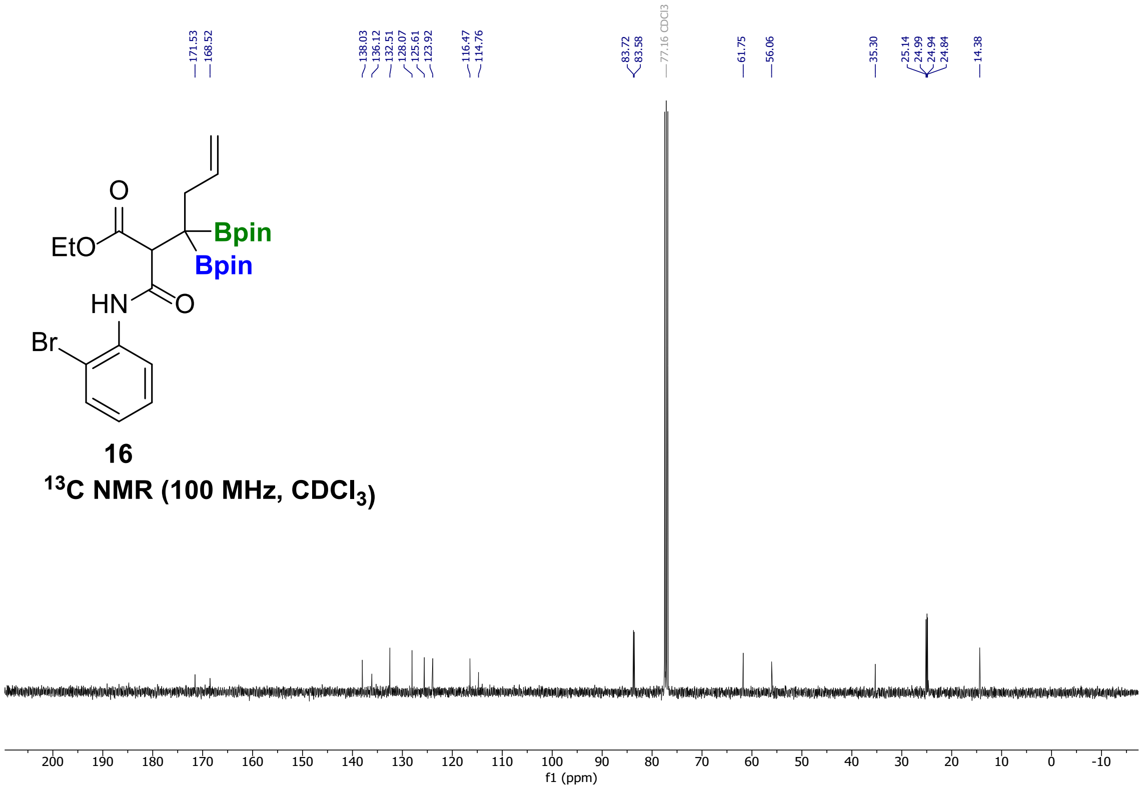

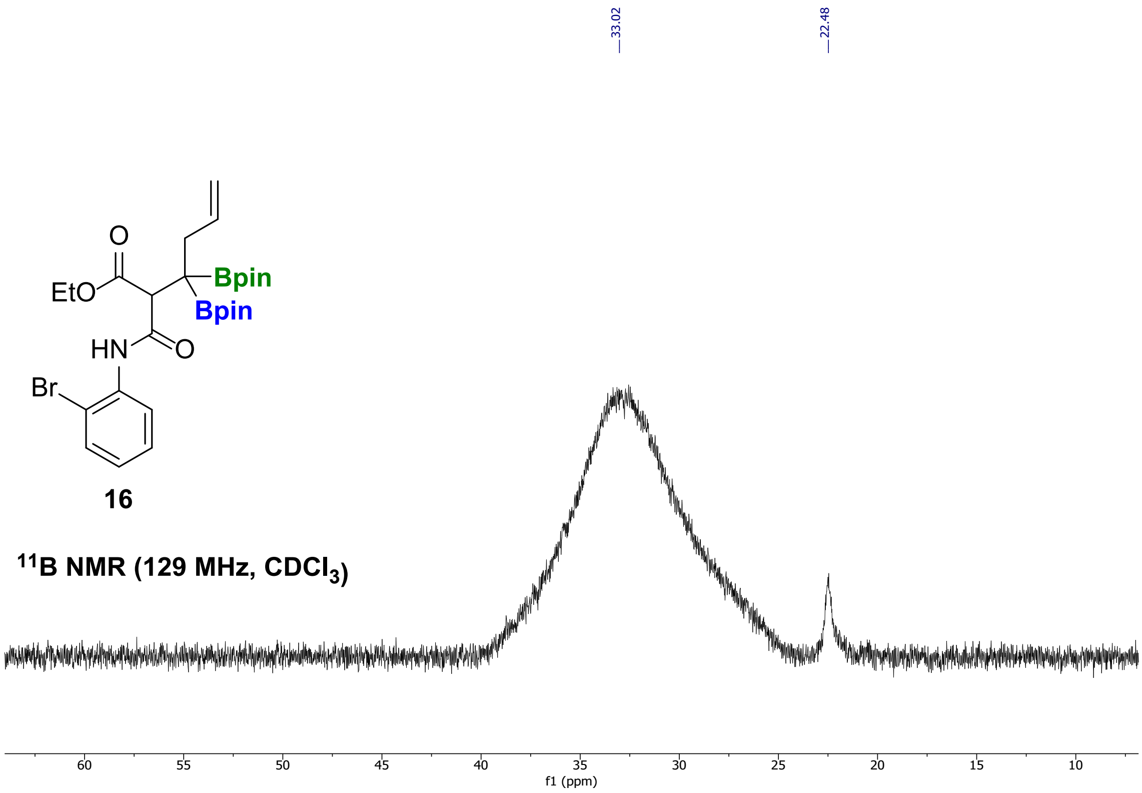

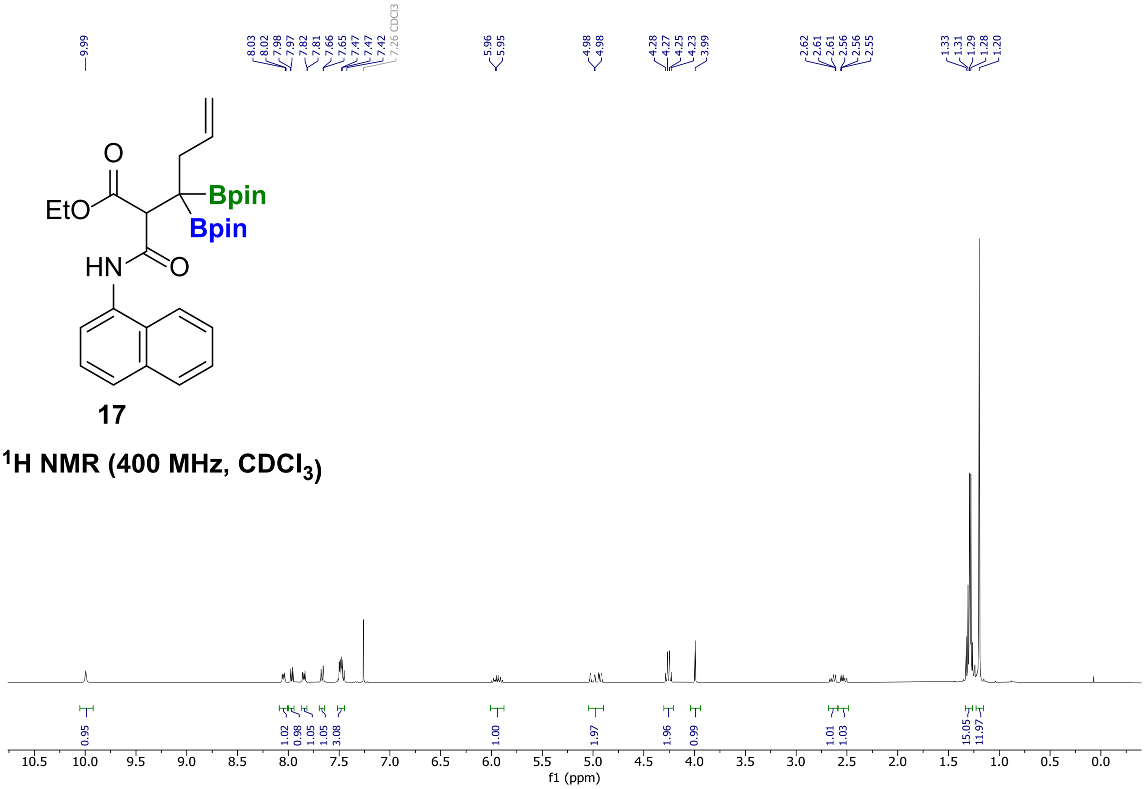

9.99
8.03 8.02 7.98 7.97 7.82 7.81 7.66 7.65 7.47 7.47 7.42 7.26 CDCl3
5.96 5.95
4.98 4.98
4.28 4.27 4.25 4.23 3.99
2.62 2.61 2.61 2.56 2.56 2.55
1.33 1.31 1.29 1.28 1.20
O
EtO
Bpin
Bpin
HN
O
17
^{1}H NMR (400 MHz, $CDCl_3$)
0.95
1.02 0.98 1.05 1.05 3.08
1.00
1.97
1.96 0.99
1.01 1.03
15.05 11.97
f1 (ppm)

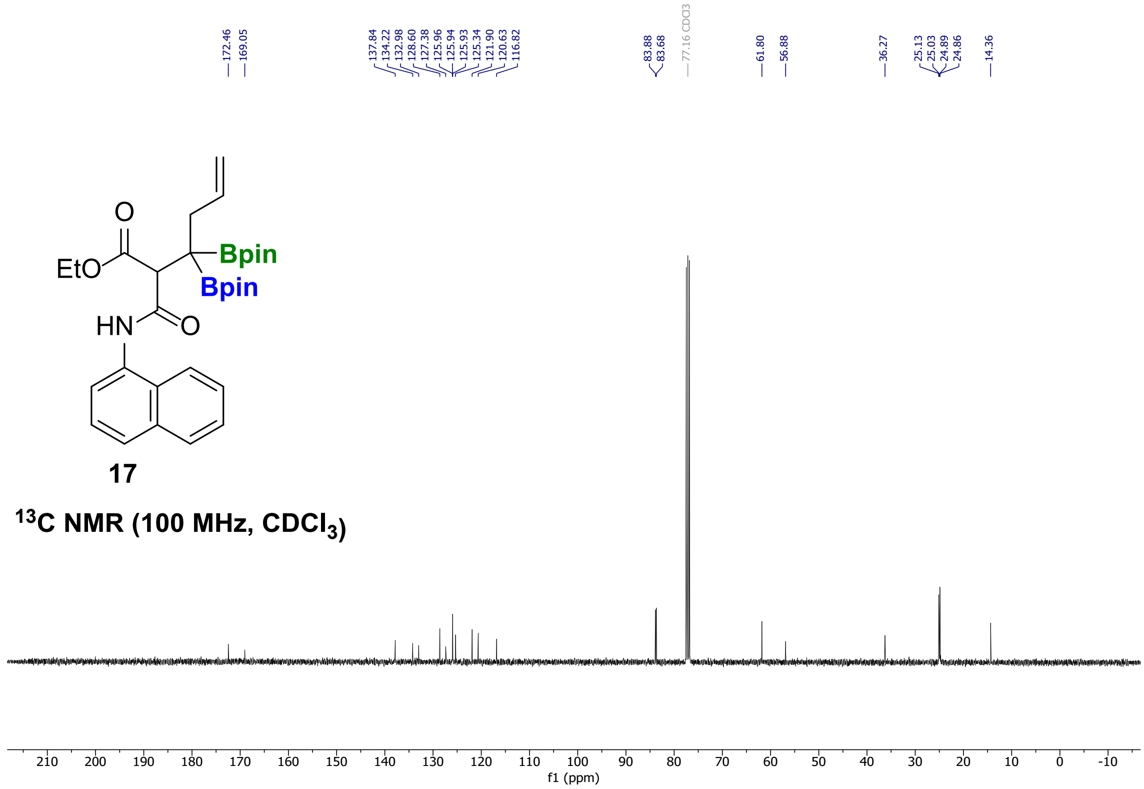

172.46 169.05
137.84 134.22 132.98 128.60 127.38 125.96 125.94 125.93 125.34 121.90 120.63 116.82
83.88 83.68 77.16 CDCl3
61.80 56.88
36.27
25.13 25.03 24.89 24.86
14.36
O
EtO
Bpin
Bpin
HN
O
17
^{13}C NMR (100 MHz, $CDCl_3$)
f1 (ppm)

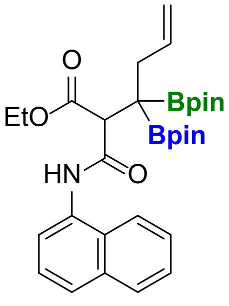

17

^{11}B NMR (129 MHz, $CDCl_3$)

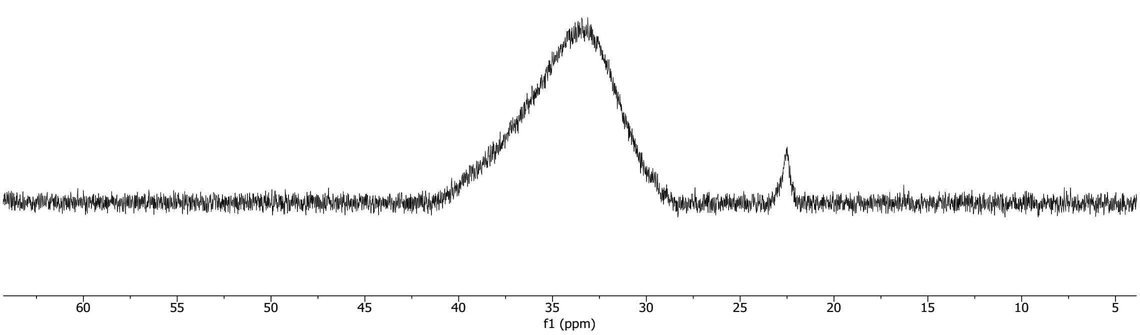

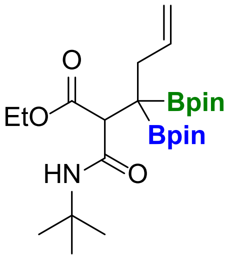

18

^{1}H NMR (400 MHz, $CDCl_3$)

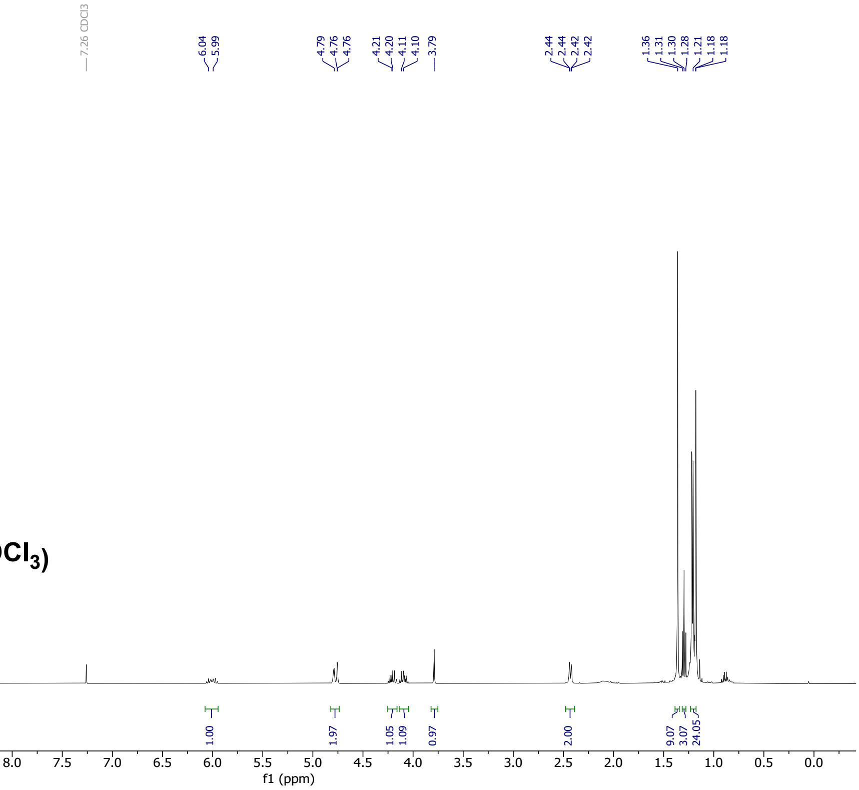

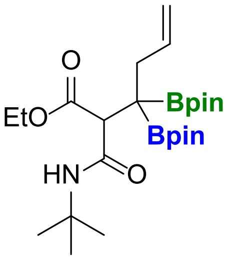

18

^{13}C NMR (100 MHz, CDCl$_3$)

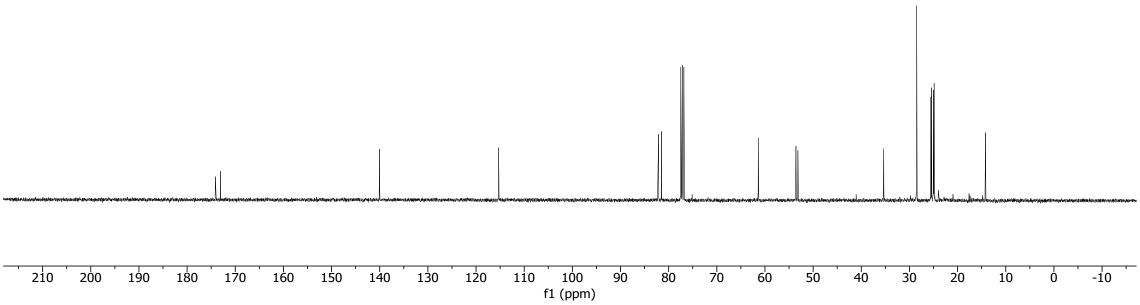

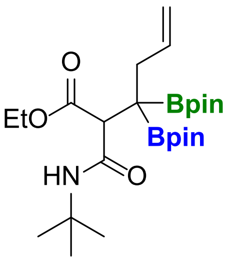

18

^{11}B NMR (129 MHz, CDCl$_3$)

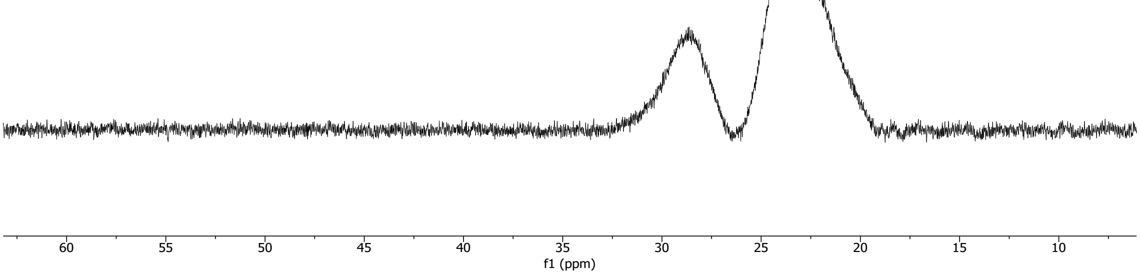

19

^{1}H NMR (400 MHz, CDCl$_3$)

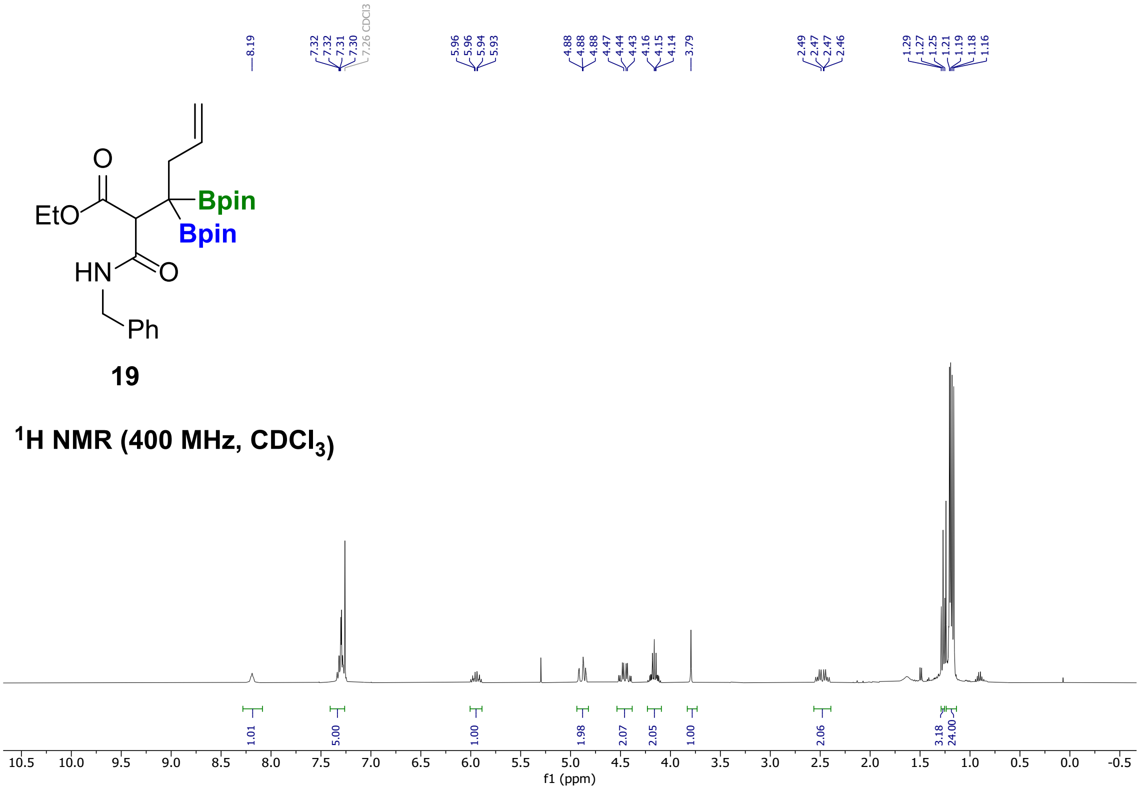

19

^{13}C NMR (100 MHz, CDCl$_3$)

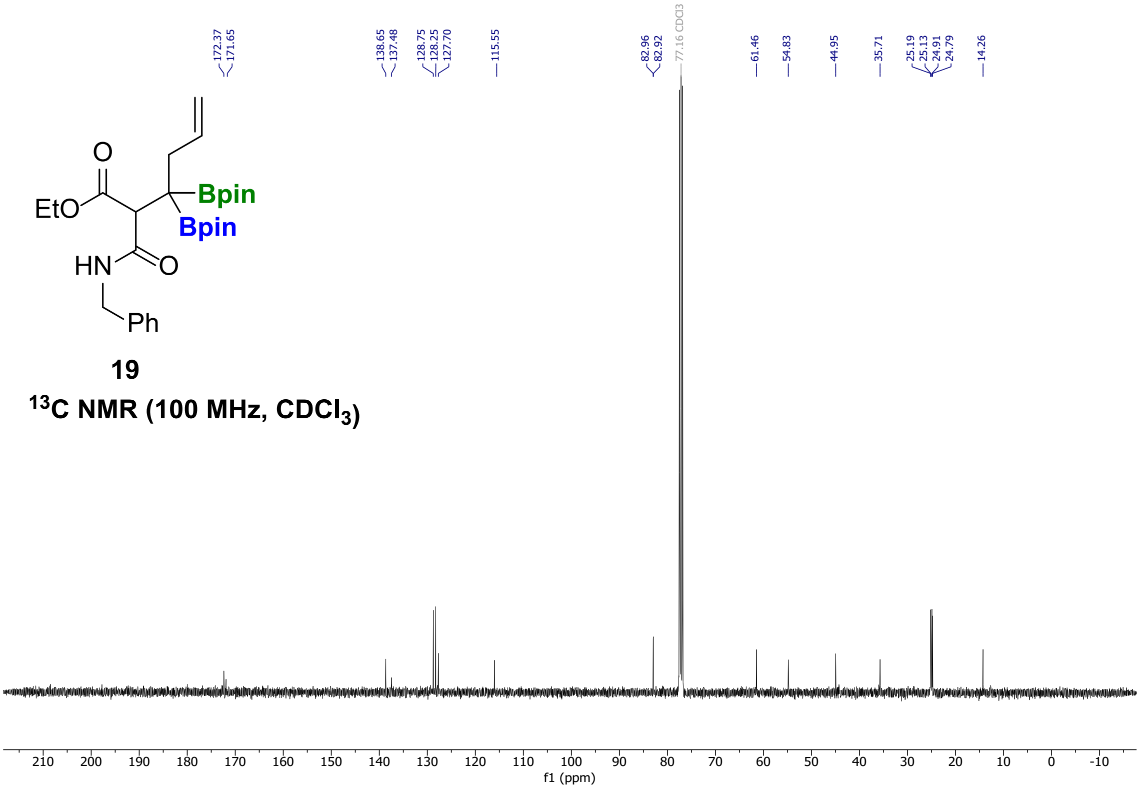

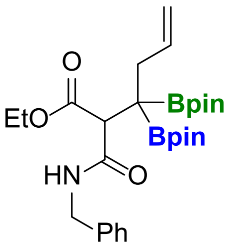

19

^{11}B NMR (129 MHz, $CDCl_3$)

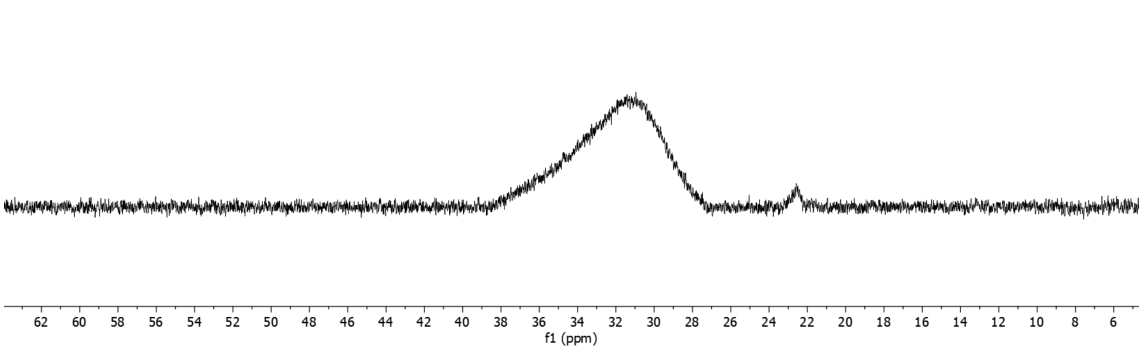

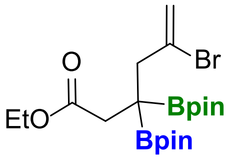

23

^{1}H NMR (400 MHz, $CDCl_3$)

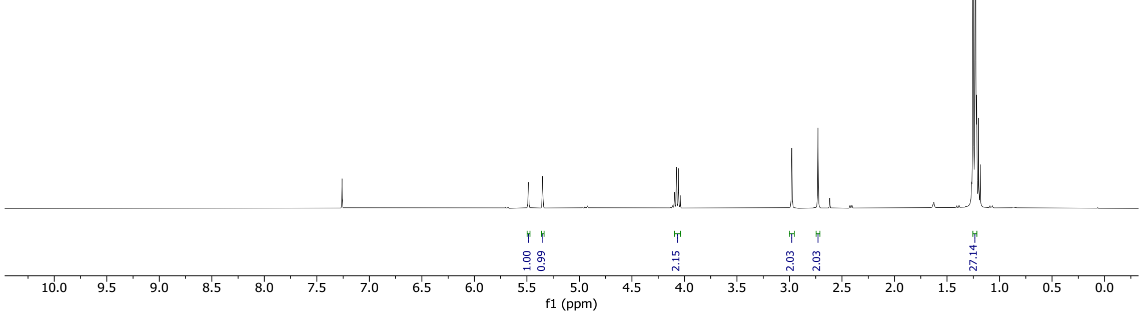

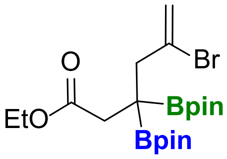

23

^{13}C NMR (100 MHz, CDCl$_3$)

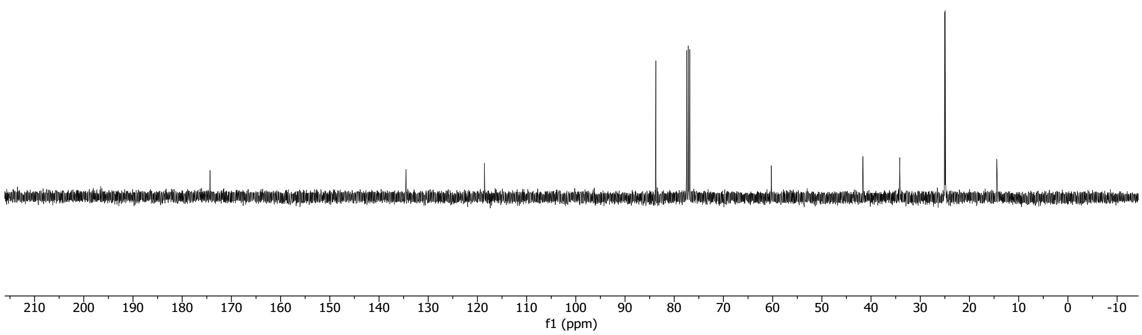

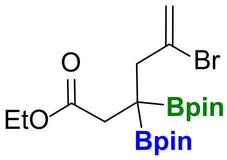

23

^{11}B NMR (129 MHz, CDCl$_3$)

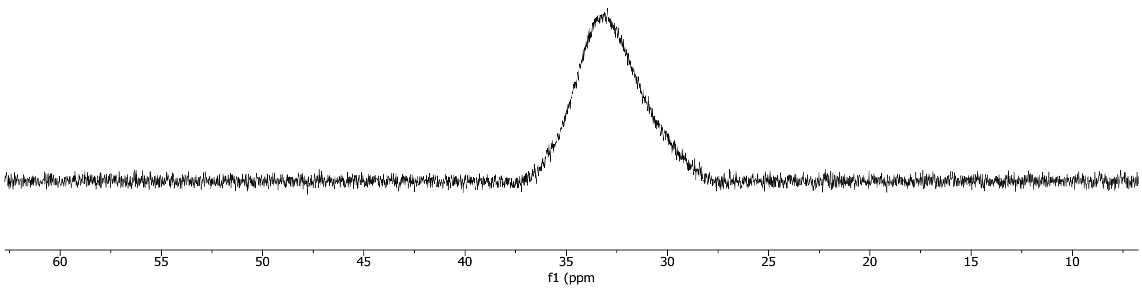

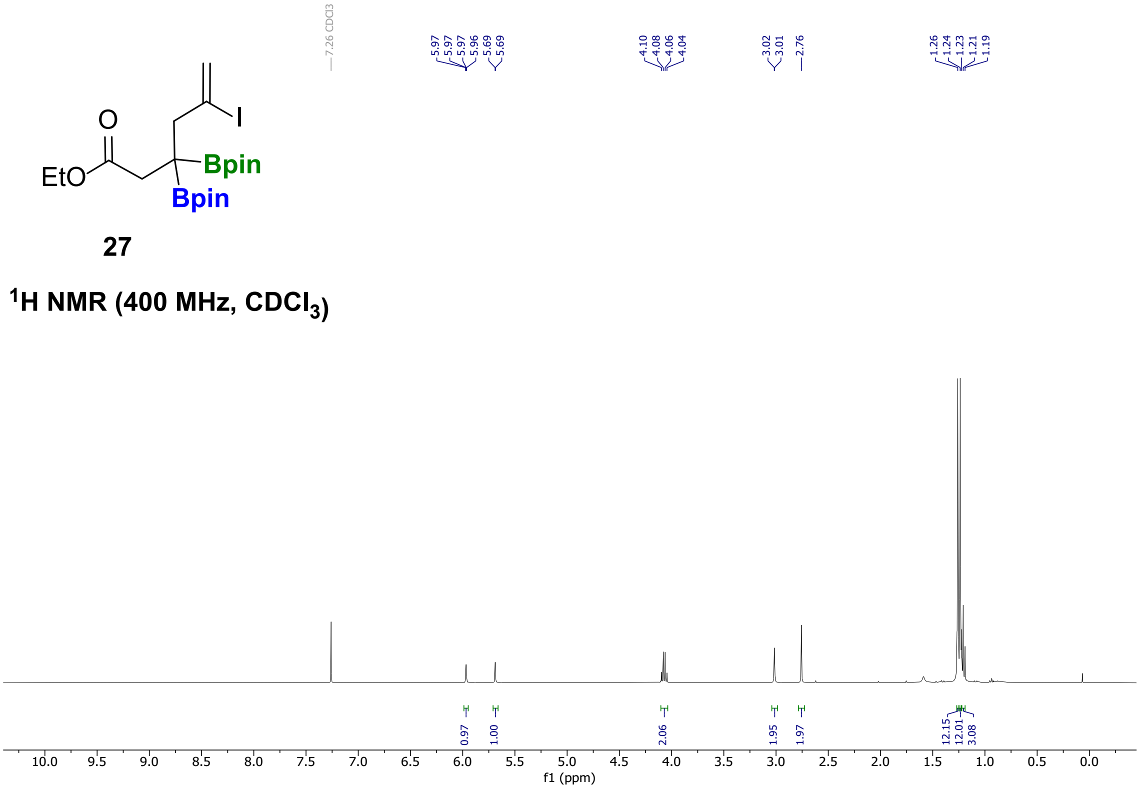

27

^{1}H NMR (400 MHz, $CDCl_3$)

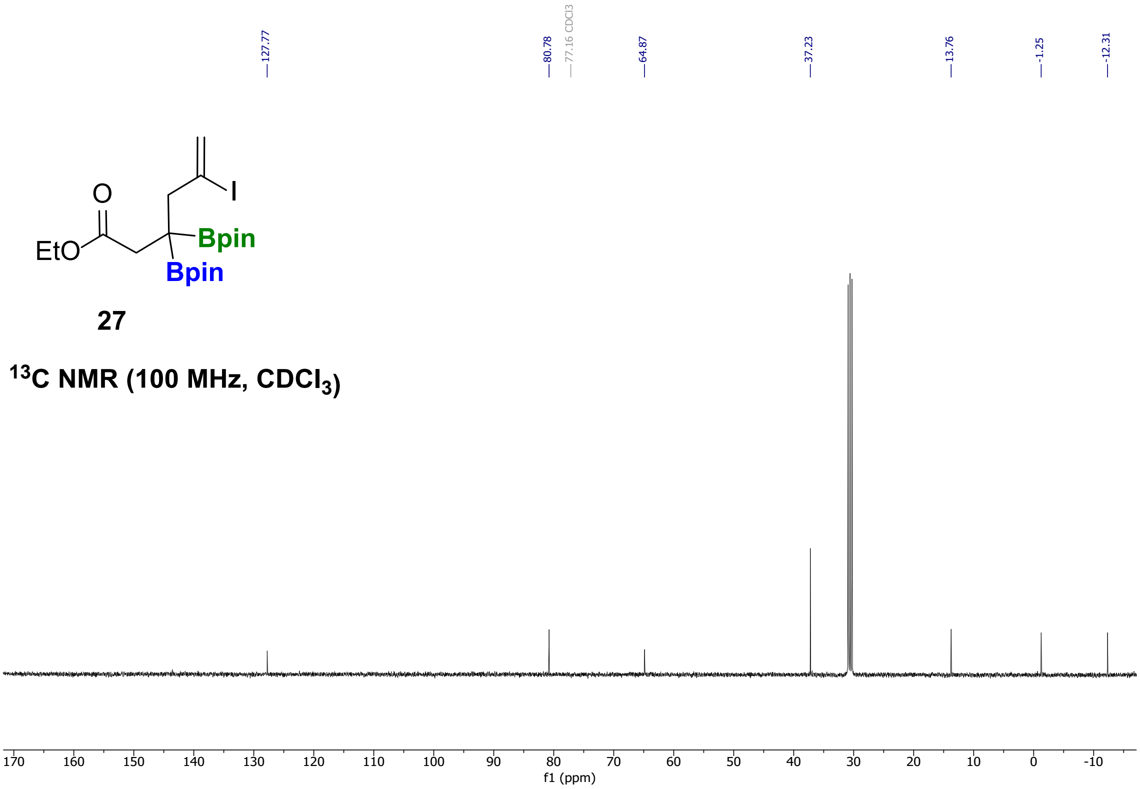

27

^{13}C NMR (100 MHz, $CDCl_3$)

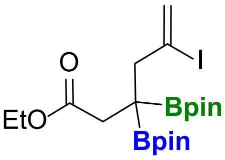

27

^{11}B NMR (129 MHz, CDCl$_3$)

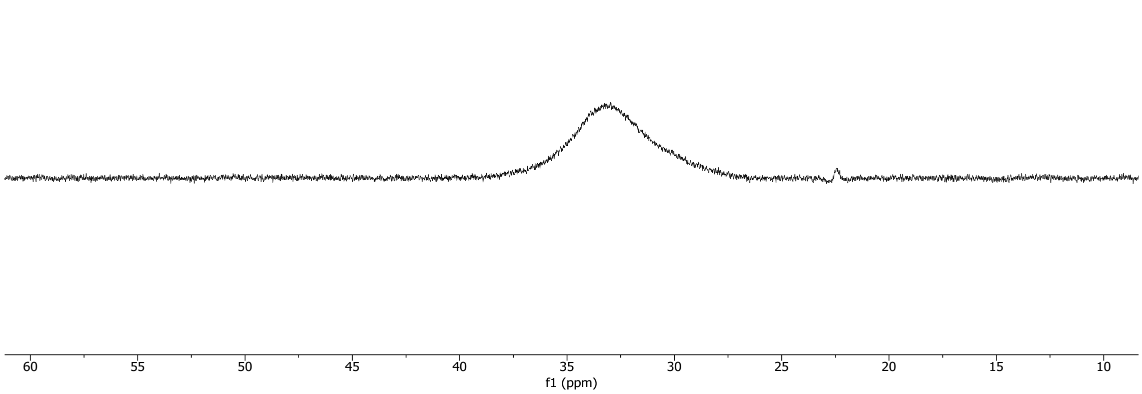

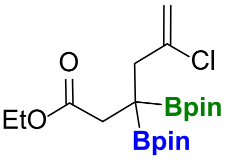

28

^{1}H NMR (400 MHz, CDCl$_3$)

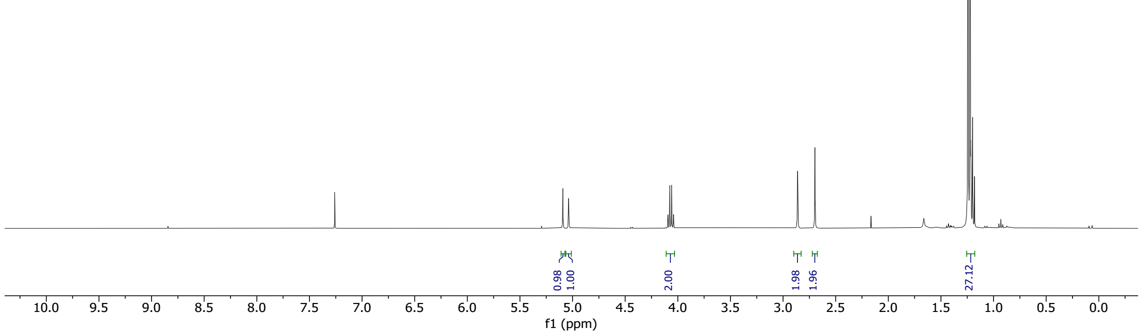

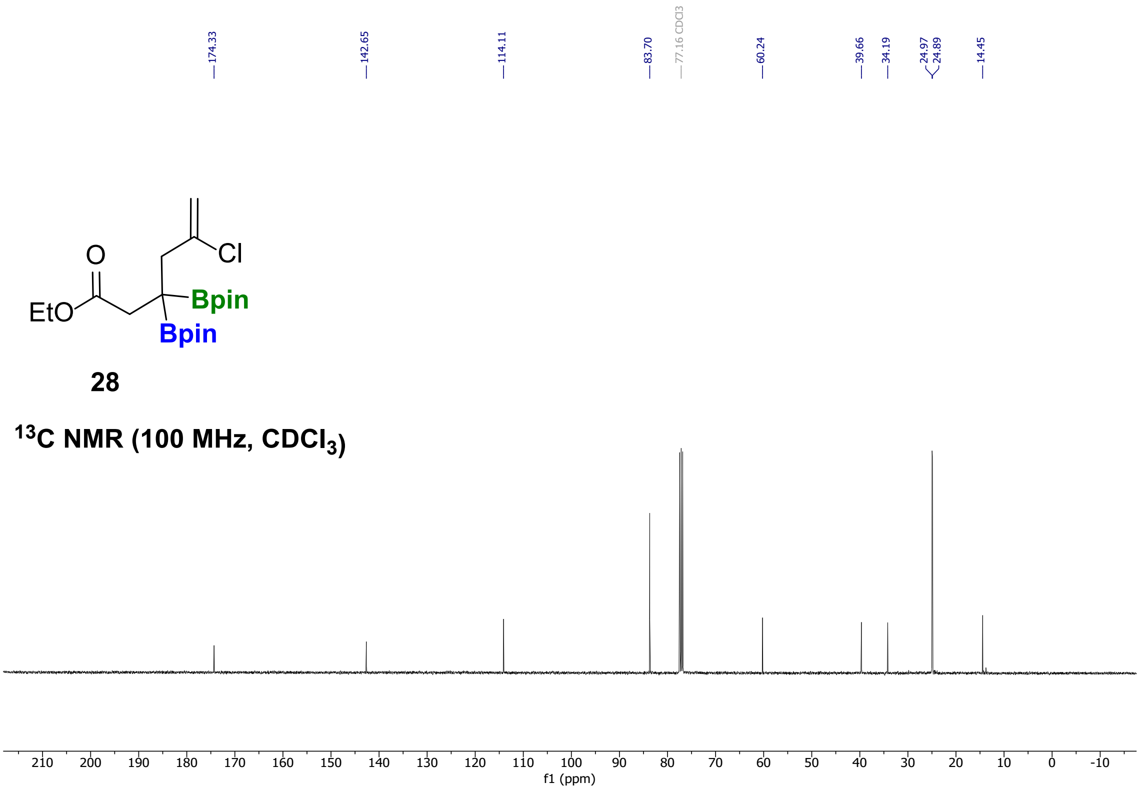
174.33
142.65
114.11
83.70
77.16 CDCl3
60.24
39.66
34.19
24.97
24.89
14.45
28
^{13}C NMR (100 MHz, $CDCl_3$)
f1 (ppm)

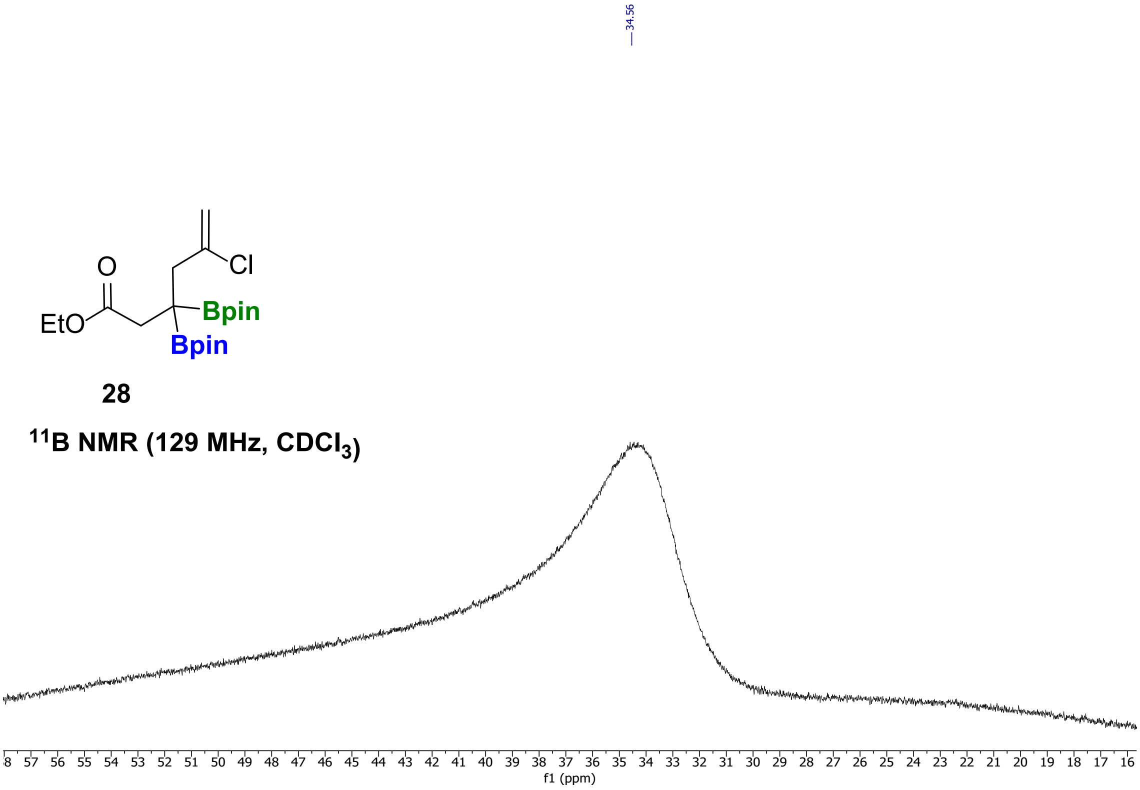
34.56
28
^{11}B NMR (129 MHz, $CDCl_3$)
f1 (ppm)

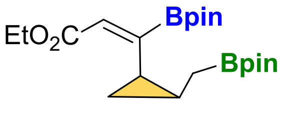

21

^{1}H NMR (400 MHz, CDCl$_3$)

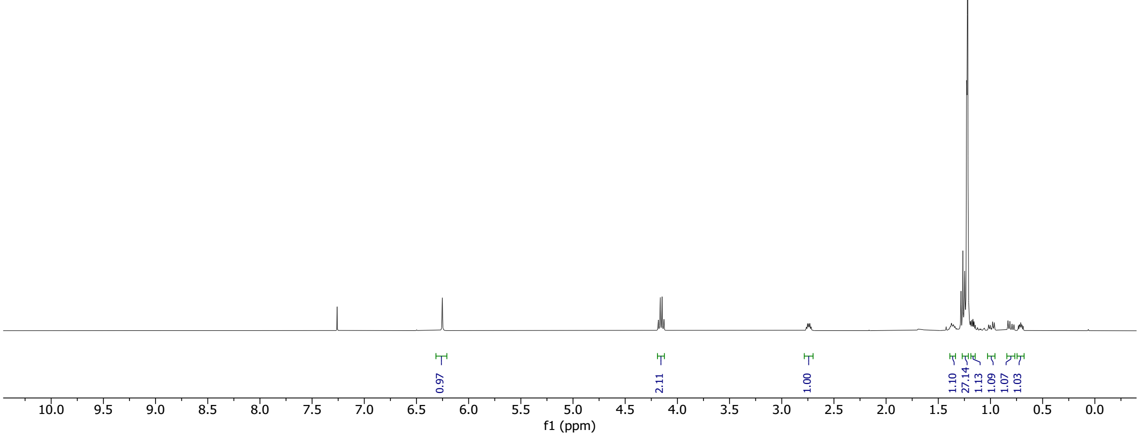

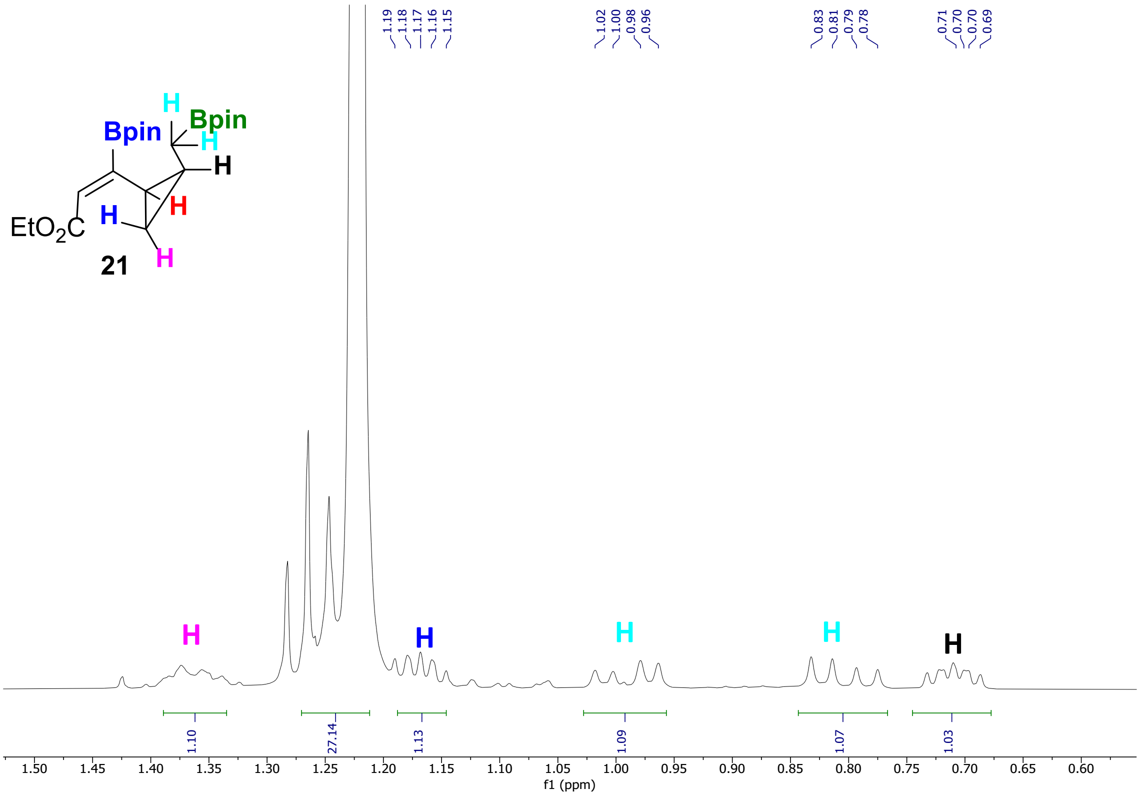

1-D NMR NOE EXPERIMENT

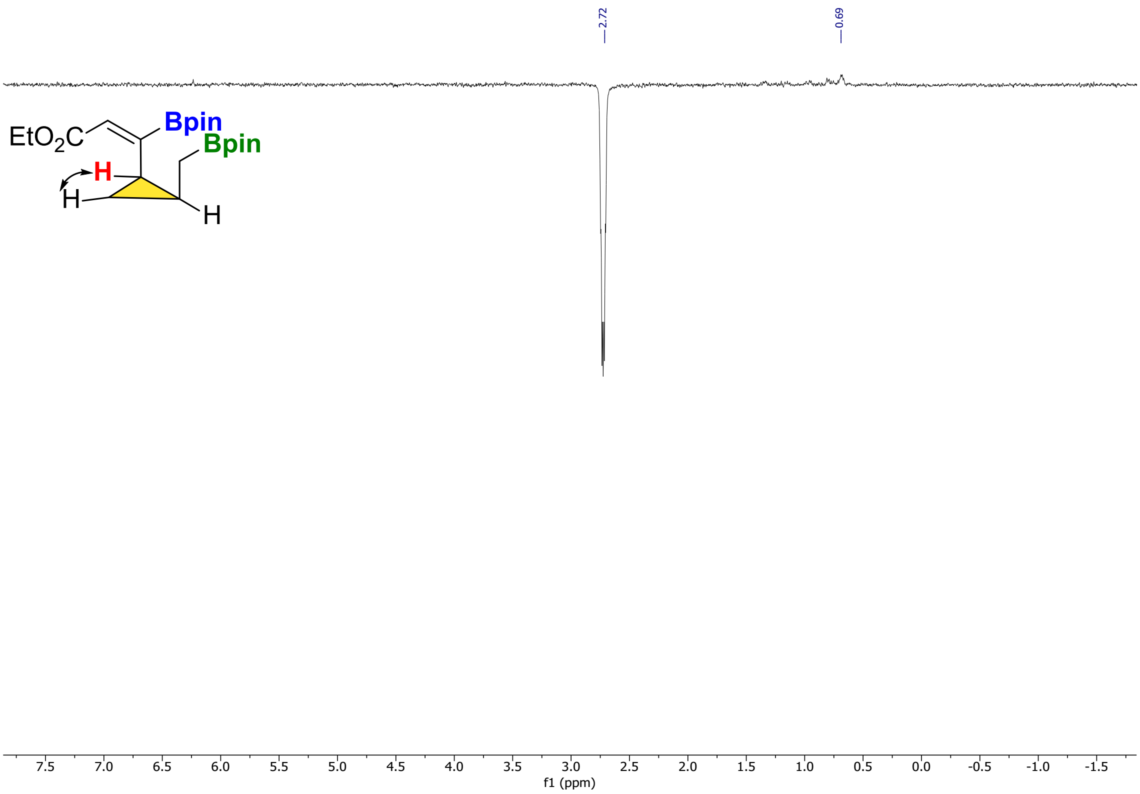

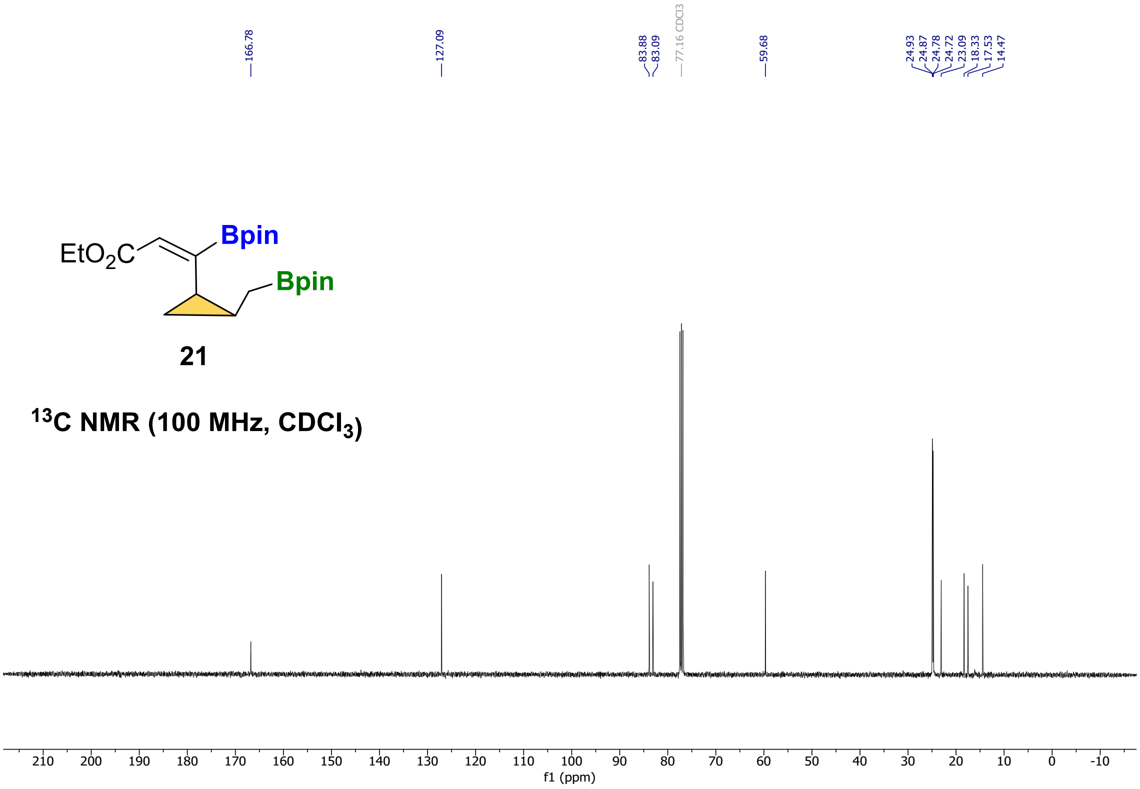

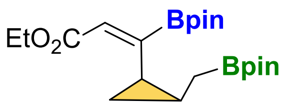

21

^{11}B NMR (129 MHz, $CDCl_3$)

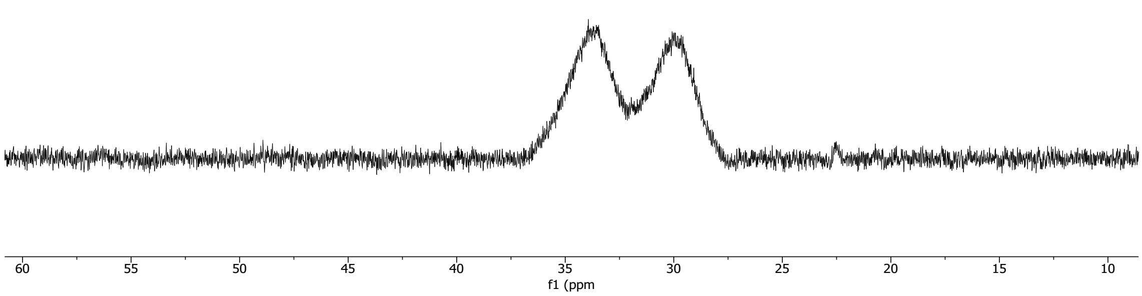

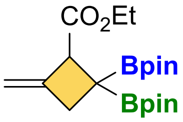

24

^{1}H NMR (400 MHz, $CDCl_3$)

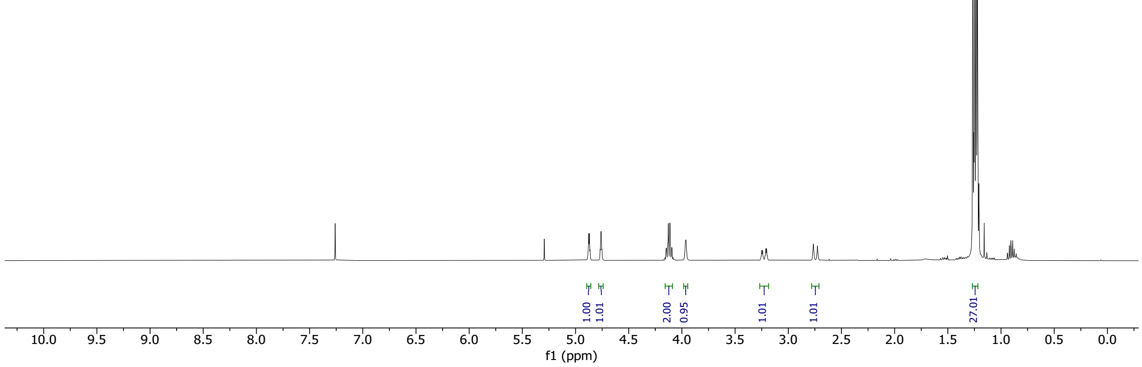

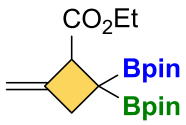

24

^{13}C NMR (100 MHz, CDCl$_3$)

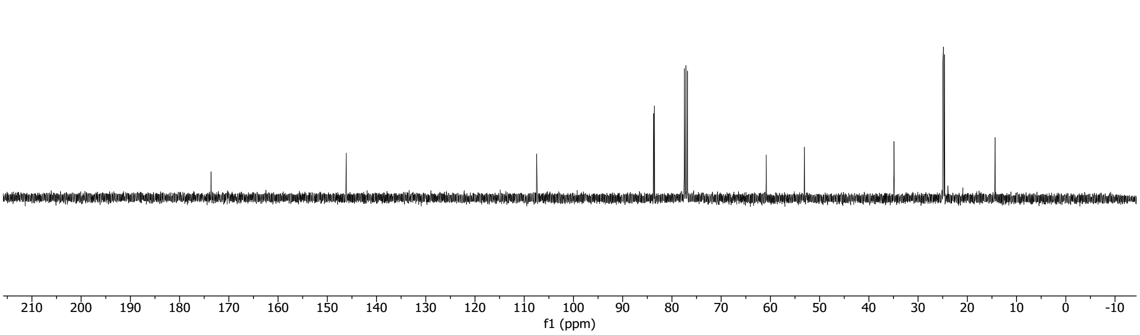

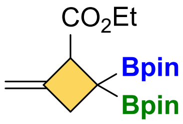

24

^{11}B NMR (129 MHz, CDCl$_3$)

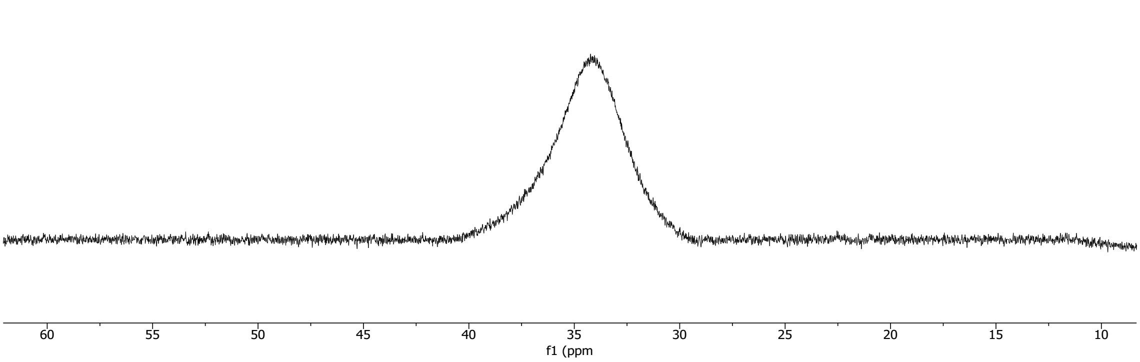

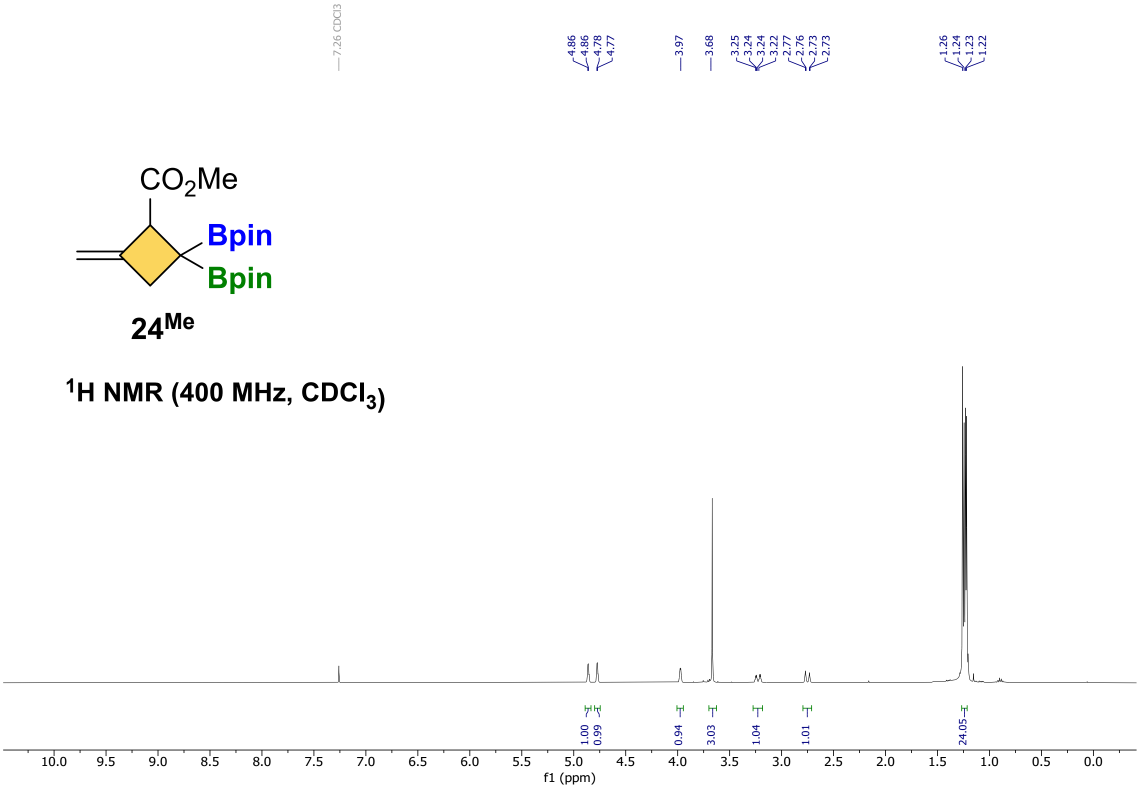

7.26 CDCl3
4.86
4.86
4.78
4.77
3.97
3.68
3.25
3.24
3.24
3.22
2.77
2.76
2.73
2.73
1.26
1.24
1.23
1.22
CO2Me
Bpin
Bpin
24Me
1H NMR (400 MHz, CDCl3)
1.00
0.99
0.94
3.03
1.04
1.01
24.05
f1 (ppm)

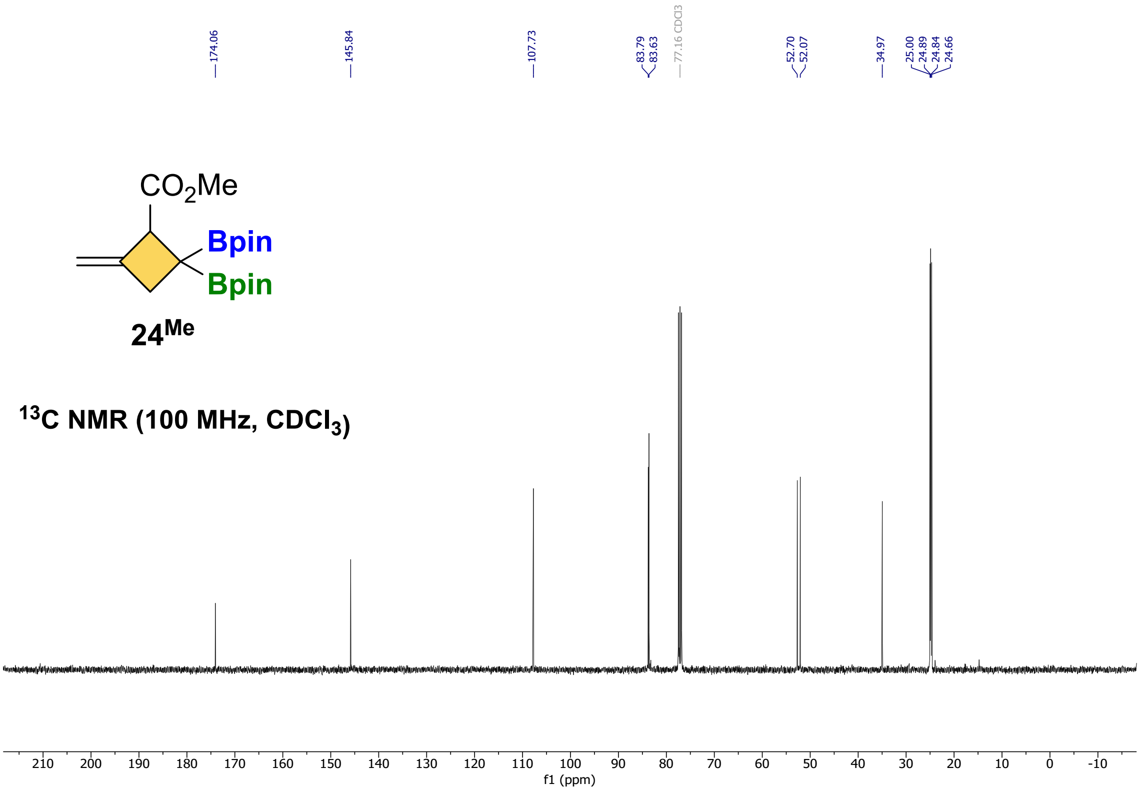

174.06
145.84
107.73
83.79
83.63
77.16 CDCl3
52.70
52.07
34.97
25.00
24.89
24.84
24.66
CO2Me
Bpin
Bpin
24Me
13C NMR (100 MHz, CDCl3)
f1 (ppm)

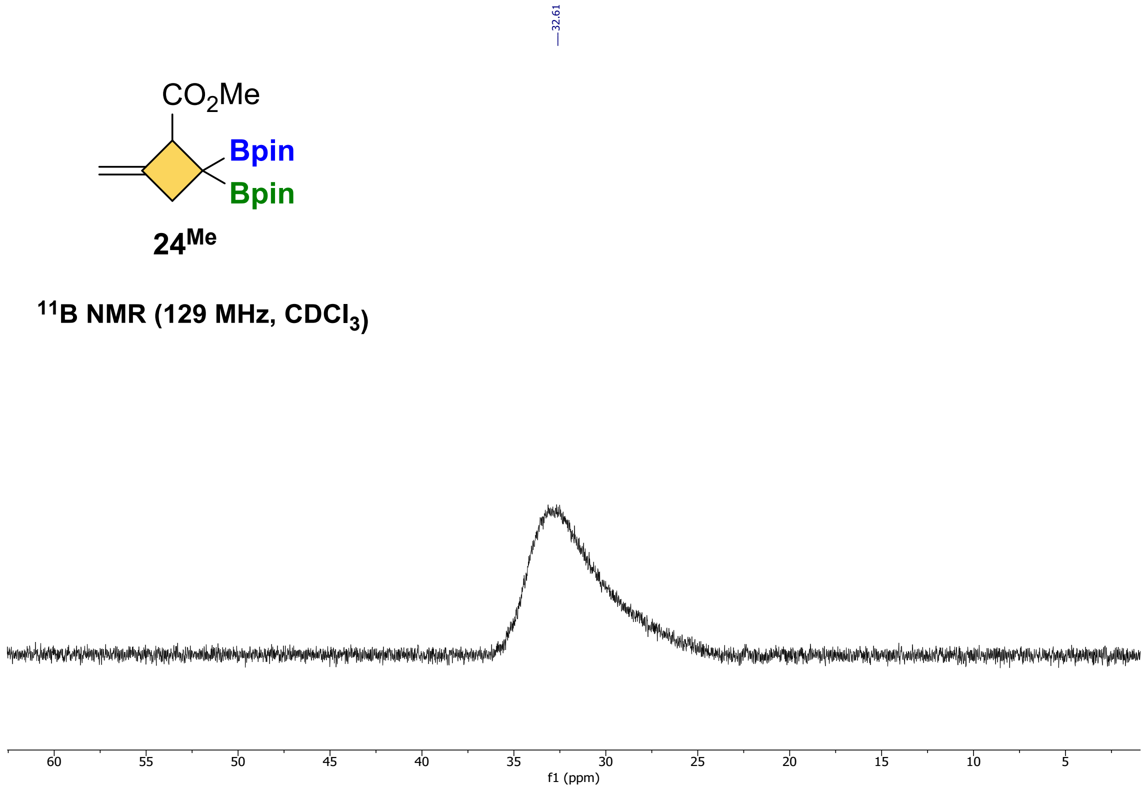

CO2Me
Bpin
Bpin
24Me
11B NMR (129 MHz, CDCl3)
32.61
f1 (ppm)

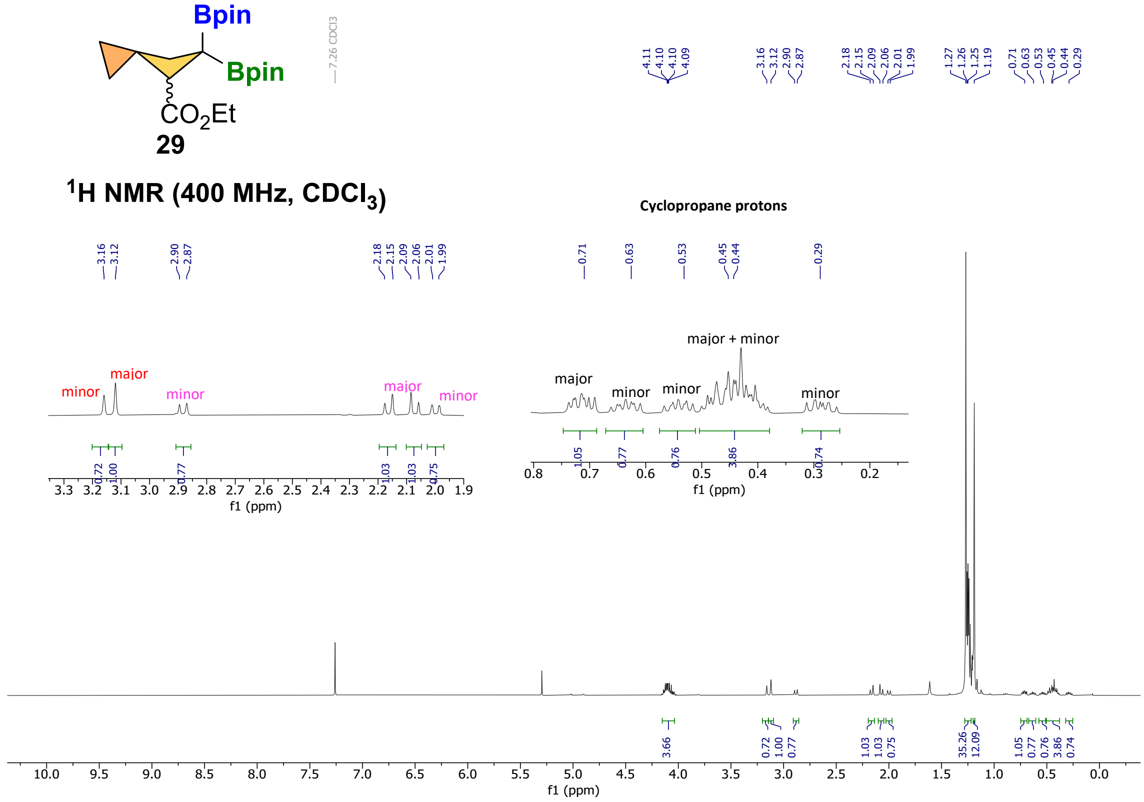

Bpin
Bpin
CO2Et
29
1H NMR (400 MHz, CDCl3)
7.26 CDCl3
4.11 4.10 4.10 4.09
3.16 3.12 2.90 2.87
2.18 2.15 2.09 2.06 2.01 1.99
1.27 1.26 1.25 1.19
0.71 0.63 0.53 0.45 0.44 0.29
Cyclopropane protons
minor
major
minor
major
minor
major
minor
minor
major + minor
minor
f1 (ppm)

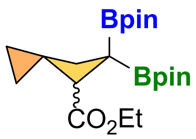

29

^{13}C NMR (100 MHz, $CDCl_3$)

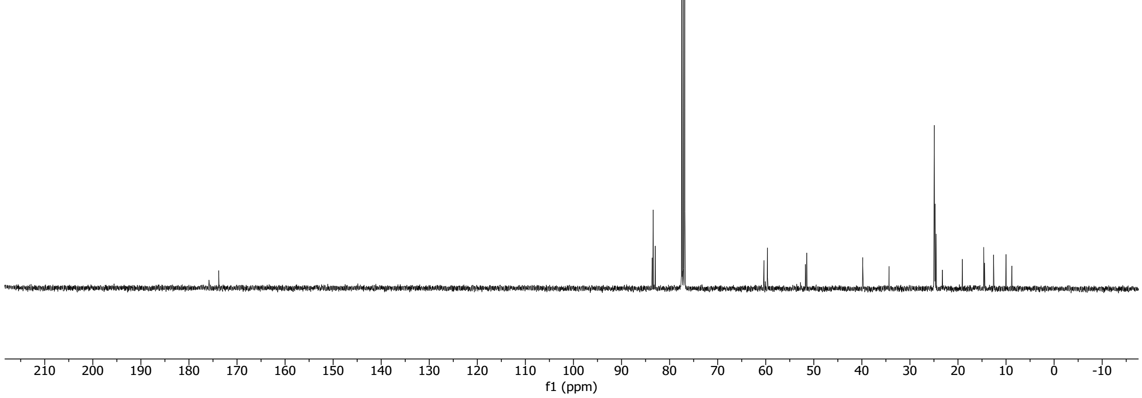

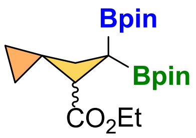

29

^{11}B NMR (129 MHz, $CDCl_3$)

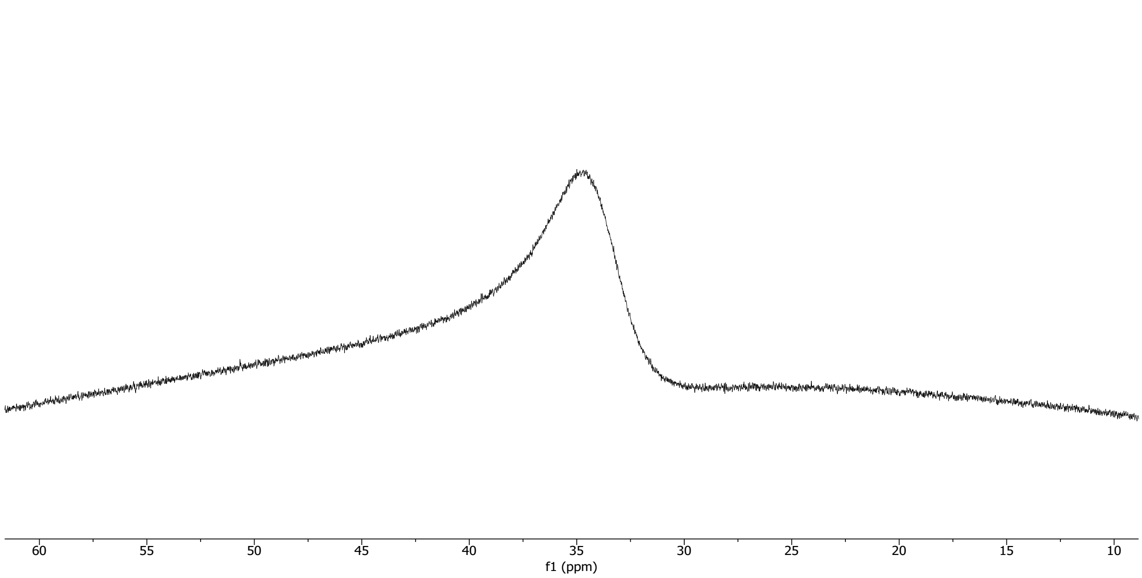

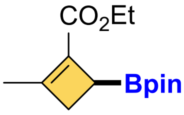

30

^{1}H NMR (400 MHz, $CDCl_3$)

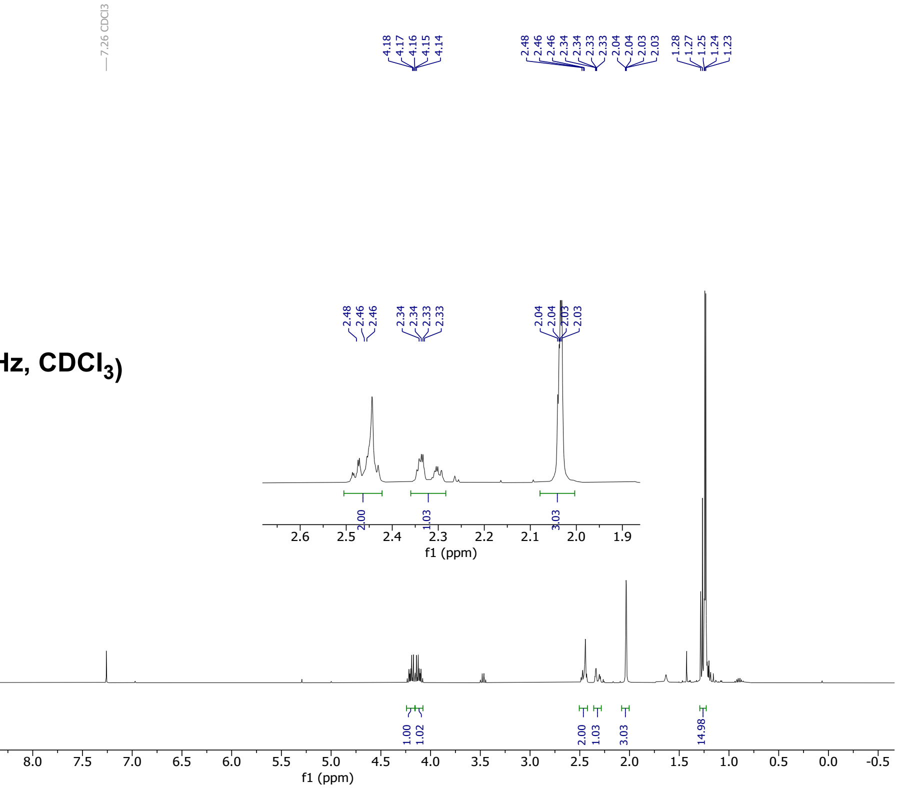

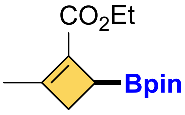

30

^{13}C NMR (100 MHz, $CDCl_3$)

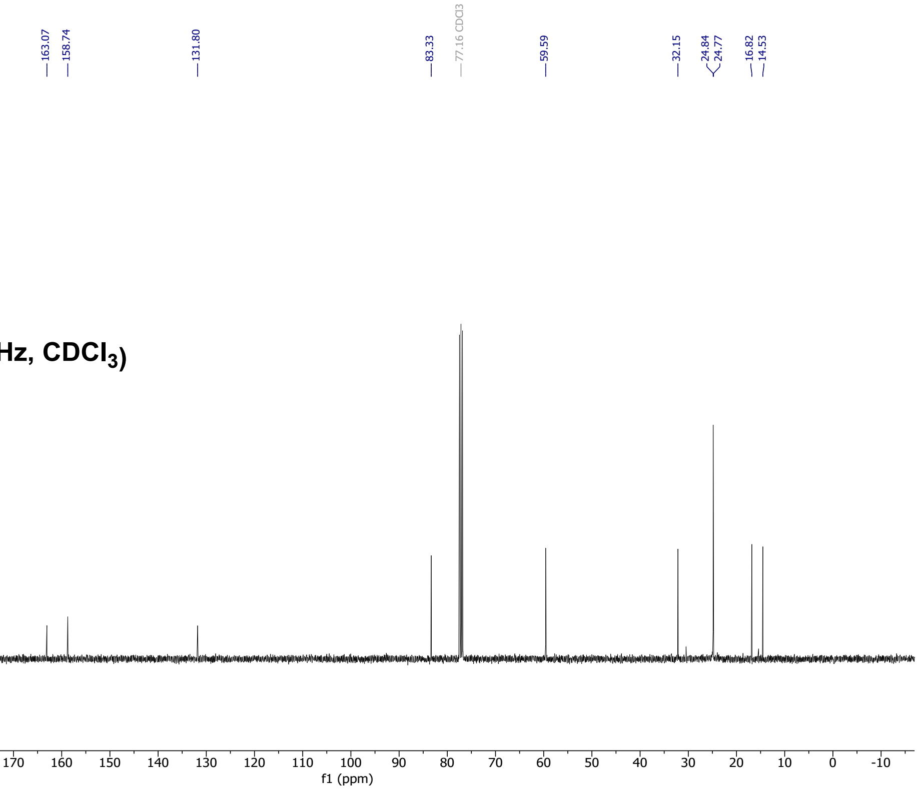

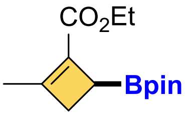

30

^{11}B NMR (129 MHz, CDCl$_3$)

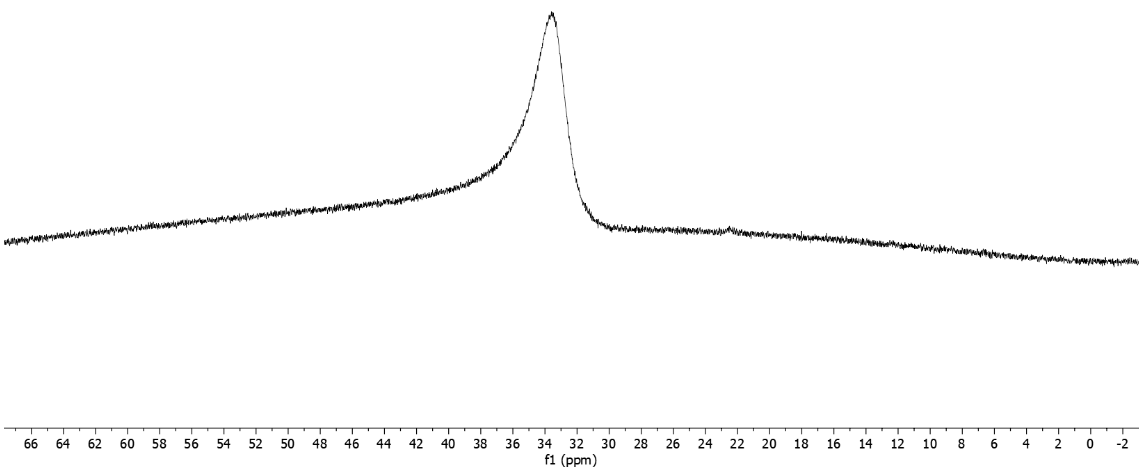

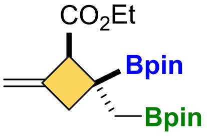

31

^{1}H NMR (400 MHz, CDCl$_3$)

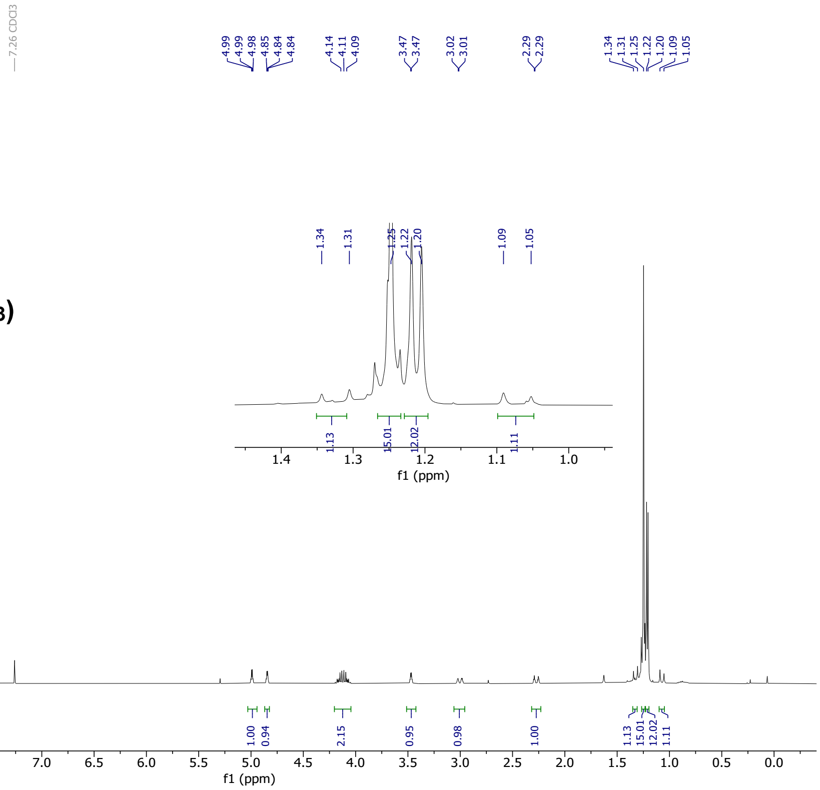

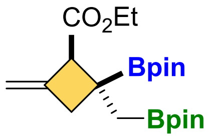

31

^{13}C NMR (100 MHz, $CDCl_3$)

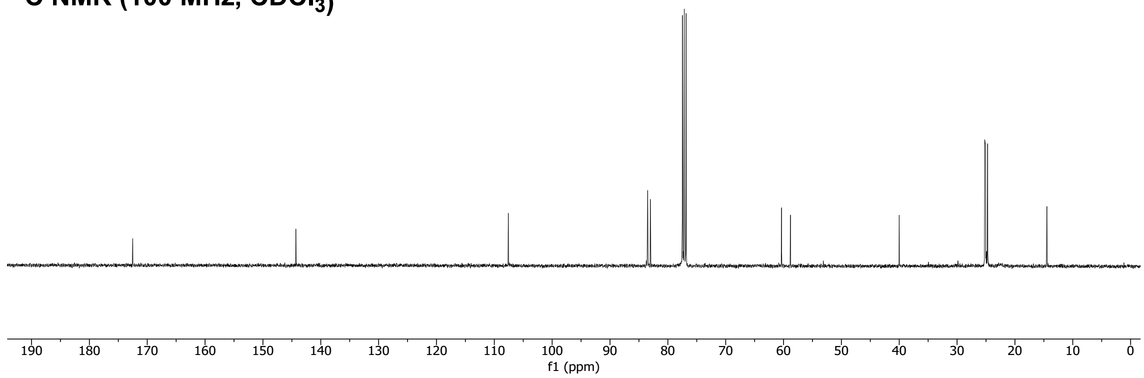

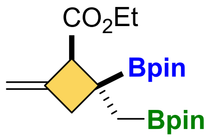

31

^{11}B NMR (129 MHz, $CDCl_3$)

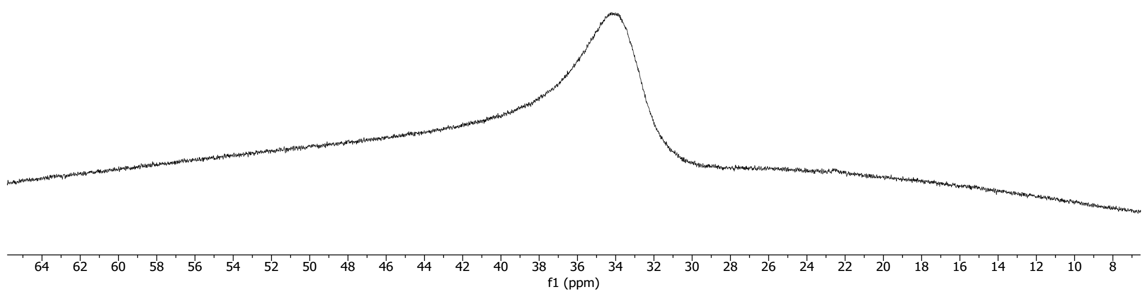

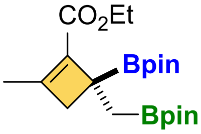

32

^{1}H NMR (400 MHz, $CDCl_3$)

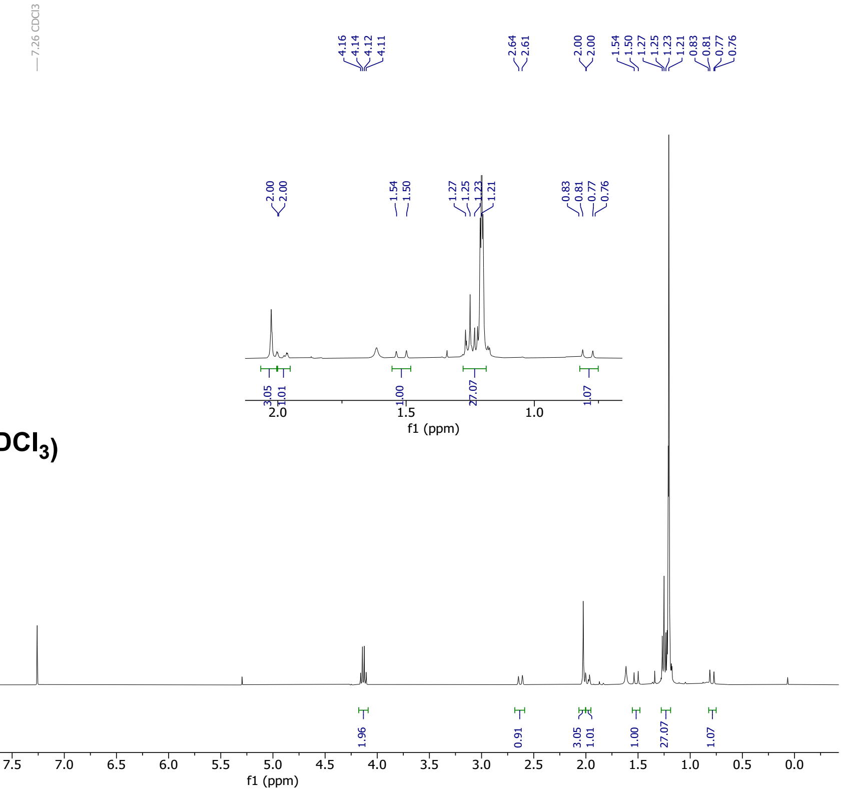

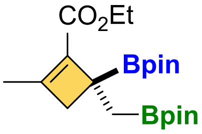

32

^{13}C NMR (100 MHz, $CDCl_3$)

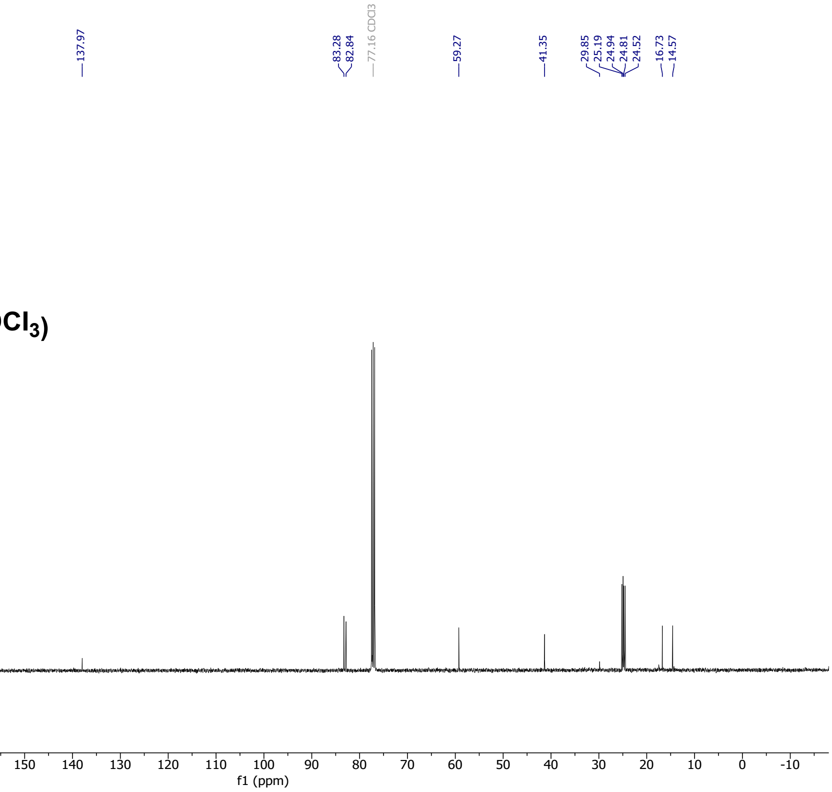

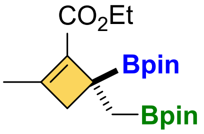

32

^{11}B NMR (129 MHz, CDCl$_3$)

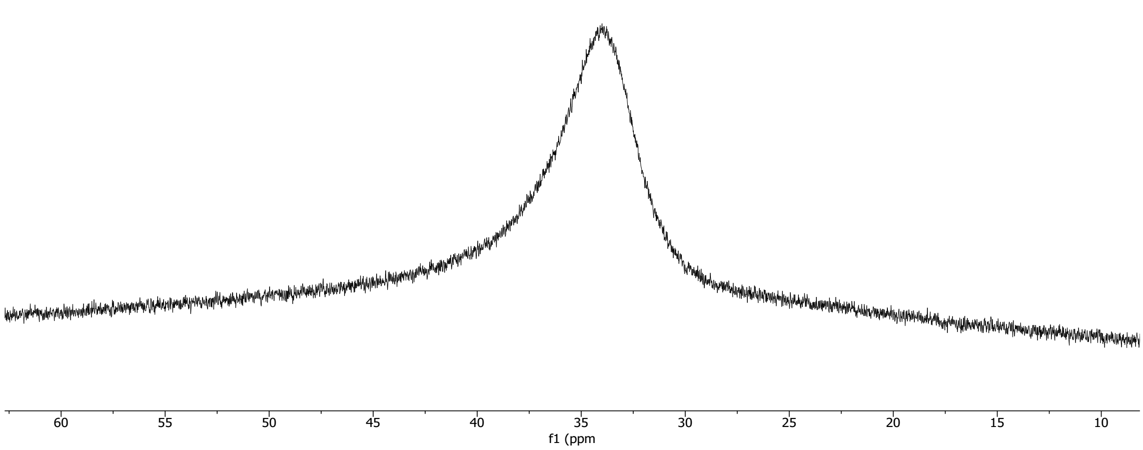

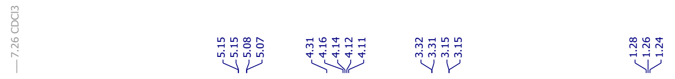

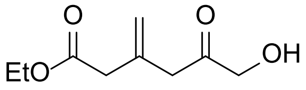

33

^{1}H NMR (400 MHz, CDCl$_3$)

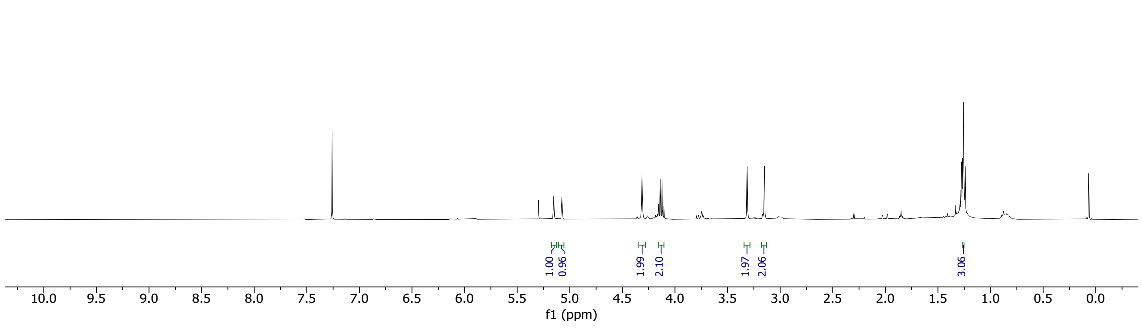

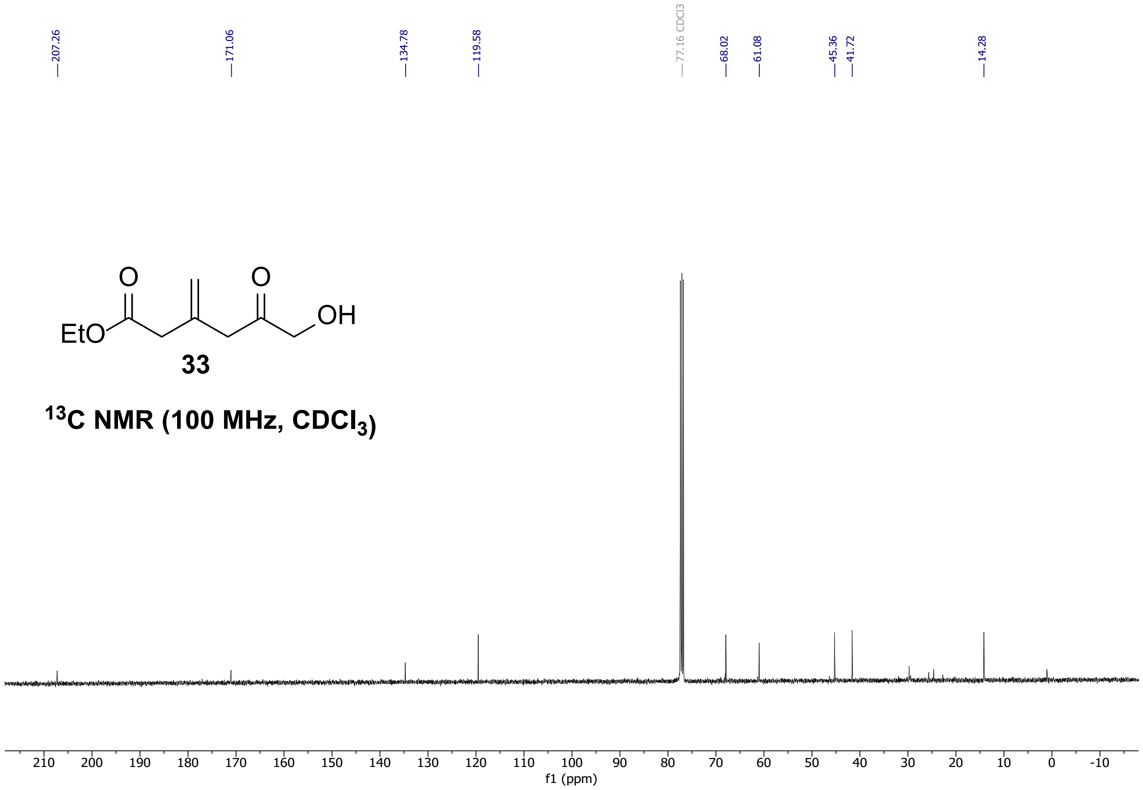
207.26
171.06
134.78
119.58
77.16 CDCl3
68.02
61.08
45.36
41.72
14.28
O
O
EtO
OH
33
^{13}C NMR (100 MHz, $CDCl_3$)
210 200 190 180 170 160 150 140 130 120 110 100 90 80 70 60 50 40 30 20 10 0 -10
f1 (ppm)

Complementary Reactivity

Suzuki reaction of product 2

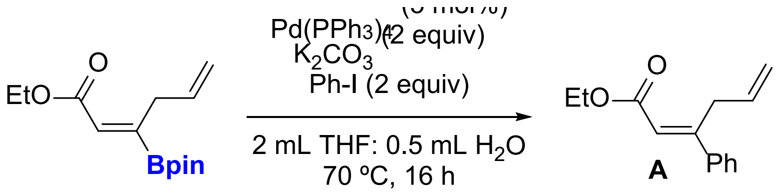

In a flamed Schlenk-tube equipped with a magnetic stir bar, $Pd(PPh_3)_4$ (0.20 mmol, 5 mol%), skipped (*Z*)-dienoate (0.4 mmol, 1 equiv), PhI (0.8 mmol, 2 equiv) and K_2CO_3 (0.8 mmol, 2 equiv) were added in THF (2 mL) and water (0.5 mL) under argon atmosphere. The reaction mixture was stirred at 70 °C in an oil bath for 16 h. After that, the reaction was concentrated under vacuum and the crude was purified by flash chromatography to obtain the product **A** with a 55% isolated yield (47 mg).

Ethyl (*E*)-3-phenylhexa-2,5- dienoate (A)

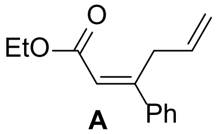

A

The product was purified by flash chromatography using as eluent a mixture of petroleum ether/ethyl acetate (100:1). The product was isolated as a pail yellowish oil (47 mg, 0.217 mmol, 55%).

^{1}H NMR (400 MHz, $CDCl_3$) δ= 7.55 – 7.42 (m, 2H), 7.37 – 7.31 (m, 3H), 6.15 (s, 1H), 5.87 (ddt, *J* = 17.2, 10.1, 6.2 Hz, 1H), 5.10 (dq, *J* = 17.2, 1.7 Hz, 1H), 5.02 (dq, *J* = 10.1, 1.6 Hz, 1H), 4.22 (q, *J* = 7.1 Hz, 2H), 3.92 – 3.85 (m, 2H), 1.32 (t, *J* = 7.1 Hz, 3H).

^{13}C NMR (100 MHz, $CDCl_3$) δ= 166.5, 157.0, 141.3, 135.5, 129.1, 128.6, 126.9, 118.1, 116.4, 60.1, 35.5, 14.5.

^{1}H, ^{13}C NMR spectra of substrate A

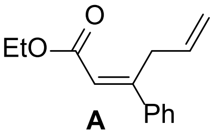

^{1}H NMR (400 MHz, $CDCl_3$)

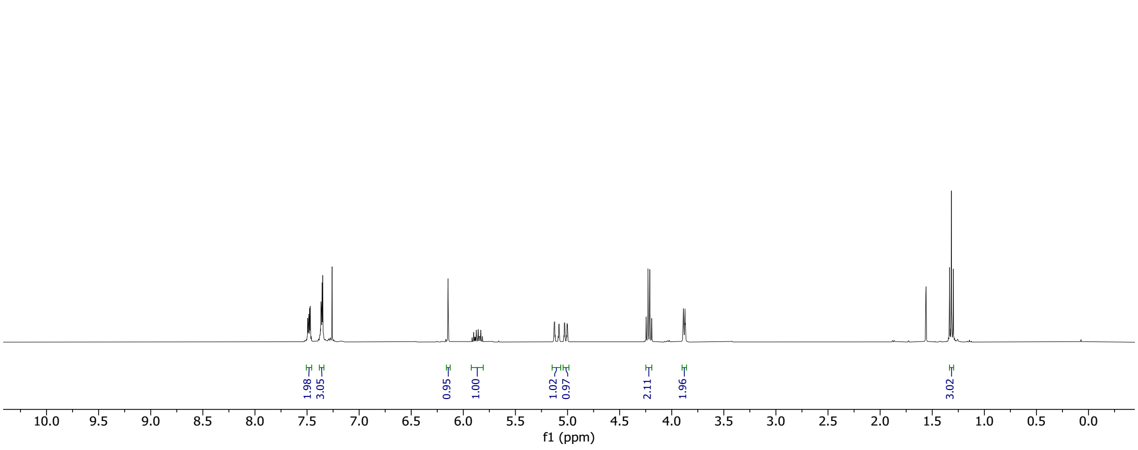

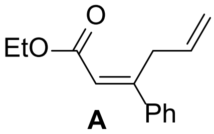

^{13}C NMR (100 MHz, $CDCl_3$)

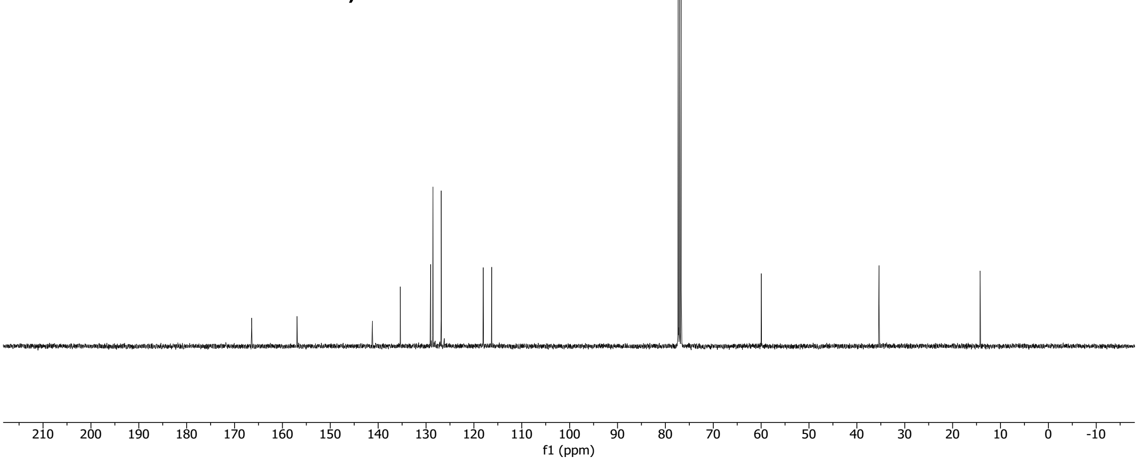

Borylcupration of substrate A

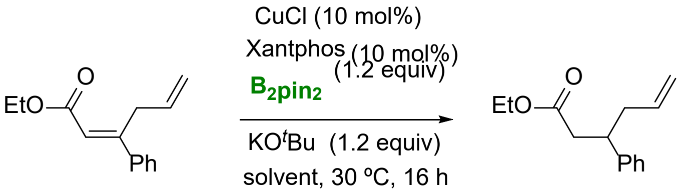

In a flamed Schlenk-tube equipped with a magnetic stir bar CuCl (1.98 mg, 10 mol%, 0.02 mmol), diboron reagent (60.9 mg, 1.2 equiv, 0.24 mmol) and Xantphos (277,6 mg, 10 mol%, 0.02 mmol) were placed. The vial was evacuated and backfilled with nitrogen and THF (1 mL) was added. Next, KO^{t}Bu (26.9 mg, 1.2 equiv, 0.24 mmol) in THF (1 mL) was poured in the vial through the rubber septum. Then, substrate **A** (1 equiv, 0.2 mmol) in THF (1 mL) was added dropwise at 30 °C. After the reaction was completed, (16h) the reaction mixture was filtered over Celite. The solvents were evaporated at the rotatory evaporator and the crude was purified by silica gel chromatography to obtain product B in 12% isolated yield (8 mg).

Ethyl 3-phenylhex-5-enoate (B)

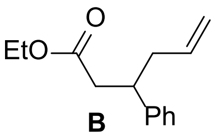

B

The product was purified by flash chromatography using as eluent a mixture of petroleum ether/ethyl acetate (100:2). The product was isolated as a pail yellowish oil (8 mg, 0.023 mmol, 12%).

^{1}H NMR (400 MHz, CDCl$_3$) δ= 7.37 – 7.26 (m, 2H), 7.24 – 7.09 (m, 3H), 5.66 (ddt, J = 17.1, 10.2, 7.0 Hz, 1H), 5.05 – 4.93 (m, 2H), 4.03 (q, J = 7.1, 2H), 3.27 – 3.15 (m, 1H), 2.68 (dd, J = 15.3, 6.7 Hz, 1H), 2.55 (dd, J = 15.3, 8.5 Hz, 1H), 2.47 – 2.32 (m, 2H), 1.13 (t, J = 7.1 Hz, 3H).

^{13}C NMR (100 MHz, CDCl$_3$) δ= 172.48, 143.74, 136.13, 128.52, 127.60, 126.65, 116.98, 60.39, 42.00, 40.81, 29.85, 14.25.

^{1}H, ^{13}C NMR spectra

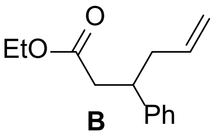

B

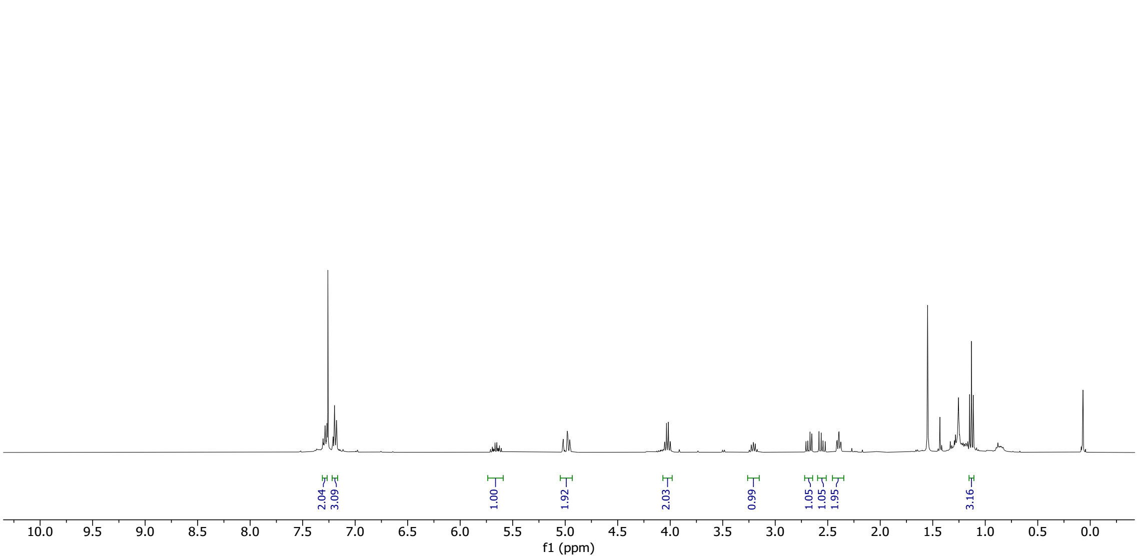

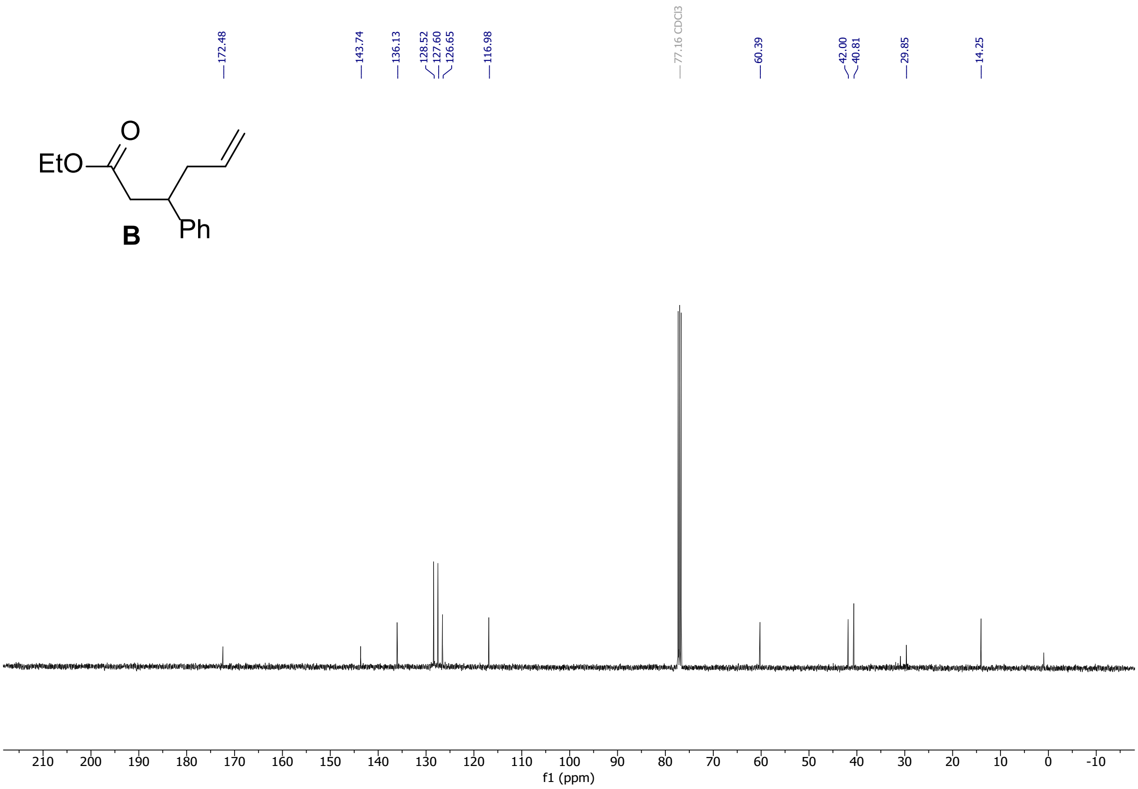

Computational Studies

Computational Details.

Geometry optimizations, transition state searches, and energy evaluations were performed with Gaussian 16 package.[5] The quantum mechanics calculations were performed within the framework of Density Functional Theory (DFT)[6] by using the ωB97X-D functional.[7] For Cu, P, K, Br, Cl and I, effective core potentials (ECPs) with double-ζ valence basis set (LANL2DZ) were employed,[8] supplemented with polarized shells with the following exponents: Cu (f = 3.525), P (d = 0.387), K (d = 1.000), Br (d = 0.428), Cl (d = 0.640), I (d = 0.289).[9,10] For all other atoms, the 6-31G(d,p) basis set was used.[11] Basis sets were obtained via the Basis Set Exchange software.[12] Solvent effects of THF were included using the implicit solvation model SMD.[13] All minima were characterized by the lack of imaginary frequencies whereas only one imaginary frequency was identified for transition-state structures, which is associated to the normal mode of vibration connecting reactants and products. Free energies are reported at a concentration of 1 M and a temperature of 298.15 K employing the quasi-harmonic correction to vibrational entropy given by GoodVibes.[14] All intermediates were considered in the singlet state, as tests of the Cu^{III} intermediate **I_6** showed the triplet state was over 1.5 eV (~35 kcal mol^{-1}) higher in energy compared with the singlet. To check our methodology, we have reevaluated the energy barrier of the rate-determining step (**I_5** → **TS2**) using a larger basis set: LANL2TZ(f) for Cu,[8,9,15,16] LANL08 for K,[¡Error! Marcador no definido.16] LANL08(d) for P, Br and I,[8,9,15,16] and 6-311++G(d,p) for other atoms.[11] The free-energy barrier obtained with the larger basis set (19.5 kcal mol^{-1}) is close to the actual value (21.4 kcal mol^{-1}). Computed structures discussed in the main text are available the ioChem-BD database[17]: https://iochem-bd.urv.es/browse/review-

collection/100/2143/e6c638110a0af5476c181eed. (temporary link for reviewing purposes).

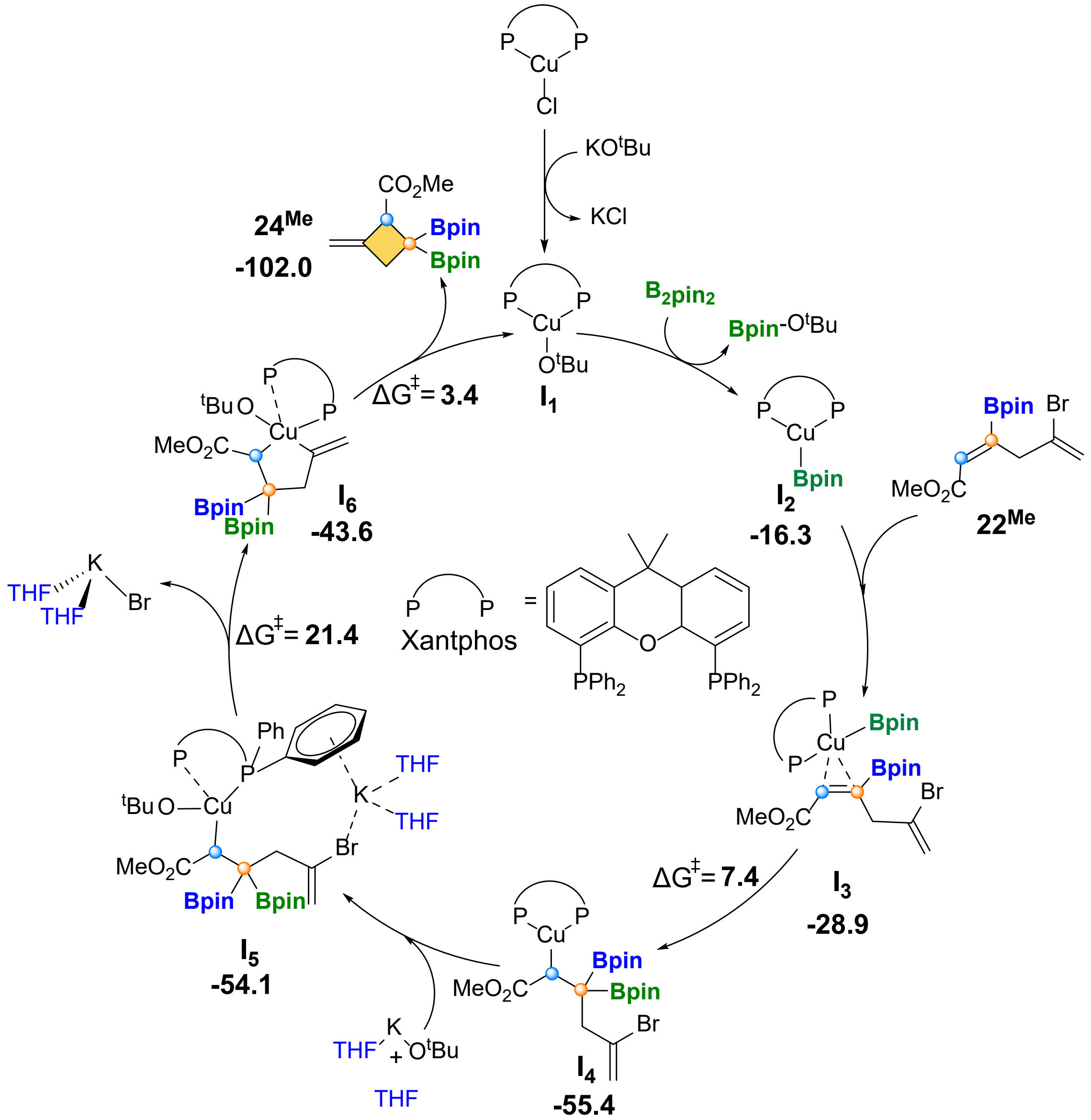

Figure S1. Proposed catalytic cycle for the for the borylcupration and ring-closing of **22^{Me}** to yield **24^{Me}**. Gibbs free energies in kcal mol^{-1}.

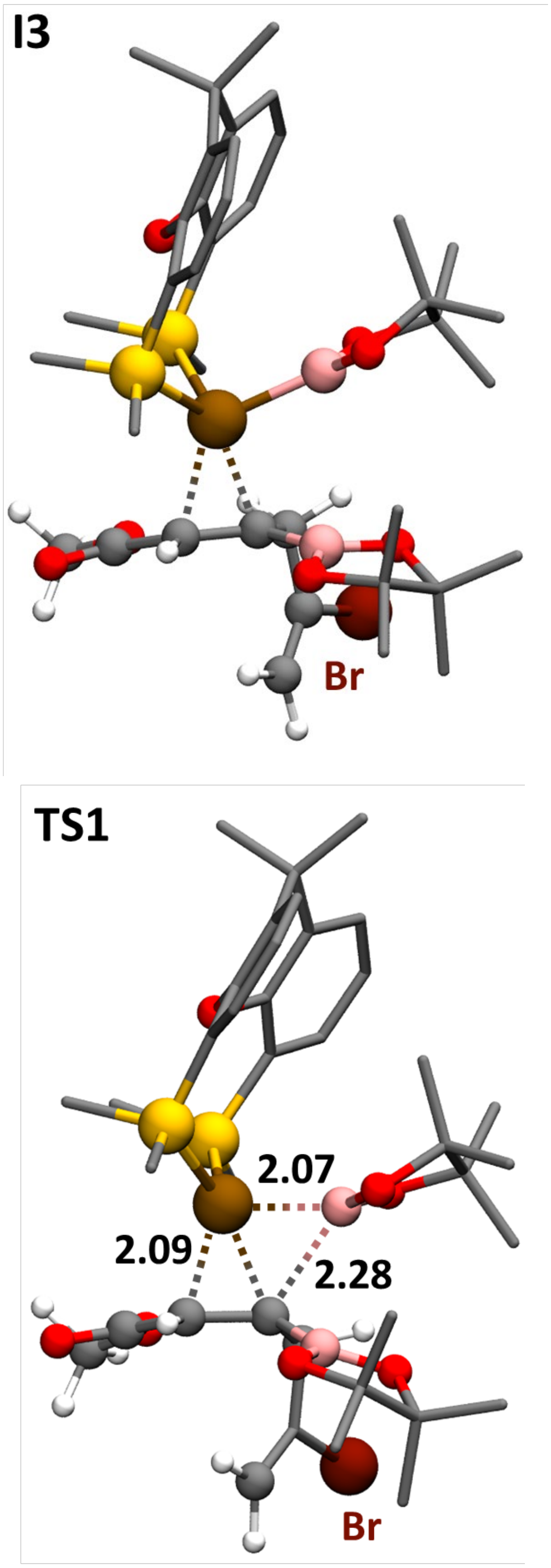

Figure S2. 3D representations of computed structures **I$_3$** and **TS1**. The Ph substituents of Xantphos ligand are omitted for clarity. Relevant distances are reported in Å.

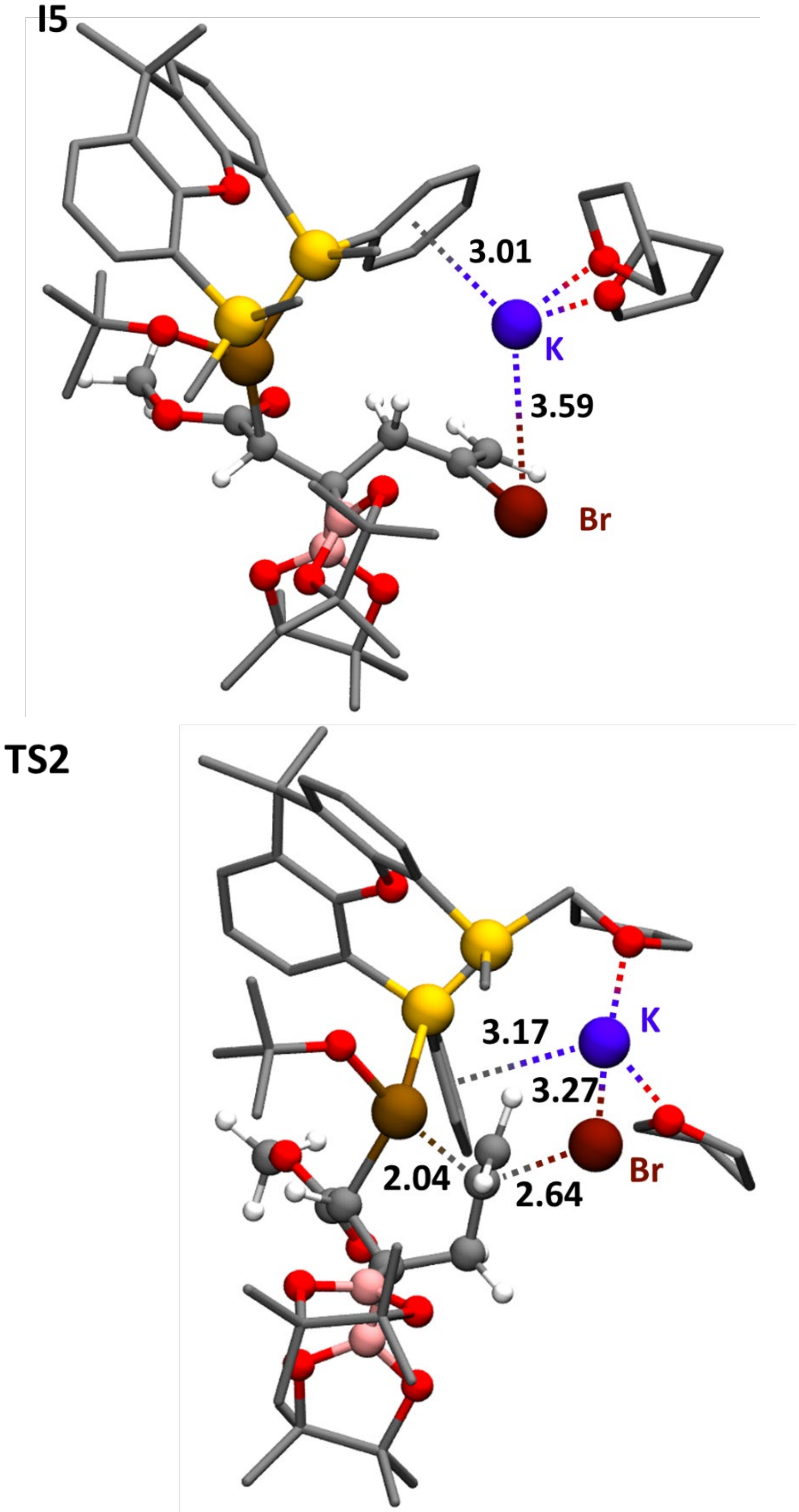

Figure S3. 3D representations of computed structures **I$_5$** and **TS2**. Some of the Ph substituents of Xantphos ligand are omitted for clarity. The model includes two solvent THF molecules coordinated to K cation. In **I$_5$**, the **K$^+$** does also interact with the Br atom and the aromatic ring of a phenyl substituent. Relevant distances are reported in Å.

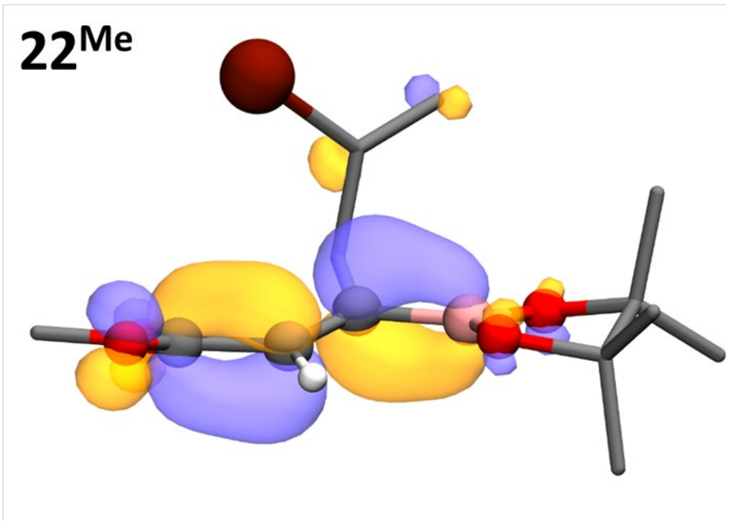

Figure S4. 3D representation of the Lowest Unoccupied Molecular Orbital (LUMO) of reactant **22^{Me}**, corresponding C=C π* interaction with some contribution of the C=C π* interaction and the empty p orbital perpendicular to the boryl plane.

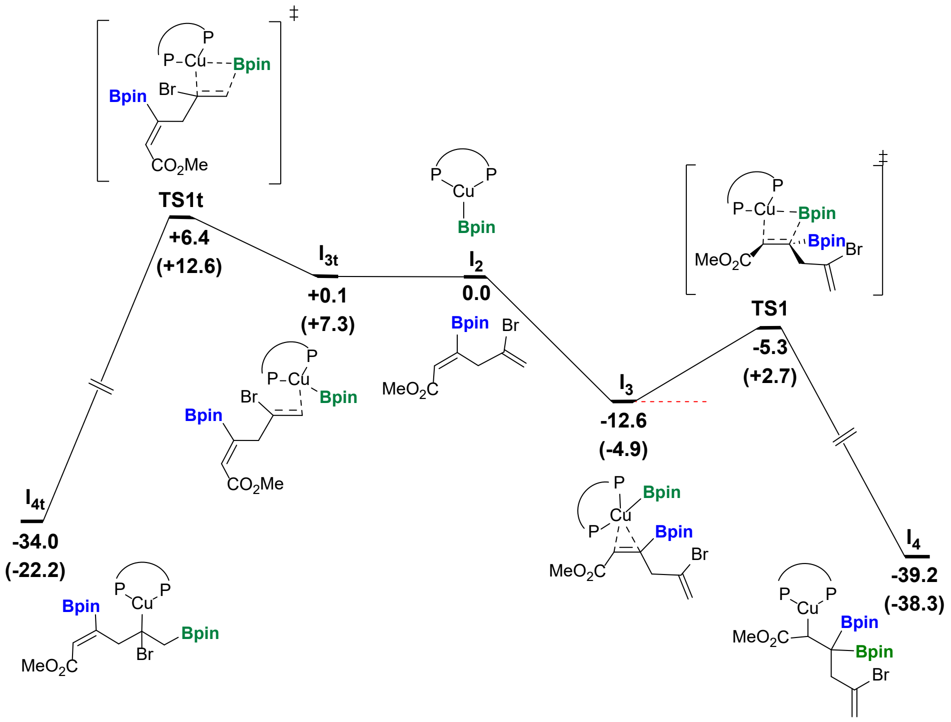

Figure S5. Free-energy profile (kcal·mol^{-1}) for the regioselective borylcupration of substrate **22^{Me}** setting the zero intermediate **I$_2$**. In parenthesis, we show the free-energy values for the analogous reaction of substrate **20**.

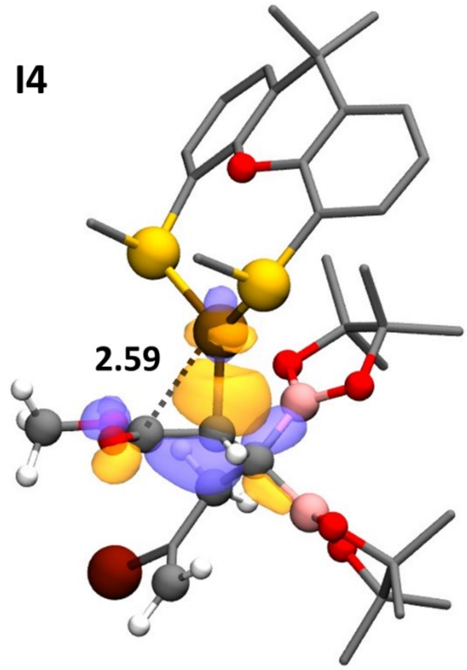

Figure S6. 3D representation of the Highest Occupied Molecular Orbital (HOMO) of intermediate **I₄**.

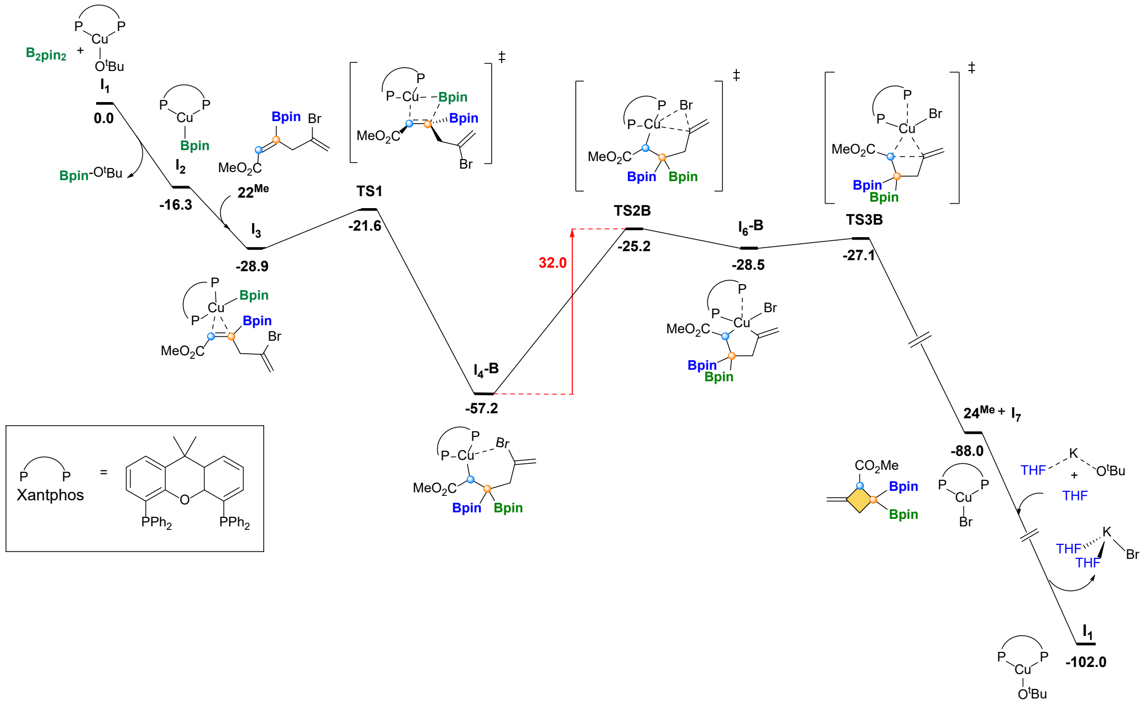

Figure S7. Free-energy profile (kcal mol^{-1}) for the Cu-catalyzed borylcupration with concomitant ring closing ring-closing of (Z)-skipped dienoate **22^{Me}** through a mechanism involving the oxidative addition of C(*sp2*)-Br bond to Cu(I) without the assistance of potassium atom (mechanism **B**). The overall free-energy barrier through transition state **TS2B** is too high (33.9 kcal mol^{-1}), and therefore, the mechanism is not feable.

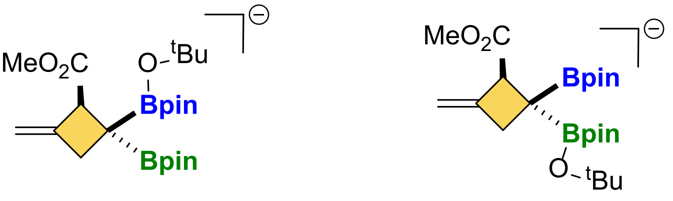

+0.8 **0.0**

(+1.9) **0.0**

Figure S8. Comparison of the activation of the two Bpin moieties in product **24** by a tertbutoxide base. Free-energy barriers in kcal·mol^{-1}. In parenthesis values computed with a larger basis set, 6-311g++(d,p).

Cartesian coordinates in Å

B2pin2

B	0.852117	-0.005275	-0.003533
O	1.615348	1.129414	0.103623
O	1.610007	-1.143417	-0.111977
C	2.986152	-0.734507	-0.303190
B	-0.854713	-0.001402	-0.002103
O	-1.617759	-1.135988	0.107440
O	-1.612787	1.136632	-0.110389
C	-2.992035	-0.720654	0.298227
C	-2.989248	0.727532	-0.298896
C	-3.900964	1.714181	0.414584
H	-3.833461	2.694248	-0.066596
H	-4.941976	1.380053	0.359836
H	-3.626498	1.831704	1.464779
C	-3.256224	0.759811	-1.803939
H	-4.300292	0.524606	-2.029550
H	-3.040621	1.763254	-2.181507
H	-2.615732	0.051356	-2.337774
C	-3.256666	-0.751743	1.803711
H	-4.299294	-0.511864	2.031049
H	-3.044939	-1.756143	2.180927
H	-2.612123	-0.046167	2.336482
C	-3.909392	-1.703149	-0.413750
H	-3.845495	-2.683533	0.067277
H	-4.948794	-1.364334	-0.357230
H	-3.637234	-1.821859	-1.464412
C	3.899056	-1.720450	0.409744
H	3.830709	-2.701008	-0.070315
H	4.939983	-1.386408	0.352882
H	3.626374	-1.836886	1.460525
C	3.250620	-0.768264	-1.808644
H	4.294329	-0.533360	-2.036219
H	3.034313	-1.772054	-2.184885
H	2.609296	-0.060273	-2.342096
C	2.989944	0.714262	0.292503
C	3.257114	0.746810	1.797507
H	4.300120	0.507132	2.023319
H	3.046039	1.751580	2.174103
H	2.613458	0.041764	2.332050
C	3.906104	1.696057	-0.421978
H	3.843024	2.676909	0.058204
H	4.945598	1.357290	-0.366884
H	3.632172	1.813747	-1.472293

I_1

Cu	0.001628	-0.597857	-1.489335
O	-0.512502	-2.220778	-2.402596
C	-0.274927	-3.443272	-1.794072
C	-0.821119	-3.463113	-0.349651
C	-0.990282	-4.538953	-2.606399
C	1.233670	-3.759172	-1.745091
H	-0.279848	-2.733529	0.267224
H	-1.881345	-3.184968	-0.345866
H	-0.719872	-4.445717	0.128772
H	-0.625045	-4.534786	-3.639587
H	-0.835603	-5.542324	-2.190166
H	-2.068025	-4.339694	-2.629856
H	1.444977	-4.735547	-1.290450
H	1.651927	-3.754693	-2.758442
H	1.756279	-2.991910	-1.159701
P	1.947622	0.292578	-0.663005
P	-1.914861	0.416689	-0.469103
C	3.501497	0.144850	-1.628539
C	4.650793	0.887227	-1.333926
C	3.522195	-0.763782	-2.690848
C	5.806718	0.713398	-2.088193
H	4.642114	1.602135	-0.515955
C	4.682230	-0.939148	-3.442134
H	2.627575	-1.334159	-2.927225
C	5.823828	-0.201234	-3.140983
H	6.695094	1.292536	-1.855417
H	4.690052	-1.647633	-4.264686
H	6.727183	-0.334201	-3.728604
C	1.897797	2.062319	-0.169889
C	2.401348	2.536687	1.043961
C	1.313367	2.961534	-1.067822
C	2.326701	3.893353	1.351178
H	2.850789	1.849156	1.754594
C	1.254025	4.317911	-0.766453
H	0.895747	2.597309	-2.003468
C	1.757478	4.785034	0.445901
H	2.715378	4.252414	2.299321
H	0.797253	5.006670	-1.469644
H	1.698272	5.842010	0.687175
C	-2.025133	2.225550	-0.145237
C	-1.756376	2.784299	1.106283
C	-2.299019	3.075939	-1.224704
C	-1.773205	4.166734	1.278122
H	-1.539442	2.147736	1.957876
C	-2.330805	4.454888	-1.047049
H	-2.502271	2.658133	-2.207581

C	-2.066107	5.004617	0.206209
H	-1.559734	4.586885	2.256234
H	-2.558003	5.100438	-1.890087
H	-2.084085	6.081328	0.344577
C	-3.493033	0.018535	-1.318956
C	-3.473237	-1.071190	-2.198834
C	-4.685978	0.718793	-1.102874
C	-4.646192	-1.454989	-2.847620
H	-2.534429	-1.609507	-2.368926
C	-5.850152	0.332576	-1.759984
H	-4.705060	1.565577	-0.422663
C	-5.831327	-0.756460	-2.631154
H	-4.629992	-2.300837	-3.528933
H	-6.772745	0.879711	-1.590679
H	-6.741261	-1.056198	-3.142801
C	2.324294	-0.587971	0.904995
C	1.309814	-0.637173	1.862940
C	3.500635	-1.292157	1.166630
C	1.414213	-1.342816	3.058824
C	3.629634	-2.022442	2.344669
H	4.309997	-1.282817	0.443864
C	-1.014643	-0.507978	2.012940
C	2.597252	-2.048343	3.279250
H	4.543570	-2.574726	2.537703
C	-2.133913	-0.359791	1.190867
C	-1.033586	-1.196797	3.225694
H	2.724101	-2.621350	4.191175
C	-3.332134	-0.930419	1.627611
C	-2.244085	-1.765868	3.616753
C	-3.384004	-1.629221	2.828497
H	-4.223315	-0.849400	1.014621
H	-2.307228	-2.319954	4.546685
H	-4.318853	-2.075587	3.151557
O	0.159765	0.057707	1.573640
C	0.255122	-1.229112	4.047633
C	0.390461	0.120832	4.792022
H	1.320960	0.140661	5.368618
H	0.403183	0.963695	4.094105
H	-0.449871	0.257599	5.480239
C	0.257279	-2.365381	5.072299
H	-0.561591	-2.241482	5.785979
H	0.157425	-3.343783	4.592638
H	1.182455	-2.355534	5.654563

Bpin-O^{t}Bu

B	-1.655301	-1.317227	-0.665035
O	-0.745271	-0.281368	-0.695093
O	-1.096032	-2.516760	-0.288458
C	0.563133	-0.879325	-0.564264
C	0.237452	-2.222925	0.177850
C	1.155258	-3.381973	-0.179277
H	2.192263	-3.141177	0.075422
H	0.864886	-4.271869	0.386892
H	1.102829	-3.623806	-1.242774
C	0.160839	-2.059676	1.696163
H	-0.265018	-2.969018	2.129346
H	1.151032	-1.901527	2.132940
H	-0.481021	-1.218053	1.974306
C	1.467494	0.071379	0.203861
H	2.436438	-0.398545	0.399781
H	1.642420	0.974962	-0.387574
H	1.023579	0.368857	1.155982
C	1.100973	-1.093568	-1.979051
H	1.111583	-0.134105	-2.504023
H	2.120918	-1.488055	-1.964527
H	0.468414	-1.784322	-2.545052
O	-2.972994	-1.240447	-0.959289
C	-3.631932	-0.062989	-1.466493
C	-3.544760	1.062490	-0.435472
C	-3.000767	0.349481	-2.796763
C	-5.084080	-0.484959	-1.673359
H	-3.959776	0.731261	0.521842
H	-2.508105	1.371092	-0.279818
H	-4.117355	1.931063	-0.775452
H	-3.035725	-0.483291	-3.506536
H	-3.548416	1.192475	-3.229396
H	-1.958719	0.648140	-2.657847
H	-5.674662	0.350462	-2.061027
H	-5.143699	-1.312815	-2.386497
H	-5.525503	-0.812543	-0.727123

I_2

C	-0.864100	-2.380690	1.021016
C	-1.681435	-1.453363	1.667984
C	-2.248875	-1.664469	2.922330
C	-1.957437	-2.873010	3.554668
C	-1.149850	-3.825511	2.936698
C	-0.610623	-3.587258	1.675753
H	-2.366739	-3.083110	4.536940
H	-0.940381	-4.762238	3.443092
H	0.020786	-4.334531	1.206031
C	-3.190623	-0.588245	3.462173
C	-2.623903	0.765538	3.031928
C	-2.028071	0.849733	1.773911
C	-2.700281	1.939787	3.778796
C	-1.518479	2.032502	1.233577

C	-2.210711	3.140632	3.270335
H	-3.147722	1.926636	4.766659
C	-1.625667	3.189890	2.009410
H	-2.281094	4.045473	3.865268
H	-1.233764	4.130055	1.637055
O	-1.921097	-0.278587	0.997981
P	-0.124213	-1.869005	-0.584198
P	-0.602789	1.928178	-0.365913
Cu	1.048433	0.201687	-0.302672
B	3.048024	0.323697	0.153331
O	3.695170	-0.433453	1.140651
O	4.001421	1.129705	-0.481706
C	5.120263	-0.295716	0.993728
C	5.244289	1.066108	0.242522
C	-1.530948	-1.986674	-1.761274
C	-1.357390	-1.396531	-3.018129
C	-2.741510	-2.617800	-1.464008
C	-2.369916	-1.455294	-3.969447
H	-0.427243	-0.883269	-3.250196
C	-3.761655	-2.660292	-2.411929
H	-2.891755	-3.078503	-0.491987
C	-3.575950	-2.084327	-3.665858
H	-2.224456	-0.993260	-4.940671
H	-4.700884	-3.148703	-2.169947
H	-4.371791	-2.118688	-4.403798
C	-1.916121	1.872002	-1.660636
C	-1.551774	2.210615	-2.970774
C	-3.219027	1.434563	-1.412754
C	-2.479921	2.137832	-4.003858
H	-0.539987	2.546465	-3.183876
C	-4.143908	1.351683	-2.451369
H	-3.527646	1.164783	-0.408439
C	-3.780501	1.708413	-3.746420
H	-2.185175	2.414497	-5.011689
H	-5.152720	1.008746	-2.242752
H	-4.504435	1.646132	-4.553213
C	0.074165	3.626529	-0.576723
C	1.450966	3.813934	-0.422429
C	-0.739213	4.726604	-0.880365
C	2.004427	5.086235	-0.559288
H	2.092280	2.961439	-0.210322
C	-0.183854	5.994077	-1.016869
H	-1.809015	4.589433	-1.010846
C	1.189865	6.175218	-0.854421
H	3.075137	5.222402	-0.440964
H	-0.821605	6.840871	-1.251677
H	1.622882	7.165067	-0.963944
C	0.932111	-3.312584	-1.016990
C	2.264786	-3.287753	-0.586774
C	0.463654	-4.416038	-1.736873
C	3.111599	-4.356127	-0.869400
H	2.638853	-2.430267	-0.031211
C	1.316533	-5.478579	-2.025131
H	-0.567578	-4.447075	-2.076095
C	2.640438	-5.450665	-1.591382
H	4.143735	-4.327375	-0.532613
H	0.945503	-6.329352	-2.588753
H	3.304054	-6.279632	-1.818655
C	5.773410	-0.329085	2.368940
H	5.633238	-1.315353	2.822173
H	6.850128	-0.142458	2.291987
H	5.337937	0.414980	3.039269
C	5.602037	-1.489269	0.163720
H	6.690383	-1.493999	0.047373
H	5.309815	-2.412175	0.674322
H	5.142379	-1.490187	-0.829265
C	5.274760	2.272006	1.185451
H	6.217720	2.335382	1.737618
H	5.163438	3.186032	0.594218
H	4.449699	2.230840	1.902882
C	6.403836	1.150639	-0.740916
H	6.412695	2.134252	-1.220845
H	7.360918	1.018042	-0.224781
H	6.322751	0.393976	-1.524299
C	-3.364419	-0.683538	4.979379
H	-2.412891	-0.558084	5.504844
H	-4.062637	0.078477	5.335659
H	-3.790796	-1.651240	5.256669
C	-4.569963	-0.773298	2.785406
H	-4.991608	-1.748011	3.051557
H	-5.260678	0.008705	3.116993
H	-4.491398	-0.722059	1.695095

22

C	-2.179912	1.604099	-1.209452
C	-1.217307	1.607279	-2.145955
H	-0.965045	0.669911	-2.635474
C	-0.467418	2.749009	-2.731545
O	-0.001918	2.706063	-3.851447
O	-0.328082	3.804417	-1.920479
C	0.340523	4.936384	-2.484814
H	0.359510	5.689463	-1.697764
H	1.358930	4.675890	-2.782335
H	-0.205968	5.312506	-3.352939
C	-2.736733	2.817708	-0.492684

H	-2.849609	2.577620	0.569612
H	-2.080931	3.682722	-0.582691
C	-4.111928	3.150657	-1.015059
C	-5.263096	2.890833	-0.407943
H	-6.227316	3.137841	-0.837185
H	-5.243532	2.406823	0.564107
Br	-4.123859	3.986106	-2.757302
B	-2.856777	0.231407	-0.861906
O	-2.788647	-0.873939	-1.661241
O	-3.577360	0.039036	0.283831
C	-4.256135	-1.234175	0.140127
C	-4.342883	-1.905994	1.500784
H	-4.991805	-1.322724	2.160441
H	-4.771658	-2.908167	1.402811
H	-3.362498	-1.989641	1.973779
C	-5.656003	-0.925817	-0.390384
H	-6.160423	-0.251279	0.307051
H	-5.614461	-0.434695	-1.367324
H	-6.255984	-1.835627	-0.481097
C	-3.348625	-1.976396	-0.902766
C	-2.174239	-2.713754	-0.261658
H	-2.510464	-3.603414	0.278074
H	-1.481526	-3.029171	-1.046712
H	-1.629312	-2.068896	0.434388
C	-4.095658	-2.897182	-1.854024
H	-3.389144	-3.370219	-2.542036
H	-4.606295	-3.687645	-1.295099
H	-4.834507	-2.352551	-2.445084

I_3

Cu	0.230287	0.252491	0.463148
C	2.273313	0.063869	1.059684
C	1.496674	0.035284	2.223414
P	-0.879247	-2.036387	-0.148138
P	-1.643438	1.498087	1.293200
B	0.660925	0.788117	-1.477943
O	0.942124	-0.178725	-2.438092
O	0.613940	2.038452	-2.081991
C	-4.584392	-0.414319	4.327550
C	-2.391190	0.379854	3.712726
C	-4.194084	0.466459	2.112315
C	-5.049225	-0.105852	3.053145
C	-3.251629	-0.168512	4.655947
H	-4.582198	0.728085	1.133331
H	-6.084285	-0.299324	2.788187
H	-5.255229	-0.851282	5.061116
C	-2.159848	-2.830397	0.916191
C	-3.975235	-4.094286	2.632937
C	-3.156935	-3.667925	0.402543
C	-2.091193	-2.626080	2.295619
C	-2.986930	-3.263563	3.151662
C	-4.062928	-4.290163	1.255439
H	-1.343792	-1.957126	2.709589
H	-2.917252	-3.095624	4.221442
H	-4.679517	-4.584395	3.298494
C	-1.786127	-1.912262	-1.755005
C	-3.201621	-1.473481	-4.138753
C	-1.619801	-2.764846	-2.847342
C	-2.700866	-0.868412	-1.891586
C	-3.420565	-0.617771	-3.059414
C	-2.317210	-2.542723	-4.030569
H	-0.931085	-3.599403	-2.783259
H	-2.169844	-3.208451	-4.874901
H	-3.733717	-1.318808	-5.070888
C	-2.670038	2.158262	-0.084763
C	-4.097479	2.991177	-2.346756
C	-2.914681	3.514648	-0.305386
C	-3.153938	1.251811	-1.027440
C	-3.883671	1.626865	-2.153442
C	-3.615912	3.926102	-1.434434
H	-2.544502	4.253776	0.396619
H	-3.793087	4.983537	-1.602077
H	-4.648444	3.335655	-3.214912
C	-1.242604	3.024111	2.238869
C	-0.545807	5.354623	3.618463
C	0.049251	3.539215	2.144968
C	-2.190002	3.687227	3.030050
C	-1.842225	4.847094	3.713640
C	0.398049	4.699308	2.834344
H	0.795504	3.033503	1.542999
H	-3.200350	3.297128	3.111551
H	-2.582683	5.354969	4.324200
H	1.412376	5.080362	2.760030
H	-0.275231	6.256537	4.159400
C	0.261324	-3.448836	-0.483336
C	2.123293	-5.481552	-0.987507
C	0.224877	-4.638997	0.246755
C	1.248395	-3.283067	-1.465526
C	2.164440	-4.296894	-1.721115
C	1.155645	-5.647386	-0.001529
H	-0.525603	-4.787500	1.016558
H	1.298203	-2.346255	-2.016008
H	2.922933	-4.153975	-2.484632
H	1.117860	-6.565640	0.577290
H	2.846253	-6.268494	-1.180352

H	-1.345774	0.533409	3.967898
H	-3.224574	-3.838751	-0.667813
H	-2.876739	-0.417268	5.644252
H	-4.834689	-4.934194	0.844426
O	-2.882931	-0.071415	-0.788574
C	-2.860367	0.715887	2.436070
C	-4.438173	0.522014	-3.050472
C	-5.745241	-0.006138	-2.413641
H	-6.487114	0.796680	-2.350778
H	-6.159073	-0.818544	-3.019856
H	-5.568689	-0.390400	-1.404159
C	-4.737898	1.032851	-4.462133
H	-5.491404	1.824279	-4.433872
H	-3.838441	1.423944	-4.948133
H	-5.150630	0.234434	-5.083792
C	0.632121	1.871848	-3.515317
C	1.291436	0.464172	-3.680011
C	0.744851	-0.366756	-4.831846
H	-0.328482	-0.539542	-4.731192
H	0.934513	0.128264	-5.790444
H	1.241796	-1.341936	-4.851208
C	2.818218	0.512075	-3.736604
H	3.219035	1.062951	-2.881844
H	3.205921	-0.510567	-3.686524
H	3.177962	0.968101	-4.664415
C	1.406470	3.022178	-4.140386
H	1.483011	2.891413	-5.225044
H	0.885393	3.965133	-3.948044
H	2.414734	3.101585	-3.730363
C	-0.822591	1.911605	-3.985565
H	-1.284875	2.839275	-3.636242
H	-0.895994	1.874473	-5.077251
H	-1.388456	1.075052	-3.569653
H	1.313324	0.976228	2.735957
C	1.136858	-1.110555	3.071571
O	0.608067	-0.986567	4.166019
O	1.463726	-2.316412	2.575067
C	1.226537	-3.438239	3.421212
H	0.158539	-3.575891	3.608169
H	1.622368	-4.298587	2.882150
H	1.746252	-3.320563	4.375539
B	2.931772	1.398260	0.578039
O	2.899885	2.590915	1.259232
O	3.778251	1.432207	-0.504629
C	3.617565	3.561406	0.454974
C	4.560341	2.639845	-0.388914
C	4.339501	4.526307	1.381396
H	4.979670	5.199601	0.802412
H	3.611775	5.137351	1.923421
H	4.955891	4.000624	2.113401
C	2.591486	4.304190	-0.399032
H	1.872935	4.806052	0.255454
H	3.075077	5.068795	-1.014372
H	2.039436	3.616850	-1.047721
C	4.886831	3.163957	-1.776786
H	5.423421	4.115596	-1.707828
H	5.526894	2.449097	-2.302182
H	3.983824	3.316461	-2.369156
C	5.847233	2.262280	0.345919
H	6.339623	1.451903	-0.198720
H	6.536747	3.109306	0.405550
H	5.639565	1.907864	1.359890
C	2.917962	-1.156055	0.426466
H	2.314310	-2.054605	0.540402
H	3.030207	-0.972300	-0.644832
C	4.278497	-1.424266	1.018251
C	4.812801	-0.920412	2.124207
H	5.805374	-1.187952	2.467364
H	4.244242	-0.214946	2.722982
Br	5.314839	-2.694112	-0.008339

TS1

Cu	0.060203	-0.065634	-0.546706
C	2.178841	-0.043108	-0.635348
C	1.581579	-0.061964	-1.980506
P	-1.098394	1.988472	0.081189
P	-1.532812	-1.678116	-1.131105
B	0.987246	-0.536499	1.246039
O	1.025420	-1.821649	1.744207
O	1.059500	0.397266	2.254506
C	-4.231875	-0.160282	-4.574823
C	-2.064611	-0.668263	-3.644803
C	-4.050398	-0.952198	-2.300186
C	-4.829194	-0.499869	-3.365116
C	-2.847052	-0.247484	-4.712921
H	-4.532635	-1.243834	-1.372056
H	-5.906245	-0.426174	-3.248492
H	-4.841822	0.180864	-5.405945
C	-2.392066	2.552705	-1.097154
C	-4.212108	3.515014	-2.988048
C	-3.558048	3.206722	-0.685819
C	-2.151896	2.374785	-2.461421
C	-3.052549	2.866490	-3.402685
C	-4.467693	3.677583	-1.627673
H	-1.262681	1.847684	-2.794464

H	-2.852162	2.725963	-4.459815
H	-4.918719	3.889244	-3.722782
C	-2.041270	1.861183	1.666131
C	-3.495045	1.441468	4.032061
C	-1.942221	2.758535	2.731420
C	-2.909077	0.779121	1.821105
C	-3.650920	0.541669	2.978286
C	-2.654239	2.543455	3.906955
H	-1.295464	3.624480	2.652623
H	-2.555919	3.243465	4.730331
H	-4.044711	1.296532	4.955540
C	-2.634613	-2.321515	0.192119
C	-4.135565	-3.114298	2.421594
C	-2.813845	-3.676922	0.477871
C	-3.232957	-1.396147	1.046085
C	-3.991675	-1.752670	2.159361
C	-3.555480	-4.067403	1.588008
H	-2.357703	-4.429224	-0.156342
H	-3.681934	-5.122797	1.806576
H	-4.707703	-3.443120	3.282134
C	-0.940097	-3.215864	-1.944052
C	0.041415	-5.513840	-3.194689
C	0.384313	-3.606371	-1.755235
C	-1.776263	-3.987651	-2.761737
C	-1.288568	-5.134203	-3.378177
C	0.875056	-4.748505	-2.386017
H	1.045995	-3.013585	-1.134485
H	-2.808675	-3.689056	-2.919828
H	-1.943122	-5.727285	-4.009593
H	1.915615	-5.028076	-2.252638
H	0.425639	-6.401269	-3.688755
C	-0.124538	3.533446	0.355489
C	1.438643	5.814880	0.808535
C	-0.334599	4.694996	-0.391018
C	0.880384	3.525981	1.332315
C	1.651046	4.660678	1.559902
C	0.446300	5.827935	-0.166746
H	-1.102985	4.724675	-1.156433
H	1.059591	2.621973	1.908073
H	2.427160	4.637668	2.318617
H	0.273603	6.722202	-0.758176
H	2.045640	6.698437	0.981689
H	-0.981359	-0.685883	-3.747600
H	-3.755177	3.354799	0.371849
H	-2.371687	0.032833	-5.648104
H	-5.372946	4.178999	-1.298634
O	-3.024080	-0.072270	0.749754
C	-2.665559	-1.041783	-2.435472
C	-4.630100	-0.631657	2.975878
C	-5.921582	-0.179267	2.254291
H	-6.633363	-1.009354	2.199007
H	-6.389009	0.646444	2.800495
H	-5.711632	0.161130	1.235679
C	-4.981754	-1.087806	4.393832
H	-5.697533	-1.913459	4.366248
H	-4.093810	-1.414285	4.944189
H	-5.460379	-0.279487	4.952287
C	1.300790	-0.289529	3.506299
C	0.818084	-1.742062	3.177923
C	1.607553	-2.845891	3.860566
H	2.667531	-2.800510	3.606050
H	1.507990	-2.766325	4.947967
H	1.220501	-3.822561	3.555088
C	-0.678207	-1.942157	3.411985
H	-1.253945	-1.133148	2.957676
H	-0.992098	-2.882651	2.950522
H	-0.917713	-1.980329	4.478932
C	0.507987	0.412710	4.597807
H	0.604595	-0.123692	5.547493
H	0.895800	1.425986	4.740202
H	-0.550957	0.489978	4.343010
C	2.799555	-0.193579	3.782492
H	3.083760	0.862429	3.826210
H	3.059955	-0.656152	4.739385
H	3.375675	-0.667928	2.983300
H	1.576941	-1.017238	-2.496604
C	1.302512	1.031620	-2.872342
O	0.987632	0.905641	-4.057001
O	1.406327	2.279726	-2.324341
C	1.278564	3.370052	-3.219930
H	0.262855	3.452425	-3.619981
H	1.511837	4.260989	-2.635121
H	1.978708	3.281470	-4.055830
B	3.092628	-1.272879	-0.276896
O	3.175022	-2.445604	-0.982894
O	4.005379	-1.213463	0.748678
C	4.064315	-3.329612	-0.255296
C	4.920310	-2.315857	0.578744
C	4.858609	-4.157254	-1.252832
H	5.610352	-4.759147	-0.732230
H	4.191058	-4.840109	-1.786208
H	5.362188	-3.529310	-1.990348
C	3.195273	-4.233307	0.617141
H	2.526322	-4.818146	-0.019744

H	3.807381	-4.931727	1.195033
H	2.579995	-3.640618	1.298053
C	5.353811	-2.818764	1.946259
H	5.991791	-3.702335	1.843584
H	5.928892	-2.042305	2.459286
H	4.497689	-3.079969	2.570476
C	6.129038	-1.775325	-0.185501
H	6.538929	-0.921153	0.360458
H	6.911329	-2.533763	-0.281682
H	5.849653	-1.431211	-1.185095
C	2.820841	1.250826	-0.120256
H	2.144981	2.097944	-0.217849
H	3.051686	1.144570	0.941691
C	4.098360	1.604652	-0.835547
C	4.639747	1.073395	-1.924384
H	5.578201	1.422556	-2.339204
H	4.127438	0.264429	-2.436100
Br	5.031271	3.076281	0.019037

I_4

Cu	-0.245492	-0.009397	-0.535105
C	2.819293	0.045202	0.131967
C	1.786060	0.004164	-1.044859
P	-1.477660	1.943075	0.046256
P	-1.714876	-1.759245	-1.142051
B	2.016292	-0.225691	1.460428
O	1.637906	-1.492264	1.852127
O	1.671057	0.723603	2.394056
C	-4.262744	-0.534439	-4.823042
C	-2.164337	-1.188893	-3.829618
C	-4.138855	-1.007092	-2.457080
C	-4.874124	-0.621004	-3.576058
C	-2.904199	-0.819059	-4.946921
H	-4.638775	-1.085746	-1.497459
H	-5.930391	-0.393360	-3.469173
H	-4.839961	-0.239308	-5.694049
C	-2.603358	2.326481	-1.353189
C	-4.173753	2.955478	-3.576175
C	-3.913117	2.781913	-1.183299
C	-2.087207	2.175966	-2.645885
C	-2.868597	2.500304	-3.750499
C	-4.696809	3.090420	-2.292634
H	-1.072814	1.807315	-2.795675
H	-2.460980	2.380299	-4.749226
H	-4.784858	3.196791	-4.440741
C	-2.589908	1.860314	1.511430
C	-4.145806	1.435187	3.804136
C	-2.598121	2.798945	2.545756
C	-3.402969	0.734056	1.654855
C	-4.192340	0.491900	2.777486
C	-3.363970	2.581189	3.686621
H	-1.982411	3.688813	2.471926
H	-3.351407	3.309819	4.490672
H	-4.734713	1.287349	4.702627
C	-2.883253	-2.404314	0.128208
C	-4.450907	-3.204035	2.312045
C	-3.022250	-3.758046	0.444016
C	-3.558154	-1.482887	0.931822
C	-4.353505	-1.844641	2.018440
C	-3.795515	-4.151898	1.530824
H	-2.505507	-4.507727	-0.145023
H	-3.887409	-5.205944	1.771926
H	-5.048205	-3.534850	3.154632
C	-0.879316	-3.275028	-1.758580
C	0.491045	-5.516234	-2.713383
C	0.459178	-3.480263	-1.413456
C	-1.528442	-4.205486	-2.581320
C	-0.848298	-5.323213	-3.051284
C	1.140326	-4.595288	-1.897736
H	0.981035	-2.766660	-0.781680
H	-2.567184	-4.050940	-2.859183
H	-1.359625	-6.038631	-3.688148
H	2.187152	-4.727314	-1.648553
H	1.027753	-6.380626	-3.092860
C	-0.604233	3.537458	0.327926
C	0.819322	5.890749	0.831345
C	-1.030557	4.749057	-0.220809
C	0.540263	3.512078	1.131969
C	1.243802	4.684343	1.387247
C	-0.317460	5.920754	0.027606
H	-1.915216	4.784495	-0.849231
H	0.887919	2.571651	1.550637
H	2.133772	4.652121	2.008958
H	-0.652819	6.856804	-0.408835
H	1.375131	6.804046	1.022096
H	-1.104557	-1.403479	-3.936300
H	-4.322959	2.902918	-0.184644
H	-2.415744	-0.747001	-5.913903
H	-5.715131	3.440520	-2.152282
O	-3.398038	-0.157431	0.611129
C	-2.779217	-1.300232	-2.575746
C	-5.099190	-0.737400	2.761758
C	-6.370716	-0.384271	1.953935
H	-7.029366	-1.256161	1.886406
H	-6.915062	0.428407	2.445758

H	-6.121994	-0.062488	0.937917
C	-5.508578	-1.168812	4.171948
H	-6.178322	-2.031699	4.131253
H	-4.639371	-1.430257	4.783421
H	-6.060398	-0.370079	4.674161
C	1.207931	0.038953	3.579711
C	0.800996	-1.370080	3.020638
C	1.094026	-2.535876	3.952819
H	2.156504	-2.601731	4.196179
H	0.527238	-2.434965	4.883841
H	0.794702	-3.472328	3.472840
C	-0.646171	-1.435128	2.542408
H	-0.869007	-0.614751	1.851217
H	-0.800414	-2.376725	2.009339
H	-1.354252	-1.390640	3.374787
C	0.063461	0.833688	4.187388
H	-0.347759	0.312613	5.057808
H	0.425415	1.812589	4.516021
H	-0.739322	0.993715	3.466056
C	2.396014	-0.012964	4.539030
H	2.737296	1.007071	4.736777
H	2.123038	-0.475723	5.491674
H	3.231093	-0.571146	4.104349
H	1.898550	-0.905050	-1.635707
C	1.603964	1.100542	-1.973333
O	1.278668	0.998875	-3.161791
O	1.742412	2.342401	-1.423059
C	1.641415	3.442941	-2.303963
H	0.649331	3.510592	-2.761583
H	1.821849	4.328891	-1.691923
H	2.393020	3.385564	-3.098411
B	3.796223	-1.188272	0.134720
O	3.672601	-2.342196	-0.602207
O	4.869506	-1.229077	0.997598
C	4.576911	-3.312863	-0.021790
C	5.643893	-2.398056	0.669432
C	5.135343	-4.204964	-1.118982
H	5.922029	-4.851598	-0.716852
H	4.349858	-4.849370	-1.523650
H	5.550844	-3.618663	-1.941032
C	3.766419	-4.134611	0.980603
H	2.926871	-4.608267	0.464861
H	4.375553	-4.921329	1.435101
H	3.354809	-3.496809	1.767399
C	6.242108	-2.968996	1.946173
H	6.754618	-3.914620	1.742231
H	6.975123	-2.267630	2.355671
H	5.476073	-3.141922	2.705060
C	6.755265	-1.946656	-0.279051
H	7.328891	-1.150215	0.203233
H	7.437459	-2.767427	-0.518803
H	6.345714	-1.547389	-1.211613
C	3.648799	1.337658	0.307045
H	2.998137	2.196276	0.479804
H	4.281063	1.234913	1.197617
C	4.553929	1.671838	-0.844860
C	4.831591	0.957230	-1.928276
H	5.520207	1.293573	-2.694439
H	4.334678	0.003479	-2.081585
Br	5.416650	3.401058	-0.643823

THF

O	5.258629	2.020812	-1.500054
C	5.787706	2.881140	-0.493715
C	4.850947	2.790367	-2.628960
C	5.990608	4.243546	-1.152984
H	5.073879	2.953272	0.340444
H	6.717033	2.447755	-0.108059
C	4.858140	4.250501	-2.181869
H	5.558384	2.631767	-3.456574
H	3.863746	2.446048	-2.956186
H	6.962154	4.283712	-1.657383
H	5.938440	5.067858	-0.437783
H	5.023188	4.942548	-3.011060
H	3.908728	4.507804	-1.699847

$(THF)_2\cdot KO^tBu$

K	2.182369	5.414054	0.749807
O	4.111131	3.960756	-0.297351
C	5.080185	3.896303	0.767461
C	4.048849	2.701125	-0.977161
C	5.206287	2.415113	1.087599
H	6.037877	4.303928	0.411131
H	4.691971	4.500749	1.601453
C	5.077491	1.788707	-0.303649
H	3.031499	2.299675	-0.876850
H	4.252434	2.852817	-2.043100
H	4.380240	2.100011	1.735249
H	6.147500	2.171466	1.586241
H	4.754108	0.745306	-0.286186
H	6.036500	1.841306	-0.829374
O	3.226933	5.427348	2.870689
C	3.557597	5.291028	4.180828
C	2.377058	5.701721	5.098535
C	3.928403	3.821500	4.512230
C	4.773914	6.182019	4.541191

H	2.095266	6.741315	4.889755
H	1.505860	5.070418	4.882308
H	2.601384	5.617306	6.171109
H	4.799680	3.514797	3.920291
H	4.163989	3.654257	5.572707
H	3.092573	3.164701	4.240299
H	5.077175	6.111408	5.595280
H	5.632864	5.900496	3.919442
H	4.536451	7.230447	4.322726

I_5

C	1.256718	-2.107789	-1.403461
H	0.836430	-3.115694	-1.381115
C	1.676089	-1.769733	-2.741906
O	2.527651	-0.938885	-3.085270
O	0.981181	-2.444870	-3.709551
C	1.163058	-1.990361	-5.037171
H	0.504719	-2.602187	-5.657210
H	0.884416	-0.935915	-5.143779
H	2.199014	-2.112386	-5.368829
C	2.349331	-1.919604	-0.316187
C	2.665240	-0.405137	-0.086169
H	2.205386	0.127541	-0.917661
H	2.183230	-0.077489	0.840162
C	4.110216	0.021354	-0.077285
C	4.828307	0.307368	-1.158723
H	5.869378	0.607104	-1.121866
H	4.339851	0.175144	-2.122029
Br	4.931722	0.208076	1.671298
P	-3.527247	-0.836491	1.285535
P	-0.892268	1.318875	-1.208945
C	-5.018979	-0.624440	0.212150
C	-6.154814	-1.430981	0.303112
C	-5.016222	0.377174	-0.758269
C	-7.232393	-1.247785	-0.555276
H	-6.186024	-2.226111	1.040920
C	-6.103074	0.621431	-1.601649
C	-7.206667	-0.223597	-1.496758
H	-8.100002	-1.896167	-0.483785
C	-3.578365	1.676378	-2.067452
H	-8.063045	-0.080660	-2.146330
C	-4.591777	1.980914	-2.981629
C	-2.226440	1.908895	-2.336532
C	-4.218321	2.464142	-4.233952
C	-1.896241	2.417430	-3.597661
C	-2.879235	2.677303	-4.543785
H	-4.976086	2.688834	-4.976075
H	-0.857103	2.595128	-3.853096
H	-2.601270	3.056995	-5.521711
O	-3.892434	1.160233	-0.841394
C	-6.038078	1.837959	-2.518403
C	-6.398738	3.088795	-1.680920
H	-6.326043	3.991807	-2.296357
H	-7.422406	3.006030	-1.300858
H	-5.725735	3.202109	-0.825192
C	-7.013870	1.732549	-3.693236
H	-8.043375	1.655932	-3.334112
H	-6.970027	2.630967	-4.314592
H	-6.797675	0.862285	-4.320436
C	-4.084801	0.086465	2.796997
C	-3.429455	-0.131754	4.018367
C	-5.017124	1.128144	2.731321
C	-3.709357	0.648378	5.136006
H	-2.693001	-0.925526	4.100288
C	-5.286735	1.922250	3.844331
H	-5.544018	1.327763	1.803031
C	-4.636507	1.685546	5.052238
H	-3.195687	0.449301	6.072335
H	-6.014262	2.725168	3.765290
H	-4.852309	2.300011	5.921267
C	-3.746489	-2.613046	1.760891
C	-4.344570	-3.089859	2.930133
C	-3.222348	-3.529487	0.837052
C	-4.408514	-4.461265	3.180084
H	-4.764896	-2.397155	3.653052
C	-3.306400	-4.896973	1.081496
H	-2.765018	-3.131956	-0.075240
C	-3.891370	-5.367164	2.257659
H	-4.871441	-4.819935	4.095202
H	-2.900045	-5.598047	0.357376
H	-3.943930	-6.434315	2.453773
C	-1.259178	1.982558	0.473080
C	-2.158049	3.015617	0.750423
C	-0.565104	1.383039	1.532745
C	-2.363251	3.438982	2.062236
H	-2.712905	3.483904	-0.056972
C	-0.747361	1.828585	2.839679
H	0.077848	0.525324	1.343270
C	-1.652568	2.854080	3.107369
H	-3.081665	4.226761	2.267370
H	-0.208363	1.349260	3.651850
H	-1.819562	3.180417	4.128949
C	5.527615	-3.982181	-0.273606
C	5.122598	-4.055931	-1.785529
B	3.628244	-2.785820	-0.647654

O	3.764510	-3.577300	-1.760473
O	4.699967	-2.905889	0.209346
O	1.630410	-1.823444	2.180262
O	1.470098	-3.864498	1.163112
B	1.807479	-2.539970	1.020697
C	0.903922	-2.669377	3.097200
C	1.251749	-4.103007	2.571170
C	-0.568984	-2.318699	2.924264
H	-0.703367	-1.251281	3.120925
H	-0.896440	-2.511261	1.899583
H	-1.209717	-2.885959	3.605681
C	1.364919	-2.375916	4.515578
H	1.076390	-1.357478	4.793855
H	0.892637	-3.066085	5.221969
H	2.449251	-2.461305	4.613172
C	0.137352	-5.123868	2.734188
H	0.462069	-6.091921	2.341037
H	-0.113203	-5.253418	3.792231
H	-0.763392	-4.822083	2.196706
C	2.562248	-4.644795	3.141002
H	3.374613	-3.922181	3.018412
H	2.465052	-4.889846	4.202747
H	2.834458	-5.556668	2.602633
C	5.915802	-3.089074	-2.665796
H	5.434145	-3.028964	-3.645615
H	5.926198	-2.083398	-2.234421
H	6.948014	-3.424066	-2.804875
C	5.145468	-5.452754	-2.387249
H	4.850661	-5.405983	-3.439773
H	6.153232	-5.877542	-2.334846
H	4.454936	-6.123136	-1.871541
C	6.987131	-3.636920	-0.019742
H	7.176757	-3.603531	1.057236
H	7.644490	-4.395262	-0.457519
H	7.246587	-2.662700	-0.439304
C	5.134809	-5.234247	0.510365
H	5.278192	-5.044553	1.577775
H	4.082166	-5.483583	0.348284
H	5.749378	-6.094491	0.229064
C	0.457555	2.491058	-1.721408
C	0.427573	3.855888	-1.396280
C	1.530920	2.000837	-2.473443
C	1.464030	4.701671	-1.782750
H	-0.404190	4.261831	-0.829079
C	2.570427	2.850372	-2.860778
H	1.581679	0.949740	-2.750650
C	2.544624	4.196908	-2.508853
H	1.430390	5.753349	-1.514502
H	3.401243	2.450159	-3.433688
H	3.360903	4.852597	-2.795595
K	2.658859	2.686210	0.535732
O	2.050261	4.595920	2.172134
C	2.463741	4.762812	3.529022
C	0.811991	5.291448	1.957508
C	1.171177	4.961994	4.305562
H	3.029954	3.874571	3.823194
H	3.118780	5.641792	3.616600
C	0.358691	5.814878	3.325778
H	0.971436	6.098585	1.233374
H	0.094984	4.579249	1.535772
H	1.329862	5.448403	5.270548
H	0.683901	3.996234	4.472608
H	0.619885	6.871521	3.438678
H	-0.720091	5.712568	3.462152
O	4.718156	4.168262	-0.013756
C	5.740835	3.869311	-0.973174
C	4.636232	5.581607	0.201203
C	6.203957	5.212685	-1.531376
H	6.556591	3.342351	-0.462622
H	5.323864	3.206660	-1.738752
C	5.932721	6.155066	-0.356122
H	3.763173	5.980289	-0.334631
H	4.495577	5.760320	1.270248
H	5.595289	5.499664	-2.395566
H	7.251072	5.192433	-1.840724
H	5.832251	7.201420	-0.652880
H	6.732992	6.078783	0.387478
Cu	-0.562571	-1.042406	-1.305780
O	-2.427905	-1.721356	-1.577403
C	-2.803768	-2.362061	-2.743003
C	-2.067622	-3.710449	-2.903882
C	-4.322594	-2.633862	-2.711109
C	-2.490294	-1.486996	-3.977674
H	-0.989813	-3.531301	-2.958617
H	-2.271655	-4.352254	-2.037905
H	-2.375255	-4.255192	-3.807758
H	-4.872986	-1.690285	-2.639062
H	-4.670679	-3.166368	-3.606808
H	-4.577894	-3.238059	-1.833125
H	-2.758068	-1.973767	-4.926159
H	-3.037245	-0.538730	-3.914393
H	-1.416853	-1.263710	-3.997378

TS2

Cu	-1.229402	-0.491988	0.175776

P	1.051530	-3.334001	-1.442132
P	1.325506	-0.049013	0.937077
C	2.977467	-0.344735	0.127809
C	5.435513	-0.666415	-1.193069
C	3.036613	-0.393724	-1.267532
C	4.167040	-0.488973	0.854411
C	5.384261	-0.649802	0.200593
C	4.259432	-0.540660	-1.925081
H	4.145160	-0.496319	1.940444
H	6.295039	-0.764416	0.780934
H	6.385318	-0.793281	-1.703568
C	2.490806	-3.531339	-2.583018
C	4.718760	-3.489052	-4.295473
C	3.695505	-4.127914	-2.200924
C	2.423825	-2.901376	-3.834962
C	3.520739	-2.893434	-4.690091
C	4.802663	-4.100434	-3.047894
H	1.503818	-2.409190	-4.142930
H	3.443693	-2.411491	-5.660406
H	5.580009	-3.474534	-4.956741
C	1.421018	-4.597874	-0.139812
C	1.827021	-6.394595	1.974806
C	1.192555	-5.966228	-0.311103
C	1.876811	-4.172199	1.108565
C	2.094201	-5.043256	2.180084
C	1.385629	-6.857012	0.737222
H	0.834081	-6.331413	-1.268450
H	1.189848	-7.914936	0.593778
H	1.965711	-7.103638	2.783489
C	1.538225	-0.939050	2.531381
C	1.774356	-2.460665	4.872703
C	1.273831	-0.369272	3.776642
C	1.887535	-2.290786	2.496820
C	2.058985	-3.061978	3.646116
C	1.375668	-1.128640	4.938153
H	0.978936	0.671588	3.839511
H	1.153766	-0.676903	5.899784
H	1.874244	-3.029015	5.790977
C	1.587314	1.719306	1.441865
C	1.983879	4.453755	1.949172
C	0.741095	2.679894	0.884370
C	2.633233	2.149852	2.273988
C	2.828739	3.502924	2.527649
C	0.939446	4.040219	1.129389
H	-0.067804	2.366893	0.236729
H	3.298021	1.425997	2.733799
H	3.639845	3.816866	3.177732
H	0.266580	4.765147	0.683998
H	2.139378	5.509999	2.146116
C	-0.313684	-4.209651	-2.330819
C	-2.540375	-5.487083	-3.450025
C	-0.180394	-4.971502	-3.494995
C	-1.577806	-4.078932	-1.737596
C	-2.678206	-4.731484	-2.286271
C	-1.291854	-5.599036	-4.056651
H	0.791395	-5.084015	-3.966438
H	-1.668892	-3.469654	-0.834331
H	-3.648695	-4.646746	-1.806025
H	-1.177146	-6.185843	-4.963576
H	-3.402800	-5.986257	-3.882219
H	2.122238	-0.305457	-1.846003
H	3.777410	-4.612879	-1.232660
H	4.280914	-0.585663	-3.009583
H	5.732018	-4.564339	-2.730227
O	2.112192	-2.832891	1.261438
C	-1.981435	-2.885939	1.894865
C	-1.901748	-2.171759	3.257568
C	-3.448574	-2.948234	1.428962
C	-1.486666	-4.332842	2.090604
H	-0.881761	-2.236228	3.649304
H	-2.147125	-1.110784	3.154316
H	-2.578495	-2.618327	3.998502
H	-3.515166	-3.513606	0.490884
H	-4.099149	-3.442773	2.162756
H	-3.849251	-1.949655	1.242929
H	-2.115744	-4.886210	2.800171
H	-1.488085	-4.871392	1.136395
H	-0.462536	-4.330882	2.472813
Br	0.399761	1.788590	-2.576355
K	3.174456	2.743728	-1.126598
C	-2.170321	1.867183	1.860340
O	-2.192921	3.066105	1.581995
O	-1.610066	1.430714	3.015784
C	-1.701804	-0.642170	-2.103606
H	-0.801387	-1.229301	-2.260761
H	-2.647352	-1.036292	-2.476610
B	-4.652476	0.270282	-0.581140
O	-5.034124	0.083335	-1.888667
O	-5.405990	-0.460862	0.299246
O	-4.464313	3.713813	-0.525238
O	-5.279214	2.597527	1.298061
B	-4.394999	2.581315	0.248416
C	-5.583175	4.493429	-0.057217
C	-5.810011	3.933733	1.394308

C	-5.974827	-1.015921	-1.904633
C	-5.173731	-2.275574	-2.226215
H	-5.818540	-3.156578	-2.293653
H	-4.669640	-2.148757	-3.188931
H	-4.411067	-2.454207	-1.462598
C	-7.019298	-0.757961	-2.978426
H	-6.548462	-0.781922	-3.965567
H	-7.792940	-1.531964	-2.952211
H	-7.494930	0.216877	-2.852230
C	-6.521698	-1.005679	-0.435464
C	-6.867052	-2.375711	0.127073
H	-7.224109	-2.271252	1.155819
H	-7.662289	-2.841779	-0.463651
H	-6.000392	-3.039069	0.135620
C	-7.691997	-0.042551	-0.236636
H	-7.875704	0.074792	0.834734
H	-7.466389	0.944752	-0.650233
H	-8.605222	-0.418010	-0.707276
C	2.646231	-4.462139	3.479745
C	2.348891	-5.357950	4.684736
H	1.272947	-5.494772	4.830111
H	2.812195	-6.340407	4.560930
H	2.771982	-4.929325	5.597086
C	4.179837	-4.316054	3.328861
H	4.608143	-3.867202	4.231449
H	4.639758	-5.297910	3.174898
H	4.436586	-3.680957	2.475090
C	-1.678889	0.552181	-1.512141
C	-3.548955	1.295145	-0.140387
C	-1.127749	2.438119	3.894061
H	-0.246731	2.939738	3.482008
H	-1.897630	3.188033	4.096488
H	-0.862644	1.925151	4.820126
C	-7.269131	3.848542	1.818956
H	-7.844421	3.198350	1.156239
H	-7.334006	3.441612	2.832397
H	-7.728650	4.842296	1.822158
C	-4.993099	4.665685	2.459192
H	-5.362791	5.682858	2.619861
H	-5.076396	4.117335	3.402463
H	-3.935892	4.698633	2.185398
C	-5.216918	5.969024	-0.117527
H	-5.070370	6.270992	-1.158880
H	-6.019847	6.582488	0.303955
H	-4.294684	6.174616	0.429473
C	-6.746427	4.203463	-1.006275
H	-7.631174	4.795693	-0.755279
H	-6.442286	4.452938	-2.026786
H	-7.018158	3.143708	-0.985979
C	-2.716583	0.805228	1.036996
H	-3.155976	0.010998	1.636785
C	-2.604540	1.682886	-1.310441
H	-2.040353	2.564986	-1.003976
H	-3.121039	1.928416	-2.245336
O	-1.176681	-2.264222	0.951046
C	3.406475	5.984092	-2.921658
C	4.363533	7.393901	-1.254641
C	3.664450	7.460308	-2.615457
H	2.463280	5.816687	-3.450551
H	4.220874	5.546161	-3.512298
H	4.287702	8.321055	-0.682441
H	5.423384	7.144144	-1.377015
H	2.718038	8.002902	-2.526046
H	4.265440	7.942449	-3.389311
C	6.155074	2.832478	0.961113
C	7.013409	4.962396	0.307139
C	6.476816	4.205637	1.537422
H	7.058051	2.207192	0.891467
H	5.387133	2.280488	1.507487
H	8.106225	4.971801	0.296807
H	6.670552	5.999718	0.285761
H	7.195377	4.160739	2.358848
H	5.557605	4.670744	1.908093
O	5.652580	3.114643	-0.339686
C	6.469271	4.150841	-0.888402
H	5.839358	4.735886	-1.563123
H	7.286994	3.704076	-1.469022
C	3.621577	6.239933	-0.597313
H	4.196239	5.719051	0.175129
H	2.673489	6.579631	-0.158387
O	3.358402	5.311721	-1.653045

I_6

Cu	-0.022342	0.787167	-0.131175
P	1.795900	1.847023	1.077602
P	1.301638	-2.015197	-0.532577
C	1.823067	-2.895291	1.007239
C	2.430623	-4.099689	3.467424
C	0.803744	-3.255465	1.899129
C	3.149486	-3.153966	1.361913
C	3.450550	-3.746347	2.588698
C	1.102828	-3.858856	3.114884
H	3.959148	-2.902480	0.683952
H	4.486954	-3.937926	2.850586
H	2.666918	-4.563645	4.420238

C	2.423145	1.480533	2.775354
C	3.562126	0.789676	5.239541
C	2.582792	2.443779	3.774791
C	2.864654	0.173163	3.015560
C	3.428510	-0.170899	4.239155
C	3.143141	2.096252	5.002435
H	2.775443	-0.582313	2.240337
H	3.756973	-1.192177	4.407253
H	3.996794	0.522648	6.198048
C	3.422038	2.152096	0.277540
C	5.951931	2.491828	-0.866221
C	4.032496	3.408013	0.264126
C	4.133057	1.071880	-0.249943
C	5.402656	1.212631	-0.814149
C	5.278730	3.581934	-0.324027
H	3.526923	4.259353	0.706354
H	5.732762	4.567122	-0.348728
H	6.927657	2.642074	-1.314525
C	2.873101	-2.020893	-1.499121
C	5.304661	-1.884468	-2.888438
C	3.147719	-2.906595	-2.543550
C	3.841295	-1.059032	-1.194724
C	5.079726	-0.999262	-1.835021
C	4.342717	-2.822772	-3.249242
H	2.413815	-3.657863	-2.815238
H	4.534618	-3.505661	-4.070546
H	6.244822	-1.855103	-3.427977
C	0.313004	-3.330360	-1.376518
C	-1.299083	-5.220397	-2.679430
C	-0.662592	-2.924232	-2.293995
C	0.474956	-4.698926	-1.126485
C	-0.326441	-5.636696	-1.771553
C	-1.462853	-3.862874	-2.941873
H	-0.812171	-1.870286	-2.508227
H	1.229011	-5.035729	-0.421445
H	-0.190454	-6.694107	-1.564745
H	-2.218029	-3.526810	-3.645911
H	-1.926816	-5.952431	-3.178811
C	0.941692	3.467586	1.166016
C	-0.725176	5.720697	1.117728
C	0.031450	3.702674	2.205284
C	0.983931	4.359468	0.087767
C	0.164464	5.484316	0.072358
C	-0.792816	4.824162	2.181865
H	-0.051942	2.998258	3.027349
H	1.623133	4.148877	-0.761210
H	0.209627	6.166542	-0.771090

H	-1.492901	4.991322	2.994651
H	-1.369077	6.594706	1.099475
H	-0.229334	-3.044405	1.633891
H	2.278187	3.470991	3.602115
H	0.299712	-4.135189	3.792203
H	3.257207	2.853356	5.772392
O	3.543844	-0.168765	-0.186631
O	0.682680	1.747440	-1.620673
C	1.079661	1.700189	-2.955010
C	0.901288	0.309919	-3.579800
C	0.256251	2.730183	-3.753855
C	2.566870	2.083004	-3.070959
H	1.492252	-0.425363	-3.026575
H	-0.148082	0.011961	-3.557001
H	1.240674	0.294052	-4.623365
H	0.360339	3.719788	-3.292592
H	0.591569	2.801209	-4.796162
H	-0.803231	2.463316	-3.749096
H	2.874449	2.184404	-4.119214
H	2.763687	3.032774	-2.561438
H	3.195276	1.316088	-2.612556
C	6.128486	-0.049665	-1.265880
C	7.247051	0.248283	-2.266350
H	6.862863	0.724549	-3.173553
H	7.998929	0.903786	-1.819366
H	7.765280	-0.672093	-2.547879
C	6.742409	-0.720307	-0.012389
H	7.232965	-1.659492	-0.288560
H	7.485178	-0.057713	0.443693
H	5.976523	-0.939872	0.738217
C	-0.919984	0.466344	1.577828
C	-0.399611	-0.286372	2.536394
H	-0.982198	-0.496337	3.435932
H	0.577356	-0.744533	2.480094
C	-1.888836	0.230309	-0.710063
H	-1.640057	-0.806107	-0.929188
C	-2.225481	0.859124	-2.033337
O	-2.318501	0.189147	-3.044584
O	-2.553228	2.161277	-2.126048
C	-2.090125	3.127699	-1.178208
H	-2.254219	4.096523	-1.653341
H	-2.671330	3.092912	-0.254801
H	-1.022857	2.987422	-1.004604
C	-2.981498	0.345238	0.389037
B	-3.399475	-1.118882	0.830277
B	-4.330184	1.021670	-0.069810
O	-2.561458	-2.202182	0.853499

O	-4.673818	-1.431283	1.236294
C	-3.273836	-3.281310	1.503394
C	-4.763856	-2.876648	1.254091
C	-2.903582	-3.245697	2.985935
H	-3.363395	-4.077767	3.526794
H	-1.820065	-3.326342	3.095425
H	-3.221580	-2.308634	3.452893
C	-2.842941	-4.594350	0.871508
H	-2.931960	-4.561543	-0.216146
H	-1.796783	-4.799006	1.117555
H	-3.450305	-5.421348	1.253294
C	-5.739903	-3.299676	2.339055
H	-6.750114	-2.975032	2.072631
H	-5.749400	-4.389556	2.439992
H	-5.485177	-2.860540	3.305635
C	-5.273614	-3.308297	-0.120368
H	-5.427232	-4.390341	-0.163996
H	-6.229792	-2.815449	-0.315626
H	-4.575467	-3.022486	-0.913111
O	-4.917986	2.072453	0.584631
O	-5.061945	0.572841	-1.136107
C	-6.355024	1.211067	-1.067808
C	-7.295935	0.229148	-0.369293
H	-6.938491	-0.015540	0.634411
H	-8.312617	0.627384	-0.303075
H	-7.327979	-0.697937	-0.948675
C	-6.843139	1.491060	-2.480949
H	-6.106579	2.053853	-3.057382
H	-7.028262	0.546008	-2.999749
H	-7.780830	2.055663	-2.458442
C	-6.051695	2.492771	-0.213235
C	-7.174633	2.918619	0.719175
H	-6.876533	3.814451	1.272022
H	-8.076603	3.157993	0.147040
H	-7.415660	2.136298	1.441799
C	-5.586012	3.676906	-1.059358
H	-6.414567	4.107762	-1.628790
H	-5.183906	4.450860	-0.399011
H	-4.800458	3.376706	-1.757719
C	-2.304357	1.033864	1.596738
H	-2.800509	0.852560	2.562055
H	-2.283340	2.118533	1.449613

(THF)$_2$·KBr

Br	-0.364684	0.515570	0.200774
K	2.467304	-0.068137	-0.850781
O	4.395162	1.653506	-1.333387
C	4.969875	2.212221	-0.144381
C	5.181673	2.018058	-2.477573
C	6.402573	2.569088	-0.513639
H	4.402812	3.104748	0.152161
H	4.892428	1.466261	0.652793
C	6.238572	2.998971	-1.973594
H	5.642060	1.110510	-2.889526
H	4.523510	2.449984	-3.237612
H	7.046242	1.685088	-0.443308
H	6.815719	3.351644	0.126253
H	7.163876	2.940767	-2.550678
H	5.862913	4.025889	-2.025236
O	4.432313	-1.152538	0.515141
C	4.486257	-1.288647	1.944204
C	5.755111	-1.200321	-0.034020
C	5.935055	-1.630744	2.282645
H	4.183247	-0.336996	2.400417
H	3.777076	-2.062405	2.252557
C	6.695715	-0.956206	1.138336
H	5.928590	-2.188667	-0.480976
H	5.823153	-0.441883	-0.819837
H	6.089234	-2.714250	2.253085
H	6.224736	-1.267635	3.270968
H	7.689831	-1.375903	0.969900
H	6.798838	0.117994	1.328129

TS3

Cu	-0.059405	-0.554496	-0.271928
P	-1.661367	-1.694827	1.246076
P	-1.642479	1.996173	-0.579041
C	-2.216472	3.012676	0.863753
C	-2.930127	4.405405	3.200516
C	-1.266701	3.761135	1.575472
C	-3.530725	2.983346	1.340297
C	-3.883220	3.672767	2.498838
C	-1.618498	4.450137	2.731321
H	-4.296447	2.427128	0.810671
H	-4.909793	3.635041	2.850548
H	-3.207177	4.941958	4.102818
C	-2.197677	-1.151055	2.925385
C	-3.134848	-0.215114	5.391338
C	-2.681872	-2.047397	3.887139
C	-2.204378	0.216031	3.208417
C	-2.671005	0.683552	4.436272
C	-3.142140	-1.582706	5.113965
H	-1.857045	0.922125	2.463851
H	-2.672123	1.750440	4.637163
H	-3.496586	0.146260	6.349373
C	-3.272439	-2.282255	0.582473

C	-5.750254	-3.009773	-0.499681
C	-3.709472	-3.607080	0.636390
C	-4.122953	-1.338248	0.003528
C	-5.367135	-1.670302	-0.534293
C	-4.933164	-3.971232	0.086801
H	-3.082089	-4.364427	1.093766
H	-5.254386	-5.007302	0.120226
H	-6.702686	-3.311175	-0.921303
C	-3.219932	1.753173	-1.503604
C	-5.630905	1.244240	-2.838472
C	-3.591158	2.510873	-2.616958
C	-4.080804	0.728846	-1.098843
C	-5.308300	0.481783	-1.717369
C	-4.776058	2.244257	-3.292255
H	-2.938639	3.302896	-2.968512
H	-5.043227	2.828781	-4.166669
H	-6.563990	1.068321	-3.362047
C	-0.760605	3.267321	-1.583960
C	0.741488	5.088881	-3.093006
C	0.299071	2.826521	-2.382358
C	-1.068536	4.634371	-1.560462
C	-0.321249	5.538160	-2.308984
C	1.046694	3.731795	-3.133051
H	0.554722	1.772592	-2.425495
H	-1.890589	4.994769	-0.949220
H	-0.567467	6.595401	-2.279235
H	1.871491	3.370597	-3.739191
H	1.328110	5.797158	-3.670529
C	-0.692376	-3.227881	1.549988
C	1.069984	-5.393193	1.814032
C	0.054660	-3.397326	2.721012
C	-0.528479	-4.145336	0.503191
C	0.336959	-5.225860	0.640189
C	0.931003	-4.473212	2.849932
H	-0.036374	-2.685961	3.536044
H	-1.045184	-3.971610	-0.436220
H	0.450232	-5.929018	-0.179671
H	1.505164	-4.589662	3.764162
H	1.751015	-6.232525	1.917499
H	-0.241907	3.819786	1.217973
H	-2.699810	-3.112973	3.675557
H	-0.865856	5.025455	3.262097
H	-3.513204	-2.286614	5.852849
O	-3.691660	-0.031505	-0.020275
O	-0.818786	-1.703822	-1.642992
C	-1.241565	-1.781741	-2.961134
C	-1.135316	-0.431429	-3.687234
C	-0.389081	-2.821305	-3.718169
C	-2.712357	-2.239079	-3.003296
H	-1.731051	0.320234	-3.160123
H	-0.095171	-0.097439	-3.714811
H	-1.506002	-0.490463	-4.719047
H	-0.448206	-3.791428	-3.209443
H	-0.727381	-2.958918	-4.753175
H	0.659209	-2.509471	-3.736748
H	-3.055456	-2.434568	-4.027572
H	-2.842585	-3.153384	-2.412784
H	-3.358087	-1.467782	-2.575492
C	-6.239150	-0.539336	-1.069733
C	-7.310239	-1.042442	-2.039971
H	-6.867559	-1.534740	-2.911319
H	-7.979648	-1.748915	-1.542475
H	-7.934939	-0.214776	-2.386091
C	-6.933726	0.144055	0.132913
H	-7.534031	0.993284	-0.209758
H	-7.592050	-0.567702	0.641640
H	-6.203168	0.510786	0.860683
C	1.200675	-0.108615	1.204011
C	0.803484	0.925942	1.943159
H	1.205966	1.045411	2.949011
H	0.077581	1.653133	1.610591
C	1.986064	0.035349	-0.627583
H	1.856473	1.093346	-0.833785
C	2.111542	-0.607112	-2.002540
O	2.108290	0.096077	-2.993385
O	2.437762	-1.898908	-2.158352
C	1.981406	-2.921460	-1.263045
H	2.133473	-3.858505	-1.801384
H	2.575572	-2.947661	-0.349685
H	0.915829	-2.778252	-1.081911
C	3.193347	-0.251217	0.325841
B	3.752476	1.157567	0.785100
B	4.440972	-1.033422	-0.241628
O	3.633122	2.289043	0.026389
O	4.398477	1.366721	1.972225
C	4.019351	3.407384	0.854347
C	4.922799	2.718376	1.939475
C	2.728033	4.001285	1.418694
H	2.924534	4.903339	2.005223
H	2.073076	4.267949	0.583372
H	2.199987	3.279597	2.048450
C	4.729311	4.436480	-0.011627
H	5.567996	3.997444	-0.555285
H	4.025792	4.844803	-0.743188

H	5.101263	5.262711	0.602398
C	4.814863	3.319870	3.331325
H	5.472710	2.781093	4.019470
H	5.123958	4.369751	3.317815
H	3.795230	3.260056	3.717712
C	6.386492	2.609973	1.516547
H	6.878243	3.586765	1.525394
H	6.911852	1.954471	2.216831
H	6.477261	2.181501	0.514337
O	4.876698	-2.225359	0.268040
O	5.262820	-0.520845	-1.208318
C	6.463408	-1.330994	-1.220348
C	7.495848	-0.595021	-0.367105
H	7.151721	-0.481362	0.665424
H	8.454881	-1.120740	-0.360137
H	7.655452	0.402749	-0.785124
C	6.957971	-1.459191	-2.652134
H	6.175732	-1.830440	-3.316787
H	7.280129	-0.480643	-3.019527
H	7.814185	-2.139412	-2.700723
C	5.971265	-2.672811	-0.571586
C	6.999759	-3.375004	0.300571
H	6.571239	-4.295812	0.706877
H	7.881262	-3.642903	-0.290301
H	7.315752	-2.748536	1.137079
C	5.383959	-3.649719	-1.588638
H	6.167501	-4.086426	-2.214300
H	4.880756	-4.460256	-1.053657
H	4.651990	-3.157467	-2.234368
C	2.422974	-0.919115	1.489156
H	2.829145	-0.763378	2.493675
H	2.275854	-1.988772	1.329795

24

C	0.309887	0.875895	-0.677267
C	0.583518	1.492322	-2.087575
C	1.089884	2.048929	-0.016245
H	-0.284827	1.663493	-2.731280
H	1.336442	0.943150	-2.662577
H	0.575337	2.620382	0.759885
B	0.805698	-0.594350	-0.414179
B	-1.198126	0.792395	-0.261320
O	0.658566	-1.189536	0.816288
O	1.177453	-1.472612	-1.397107
O	-1.728618	1.366489	0.862788
O	-2.105108	0.040768	-0.964745
C	1.170429	-2.535043	0.703010
C	1.062752	-2.801929	-0.840352
C	2.166651	-3.677213	-1.412431
H	2.150464	-4.667527	-0.946023
H	2.015806	-3.806844	-2.488186
H	3.152148	-3.233393	-1.257858
C	-0.308160	-3.331403	-1.263342
H	-0.370109	-3.319936	-2.355202
H	-0.468086	-4.357644	-0.920389
H	-1.111780	-2.697297	-0.876307
C	2.610489	-2.515207	1.213676
H	2.614115	-2.144936	2.243028
H	3.049636	-3.517101	1.207618
H	3.232095	-1.845752	0.614591
C	0.318750	-3.453643	1.566392
H	0.633861	-4.495149	1.445794
H	0.437087	-3.183156	2.619787
H	-0.740976	-3.378711	1.312861
C	-3.399660	0.248784	-0.353523
C	-3.013315	0.736407	1.086492
C	-4.113584	1.317559	-1.179787
H	-4.170184	0.983312	-2.219528
H	-5.131210	1.489496	-0.817854
H	-3.569957	2.266959	-1.156898
C	-4.176325	-1.057890	-0.395302
H	-5.130510	-0.953102	0.130815
H	-4.388803	-1.328305	-1.433687
H	-3.613972	-1.874965	0.061534
C	-3.967009	1.753326	1.692230
H	-4.968420	1.323169	1.794027
H	-3.618126	2.039709	2.688655
H	-4.034816	2.656590	1.082517
C	-2.767897	-0.413436	2.063555
H	-2.329671	-0.010815	2.981019
H	-3.698704	-0.925920	2.322400
H	-2.063371	-1.140555	1.648494
C	1.142715	2.716565	-1.393628
C	1.493227	3.936751	-1.775794
H	1.852753	4.670524	-1.059097
H	1.427432	4.241067	-2.817429
C	2.481114	1.695780	0.444613
O	3.075591	0.680606	0.143011
O	3.015164	2.666751	1.188011
C	4.369402	2.460632	1.604403
H	4.629755	3.330311	2.206832
H	4.454871	1.549249	2.200652
H	5.032265	2.390641	0.738582

I_5-B

Cu	0.405470	0.086715	0.120961

P	1.761284	1.983409	0.131716
P	1.701320	-1.847474	0.695104
C	2.614687	-1.602445	2.267193
C	3.855896	-1.179870	4.732456
C	1.948393	-0.905216	3.281710
C	3.909377	-2.076191	2.491867
C	4.529401	-1.858847	3.720312
C	2.563498	-0.706780	4.512985
H	4.435356	-2.616077	1.709714
H	5.537987	-2.225154	3.886739
H	4.340151	-1.012570	5.689876
C	2.661111	2.216306	1.723783
C	3.854883	2.514777	4.238984
C	1.912758	2.667809	2.818376
C	4.009586	1.905325	1.905669
C	4.600470	2.051727	3.159204
C	2.506993	2.823650	4.065181
H	4.611872	1.555352	1.074589
H	5.649718	1.804004	3.287440
H	4.319948	2.630177	5.213231
C	3.052185	2.038228	-1.184554
C	4.887495	1.866470	-3.302075
C	3.206599	3.100027	-2.080145
C	3.840265	0.903664	-1.393496
C	4.771472	0.788814	-2.425858
C	4.113062	3.010551	-3.130761
H	2.603385	3.994169	-1.970847
H	4.215971	3.839770	-3.823076
H	5.590181	1.822027	-4.126792
C	2.987089	-2.389096	-0.498651
C	4.848887	-2.945789	-2.515882
C	3.126701	-3.688286	-0.989567
C	3.816482	-1.401682	-1.031193
C	4.752668	-1.640081	-2.034506
C	4.049865	-3.960574	-1.994653
H	2.500517	-4.483860	-0.599060
H	4.147059	-4.971509	-2.377057
H	5.558060	-3.182800	-3.301422
C	0.742077	-3.389943	0.973750
C	-0.783538	-5.716398	1.261433
C	-0.311344	-3.659646	0.091645
C	1.018787	-4.291941	2.005136
C	0.254084	-5.446810	2.150978
C	-1.061648	-4.823677	0.228667
H	-0.551323	-2.956156	-0.702513
H	1.831255	-4.094300	2.697977
H	0.472755	-6.138479	2.958845

H	-1.867653	-5.030542	-0.468477
H	-1.376591	-6.618901	1.374312
C	0.890360	3.585049	-0.059252
C	-0.503946	5.969814	-0.471549
C	1.502235	4.816632	0.213804
C	-0.429276	3.559816	-0.510889
C	-1.119844	4.749867	-0.728281
C	0.806563	6.002467	0.009694
H	2.524028	4.844537	0.582261
H	-0.937008	2.615261	-0.679039
H	-2.140905	4.698387	-1.088125
H	1.286078	6.953208	0.222189
H	-1.043416	6.897807	-0.636777
H	0.947226	-0.517452	3.104691
H	0.862144	2.913834	2.691635
H	2.040599	-0.165948	5.295483
H	1.915563	3.184398	4.901421
O	3.668446	-0.137646	-0.514169
C	5.634159	-0.472585	-2.474151
C	6.243100	-0.700406	-3.859562
H	5.471837	-0.815435	-4.627197
H	6.890189	0.135532	-4.137883
H	6.872029	-1.594374	-3.860698
C	6.776589	-0.315535	-1.442264
H	7.400903	-1.214857	-1.432858
H	7.404030	0.543190	-1.701822
H	6.385443	-0.161854	-0.431753
C	-1.623735	-0.256352	0.548085
C	-2.327078	0.183304	-1.940860
C	-2.753336	-0.057567	-0.475737
Br	0.406481	-0.518363	-2.708452
C	-1.492489	-0.908835	-2.564442
C	-1.916201	-2.082605	-3.019641
H	-2.979972	-2.293694	-2.960047
H	-1.261453	-2.832431	-3.449158
H	-1.404409	-1.267831	0.895405
C	-1.870094	0.642079	1.597496
O	-2.747441	1.563844	1.451022
O	-1.227361	0.563619	2.763780
C	-1.530344	1.553039	3.747547
H	-1.382794	2.560876	3.352547
H	-2.560815	1.450757	4.097332
H	-0.838719	1.369280	4.570084
H	-3.230600	0.272778	-2.554906
H	-1.801899	1.136816	-2.032961
B	-3.555422	1.291541	0.099638
O	-3.512099	2.471090	-0.728210

O	-4.945404	1.104660	0.477363
C	-4.723134	3.189727	-0.521812
C	-5.740233	2.035202	-0.239568
C	-5.031212	4.015243	-1.765866
H	-4.974119	3.404281	-2.669617
H	-6.031513	4.458071	-1.703998
H	-4.311285	4.834659	-1.866441
C	-4.568633	4.121168	0.688233
H	-4.400310	3.549049	1.603891
H	-3.703424	4.772782	0.536579
H	-5.451504	4.754605	0.825039
C	-5.176697	-2.696285	0.695390
C	-5.410545	-2.746416	-0.856981
C	-6.236584	-1.880421	1.437132
H	-7.211526	-2.377187	1.426254
H	-5.922615	-1.763499	2.478774
H	-6.321790	-0.880009	1.004306
C	-5.001478	-4.056870	1.355648
H	-4.840750	-3.925835	2.429883
H	-5.896610	-4.672662	1.219172
H	-4.139876	-4.592406	0.950443
C	-6.868576	-2.653265	-1.283522
H	-6.933914	-2.653292	-2.375774
H	-7.434358	-3.512833	-0.909174
H	-7.339822	-1.740230	-0.915251
C	-4.754237	-3.956563	-1.523208
H	-5.248528	-4.891108	-1.242327
H	-4.822590	-3.849369	-2.610110
H	-3.696426	-4.023514	-1.253645
B	-3.806908	-1.203582	-0.340513
O	-3.941032	-1.969234	0.798053
O	-4.709998	-1.576242	-1.318089
C	-6.235691	1.374458	-1.530977
H	-6.752186	0.444902	-1.278264
H	-6.928628	2.020157	-2.081286
H	-5.398099	1.115265	-2.183550
C	-6.938696	2.433724	0.615936
H	-7.512867	3.238117	0.142475
H	-7.606321	1.574587	0.741870
H	-6.626570	2.764211	1.609124

TS2B

Cu	-0.139934	-0.022979	-0.643210
P	-1.609382	-1.871216	0.389230
P	-1.555149	2.037842	0.312487
C	-2.343411	1.748518	1.952672
C	-3.333921	1.390328	4.543070
C	-1.526004	1.218176	2.956023
C	-3.668150	2.076237	2.252618
C	-4.160833	1.895053	3.542238
C	-2.016761	1.048293	4.247025
H	-4.318814	2.479235	1.481527
H	-5.192115	2.152082	3.765413
H	-3.719977	1.249194	5.548072
C	-2.338136	-2.025740	2.083438
C	-3.381327	-2.285813	4.676843
C	-1.552483	-2.531954	3.123873
C	-3.653021	-1.640612	2.363724
C	-4.169655	-1.775452	3.649406
C	-2.067222	-2.660841	4.410027
H	-4.289956	-1.239258	1.584124
H	-5.194302	-1.475110	3.845568
H	-3.788316	-2.391207	5.677854
C	-3.086862	-2.147167	-0.685511
C	-5.262403	-2.332550	-2.446499
C	-3.392771	-3.356643	-1.309811
C	-3.900198	-1.050753	-0.975664
C	-4.995357	-1.108425	-1.835721
C	-4.470265	-3.446367	-2.184867
H	-2.776072	-4.230797	-1.134960
H	-4.691668	-4.391738	-2.669706
H	-6.097045	-2.425354	-3.132872
C	-2.975039	2.359855	-0.806555
C	-5.087076	2.540759	-2.640189
C	-3.190483	3.539302	-1.521626
C	-3.833889	1.287206	-1.054239
C	-4.910518	1.350073	-1.934972
C	-4.232428	3.621734	-2.441336
H	-2.537875	4.392581	-1.369136
H	-4.386421	4.539860	-2.999117
H	-5.903231	2.634307	-3.348448
C	-0.849009	3.723497	0.589795
C	0.266266	6.256572	1.024807
C	0.488173	3.973316	0.268791
C	-1.616066	4.754844	1.149448
C	-1.064567	6.012513	1.363100
C	1.039639	5.236844	0.480927
H	1.103338	3.183313	-0.151347
H	-2.652141	4.572821	1.420660
H	-1.671728	6.802091	1.795272
H	2.076002	5.423712	0.216868
H	0.698507	7.238931	1.189422
C	-0.663802	-3.438166	0.213971
C	0.813149	-5.775327	-0.223938
C	-1.109633	-4.666204	0.724948

C	0.537086	-3.397140	-0.496997
C	1.268419	-4.560248	-0.723234
C	-0.374582	-5.825906	0.507603
H	-2.035162	-4.714659	1.291134
H	0.918097	-2.454817	-0.877443
H	2.199943	-4.490266	-1.273329
H	-0.730156	-6.771317	0.905885
H	1.384023	-6.683190	-0.395362
H	-0.505239	0.924322	2.726709
H	-0.540674	-2.862765	2.922737
H	-1.373822	0.634141	5.017526
H	-1.442009	-3.067540	5.199434
O	-3.575513	0.132622	-0.358243
C	-5.850375	0.147916	-1.993721
C	-6.677572	0.124804	-3.280871
H	-6.041843	0.063062	-4.169285
H	-7.363368	-0.726478	-3.282556
H	-7.296152	1.022950	-3.356150
C	-6.809606	0.233686	-0.782132
H	-7.419464	1.140853	-0.846514
H	-7.475613	-0.635190	-0.765468
H	-6.258118	0.260326	0.163102
C	1.661617	0.170023	0.428045
C	2.357656	-0.028949	-1.976118
C	2.861542	0.082969	-0.523601
Br	-1.026647	-0.512986	-2.862140
C	1.136120	0.806287	-2.047678
C	0.913013	2.044194	-2.442101
H	1.767381	2.631129	-2.782749
H	-0.062040	2.517204	-2.440166
H	1.425540	1.139613	0.871515
C	1.901613	-0.805852	1.453283
O	2.850049	-1.628513	1.318092
O	1.185510	-0.816548	2.559995
C	1.648790	-1.660054	3.625271
H	1.671118	-2.706054	3.314178
H	2.648503	-1.350990	3.938360
H	0.938546	-1.518026	4.438185
H	3.072826	0.332596	-2.727630
H	2.139180	-1.071062	-2.225000
B	3.706630	-1.249210	-0.040678
O	3.694382	-2.378002	-0.916499
O	5.063887	-1.047658	0.394018
C	4.930028	-3.070760	-0.746527
C	5.903237	-1.903308	-0.372988
C	5.268512	-3.795845	-2.042709
H	6.273020	-4.230495	-1.997624
H	4.557434	-4.611592	-2.211985
H	5.216881	-3.120825	-2.899907
C	4.790790	-4.087710	0.392983
H	4.588362	-3.588359	1.343784
H	3.953284	-4.757873	0.180624
H	5.694808	-4.695926	0.500718
C	5.101547	2.835646	0.730889
C	5.238189	3.006208	-0.821888
C	6.260264	2.054473	1.351651
H	7.190311	2.630296	1.332417
H	6.015325	1.832140	2.394406
H	6.404857	1.101187	0.836116
C	4.867438	4.129428	1.495213
H	4.795313	3.918682	2.566320
H	5.699423	4.824301	1.341282
H	3.940715	4.616470	1.185445
C	6.668796	3.111596	-1.327123
H	6.670460	3.212871	-2.416495
H	7.162540	3.992048	-0.903008
H	7.251591	2.225475	-1.067581
C	4.395081	4.156169	-1.374381
H	4.792931	5.131730	-1.079669
H	4.391938	4.102417	-2.466984
H	3.359607	4.080051	-1.028826
B	3.823136	1.296510	-0.334873
O	3.927982	2.005375	0.841164
O	4.651392	1.787617	-1.318586
C	6.383546	-1.131630	-1.607019
H	6.869863	-0.208042	-1.280450
H	7.100172	-1.711247	-2.198415
H	5.541752	-0.850878	-2.245651
C	7.102935	-2.316629	0.471326
H	7.711846	-3.063586	-0.049843
H	7.736003	-1.445159	0.668484
H	6.790114	-2.729206	1.433061

I_6-B

Cu	-0.046103	-0.286051	-1.078213
P	-1.067866	-2.043234	0.313631
P	-1.641117	2.110712	0.212411
C	-2.370519	2.197274	1.919754
C	-3.280087	2.290928	4.577714
C	-1.492503	2.494493	2.969501
C	-3.715699	1.967920	2.222336
C	-4.165561	2.009605	3.541442
C	-1.940734	2.541370	4.285066
H	-4.432574	1.767942	1.433146
H	-5.215202	1.827969	3.753663

H	-3.631269	2.322676	5.604685
C	-1.206001	-2.012054	2.155087
C	-1.498748	-1.968148	4.943834
C	-0.260304	-2.632087	2.979087
C	-2.279122	-1.334755	2.748239
C	-2.422006	-1.311641	4.131885
C	-0.416355	-2.621983	4.363266
H	-3.011021	-0.818936	2.136338
H	-3.260724	-0.781654	4.571736
H	-1.618981	-1.961440	6.022888
C	-2.792069	-2.459896	-0.218747
C	-5.318454	-2.999891	-1.307716
C	-3.170222	-3.765673	-0.536080
C	-3.727189	-1.444195	-0.442329
C	-4.988479	-1.684038	-0.990098
C	-4.422933	-4.035674	-1.072179
H	-2.473142	-4.582417	-0.395570
H	-4.695454	-5.056679	-1.318723
H	-6.286623	-3.225672	-1.740241
C	-3.160757	2.065079	-0.825635
C	-5.551084	1.793604	-2.260392
C	-3.634412	3.111816	-1.617747
C	-3.887510	0.871168	-0.828319
C	-5.116123	0.729729	-1.469920
C	-4.804999	2.964323	-2.355615
H	-3.086342	4.047090	-1.659874
H	-5.155813	3.779135	-2.980859
H	-6.488832	1.719695	-2.799783
C	-1.080433	3.867666	0.046601
C	-0.125613	6.495131	-0.183247
C	0.159879	4.126123	-0.544541
C	-1.833205	4.946703	0.531494
C	-1.361798	6.249591	0.414904
C	0.633508	5.432662	-0.662625
H	0.760542	3.305226	-0.924259
H	-2.793493	4.763523	1.005550
H	-1.957367	7.074898	0.793414
H	1.598042	5.615345	-1.127794
H	0.243565	7.512525	-0.271386
C	-0.147893	-3.591286	-0.071677
C	1.164166	-5.930375	-0.884063
C	-0.280436	-4.776251	0.667415
C	0.658676	-3.595751	-1.211765
C	1.304536	-4.759573	-1.621017
C	0.375188	-5.934942	0.266503
H	-0.908519	-4.799654	1.552361
H	0.775829	-2.691427	-1.799014
H	1.926028	-4.741417	-2.510310
H	0.262277	-6.844781	0.847846
H	1.671113	-6.837389	-1.199406
H	-0.447459	2.692499	2.752922
H	0.603288	-3.123629	2.547395
H	-1.240884	2.771961	5.082871
H	0.319602	-3.121095	4.986339
O	-3.361082	-0.167065	-0.094727
C	-5.947789	-0.508817	-1.150917
C	-7.018145	-0.775156	-2.212763
H	-6.574968	-0.954965	-3.196837
H	-7.627735	-1.640563	-1.940665
H	-7.703413	0.073134	-2.288077
C	-6.644009	-0.268627	0.210820
H	-7.305534	0.602012	0.151120
H	-7.240608	-1.143731	0.489061
H	-5.912685	-0.090002	1.005811
C	1.592353	0.428993	-0.041954
C	2.474830	-0.035215	-2.283794
C	2.903129	0.149070	-0.807244
Br	-1.469539	-0.882871	-2.985749
C	1.157608	0.652189	-2.338740
C	0.787143	1.746571	-2.979062
H	1.497082	2.242317	-3.643728
H	-0.207623	2.173001	-2.902616
H	1.347360	1.486129	0.014059
C	1.577772	-0.110911	1.343607
O	2.152840	-1.129100	1.690427
O	0.927884	0.694272	2.188213
C	0.962403	0.393745	3.584857
H	1.327096	1.287398	4.096781
H	-0.051241	0.166231	3.914931
H	1.624415	-0.448408	3.781187
H	3.171438	0.385057	-3.020203
H	2.360310	-1.102029	-2.507874
B	3.741294	-1.095933	-0.288232
O	3.596340	-2.366937	-0.779787
O	4.864637	-0.953721	0.493788
C	4.523780	-3.211760	-0.063396
C	5.616097	-2.181879	0.396645
C	5.029388	-4.293748	-1.006021
H	5.822035	-4.878071	-0.527493
H	4.210393	-4.974519	-1.255207
H	5.418123	-3.871940	-1.935359
C	3.767487	-3.847764	1.103211
H	3.401786	-3.088816	1.796280
H	2.903586	-4.389768	0.709124

H	4.402863	-4.557317	1.642178
C	4.824019	3.205519	0.321153
C	5.110633	3.187703	-1.219461
C	5.982149	2.656231	1.152978
H	6.833536	3.342846	1.158379
H	5.643084	2.519425	2.183646
H	6.310546	1.683438	0.776178
C	4.361975	4.547200	0.867522
H	4.199780	4.471258	1.946664
H	5.120467	5.316513	0.690997
H	3.423292	4.864517	0.407802
C	6.565142	3.409341	-1.600486
H	6.672425	3.379596	-2.688730
H	6.906092	4.389499	-1.252103
H	7.212895	2.641217	-1.173029
C	4.200731	4.131589	-2.007036
H	4.453170	5.180376	-1.826013
H	4.316701	3.928569	-3.075440
H	3.149404	3.971863	-1.745991
B	3.816505	1.422206	-0.647367
O	3.739612	2.258891	0.435393
O	4.723018	1.843520	-1.587007
C	6.701144	-1.948637	-0.655350
H	7.307200	-1.088523	-0.356306
H	7.360618	-2.815762	-0.753849
H	6.262944	-1.728890	-1.633591
C	6.248701	-2.483698	1.746276
H	6.760784	-3.451051	1.723477
H	6.988268	-1.714112	1.987231
H	5.502666	-2.500146	2.543102

TS3B

Cu	-0.149045	-0.186481	-1.008430
P	-1.128836	-2.018124	0.323141
P	-1.666024	2.142904	0.231900
C	-2.439724	2.165108	1.922290
C	-3.420733	2.209075	4.555131
C	-1.588565	2.430724	3.001484
C	-3.795087	1.944676	2.181841
C	-4.280384	1.961909	3.488766
C	-2.071224	2.450754	4.305481
H	-4.492332	1.774018	1.368782
H	-5.337462	1.788370	3.668082
H	-3.799826	2.221228	5.572537
C	-1.288669	-2.029362	2.163013
C	-1.570289	-1.953081	4.953007
C	-0.344564	-2.647028	2.990751
C	-2.361304	-1.348945	2.752886
C	-2.499130	-1.309903	4.137023
C	-0.493233	-2.619053	4.375229
H	-3.094494	-0.838374	2.137366
H	-3.336657	-0.775918	4.574143
H	-1.683888	-1.930203	6.032605
C	-2.848057	-2.428203	-0.232440
C	-5.356076	-2.945140	-1.374075
C	-3.238929	-3.732212	-0.540832
C	-3.764458	-1.403227	-0.486437
C	-5.014720	-1.630446	-1.063738
C	-4.482631	-3.991116	-1.102637
H	-2.556849	-4.556932	-0.374478
H	-4.764912	-5.011099	-1.342611
H	-6.316501	-3.162220	-1.827870
C	-3.153384	2.102759	-0.852204
C	-5.497963	1.850319	-2.363152
C	-3.591695	3.152536	-1.660745
C	-3.887622	0.913501	-0.880437
C	-5.098296	0.783700	-1.557957
C	-4.738264	3.013752	-2.436785
H	-3.035475	4.083504	-1.686022
H	-5.060871	3.830566	-3.074433
H	-6.419235	1.784901	-2.931312
C	-1.145986	3.919969	0.154562
C	-0.273397	6.585899	0.103681
C	0.134140	4.242903	-0.303450
C	-1.981491	4.955106	0.599494
C	-1.551548	6.276483	0.570144
C	0.568267	5.568083	-0.331136
H	0.801923	3.461513	-0.649753
H	-2.974235	4.722132	0.974522
H	-2.211648	7.066429	0.915590
H	1.566013	5.799589	-0.691755
H	0.063628	7.617958	0.084875
C	-0.209018	-3.560229	-0.090856
C	1.087653	-5.888789	-0.960262
C	-0.334133	-4.757259	0.628902
C	0.584692	-3.546677	-1.240265
C	1.223412	-4.704463	-1.677039
C	0.312696	-5.911309	0.199217
H	-0.951702	-4.795451	1.520782
H	0.685386	-2.633636	-1.816575
H	1.833765	-4.672624	-2.573850
H	0.203590	-6.831081	0.765584
H	1.587187	-6.791710	-1.298486
H	-0.538466	2.632450	2.815795
H	0.515872	-3.144552	2.559882

H	-1.391355	2.656460	5.126946
H	0.243622	-3.113180	5.001365
O	-3.390428	-0.128829	-0.135212
C	-5.953498	-0.444451	-1.261121
C	-6.991703	-0.702943	-2.356370
H	-6.518572	-0.888353	-3.325305
H	-7.617021	-1.562677	-2.102801
H	-7.667056	0.150952	-2.454663
C	-6.691100	-0.191277	0.076310
H	-7.335928	0.690113	-0.005052
H	-7.310835	-1.056412	0.334539
H	-5.983682	-0.025294	0.895315
C	1.641424	0.599870	-0.124681
C	2.442165	-0.023239	-2.284211
C	2.951953	0.182881	-0.840124
Br	-1.452486	-0.883420	-3.034565
C	1.184694	0.760961	-2.149758
C	0.796914	1.873034	-2.753199
H	1.367042	2.234525	-3.609336
H	-0.103739	2.415434	-2.488098
H	1.536791	1.670949	0.012047
C	1.484458	-0.028980	1.227735
O	1.861141	-1.151593	1.512396
O	0.998582	0.835275	2.119714
C	0.940738	0.423547	3.490103
H	1.644964	-0.387146	3.675139
H	1.204123	1.299809	4.084440
H	-0.074067	0.104732	3.729170
H	3.089534	0.353622	-3.083979
H	2.238117	-1.081579	-2.476723
B	3.704536	-1.085741	-0.251769
O	3.618570	-2.345006	-0.778539
O	4.715019	-0.959961	0.674262
C	4.455952	-3.202471	0.032064
C	5.485175	-2.180999	0.631322
C	5.063626	-4.271748	-0.862495
H	5.785332	-4.875279	-0.302598
H	4.274038	-4.936738	-1.224749
H	5.567829	-3.836464	-1.727916
C	3.571942	-3.849039	1.098191
H	3.128560	-3.096039	1.751770
H	2.760933	-4.389759	0.605654
H	4.145110	-4.560748	1.700239
C	5.194810	3.044199	0.171856
C	5.589206	2.846593	-1.332313
C	6.171350	2.378170	1.140715
H	7.129536	2.904699	1.169231
H	5.739002	2.394823	2.145140
H	6.343185	1.332875	0.868109
C	4.939274	4.485934	0.580381
H	4.669659	4.527820	1.639630
H	5.840200	5.090305	0.434547
H	4.123526	4.928673	0.005158
C	7.084837	2.781880	-1.595617
H	7.267298	2.644883	-2.665460
H	7.568803	3.713233	-1.284844
H	7.549562	1.950385	-1.061723
C	4.924978	3.861425	-2.262814
H	5.355277	4.859554	-2.141493
H	5.076064	3.545198	-3.298754
H	3.846988	3.919517	-2.080472
B	3.985347	1.364513	-0.738606
O	3.954917	2.304543	0.257681
O	5.001987	1.559318	-1.635920
C	6.675843	-1.917763	-0.291207
H	7.243775	-1.065863	0.094275
H	7.344967	-2.781809	-0.339123
H	6.346451	-1.673318	-1.305463
C	5.969920	-2.509823	2.034438
H	6.487423	-3.474428	2.046117
H	6.674347	-1.742833	2.370269
H	5.142442	-2.546812	2.745516

I_7

Cu	0.075477	-1.441602	-0.763662
P	-1.931652	-0.535711	0.053730
P	1.981952	-0.341405	0.056948
C	-3.495639	-1.472838	-0.153183
C	-4.619071	-1.249186	0.650959
C	-3.555512	-2.424759	-1.175515
C	-5.790461	-1.965646	0.427704
H	-4.578689	-0.516057	1.451544
C	-4.732282	-3.136542	-1.398863
H	-2.676157	-2.616240	-1.786914
C	-5.848758	-2.907403	-0.599155
H	-6.658654	-1.789012	1.055285
H	-4.771995	-3.874716	-2.194018
H	-6.764138	-3.465669	-0.771168
C	-1.897262	-0.141252	1.848667
C	-2.346746	1.070509	2.378492
C	-1.409396	-1.128780	2.711074
C	-2.314574	1.287806	3.754163
H	-2.726822	1.847379	1.721749
C	-1.392849	-0.915506	4.084960
H	-1.039256	-2.067636	2.305967

C	-1.843604	0.294722	4.608391
H	-2.662732	2.234076	4.157044
H	-1.012384	-1.688034	4.745390
H	-1.820032	0.465378	5.680400
C	1.964710	-0.173483	1.890429
C	1.693680	1.027165	2.548194
C	2.186167	-1.332603	2.645486
C	1.658302	1.071051	3.940405
H	1.519986	1.937944	1.984878
C	2.164229	-1.282430	4.034623
H	2.392061	-2.276282	2.146137
C	1.900234	-0.078722	4.685774
H	1.443836	2.010308	4.440812
H	2.349830	-2.185381	4.608277
H	1.876604	-0.039869	5.770527
C	3.621546	-1.100202	-0.264728
C	3.732094	-1.998042	-1.329810
C	4.753065	-0.794738	0.502543
C	4.963086	-2.578864	-1.629596
H	2.850842	-2.254656	-1.913476
C	5.978876	-1.378456	0.201931
H	4.674297	-0.100741	1.334518
C	6.084819	-2.270073	-0.865721
H	5.040547	-3.278116	-2.456477
H	6.852281	-1.138246	0.800439
H	7.042398	-2.726845	-1.097149
C	-2.299915	1.082559	-0.736610
C	-1.283142	2.038570	-0.744236
C	-3.498942	1.404101	-1.374745
C	-1.408012	3.291620	-1.339555
C	-3.647577	2.637974	-2.000948
H	-4.312552	0.686526	-1.393010
C	1.043518	2.193977	-0.651914
C	-2.613848	3.571253	-1.982352
H	-4.579054	2.877214	-2.503528
C	2.173355	1.375291	-0.586626
C	1.044413	3.464747	-1.226371
H	-2.759072	4.529351	-2.469149
C	3.363900	1.875121	-1.120911
C	2.248022	3.920935	-1.761074
C	3.397395	3.136813	-1.704180
H	4.263759	1.270313	-1.099825
H	2.298628	4.900022	-2.224449
H	4.326096	3.511139	-2.122170
O	-0.114676	1.692433	-0.109897
C	-0.250654	4.275130	-1.170554
C	-0.364326	4.909984	0.236307
H	-1.297457	5.476654	0.318573
H	-0.357701	4.147828	1.021722
H	0.474743	5.590977	0.411577
C	-0.279567	5.386256	-2.222552
H	0.537528	6.093545	-2.058141
H	-0.195994	4.985634	-3.237311
H	-1.206850	5.960429	-2.149939
Br	0.142130	-3.228567	-2.393666

I_4-I

Cu	-0.022342	0.787167	-0.131175
P	1.795900	1.847023	1.077602
P	1.301638	-2.015197	-0.532577
C	1.823067	-2.895291	1.007239
C	2.430623	-4.099689	3.467424
C	0.803744	-3.255465	1.899129
C	3.149486	-3.153966	1.361913
C	3.450550	-3.746347	2.588698
C	1.102828	-3.858856	3.114884
H	3.959148	-2.902480	0.683952
H	4.486954	-3.937926	2.850586
H	2.666918	-4.563645	4.420238
C	2.423145	1.480533	2.775354
C	3.562126	0.789676	5.239541
C	2.582792	2.443779	3.774791
C	2.864654	0.173163	3.015560
C	3.428510	-0.170899	4.239155
C	3.143141	2.096252	5.002435
H	2.775443	-0.582313	2.240337
H	3.756973	-1.192177	4.407253
H	3.996794	0.522648	6.198048
C	3.422038	2.152096	0.277540
C	5.951931	2.491828	-0.866221
C	4.032496	3.408013	0.264126
C	4.133057	1.071880	-0.249943
C	5.402656	1.212631	-0.814149
C	5.278730	3.581934	-0.324027
H	3.526923	4.259353	0.706354
H	5.732762	4.567122	-0.348728
H	6.927657	2.642074	-1.314525
C	2.873101	-2.020893	-1.499121
C	5.304661	-1.884468	-2.888438
C	3.147719	-2.906595	-2.543550
C	3.841295	-1.059032	-1.194724
C	5.079726	-0.999262	-1.835021
C	4.342717	-2.822772	-3.249242
H	2.413815	-3.657863	-2.815238
H	4.534618	-3.505661	-4.070546

H	6.244822	-1.855103	-3.427977
C	0.313004	-3.330360	-1.376518
C	-1.299083	-5.220397	-2.679430
C	-0.662592	-2.924232	-2.293995
C	0.474956	-4.698926	-1.126485
C	-0.326441	-5.636696	-1.771553
C	-1.462853	-3.862874	-2.941873
H	-0.812171	-1.870286	-2.508227
H	1.229011	-5.035729	-0.421445
H	-0.190454	-6.694107	-1.564745
H	-2.218029	-3.526810	-3.645911
H	-1.926816	-5.952431	-3.178811
C	0.941692	3.467586	1.166016
C	-0.725176	5.720697	1.117728
C	0.031450	3.702674	2.205284
C	0.983931	4.359468	0.087767
C	0.164464	5.484316	0.072358
C	-0.792816	4.824162	2.181865
H	-0.051942	2.998258	3.027349
H	1.623133	4.148877	-0.761210
H	0.209627	6.166542	-0.771090
H	-1.492901	4.991322	2.994651
H	-1.369077	6.594706	1.099475
H	-0.229334	-3.044405	1.633891
H	2.278187	3.470991	3.602115
H	0.299712	-4.135189	3.792203
H	3.257207	2.853356	5.772392
O	3.543844	-0.168765	-0.186631
O	0.682680	1.747440	-1.620673
C	1.079661	1.700189	-2.955010
C	0.901288	0.309919	-3.579800
C	0.256251	2.730183	-3.753855
C	2.566870	2.083004	-3.070959
H	1.492252	-0.425363	-3.026575
H	-0.148082	0.011961	-3.557001
H	1.240674	0.294052	-4.623365
H	0.360339	3.719788	-3.292592
H	0.591569	2.801209	-4.796162
H	-0.803231	2.463316	-3.749096
H	2.874449	2.184404	-4.119214
H	2.763687	3.032774	-2.561438
H	3.195276	1.316088	-2.612556
C	6.128486	-0.049665	-1.265880
C	7.247051	0.248283	-2.266350
H	6.862863	0.724549	-3.173553
H	7.998929	0.903786	-1.819366
H	7.765280	-0.672093	-2.547879
C	6.742409	-0.720307	-0.012389
H	7.232965	-1.659492	-0.288560
H	7.485178	-0.057713	0.443693
H	5.976523	-0.939872	0.738217
C	-0.919984	0.466344	1.577828
C	-0.399611	-0.286372	2.536394
H	-0.982198	-0.496337	3.435932
H	0.577356	-0.744533	2.480094
C	-1.888836	0.230309	-0.710063
H	-1.640057	-0.806107	-0.929188
C	-2.225481	0.859124	-2.033337
O	-2.318501	0.189147	-3.044584
O	-2.553228	2.161277	-2.126048
C	-2.090125	3.127699	-1.178208
H	-2.254219	4.096523	-1.653341
H	-2.671330	3.092912	-0.254801
H	-1.022857	2.987422	-1.004604
C	-2.981498	0.345238	0.389037
B	-3.399475	-1.118882	0.830277
B	-4.330184	1.021670	-0.069810
O	-2.561458	-2.202182	0.853499
O	-4.673818	-1.431283	1.236294
C	-3.273836	-3.281310	1.503394
C	-4.763856	-2.876648	1.254091
C	-2.903582	-3.245697	2.985935
H	-3.363395	-4.077767	3.526794
H	-1.820065	-3.326342	3.095425
H	-3.221580	-2.308634	3.452893
C	-2.842941	-4.594350	0.871508
H	-2.931960	-4.561543	-0.216146
H	-1.796783	-4.799006	1.117555
H	-3.450305	-5.421348	1.253294
C	-5.739903	-3.299676	2.339055
H	-6.750114	-2.975032	2.072631
H	-5.749400	-4.389556	2.439992
H	-5.485177	-2.860540	3.305635
C	-5.273614	-3.308297	-0.120368
H	-5.427232	-4.390341	-0.163996
H	-6.229792	-2.815449	-0.315626
H	-4.575467	-3.022486	-0.913111
O	-4.917986	2.072453	0.584631
O	-5.061945	0.572841	-1.136107
C	-6.355024	1.211067	-1.067808
C	-7.295935	0.229148	-0.369293
H	-6.938491	-0.015540	0.634411
H	-8.312617	0.627384	-0.303075
H	-7.327979	-0.697937	-0.948675

C	-6.843139	1.491060	-2.480949
H	-6.106579	2.053853	-3.057382
H	-7.028262	0.546008	-2.999749
H	-7.780830	2.055663	-2.458442
C	-6.051695	2.492771	-0.213235
C	-7.174633	2.918619	0.719175
H	-6.876533	3.814451	1.272022
H	-8.076603	3.157993	0.147040
H	-7.415660	2.136298	1.441799
C	-5.586012	3.676906	-1.059358
H	-6.414567	4.107762	-1.628790
H	-5.183906	4.450860	-0.399011
H	-4.800458	3.376706	-1.757719
C	-2.304357	1.033864	1.596738
H	-2.800509	0.852560	2.562055
H	-2.283340	2.118533	1.449613

I_5-I

C	1.096270	-2.103317	-1.518358
H	0.677589	-3.111740	-1.492934
C	1.478682	-1.750429	-2.863906
O	2.325036	-0.920103	-3.220973
O	0.749951	-2.407548	-3.820303
C	0.891649	-1.931473	-5.145215
H	0.217197	-2.535430	-5.755573
H	0.606422	-0.876406	-5.226514
H	1.917593	-2.044273	-5.509418
C	2.212543	-1.921289	-0.455837
C	2.533168	-0.406771	-0.233079
H	2.091263	0.121385	-1.077197
H	2.034320	-0.071040	0.681632
C	3.981619	0.012826	-0.211217
C	4.687494	0.320665	-1.296128
H	5.730904	0.613851	-1.270650
H	4.186457	0.219071	-2.257851
I	4.922171	0.157610	1.702357
P	-3.613017	-0.826423	1.363774
P	-1.059993	1.314681	-1.252630
C	-5.138943	-0.621958	0.336832
C	-6.272274	-1.424922	0.477965
C	-5.168265	0.365777	-0.647705
C	-7.377935	-1.253988	-0.346213
H	-6.279762	-2.208732	1.228374
C	-6.282968	0.597667	-1.457971
C	-7.382579	-0.244852	-1.303987
H	-8.243040	-1.900188	-0.235536
C	-3.777654	1.652934	-2.020377
H	-8.259797	-0.110635	-1.927105
C	-4.821741	1.944202	-2.904050
C	-2.436325	1.888803	-2.336498
C	-4.491536	2.417064	-4.172394
C	-2.149931	2.386480	-3.612521
C	-3.164469	2.633040	-4.528282
H	-5.273802	2.631878	-4.891518
H	-1.120502	2.565836	-3.903702
H	-2.920671	3.004602	-5.518354
O	-4.048606	1.147963	-0.779292
C	-6.251642	1.801668	-2.392745
C	-6.590571	3.062357	-1.560934
H	-6.540913	3.957317	-2.190280
H	-7.601409	2.981008	-1.147681
H	-5.891320	3.189435	-0.728473
C	-7.264357	1.676454	-3.534085
H	-8.281339	1.599783	-3.140945
H	-7.245001	2.566628	-4.168462
H	-7.064122	0.798844	-4.156333
C	-4.123432	0.100989	2.889114
C	-3.423912	-0.109908	4.086972
C	-5.062462	1.138141	2.852641
C	-3.666692	0.673674	5.210829
H	-2.681573	-0.900355	4.145343
C	-5.295061	1.935707	3.971449
H	-5.622147	1.331625	1.942304
C	-4.600678	1.706645	5.156050
H	-3.119240	0.480453	6.129042
H	-6.028253	2.735405	3.915586
H	-4.787619	2.323989	6.029698
C	-3.814241	-2.601645	1.851450
C	-4.373722	-3.075750	3.040719
C	-3.322741	-3.519795	0.911495
C	-4.433019	-4.446766	3.293812
H	-4.768011	-2.381252	3.776501
C	-3.403536	-4.886942	1.159639
H	-2.892985	-3.124629	-0.015488
C	-3.950501	-5.354767	2.354824
H	-4.865653	-4.803481	4.224380
H	-3.023340	-5.589707	0.423043
H	-4.000013	-6.421661	2.553098
C	-1.373057	1.981830	0.438087
C	-2.262587	3.016147	0.739406
C	-0.646673	1.385895	1.477671
C	-2.425678	3.444696	2.055174
H	-2.843502	3.480525	-0.051771
C	-0.786204	1.837199	2.788028
H	-0.009688	0.527294	1.271912

C	-1.682120	2.864123	3.079626
H	-3.137138	4.233385	2.280124
H	-0.221603	1.361000	3.584527
H	-1.817291	3.194427	4.104663
C	5.392761	-3.984796	-0.483318
C	4.972930	-4.029134	-1.992445
B	3.483892	-2.786757	-0.817275
O	3.612321	-3.558021	-1.944565
O	4.561994	-2.924867	0.030058
O	1.585550	-1.832022	2.063390
O	1.345360	-3.861410	1.039100
B	1.703027	-2.543052	0.892908
C	0.890123	-2.673278	3.007871
C	1.186388	-4.108316	2.453975
C	-0.582955	-2.294602	2.912631
H	-0.688830	-1.228498	3.133196
H	-0.964974	-2.463681	1.902599
H	-1.199025	-2.861706	3.616593
C	1.426999	-2.399418	4.403448
H	1.171908	-1.377853	4.702192
H	0.978007	-3.086111	5.128167
H	2.513139	-2.505101	4.445675
C	0.061121	-5.109122	2.659012
H	0.347368	-6.078813	2.240802
H	-0.142005	-5.245652	3.726306
H	-0.857384	-4.783310	2.167826
C	2.511230	-4.679494	2.958197
H	3.330880	-3.971633	2.801939
H	2.459286	-4.930826	4.021613
H	2.740017	-5.592180	2.401282
C	5.752550	-3.039981	-2.860144
H	5.260846	-2.962270	-3.833655
H	5.762292	-2.043231	-2.408335
H	6.785019	-3.366591	-3.015996
C	4.996959	-5.412928	-2.623351
H	4.691505	-5.345760	-3.671689
H	6.007449	-5.833047	-2.589808
H	4.315178	-6.097774	-2.115093
C	6.851219	-3.630766	-0.235081
H	7.049299	-3.616788	0.840849
H	7.512358	-4.373570	-0.693195
H	7.097641	-2.645612	-0.636935
C	5.017915	-5.255772	0.278105
H	5.172621	-5.088277	1.347584
H	3.965429	-5.509836	0.122636
H	5.635465	-6.104989	-0.028874
C	0.264697	2.494572	-1.811650

C	0.236136	3.860050	-1.488964
C	1.321030	2.008137	-2.589625
C	1.259612	4.709623	-1.901103
H	-0.583429	4.263365	-0.902486
C	2.347369	2.861425	-3.003004
H	1.369322	0.957262	-2.867296
C	2.325209	4.208154	-2.651345
H	1.227761	5.761489	-1.633456
H	3.165479	2.463918	-3.595759
H	3.132249	4.866621	-2.957554
K	2.502057	2.689743	0.392217
O	1.957576	4.633710	2.011654
C	2.426527	4.807534	3.349345
C	0.715441	5.334586	1.844623
C	1.165279	4.997174	4.177992
H	3.012437	3.925051	3.620901
H	3.076499	5.692782	3.408048
C	0.306908	5.844583	3.232714
H	0.853439	6.149908	1.125118
H	-0.015775	4.628611	1.437486
H	1.359315	5.484723	5.135932
H	0.692898	4.027600	4.364525
H	0.558271	6.903923	3.341972
H	-0.764674	5.727582	3.408553
O	4.579968	4.129803	-0.210051
C	5.570393	3.819510	-1.199351
C	4.522125	5.543959	0.008397
C	6.033177	5.157583	-1.770248
H	6.394252	3.282027	-0.713241
H	5.123037	3.163167	-1.952921
C	5.808317	6.102058	-0.586945
H	3.638249	5.953900	-0.500435
H	4.416332	5.723443	1.081279
H	5.402422	5.452239	-2.615849
H	7.070313	5.124853	-2.110567
H	5.711758	7.149877	-0.879848
H	6.629260	6.015334	0.132574
Cu	-0.719291	-1.043530	-1.350280
O	-2.584966	-1.745581	-1.539035
C	-3.001770	-2.399234	-2.683075
C	-2.262152	-3.743755	-2.860253
C	-4.516249	-2.681529	-2.588491
C	-2.743032	-1.532419	-3.935915
H	-1.189388	-3.556925	-2.966099
H	-2.420707	-4.375167	-1.977356
H	-2.605279	-4.302847	-3.742288
H	-5.069137	-1.740912	-2.497837

H	-4.896818	-3.220328	-3.467003
H	-4.731843	-3.283179	-1.698296
H	-3.047786	-2.026982	-4.869105
H	-3.290584	-0.585723	-3.856066
H	-1.672052	-1.305911	-4.000803

TS2-I

Cu	-1.198927	-0.591872	0.203100
P	1.087711	-3.315556	-1.430095
P	1.336078	-0.115217	1.057404
C	3.018670	-0.331786	0.281239
C	5.518934	-0.483297	-0.992832
C	3.106961	-0.440220	-1.108423
C	4.205034	-0.338614	1.029147
C	5.443093	-0.408190	0.398651
C	4.348519	-0.508710	-1.744058
H	4.166095	-0.306110	2.113780
H	6.350911	-0.406495	0.994568
H	6.485166	-0.537481	-1.484822
C	2.521182	-3.438739	-2.590800
C	4.718645	-3.301674	-4.337146
C	3.725545	-4.071317	-2.272253
C	2.439438	-2.722692	-3.794966
C	3.520969	-2.666819	-4.667579
C	4.818157	-3.996982	-3.135640
H	1.519976	-2.200240	-4.050771
H	3.432680	-2.117832	-5.600521
H	5.568010	-3.250957	-5.011895
C	1.502840	-4.608974	-0.171324
C	1.970292	-6.466675	1.878880
C	1.293944	-5.975023	-0.381786
C	1.971755	-4.216129	1.083519
C	2.218193	-5.118028	2.122957
C	1.517932	-6.896195	0.633770
H	0.925771	-6.315414	-1.344535
H	1.336645	-7.951935	0.458719
H	2.131712	-7.198038	2.663086
C	1.559945	-1.048711	2.622542
C	1.841271	-2.649032	4.904339
C	1.270323	-0.535986	3.886741
C	1.952922	-2.386095	2.536801
C	2.147411	-3.193750	3.656762
C	1.396200	-1.335041	5.018906
H	0.932292	0.488897	3.986394
H	1.154715	-0.928930	5.995984
H	1.957676	-3.248475	5.800659
C	1.527418	1.652901	1.595796
C	1.857423	4.403829	2.070963
C	0.707897	2.593030	0.966422
C	2.504250	2.113747	2.493793
C	2.664632	3.474372	2.732474
C	0.875292	3.960509	1.191986
H	-0.064778	2.256828	0.285843
H	3.141719	1.409376	3.017209
H	3.421437	3.811728	3.434161
H	0.227386	4.666745	0.684230
H	1.989818	5.465933	2.253162
C	-0.250456	-4.200738	-2.353036
C	-2.436071	-5.481980	-3.548304
C	-0.085887	-4.919988	-3.540318
C	-1.525040	-4.116764	-1.774480
C	-2.605539	-4.768449	-2.362356
C	-1.176455	-5.550509	-4.138454
H	0.893992	-4.995887	-4.002231
H	-1.636436	-3.538874	-0.853233
H	-3.585156	-4.715516	-1.895567
H	-1.036969	-6.104152	-5.062600
H	-3.282242	-5.981645	-4.011034
H	2.200743	-0.467911	-1.702867
H	3.818410	-4.622818	-1.341342
H	4.389795	-0.601870	-2.824839
H	5.747633	-4.491235	-2.867977
O	2.188457	-2.878130	1.281539
C	-1.911706	-3.045644	1.858133
C	-1.818688	-2.357914	3.233747
C	-3.385891	-3.106764	1.413422
C	-1.407022	-4.493655	2.019907
H	-0.796598	-2.433496	3.617185
H	-2.062088	-1.294498	3.153634
H	-2.491578	-2.816495	3.970845
H	-3.466124	-3.656186	0.466939
H	-4.023088	-3.616375	2.148628
H	-3.792550	-2.106238	1.252424
H	-2.019832	-5.061195	2.732567
H	-1.425946	-5.016806	1.057417
H	-0.375248	-4.494611	2.381302
I	0.403170	1.741109	-2.725352
K	3.332979	2.666451	-0.818256
C	-2.174656	1.742686	1.970751
O	-2.217698	2.944644	1.699644
O	-1.607155	1.313296	3.127574
C	-1.784939	-0.728621	-2.027290
H	-0.904643	-1.306850	-2.288478
H	-2.762999	-1.147776	-2.264119
B	-4.659759	0.153215	-0.456669

O	-5.072391	-0.013496	-1.757649
O	-5.388335	-0.596164	0.428891
O	-4.482651	3.598097	-0.361919
O	-5.282320	2.444446	1.444679
B	-4.399542	2.454235	0.393789
C	-5.605391	4.361033	0.123249
C	-5.824462	3.773951	1.565259
C	-6.020876	-1.106693	-1.769292
C	-5.240413	-2.365283	-2.143263
H	-5.896300	-3.237418	-2.218838
H	-4.758260	-2.217268	-3.114192
H	-4.462860	-2.574736	-1.402479
C	-7.094220	-0.817459	-2.806118
H	-6.652835	-0.823295	-3.807010
H	-7.873208	-1.585819	-2.773823
H	-7.557719	0.158021	-2.644552
C	-6.525170	-1.124838	-0.284708
C	-6.856275	-2.505602	0.259870
H	-7.178752	-2.421824	1.301800
H	-7.672102	-2.956993	-0.313904
H	-5.991910	-3.171167	0.225484
C	-7.687324	-0.164349	-0.032310
H	-7.842349	-0.072689	1.046036
H	-7.468830	0.832380	-0.426605
H	-8.613708	-0.525966	-0.487734
C	2.773259	-4.568885	3.435223
C	2.517559	-5.514007	4.611458
H	1.447978	-5.686682	4.765557
H	3.006668	-6.477931	4.447553
H	2.940725	-5.104452	5.532579
C	4.299952	-4.373487	3.271038
H	4.727166	-3.946564	4.184693
H	4.785132	-5.335481	3.075119
H	4.527934	-3.699985	2.438937
C	-1.715522	0.488753	-1.483555
C	-3.551039	1.175000	-0.022873
C	-1.140605	2.329811	4.003108
H	-0.277612	2.855730	3.583270
H	-1.926950	3.059190	4.217700
H	-0.851738	1.821607	4.924889
C	-7.282089	3.667756	1.990372
H	-7.852720	3.025357	1.316087
H	-7.341642	3.240855	2.995887
H	-7.750411	4.657135	2.013462
C	-5.012488	4.493995	2.642071
H	-5.390679	5.504941	2.821285
H	-5.090101	3.928171	3.575485
H	-3.956037	4.540267	2.367524
C	-5.249429	5.840033	0.088422
H	-5.107785	6.161555	-0.947736
H	-6.055330	6.440346	0.522986
H	-4.327073	6.041986	0.636565
C	-6.769195	4.080264	-0.827928
H	-7.657712	4.660832	-0.563331
H	-6.469844	4.351349	-1.844336
H	-7.032767	3.018252	-0.827176
C	-2.700350	0.682824	1.139223
H	-3.112432	-0.137318	1.722625
C	-2.635629	1.607231	-1.205550
H	-2.070820	2.479220	-0.871969
H	-3.181567	1.888774	-2.113009
O	-1.124648	-2.402343	0.913084
C	3.070611	5.642683	-2.857206
C	3.797092	7.424646	-1.444106
C	3.122612	7.171102	-2.795667
H	2.156725	5.265244	-3.326329
H	3.935059	5.228675	-3.390879
H	3.579238	8.411555	-1.030179
H	4.884230	7.311489	-1.525916
H	2.110457	7.587381	-2.793976
H	3.667904	7.598551	-3.639661
C	6.109307	3.210437	1.329017
C	6.689636	5.394599	0.600803
C	6.087799	4.655440	1.805829
H	7.110018	2.767288	1.441028
H	5.381760	2.561514	1.824158
H	7.773543	5.486680	0.710381
H	6.278023	6.399571	0.479653
H	6.653467	4.807101	2.727830
H	5.052634	4.968826	1.977964
O	5.756753	3.292784	-0.048849
C	6.345317	4.481148	-0.592636
H	5.609485	4.923277	-1.270664
H	7.239399	4.216494	-1.169972
C	3.211659	6.297470	-0.607307
H	3.832637	6.003605	0.244153
H	2.210451	6.558457	-0.237373
O	3.124576	5.181913	-1.496210

I_4-Cl

Cu	-0.092225	0.057543	-0.530504
C	2.947602	0.623266	0.020868
C	1.901068	0.350672	-1.112415
P	-1.608005	1.838837	-0.051911
P	-1.271999	-1.924514	-1.022067

B	2.221065	0.328478	1.387062
O	2.051321	-0.949299	1.877254
O	1.744703	1.279237	2.258940
C	-3.982806	-1.359875	-4.743618
C	-1.804531	-1.548146	-3.724580
C	-3.787859	-1.701895	-2.360169
C	-4.575631	-1.512895	-3.494215
C	-2.593573	-1.377983	-4.856120
H	-4.268956	-1.839385	-1.397247
H	-5.656863	-1.492668	-3.397077
H	-4.599521	-1.219527	-5.626171
C	-2.802016	1.951025	-1.443330
C	-4.493901	2.166252	-3.656299
C	-4.166759	2.191946	-1.265597
C	-2.289961	1.808804	-2.738272
C	-3.133031	1.930676	-3.838506
C	-5.010395	2.291819	-2.369160
H	-1.231437	1.602257	-2.894301
H	-2.727304	1.820524	-4.839142
H	-5.151803	2.245086	-4.516611
C	-2.676639	1.671573	1.437322
C	-4.130121	1.148719	3.775315
C	-2.821727	2.657634	2.415500
C	-3.303648	0.443388	1.658271
C	-4.037336	0.149698	2.805941
C	-3.535869	2.392533	3.579709
H	-2.351958	3.625794	2.278789
H	-3.630952	3.160829	4.340087
H	-4.682638	0.965031	4.690167
C	-2.320304	-2.662403	0.301710
C	-3.736015	-3.565458	2.548102
C	-2.251899	-4.001930	0.692475
C	-3.122175	-1.809203	1.063593
C	-3.845536	-2.224908	2.181099
C	-2.950809	-4.446443	1.809585
H	-1.631791	-4.696976	0.137121
H	-2.882258	-5.487406	2.108269
H	-4.271937	-3.935564	3.415126
C	-0.217601	-3.332027	-1.554388
C	1.462785	-5.412170	-2.367128
C	1.131635	-3.328065	-1.189871
C	-0.719286	-4.388134	-2.326676
C	0.115421	-5.425458	-2.726692
C	1.966298	-4.364985	-1.602343
H	1.541410	-2.513308	-0.598692
H	-1.765517	-4.395666	-2.618834
H	-0.282691	-6.239468	-3.324769

H	3.016976	-4.339331	-1.336595
H	2.119278	-6.214586	-2.690168
C	-0.994304	3.565216	0.111336
C	0.048374	6.141058	0.421210
C	-1.628320	4.657996	-0.484902
C	0.165612	3.771221	0.865685
C	0.679561	5.054120	1.024913
C	-1.105100	5.940818	-0.333097
H	-2.527524	4.513671	-1.076044
H	0.674792	2.925545	1.319619
H	1.584127	5.201678	1.607584
H	-1.600643	6.783017	-0.806645
H	0.456685	7.140815	0.535794
H	-0.722044	-1.548434	-3.820215
H	-4.574806	2.305538	-0.265411
H	-2.121315	-1.250637	-5.825461
H	-6.070951	2.472682	-2.221631
O	-3.169472	-0.495979	0.666566
C	-2.396822	-1.726221	-2.467542
C	-4.744222	-1.202814	2.876339
C	-6.064005	-1.096011	2.075874
H	-6.581164	-2.060951	2.070005
H	-6.721824	-0.349336	2.532409
H	-5.879626	-0.799390	1.038703
C	-5.066393	-1.607878	4.316997
H	-5.597041	-2.563217	4.339179
H	-4.160404	-1.697375	4.924360
H	-5.728460	-0.874635	4.784756
C	1.402928	0.619562	3.498924
C	1.212712	-0.871954	3.048297
C	1.685654	-1.908913	4.055635
H	2.747191	-1.795356	4.285167
H	1.115495	-1.827862	4.986540
H	1.529864	-2.912263	3.648315
C	-0.210588	-1.190822	2.600016
H	-0.556366	-0.469492	1.850659
H	-0.223969	-2.183511	2.142755
H	-0.913947	-1.187381	3.437501
C	0.155239	1.271921	4.071910
H	-0.164580	0.757082	4.983441
H	0.365314	2.315367	4.325474
H	-0.668260	1.256353	3.356498
C	2.593371	0.819585	4.435408
H	2.775674	1.891407	4.553517
H	2.403649	0.391734	5.423874
H	3.500418	0.365403	4.024472
H	2.128996	-0.570440	-1.649313

C	1.519735	1.343171	-2.095088
O	1.160404	1.114921	-3.256398
O	1.498538	2.627563	-1.631123
C	1.192412	3.639749	-2.568891
H	0.178907	3.534858	-2.968385
H	1.271821	4.581941	-2.022760
H	1.901668	3.636579	-3.403272
B	4.099392	-0.447110	0.072923
O	4.136670	-1.658830	-0.574794
O	5.186786	-0.258743	0.897850
C	5.194153	-2.433645	0.040889
C	6.124090	-1.317595	0.626365
C	5.856774	-3.310590	-1.009572
H	6.739363	-3.803149	-0.588676
H	5.168109	-4.090525	-1.345943
H	6.163129	-2.730039	-1.882063
C	4.545260	-3.289594	1.128772
H	3.775816	-3.923512	0.680339
H	5.279820	-3.937141	1.616325
H	4.060119	-2.662961	1.881854
C	6.834679	-1.692297	1.918151
H	7.479580	-2.563424	1.764266
H	7.462852	-0.859656	2.248314
H	6.123597	-1.918726	2.715449
C	7.129269	-0.776101	-0.390986
H	7.583816	0.133436	0.011697
H	7.924686	-1.499043	-0.594106
H	6.641180	-0.517256	-1.335295
C	3.579789	2.031062	0.084557
H	2.815197	2.797728	0.221031
H	4.241417	2.084152	0.958051
C	4.400080	2.414319	-1.113049
C	4.753038	1.680298	-2.161407
H	5.370888	2.070523	-2.961889
H	4.395227	0.659387	-2.246718
Cl	4.961296	4.095275	-1.039835

I_5-Cl

C	1.401750	-2.103997	-1.340909
H	0.974938	-3.109251	-1.335843
C	1.858840	-1.761512	-2.665052
O	2.711915	-0.922530	-2.984424
O	1.198978	-2.443183	-3.652898
C	1.409352	-1.981812	-4.973944
H	0.767481	-2.593063	-5.611607
H	1.129805	-0.927842	-5.082104
H	2.452992	-2.098967	-5.282478
C	2.453678	-1.924821	-0.213554
C	2.774529	-0.415380	0.048302
H	2.284174	0.138166	-0.751862
H	2.325166	-0.111492	0.998722
C	4.218536	0.014107	0.034847
C	4.936722	0.293794	-1.047713
H	5.975531	0.600796	-1.000840
H	4.452987	0.145564	-2.009816
Cl	4.967390	0.210500	1.633694
P	-3.430251	-0.837067	1.224902
P	-0.719141	1.339909	-1.170219
C	-4.889576	-0.601700	0.112808
C	-6.032860	-1.401322	0.163211
C	-4.855526	0.414426	-0.841767
C	-7.087451	-1.197260	-0.718731
H	-6.088452	-2.206852	0.888205
C	-5.919632	0.679569	-1.707524
C	-7.031320	-0.158902	-1.643263
H	-7.961073	-1.840237	-0.678724
C	-3.377224	1.723141	-2.095006
H	-7.870135	0.000378	-2.311735
C	-4.365522	2.048264	-3.029243
C	-2.017714	1.951564	-2.326783
C	-3.958026	2.546555	-4.264925
C	-1.652707	2.475510	-3.571780
C	-2.610214	2.754923	-4.538377
H	-4.696078	2.786909	-5.021945
H	-0.606436	2.650361	-3.798514
H	-2.305507	3.146322	-5.503629
O	-3.724913	1.190716	-0.884947
C	-5.823432	1.909897	-2.602811
C	-6.193722	3.150290	-1.754021
H	-6.098922	4.062327	-2.352914
H	-7.226796	3.070322	-1.399689
H	-5.540411	3.244239	-0.880861
C	-6.771925	1.831607	-3.801615
H	-7.810064	1.757743	-3.467605
H	-6.705955	2.739529	-4.407008
H	-6.548098	0.969759	-4.437696
C	-4.026658	0.070253	2.731298
C	-3.411966	-0.169188	3.969610
C	-4.947681	1.121061	2.650060
C	-3.719505	0.599515	5.087957
H	-2.684880	-0.969969	4.063933
C	-5.244324	1.903896	3.764298
H	-5.443910	1.337253	1.708726
C	-4.634274	1.646210	4.988722
H	-3.236778	0.384074	6.037028

H	-5.961840	2.714475	3.672931
H	-4.871303	2.252008	5.858306
C	-3.672724	-2.619333	1.666318
C	-4.303987	-3.111342	2.811469
C	-3.135298	-3.523790	0.738235
C	-4.389621	-4.486485	3.032701
H	-4.734582	-2.427847	3.537114
C	-3.242603	-4.894683	0.952788
H	-2.652636	-3.114541	-0.155494
C	-3.861970	-5.380531	2.104687
H	-4.878611	-4.857254	3.929253
H	-2.826936	-5.586384	0.224991
H	-3.932693	-6.450582	2.277892
C	-1.131364	1.989715	0.507010
C	-2.032536	3.025343	0.767152
C	-0.474090	1.375388	1.581404
C	-2.276322	3.436075	2.076295
H	-2.558943	3.505648	-0.052160
C	-0.695652	1.807132	2.886808
H	0.170942	0.517139	1.402318
C	-1.603671	2.834615	3.136952
H	-2.996218	4.226087	2.267247
H	-0.185265	1.315887	3.710299
H	-1.801745	3.150390	4.156302
C	5.636126	-3.974826	-0.042104
C	5.310594	-4.024033	-1.574844
B	3.747980	-2.785606	-0.494478
O	3.950449	-3.551094	-1.613746
O	4.771226	-2.917119	0.419098
O	1.694439	-1.871954	2.273619
O	1.481371	-3.876627	1.195983
B	1.864765	-2.560975	1.096127
C	0.923605	-2.718238	3.154089
C	1.232479	-4.147273	2.593363
C	-0.533836	-2.314214	2.966478
H	-0.637585	-1.248699	3.189980
H	-0.850767	-2.469792	1.932129
H	-1.203074	-2.878459	3.622457
C	1.368253	-2.479770	4.587779
H	1.110603	-1.459498	4.888805
H	0.858481	-3.171423	5.266068
H	2.446783	-2.607187	4.702688
C	0.082157	-5.133621	2.710410
H	0.379346	-6.100216	2.292659
H	-0.187976	-5.285669	3.760613
H	-0.799474	-4.785232	2.170003
C	2.514189	-4.748967	3.167923

H	3.352314	-4.051333	3.079033
H	2.390768	-5.020517	4.220430
H	2.764782	-5.654017	2.607951
C	6.145221	-3.040975	-2.396715
H	5.716112	-2.966932	-3.399793
H	6.130133	-2.042212	-1.950054
H	7.184322	-3.371501	-2.486129
C	5.370684	-5.410416	-2.198078
H	5.131886	-5.345975	-3.263779
H	6.375858	-5.833129	-2.099815
H	4.656342	-6.091785	-1.731614
C	7.076060	-3.617086	0.293733
H	7.209914	-3.604647	1.379527
H	7.765023	-4.357773	-0.125089
H	7.343946	-2.631071	-0.091602
C	5.220645	-5.246711	0.696337
H	5.309609	-5.078589	1.773193
H	4.180606	-5.505208	0.477477
H	5.858828	-6.093643	0.427677
C	0.653334	2.507564	-1.630327
C	0.622827	3.869193	-1.291857
C	1.744765	2.017604	-2.356172
C	1.675150	4.712055	-1.639868
H	-0.222026	4.275137	-0.744393
C	2.799569	2.864681	-2.705940
H	1.797754	0.968211	-2.640397
C	2.772334	4.207708	-2.341078
H	1.640651	5.761050	-1.361451
H	3.644079	2.464935	-3.258780
H	3.600823	4.860443	-2.597890
K	2.818429	2.677240	0.680160
O	2.162538	4.547518	2.343292
C	2.529080	4.675482	3.718211
C	0.934046	5.252766	2.104265
C	1.212186	4.868810	4.454234
H	3.075472	3.774005	4.009472
H	3.189288	5.544719	3.851116
C	0.441437	5.751839	3.467659
H	1.119088	6.072347	1.400609
H	0.226979	4.552077	1.647598
H	1.342058	5.331064	5.435260
H	0.710885	3.904254	4.581758
H	0.710049	6.802771	3.612189
H	-0.642302	5.658584	3.566133
O	4.899144	4.153640	0.196662
C	5.937019	3.879504	-0.754632
C	4.802608	5.562666	0.433322

C	6.409260	5.236909	-1.270888
H	6.744250	3.339324	-0.244608
H	5.532781	3.237028	-1.543915
C	6.109361	6.151936	-0.080991
H	3.941703	5.966205	-0.118572
H	4.632978	5.721553	1.501253
H	5.820356	5.543135	-2.142153
H	7.463247	5.225481	-1.556391
H	6.012533	7.204453	-0.356370
H	6.892616	6.060648	0.678820
Cu	-0.414179	-1.025375	-1.289538
O	-2.274364	-1.681521	-1.632423
C	-2.620704	-2.303583	-2.816984
C	-1.893183	-3.657217	-2.974285
C	-4.142398	-2.560479	-2.834409
C	-2.262375	-1.415105	-4.029276
H	-0.812429	-3.489357	-3.000539
H	-2.125281	-4.306447	-2.121009
H	-2.183660	-4.188767	-3.891610
H	-4.685872	-1.612865	-2.762160
H	-4.468894	-3.074065	-3.749077
H	-4.428936	-3.177415	-1.975207
H	-2.504543	-1.886549	-4.992255
H	-2.803508	-0.463275	-3.969124
H	-1.187146	-1.199894	-4.012923

TS2-Cl

Cu	-1.137681	-0.519590	0.152949
P	1.395535	-3.184070	-1.485921
P	1.338319	0.124214	0.916400
C	2.996992	-0.050431	0.087367
C	5.454073	-0.213817	-1.263697
C	3.045285	-0.037796	-1.309655
C	4.197931	-0.170052	0.798584
C	5.414723	-0.255024	0.129511
C	4.267291	-0.104889	-1.981083
H	4.186196	-0.217958	1.883824
H	6.334647	-0.354556	0.698184
H	6.403145	-0.282711	-1.786437
C	2.857832	-3.280830	-2.609645
C	5.099686	-3.073560	-4.291784
C	4.104507	-3.761519	-2.199344
C	2.755366	-2.680855	-3.873766
C	3.860600	-2.591765	-4.713565
C	5.217662	-3.652743	-3.031608
H	1.800655	-2.276936	-4.203661
H	3.756670	-2.135664	-5.693836
H	5.966183	-2.995827	-4.941692
C	1.842252	-4.410532	-0.172125
C	2.369886	-6.170688	1.944898
C	1.748304	-5.793387	-0.352930
C	2.227584	-3.949041	1.087436
C	2.502349	-4.801199	2.161130
C	2.002013	-6.667203	0.696565
H	1.449215	-6.185888	-1.319837
H	1.912163	-7.738281	0.545564
H	2.560751	-6.868033	2.753179
C	1.617258	-0.749332	2.509052
C	1.949090	-2.246313	4.853626
C	1.308114	-0.197496	3.752186
C	2.062646	-2.073007	2.478024
C	2.282648	-2.828063	3.629795
C	1.457042	-0.945927	4.915508
H	0.939969	0.820498	3.811773
H	1.199450	-0.509718	5.875374
H	2.085136	-2.804178	5.773683
C	1.477242	1.904714	1.425204
C	1.686944	4.653753	1.958188
C	0.540835	2.804356	0.911705
C	2.517513	2.401843	2.226042
C	2.620889	3.762572	2.492638
C	0.645948	4.172702	1.171002
H	-0.263259	2.440231	0.285236
H	3.249865	1.722202	2.650035
H	3.429546	4.128971	3.117869
H	-0.095129	4.850684	0.761021
H	1.770491	5.716536	2.164008
C	0.105336	-4.167364	-2.373600
C	-2.013016	-5.630387	-3.474913
C	0.295309	-4.918962	-3.536300
C	-1.161820	-4.140126	-1.773025
C	-2.206742	-4.884715	-2.312592
C	-0.763211	-5.639335	-4.089042
H	1.270790	-4.952621	-4.012422
H	-1.298480	-3.538157	-0.870291
H	-3.178064	-4.880079	-1.826400
H	-0.604967	-6.217952	-4.994696
H	-2.832963	-6.202135	-3.899802
H	2.121563	0.032515	-1.877321
H	4.214399	-4.218363	-1.220268
H	4.282192	-0.103697	-3.066677
H	6.179059	-4.027343	-2.692249
O	2.334583	-2.595008	1.244674
C	-1.708450	-2.970435	1.850987
C	-1.743734	-2.231008	3.202245

C	-3.147536	-3.179682	1.341153
C	-1.085131	-4.359636	2.090157
H	-0.733858	-2.179204	3.621363
H	-2.099154	-1.204645	3.077014
H	-2.388161	-2.738174	3.933009
H	-3.132890	-3.775945	0.420276
H	-3.776004	-3.708940	2.070012
H	-3.628336	-2.226582	1.111784
H	-1.690051	-4.966150	2.776972
H	-0.987412	-4.903623	1.144003
H	-0.086072	-4.254001	2.520704
Cl	0.240258	1.820010	-2.391153
K	2.772443	3.074743	-1.263067
C	-2.336284	1.731026	1.808435
O	-2.448331	2.924327	1.527862
O	-1.775663	1.340901	2.979799
C	-1.554919	-0.716072	-2.146969
H	-0.597857	-1.218151	-2.262221
H	-2.444606	-1.194679	-2.555950
B	-4.621668	-0.066064	-0.678450
O	-4.980163	-0.272230	-1.989219
O	-5.304817	-0.875247	0.190845
O	-4.737050	3.374736	-0.634197
O	-5.483349	2.199059	1.181306
B	-4.581552	2.256710	0.148148
C	-5.932124	4.050508	-0.193822
C	-6.131641	3.484050	1.258565
C	-5.810948	-1.456336	-2.021325
C	-4.890440	-2.628740	-2.352851
H	-5.446477	-3.567126	-2.434960
H	-4.395723	-2.441531	-3.310593
H	-4.118342	-2.742653	-1.586232
C	-6.872237	-1.287451	-3.096235
H	-6.398472	-1.254116	-4.081707
H	-7.567987	-2.132476	-3.082080
H	-7.439536	-0.364284	-2.960163
C	-6.360767	-1.516461	-0.554351
C	-6.572545	-2.920673	-0.010095
H	-6.934590	-2.864806	1.020687
H	-7.321926	-3.451638	-0.606035
H	-5.646820	-3.498882	-0.013085
C	-7.619872	-0.674371	-0.347775
H	-7.817163	-0.589944	0.724329
H	-7.490596	0.336086	-0.746646
H	-8.490898	-1.130475	-0.827068
C	2.973267	-4.181969	3.475305
C	2.707689	-5.099701	4.671729
H	1.640402	-5.312040	4.787167
H	3.242024	-6.046789	4.561586
H	3.074298	-4.644632	5.595505
C	4.495483	-3.924543	3.365790
H	4.865932	-3.445238	4.278264
H	5.029954	-4.870055	3.225511
H	4.726993	-3.272621	2.517367
C	-1.668900	0.465958	-1.544845
C	-3.619337	1.050155	-0.220578
C	-1.390779	2.382094	3.866434
H	-0.555248	2.962247	3.463403
H	-2.225732	3.059634	4.067098
H	-1.087670	1.889095	4.791606
C	-7.583971	3.273539	1.661793
H	-8.088134	2.568422	0.997303
H	-7.629188	2.872149	2.678492
H	-8.130065	4.222370	1.646976
C	-5.398632	4.292859	2.328886
H	-5.859950	5.273904	2.476791
H	-5.446169	3.745230	3.275036
H	-4.344743	4.418889	2.069707
C	-5.704156	5.553218	-0.262712
H	-5.567511	5.858876	-1.304330
H	-6.568393	6.092374	0.138675
H	-4.815244	5.848425	0.298289
C	-7.045501	3.644067	-1.159893
H	-7.986565	4.152322	-0.930583
H	-6.747455	3.912278	-2.177441
H	-7.216282	2.563483	-1.133681
C	-2.768555	0.627837	0.970264
H	-3.154660	-0.201658	1.558973
C	-2.688928	1.514473	-1.372104
H	-2.203736	2.440151	-1.059105
H	-3.203444	1.710773	-2.319747
O	-0.935072	-2.290120	0.920982
C	2.704878	6.339848	-3.026764
C	3.642734	7.783669	-1.385219
C	2.867386	7.824661	-2.704746
H	1.764825	6.111675	-3.537698
H	3.535667	5.969608	-3.641226
H	3.539443	8.694759	-0.791944
H	4.708185	7.606204	-1.570592
H	1.889172	8.292026	-2.553036
H	3.387858	8.364212	-3.498813
C	5.848255	3.331135	0.628108
C	6.517875	5.537679	0.004142
C	6.102311	4.706063	1.233359

H	6.786914	2.772507	0.494387
H	5.148610	2.710424	1.192134
H	7.604556	5.636216	-0.056673
H	6.092819	6.543995	0.032206
H	6.864538	4.688481	2.015513
H	5.173021	5.091317	1.665640
O	5.262918	3.615859	-0.637270
C	5.982021	4.719046	-1.190379
H	5.286314	5.274346	-1.824415
H	6.804082	4.344669	-1.814630
C	3.014546	6.573523	-0.709082
H	3.671949	6.073566	0.009580
H	2.081481	6.846140	-0.197591
O	2.729514	5.651925	-1.76615

References

1. Doan, S. H.; Ton, N. N.; Mai, B. K.; Nguyen, T. V. Organosuperbase-Catalyzed 1,1-Diboration of Alkynes, *ACS Catalysis,* **2022**, *12*, 12409–12418.
2. Pujol, M.; Méndez, M.; Fernández, E. Stereoselective Control of the Cu Activation of β,β-Diboryl Acrylates for Allylic Coupling Protocols with Concomitant Lactonization, *Org. Lett.* **2024**, *26,* 2821-2826.
3. Dominguez-Molano, P.; Solé-Daura, A.; Carbó, J. J.;Fernández, E. Remote 1,4-Carbon-to-Carbon Boryl Migration: From a Mechanistic Challenge to a Valuable Synthetic Application of Bicycles *Adv.Sci*. **2024**, *11*, 2309779.
4. Gao, C.; Tang, K.; Yang, X.; Gao, S.; Zheng, Q.;Chen, X.; Liu J. Cu-Catalyzed Diastereo- and Enantioselective Synthesis of Borylated Cyclopropanes with Three Contiguous Stereocenters. *J. Am. Chem. Soc*. **2025**, *147*, 3360–3370.
5. M. J. Frisch, G. W. Trucks, H. B. Schlegel, G. E. Scuseria, M. A. Robb, J. R. Cheeseman, G. Scalmani, V. Barone, G. A. Petersson, H. Nakatsuji, X. Li, M. Caricato, A. V. Marenich, J. Bloino, B. G. Janesko, R. Gomperts, B. Mennucci, H. P. Hratchian, J. V. Ortiz, A. F. Izmaylov, J. L. Sonnenberg, D. Williams-Young, F. Ding, F. Lipparini, F. Egidi, J. Goings, B. Peng, A. Petrone, et al., Gaussian, 16, Rev A.03 Wallingford, CT 2016.
6. R. G. Parr, W. Yang, *Density-Functional Theory of Atoms and Molecules*, Oxford University Press, New York, Oxford 1989.
7. Chai, J.-D.; Head-Gordon, M. Long-Range Corrected Hybrid Density Functionals with Damped Atom–Atom Dispersion Corrections. *Physical Chemistry Chemical Physics* **2008**, *10*(44), 6615.
8. a) Hay, P. J.; Wadt, W. R. Ab Initio Effective Core Potentials for Molecular Calculations. Potentials for the Transition Metal Atoms Sc to Hg. *The Journal of Chemical Physics* **1985**, *82*(1), 270–283.; b) Wadt, W. R.; Hay, P. J. Ab Initio Effective Core Potentials for Molecular Calculations. Potentials for Main Group Elements Na to Bi.*The Journal of Chemical Physics* **1985**, *82*(1), 284–298.; c) Hay, P. J.; Wadt, W. R. Ab Initio Effective Core Potentials for Molecular Calculations. Potentials for K to Au Including the Outermost Core Orbitals. *The Journal of Chemical Physics* **1985**, *82*(1), 299–310.
9. A. Höllwarth; Böhme, M.; Dapprich, S.; Ehlers, A. W.; Gobbi, A.; Jonas, V.; Karen Francine Köhler; Stegmann, R.; Veldkamp, A.; Gernot Frenking. A Set of D-Polarization Functions for Pseudo-Potential Basis Sets of the Main Group Elements Al-Bi and f-Type Polarization Functions for Zn, Cd, Hg. **1993**, *208* (3-4), 237–240.
10. Hariharan, P. C.; Pople, J. A. The Influence of Polarization Functions on Molecular Orbital Hydrogenation Energies. *Theoretica Chimica Acta* **1973**, *28* (3), 213–222.
11. a) Gordon, M. S. The Isomers of Silacyclopropane. *Chemical Physics Letters* **1980**, *76*(1), 163–168.; b) Binning, R. C.; Curtiss, L. A. Compact Contracted Basis Sets for Third-Row Atoms: Ga-Kr. *Journal of Computational Chemistry* **1990**, *11*(10), 1206–1216.; c) McLean, A. D.; Chandler, G. S. Contracted Gaussian Basis Sets for Molecular

Calculations. I. Second Row Atoms, *Z*=11–18. *The Journal of Chemical Physics* **1980**, *72*(10), 5639–5648.

12. *A New Basis Set Exchange: An Open, Up-to-date Resource for the Molecular Sciences Community.* Benjamin P. Pritchard, Doaa Altarawy, Brett Didier, Tara D. Gibson, Theresa L. Windus. *J. Chem. Inf. Model.* **2019**, *59(11)*, 4814-4820
13. Marenich, A. V.; Cramer, C. J.; Truhlar, D. G. Universal Solvation Model Based on Solute Electron Density and on a Continuum Model of the Solvent Defined by the Bulk Dielectric Constant and Atomic Surface Tensions. *The Journal of Physical Chemistry B* **2009**, *113*(18), 6378–6396.
14. Luchini G.; Alegre-Requena J. V.; Funes-Ardoiz I.; Paton R. S. GoodVibes: automated thermochemistry for heterogeneous computational chemistry data [version 1; peer review: 2 approved with reservations]. *F1000Research* 2020, **9**(Chem Inf Sci):291 (https://doi.org/10.12688/f1000research.22758.1)
15. Ehlers, A. W.; Böhme, M.; Dapprich, S.; Gobbi, A.; A. Höllwarth; Jonas, V.; Karen Francine Köhler; Stegmann, R.; Veldkamp, A.; Gernot Frenking. A Set of F-Polarization Functions for Pseudo-Potential Basis Sets of the Transition Metals Sc-Cu, Y-Ag and La-Au. *Chemical Physics Letters* **1993**, *208* (1-2), 111–114.
16. Roy, L. E.; P. Jeffrey Hay; Martin, R. L. Revised Basis Sets for the LANL Effective Core Potentials. *Journal of Chemical Theory and Computation* **2008**, *4*(7), 1029–1031.
17. Álvarez-Moreno, M.; de Graaf, C.; López, N.; Maseras, F.; Poblet, J. M.; Bo, C. Managing the Computational Chemistry Big Data Problem: The IoChem-BD Platform. *Journal of Chemical Information and Modeling* **2014**, *55*(1), 95–103.
